# Identification of boholamide A analogue as a potential hypoxia-selective anti-triple-negative breast cancer agent by targeting eEF1A1

**DOI:** 10.1016/j.apsb.2026.02.009

**Published:** 2026-02-11

**Authors:** Guangju Liu, Fengyuan Zhang, Fangzhi Han, Sisi Chen, Junjie Ou, Renfeng Qiao, Yixuan Jiang, Shaojie Miao, Wei Lin, Yahui Ding, Quan Zhang

**Affiliations:** a State Key Laboratory of Medicinal Chemical Biology, College of Pharmacy and Tianjin Key Laboratory of Molecular Drug Research, Nankai University, Tianjin 300353, China; b College of Chemistry, Nankai University, Tianjin 300071, China

**Keywords:** Boholamide A analogue, Hypoxia selectivity, eEF1A1, Triple-negative breast cancer

## Abstract

Triple-negative breast cancer (TNBC) is one of the most aggressive and metastatic forms of breast cancer, for which there are currently no satisfactory therapeutic agents. Here, we reported for the first time that boholamide A, a naturally occurring macrocyclic depsipeptide, exhibited hypoxia-selective anti-TNBC activity against in MDA-MB-231 cells. However, its structure‒activity relationships and target had not yet been elucidated. A series of boholamide A analogues were chemically synthesized and evaluated for anti-TNBC potency. The most promising compound **1j** was prepared in 13 linear steps with an overall yield of 7.92%, and exhibited high potency against MDA-MB-231 cells with an IC_50_ value of 0.15 μmol/L under hypoxic condition. Moreover, **1j** significantly inhibited proliferation and migration, and induced apoptosis in MDA-MB-231 cells. Compound **16**, a prodrug of **1j**, significantly inhibited the tumor volume and tumor weight in xenografts. Furthermore, we identified that **1j** covalently targeted eukaryotic translation elongation factor 1alpha 1 (eEF1A1) which might underlie the anticancer activity and hypoxia selectivity of boholamide A analogues. These results suggested that boholamide A analogues represented a promising scaffold for discovering hypoxia-selective anti-TNBC agents, and compound **16** deserved further investigation as a candidate for TNBC treatment.

## Graphical abstract

The SAR of boholamide A at multiple sites was explored by total synthesis. The optimized compound **1j** potently targeted eEF1A1 and inhibited triple-negative breast cancer growth *in vivo*.

**Figure:**
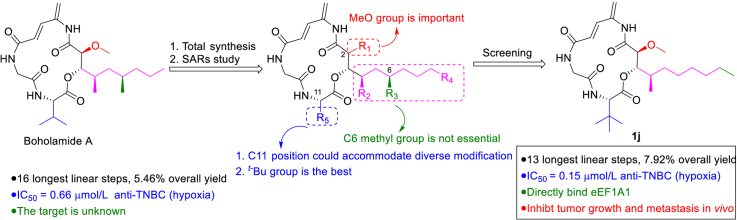

## 1 Introduction

Breast cancer is the second leading cause of cancer-related death among women in the United States, following lung cancer, and accounts for 32% of all newly diagnosed female cancers each year^1^. Triple-negative breast cancer (TNBC) is one of the most aggressive subtypes of breast cancer with a worse prognosis than other breast cancer types^2^. Its biological characteristics include high proliferative activity, increased immune cell infiltration, basal-like and mesenchymal phenotype, and homologous recombination deficiency, which is partly associated with loss of BRCA1 or BRCA2 function^3^, ^4^, ^5^. TNBC is a heterogeneous disease for which no effective targeted therapies are currently available. Conventional chemotherapy remains the common choice in clinical practice, yet it reduces the risk of recurrence by only approximately 30% in specific patient populations^6^. Due to the lack of effective therapeutic agents, the median overall survival of TNBC patients remains poor, at less than 18 months^7^. Therefore, novel compounds with a unique mechanism are urgently needed to address this clinical challenge.

**Figure 1:**
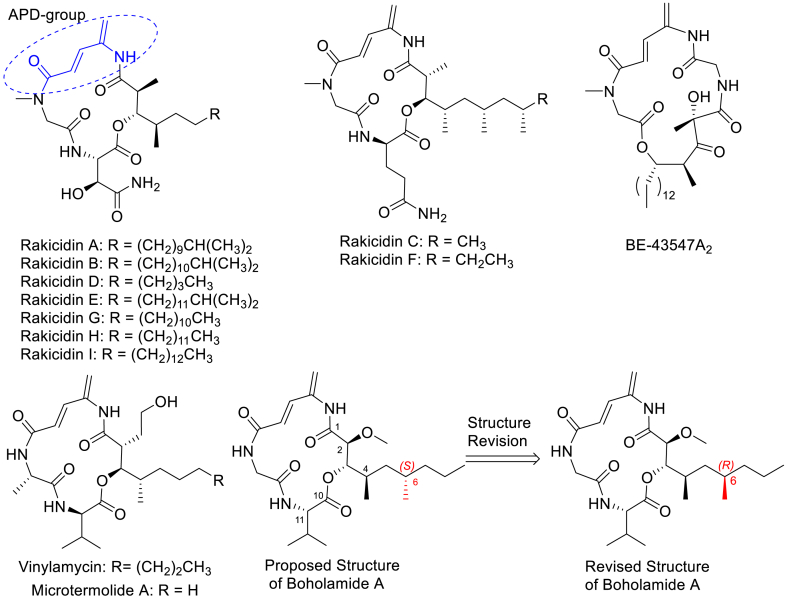
Structures of cyclodepsipeptides containing APD moiety.

Boholamide A is a macrocyclic depsipeptide featuring a 4-amino-2,4-pentadienolate (APD) moiety^8^. The unique APD moiety is found in only a few natural products, such as rakicidin (A–I)^9^, ^10^, ^11^, ^12^, ^13^, ^14^, vinylamycin^15^, microtermolide A^16^, and BE-43547^17^^,^^18^ (Fig. 1). Due to their unique structures and hypoxia-selective anticancer activity, these compounds have attracted considerable attention from our research group and other investigators^19^, ^20^, ^21^, ^22^, ^23^, ^24^, ^25^, ^26^, ^27^, ^28^, ^29^, ^30^, ^31^, ^32^. We previously reported the first total synthesis of boholamide A *via* a 16 step linear route with an overall yield of 5.46%, which prompted a revision of the C6 stereochemistry in the previously proposed structure of natural boholamide A^33^.

Recently, an in-house natural products library screening for potential anti-TNBC agents led to the identification of boholamide A (Fig. 1) as an anti-TNBC compound with moderate activity. However, its structure‒activity relationships and molecular target remained unknown. Given its structural novelty compared to previously reported anti-TNBC agents, we employed an efficient combinatorial approach to synthesize a series of boholamide A analogues and evaluated their anti-cancer activity. Notably, the most promising analogue, compound **1j** exhibited high selectivity against TNBC cells under hypoxic conditions and low cytotoxicity against normal cells. To reveal the target of compound **1j**, we synthesized probe **15** and performed target identification studies. The results indicated that compound **1j** directly bound eukaryotic translation elongation factor 1alpha 1 (eEF1A1), and its target protein exhibited complete identity to the natural product BE-43547A_2_ which possessed an APD structure. Mechanistic studies revealed that compound **1j** disrupted the eEF1A1/FoxO1 interaction, promoted FoxO1 nuclear translocation and consequently suppressed the JAK/STAT3 pathway. Furthermore, compound **16** which was a prodrug of **1j**, significantly inhibited the tumor volume and tumor weight in a dose-dependent manner and inhibited breast cancer cell metastasis *in vivo*.

## 2 Results and discussion

### 2.1 The synthesis of boholamide A analogues

**Scheme 1:**
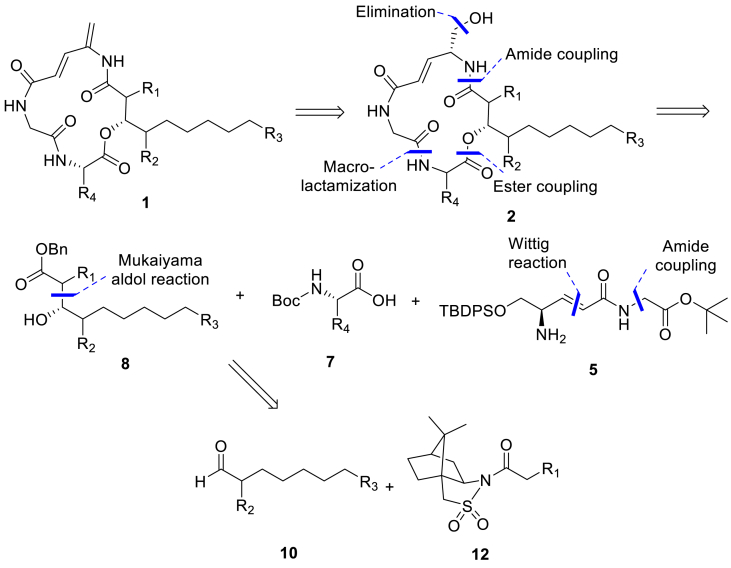
Retrosynthetic analysis of boholamides (**1**).

Scheme 1 illustrates the retrosynthetic analysis of boholamides (**1**). Boholamides was synthesized by elimination of alcohol **2**. Alcohol **2** could be disconnected into three fragments: alcohol **8**, amino acid fragment **7**, and amine fragment **5**. Alcohol **8** could be prepared by a Mukaiyama aldol reaction of D-camphorsultam derivative **12** with aldehyde **10**. Amine fragment **5** was prepared following our previously reported procedure^33^, while amino acid fragment **7** was commercially available.

**Scheme 2:**
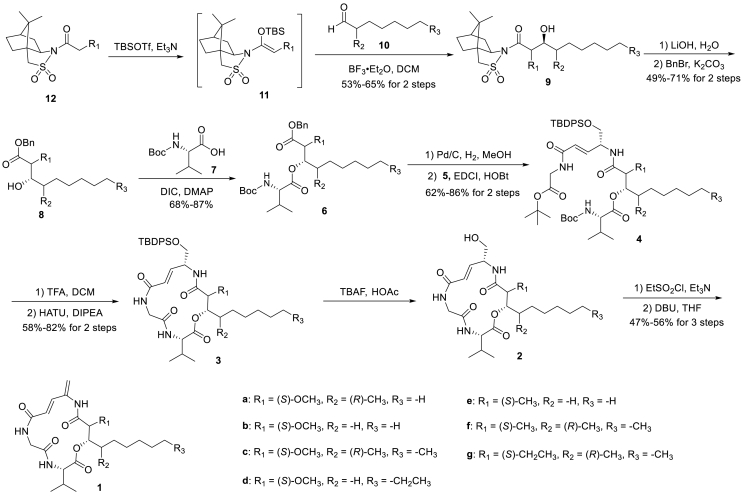
Total synthesis of boholamide A analogues **1a**‒**1g.**

Our and other groups’^23^, ^24^, ^25^, ^26^ studies demonstrated that APD moiety served as the pharmacophore for APD-containing cyclolipodepsipeptides. Therefore, we retained the APD moiety and attempted to modify the long lipophilic chain and the unique methoxy group of alcohol **8** in the first-round modification, and designed a total of seven analogues. The synthetic route is shown in Scheme 2.

The d-camphorsultam derivative **12a**–**12g** was converted to a silyl enol ether **11a**–**11g** using TBSOTf/Et_3_N. Mukaiyama aldol reaction of **11a**–**11g** with aldehydes **10a**–**10g** under BF_3_-mediated condition afforded compounds **9a**–**9g** (dr > 30:1, according to ^1^H NMR). After the hydrolysis of compounds **9a**–**9g** with LiOH, the alcohol **8a**–**8g** was obtained after protection with benzyl group. The alcohol **8a**–**8g** was then esterified with the amino acid fragment **7** under DIC/DMAP. Compound **6a**–**6g** was hydrogenated under Pd/C and coupled with amine fragment **5** to form the ring precursor **4a**–**4g**. Compound **5** was synthesized following our previously reported procedure^33^. After removal of the two protective groups by 20% TFA, compound **3a**–**3g** was obtained by macro-lactamization using HATU/DIPEA. Deprotection of TBDPS with TBAF/HOAc yielded alcohol **2a**–**2g**, which were activated with an ethyl sulfonyl group and eliminated with DBU to afford boholamide A analogues **1a**–**1g**.

**Scheme 3:**
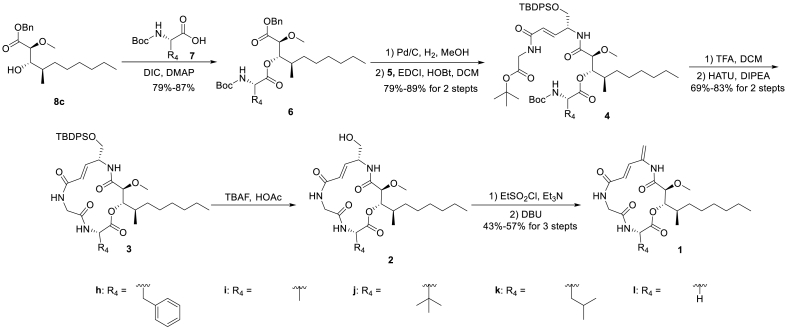
Total synthesis of boholamide A analogues **1h**‒**1l**.

As shown in Table 1, boholamide A analogue **1c** exhibited the most potent anti-TNBC activity, comparable to that of natural product. Analogue **1c** was prepared in 13 linear steps with 6.40% overall yield, representing an improvement over boholamide A, which required 16 linear steps and 5.46% overall yield. Accordingly, in the second-round modification, we started with chiral alcohol **8c**, changed the amino acid fragment **7**, and synthesized a total of five boholamide A analogues **1h**–**1l** with modification at the C-11 site. The synthetic route was shown in Scheme 3.

**Table 1:** Anti-proliferation activity of boholamide analogues **1a**‒**1g** against cancer cells.^a^

Compd.	Structure	MDA-MB-231			U118			A549		
		IC_50_ (μmol/L)		HSI^b^	IC_50_ (μmol/L)		HSI	IC_50_ (μmol/L)		HSI
		Normoxia	Hypoxia		Normoxia	Hypoxia		Normoxia	Hypoxia	
Boholamide A		2.78 ± 0.32	0.66 ± 0.18	4.21	4.69 ± 1.04	1.44 ± 0.62	3.26	7.53 ± 1.17	2.44 ± 0.77	3.09
**1a**		4.85 ± 1.65	2.27 ± 1.17	2.14	8.64 ± 3.44	4.09 ± 0.08	2.11	12.37 ± 3.81	3.01 ± 1.31	4.11
**1b**		8.87 ± 6.20	5.68 ± 1.44	1.56	17.83 ± 8.68	13.14 ± 5.33	1.36	26.22 ± 8.09	11.08 ± 1.31	2.37
**1c**		2.02 ± 0.41	1.24 ± 0.24	1.63	3.42 ± 1.49	1.55 ± 0.39	2.21	4.09 ± 0.88	1.30 ± 0.55	3.14
**1d**		6.14 ± 2.77	3.14 ± 1.37	1.96	14.87 ± 8.37	10.29 ± 2.77	1.45	15.72 ± 4.81	8.58 ± 2.55	1.83
**1e**		＞20	16.34 ± 7.09	>1.22	＞50	＞20	‒	＞50	22.62 ± 13.71	>2.21
**1f**		5.93 ± 0.17	2.38 ± 0.99	2.49	7.50 ± 0.85	4.49 ± 2.56	1.67	8.46 ± 1.75	2.51 ± 1.98	3.37
**1g**		4.36 ± 2.23	3.58 ± 1.92	1.21	7.52 ± 3.23	6.36 ± 2.35	1.18	10.16 ± 2.40	3.43 ± 2.38	2.96

a All values were the means of three independent experiments.

b HSI was calculated from: IC_50_ (normoxia)/IC_50_ (hypoxia).

To explore the biological mechanism of boholamide A analogues, probes **1m** and **15** were designed and synthesized (Fig. 5A). Compound **13** was prepared from alcohol **8c** by removal of the benzyl group followed by allyl protection. Coupling of **13** with amino acid **7m** gave **6m**. The allyl group of **6m** was removed by Pd(PPh_3_)_4_, and the resulting intermediate was coupled with amine fragment **5** to afford ring precursor **4m**. Compound **4m** was converted to compound **1m** following the same synthetic route. A click reaction between **1m** and commercially available **14**, catalyzed by Cu_2_O nanoparticles, yielded the boholamide A probe **15**^34^. Compound **16**, a prodrug of **1j,** was obtained by nucleophilic substitution of methyl piperazine with ethanesulfonyl chloride-activated alcohol **2j** (Supporting Information Fig. S1A).

**Figure 2:**
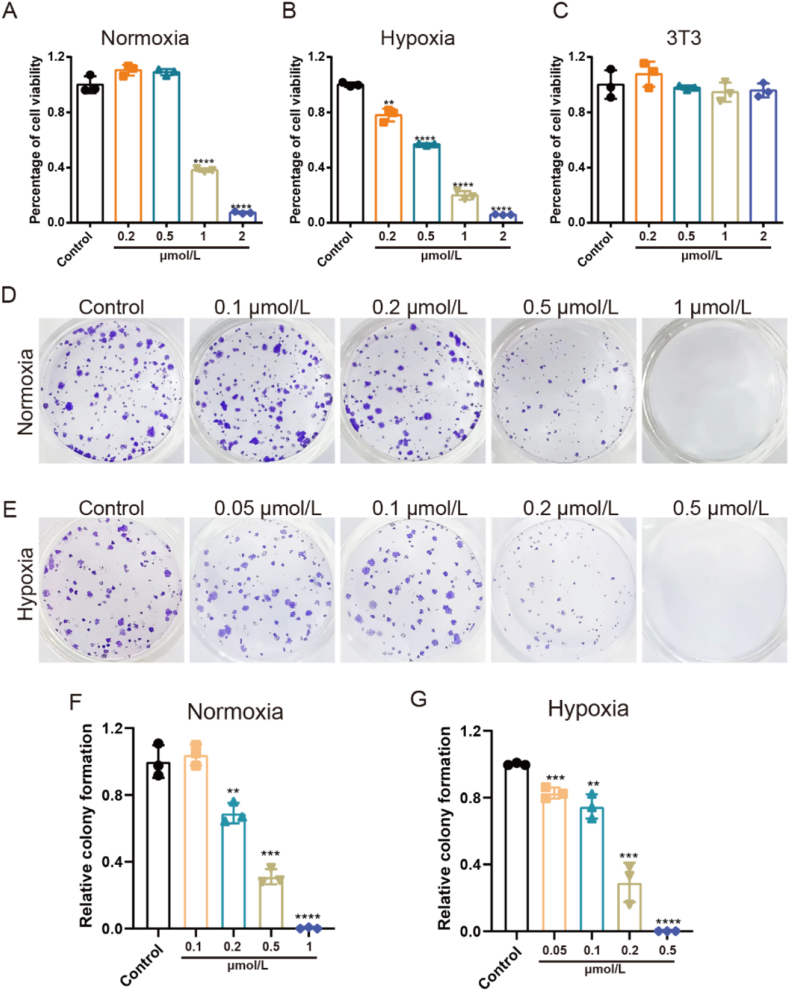
The antiproliferative activity of **1j** against MDA-MB-231 cells. (A, B) The antiproliferative activity against MDA-MB-231 cells of **1j** under normoxia and hypoxia. (C) The antiproliferative activity against normal 3T3 cells under normoxia. (D, F) The representative picture and colony formation rate statistical results after treatment of compound **1j** with different concentrations under normoxia. (E, G) The representative picture and colony formation rate statistical results after treatment of compound **1j** with different concentrations under hypoxia. ∗*P* < 0.05, ∗∗*P* < 0.01, ∗∗∗*P* < 0.005, ∗∗∗∗*P* < 0.001. Data presented as means ± SD (*n* = 3).

**Figure 3:**
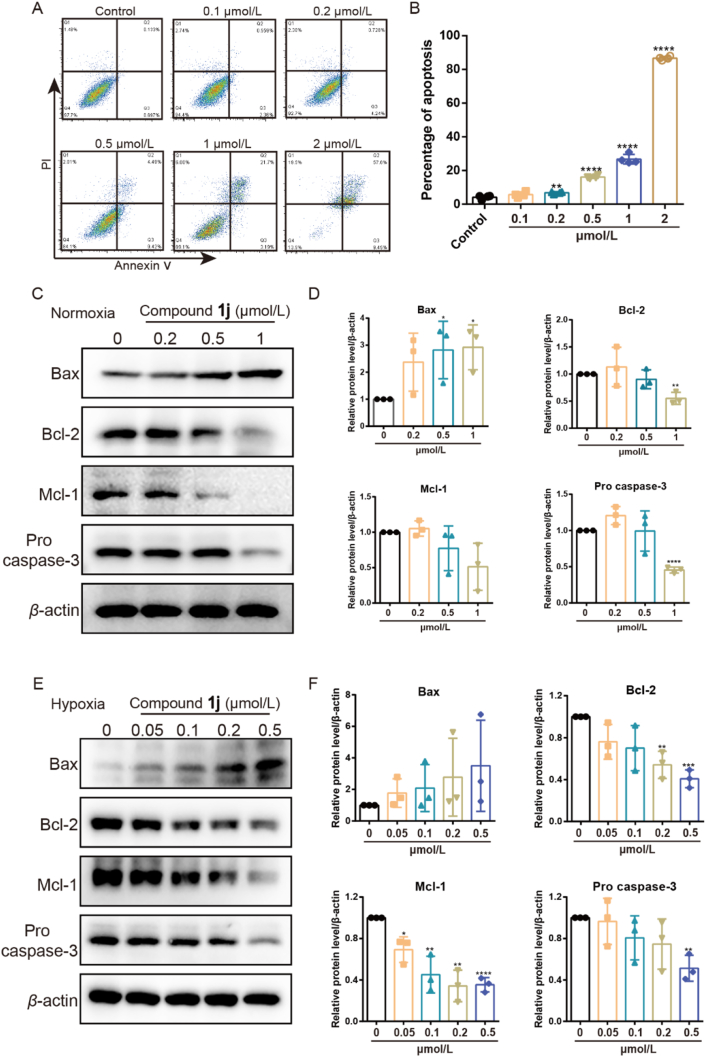
Compound **1j** induced apoptosis of TNBC cells through mitochondrial pathway. (A, B) The representative of apoptosis picture and statistical results after treatment of compound **1j** with different concentrations under hypoxia. Data presented as means ± SD (*n* = 4). (C, D) The level of apoptosis related proteins after treatment of compound **1j** at different concentrations under normoxia. (E, F) The level of apoptosis related proteins after treatment of compound **1j** at different concentrations under hypoxia. ∗*P* < 0.05, ∗∗*P* < 0.01, ∗∗∗*P* < 0.005, ∗∗∗∗*P* < 0.001. Data presented as means ± SD (*n* = 3).

**Figure 4:**
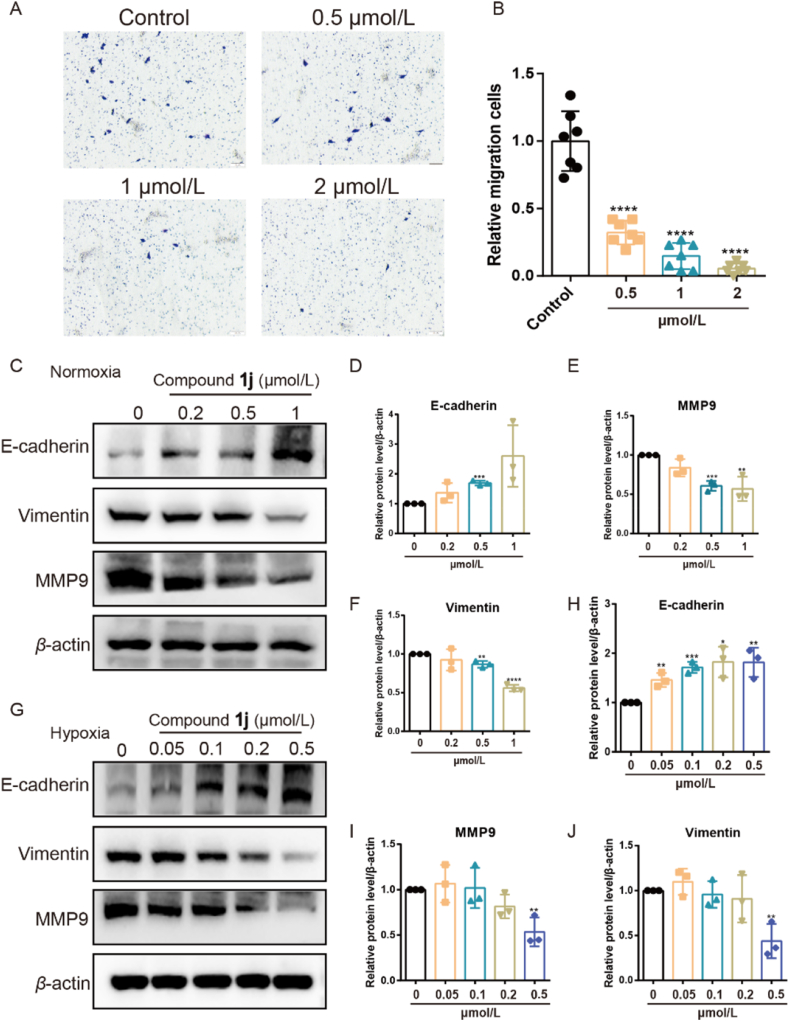
Compound **1j** inhibited migration of TNBC cells. (A, B) The representative images of migration cells and statistical results after treatment of compound **1j** at different concentrations under hypoxia. Data presented as means ± SD (*n* = 7). (C–F) The level of migration related proteins after treatment of compound **1j** at different concentrations under normoxia. (G–J) The level of migration related proteins after treatment of compound **1j** at different concentrations under hypoxia. ∗*P* < 0.05, ∗∗*P* < 0.01, ∗∗∗*P* < 0.005, ∗∗∗∗*P* < 0.001. Data presented as means ± SD (*n* = 3).

**Figure 5:**
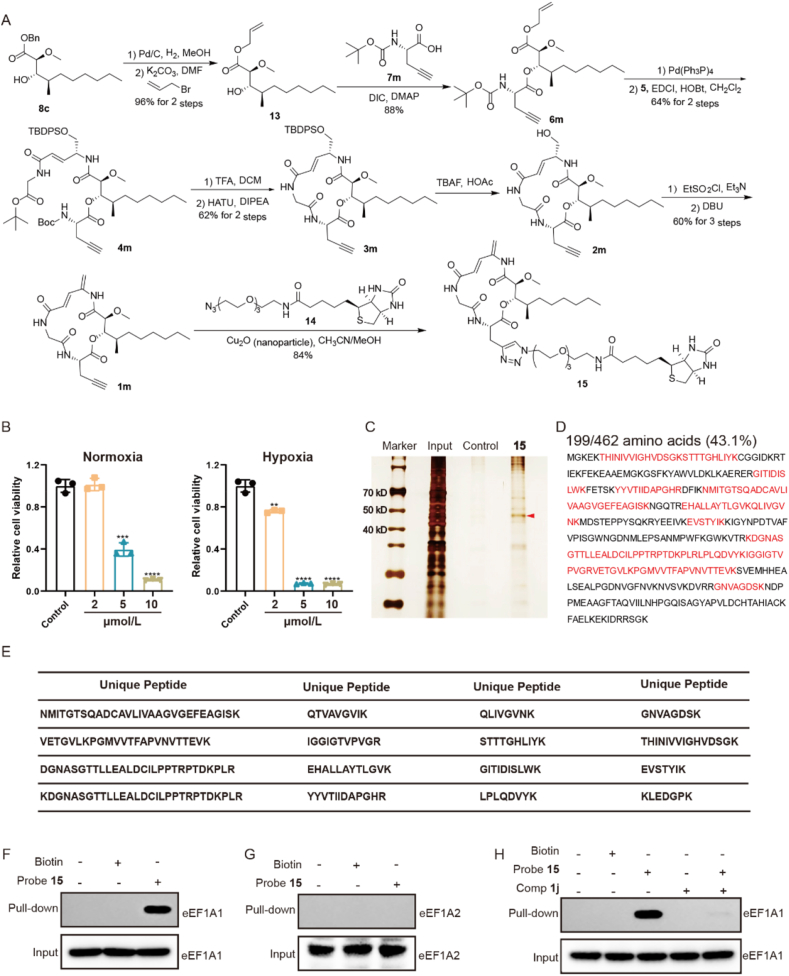
Boholamide A analogue directly targeted eEF1A1. (A) The synthesis route of **1m** and probe **15**. (B) The antiproliferative activity of **1m** against MDA-MB-231 cells under normoxia and hypoxia. (C) The silver stain picture of the protein enriched by probe **15**. (D) The peptide coverage of eEF1A1 after LC‒MS/MS analysis. (E) The unique peptides of eEF1A1 detected by LC‒MS/MS analysis. (F) Western blot analysis to detect the binding of eEF1A1 using probe **15** in cell lysates. (G) Western blot analysis to detect the binding of eEF1A2 using probe **15** in cell lysates. (H) Western blot analysis to detect the competitive binding of probe **15** and **1j** to eEF1A1 protein. ∗*P* < 0.05, ∗∗*P* < 0.01, ∗∗∗*P* < 0.005, ∗∗∗∗*P* < 0.001. Data presented as means ± SD (*n* = 3).

### 2.2 Structure‒activity relationship of boholamide A analogues

With the synthesized boholamide A analogues in hand, we first evaluated their effects on human triple-negative breast cancer cell line MDA-MB-231, the human glioblastoma cell line U118 and the human lung cancer cell line A549 under both normoxic and hypoxic conditions. The hypoxia-selective index (HSI) value was calculated by IC_50_ (normoxia)/IC_50_ (hypoxia) for evaluating their potency of hypoxia-selective effect. The natural product boholamide A was also included for comparison.

As indicated in Table 1, all the synthesized analogues **1a**–**1g** exhibited higher potency against MDA-MB-231 cells than against U118 and A549 cells, and showed greater anti-cancer activity under hypoxia than under normoxia. The natural product boholamide A demonstrated potent activity against MDA-MB-231 cells (IC_50_ = 2.78 μmol/L under normoxia, IC_50_ = 0.66 μmol/L under hypoxia, HSI = 4.21). Boholamide A also displayed hypoxia-selective activity against U118 and A549 cells with HSI value of 3.26 and 3.09, respectively. Removal of the C6 methyl group yielded compound **1a**, which was less active than boholamide A against MDA-MB-231, U118 and A549 cells under both normoxia and hypoxia. Further removal of the C4 methyl group produced compound **1b**. The anti-TNBC potency of **1b** (IC_50_ = 8.87 μmol/L under normoxia, IC_50_ = 5.68 μmol/L under hypoxia) was greatly reduced comparing with boholamide A (IC_50_ = 2.78 μmol/L under normoxia, IC_50_ = 0.66 μmol/L under hypoxia), especially under hypoxia. The HSI value was also decreased (4.21 *vs* 1.56). Repositioning the C6 methyl group to the terminal position of the long lipophilic chain generated compound **1c**, which was the most active analogue in series **1a**–**1g** against all three cell lines under both conditions However, **1c** exhibited slightly less potent activity against MDA-MB-231 cells compared with boholamide A under hypoxia, and the HSI index was also reduced (1.63 *vs* 4.21). Further relocation of the C4 methyl group to the chain terminus (**1d**) significantly diminished anti-cancer activity against all cell lines compared with **1c**. The sub-series of **1e**–**1g** was modified at the C2 position. Replacement of the methoxyl group in **1b** and **1c** with a methyl group afforded **1e** and **1f**, respectively. Compounds **1e** and **1f** showed weaker anti-cancer effect than **1b** and **1c** which indicated that the methoxyl group was important for their high anti-cancer activities.

Compound **1c** was selected as a new lead compound for further structure-activity relationship studies. Five boholamide A analogues **1h**–**1l** were rapidly synthesized using a combinatorial strategy to explore the effect of the bottom amino acid fragment on the anti-proliferative activity. The anti-proliferative activities of these boholamide analogues against different tumor cells were summarized in Table 2. Compounds **1h**–**1l** exhibited comparable inhibitory effects on MDA-MB-231 cells under hypoxia, indicating that this site could accommodate diverse modifications. Among these, compound **1j** displayed the most potent activity against MDA-MB-231 cells, with an IC_50_ value of 0.15 μmol/L under hypoxia, which was 8.3-fold more potent than **1c**. Moreover, the HSI of **1j** was substantially higher than that of **1c** for MDA-MB-231 (6.73 *vs* 1.63). Notably, **1j** (IC_50_ = 1.01 μmol/L under normoxia, IC_50_ = 0.15 μmol/L under hypoxia) was more potent than natural product boholamide A (IC_50_ = 2.78 μmol/L under normoxia, IC_50_ = 0.66 μmol/L under hypoxia) against MDA-MB-231 cells.

**Table 2:** Anti-proliferation activity of boholamide analogues **1h**–**1l** against cancer cells.^a^

Compd.	Structure	MDA-MB-231			U118			A549		
		IC_50_ (μmol/L)		HSI^b^	IC_50_ (μmol/L)		HSI	IC_50_ (μmol/L)		HSI
		Normoxia	Hypoxia		Normoxia	Hypoxia		Normoxia	Hypoxia	
**1c**		2.02 ± 0.41	1.24 ± 0.24	1.63	3.42 ± 1.49	1.55 ± 0.39	2.21	4.09 ± 0.88	1.30 ± 0.55	3.14
**1h**		1.49 ± 0.38	0.88 ± 0.08	1.69	3.05 ± 1.42	2.88 ± 1.02	1.06	3.39 ± 0.95	1.40 ± 0.13	2.42
**1i**		7.51 ± 0.61	1.61 ± 0.36	4.66	15.24 ± 10.07	3.98 ± 1.79	3.83	＞50	4.71 ± 2.69	>10.61
**1j**		1.01 ± 0.23	0.15 ± 0.04	6.73	1.93 ± 0.70	1.00 ± 0.39	1.93	2.09 ± 0.66	0.97 ± 0.23	2.15
**1k**		1.50 ± 0.35	0.56 ± 0.03	2.68	2.67 ± 0.84	1.67 ± 0.68	1.60	2.63 ± 0.95	1.01 ± 0.15	2.60
**1l**		10.34 ± 1.38	2.67 ± 0.46	3.87	28.79 ± 8.66	12.08 ± 3.80	2.38	31.79 ± 6.53	10.88 ± 3.33	2.83

a All values were the means of three independent experiments.

b HSI was calculated from: IC_50_ (normoxia)/IC_50_ (hypoxia).

### 2.3 The antiproliferative activity of **1j** against MDA-MB-231 cells

Compound **1j** showed the most potent inhibitory activity against TNBC cells and excellent hypoxia selectivity. Accordingly, compound **1j** was selected for further investigation. To evaluate the selectivity of **1j** to cancer cells, an MTT assay was performed to compare its effects on breast cancer cells versus normal cells. The results indicated that **1j** significantly inhibited breast cancer cell proliferation in a dose-dependent manner, with greater potency under hypoxia than under normoxia (Fig. 2A and B). Moreover, compound **1j** exhibited selectivity for cancer cells with little inhibitory potency on normal 3T3 cells under normoxia (Fig. 2C), which indicated that **1j** would show less toxic side effects. Furthermore, to assess the long-term cytotoxicity of **1j**, a colony formation assay was conducted. The results showed the number of colonies was clearly decreased after the treatment with **1j** in a dose-dependent manner under both normoxia and hypoxia (Fig. 2D–G).

### 2.4 Compound **1j** induced apoptosis of TNBC cells through mitochondrial pathway

To identify the mechanism of **1j** in inhibiting proliferation of TNBC cells, cell apoptosis was analyzed. The results demonstrated that **1j** induced apoptosis of TNBC cells in a dose-dependent manner. The percentage of apoptosis after the treatment of **1j** were 4.13 ± 1.23, 5.68 ± 1.61, 6.80 ± 0.64, 16.13 ± 0.82, 26.73 ± 2.84 and 86.68 ± 1.07 in MDA-MB-231 cells under hypoxia at a dose of 0, 0.1, 0.2, 0.5, 1 and 2 μmol/L, respectively (Fig. 3A and B). To further explain the mechanism of **1j** in inducing apoptosis of TNBC cells, the proteins related to mitochondrial pathway were detected. The results indicated that the apoptosis related protein Bax was significantly increased. Meanwhile, anti-apoptosis related proteins including Bcl-2, Mcl-1 were clearly decreased (Fig. 3C–F).

### 2.5 Compound **1j** inhibited migration of TNBC cells

To detect the effect of **1j** on migration of TNBC cells, a Transwell assay was performed. The results indicated that the migration cancer cells were greatly reduced after treatment with **1j** at different concentrations (Fig. 4A and B). Moreover, we detected the protein level related to migration of cancer cells including E-cadherin, Vimentin and MMP9. The results showed that Vimentin and MMP9 which promoted cancer metastasis were apparently decreased. E-cadherin which suppressed cancer metastasis was significantly increased under normoxia and hypoxia (Fig. 4C–J).

### 2.6 Boholamide A analogue directly targeted eEF1A1

To investigate the mechanism of boholamide A analogue in MDA-MB-231 cells, the probe **15** was designed and synthesized (Fig. 5A). Firstly, compound **1m** with an alkynyl group was synthesized and the anti-TNBC activity was detected. The result suggested the inhibitory effect and hypoxia selectivity of **1m** was maintained (Fig. 5B). Probe **15** containing a biotin moiety was then synthesized based on **1m**. We performed pull-down assay with probe **15** in cell lysates to precipitate the target protein of boholamide A analogue. After silver staining, a specific band with the molecular mass of about 50 kDa appeared (Fig. 5C). This band was excised, digested with trypsin and detected with LC‒MS/MS. The LC‒MS/MS analysis suggested a total of 16 unique peptides of eEF1A1 were detected, accounting for 43.1% of the total amino acids in the eEF1A1 protein (Fig. 5D and E). After fully considering molecular weight, number of unique peptides, peptide coverage, and excluding non-specific protein, we hypothesized that the direct target protein of **1j** might be eukaryotic translation elongation factor (eEF1A1).

To verify the binding of **1j** to eEF1A1 protein and to avoid non-specific binding, we performed Western blot assay and selected biotin fragment as a negative control. The results demonstrated that probe **15** specifically bound to eEF1A1 protein rather than biotin fragment (Fig. 5F). Given that eEF1A2 and eEF1A1 share 98% amino acid similarity^37^, we further assessed the binding specificity of **1j** toward eEF1A1 relative to eEF1A2 by conducting a Western blot assay with an eEF1A2-specific antibody. The results indicated that probe **15** exhibited significantly stronger specific binding to eEF1A1 than to eEF1A2 in the same condition (Fig. 5G). Moreover, competitive binding assays confirmed that **1j** competed specifically for binding to eEF1A1, indicating that eEF1A1 was a specific target of **1j** (Fig. 5H).

### 2.7 Boholamide A analogue bound to Cys31 of eEF1A1

**Figure 6:**
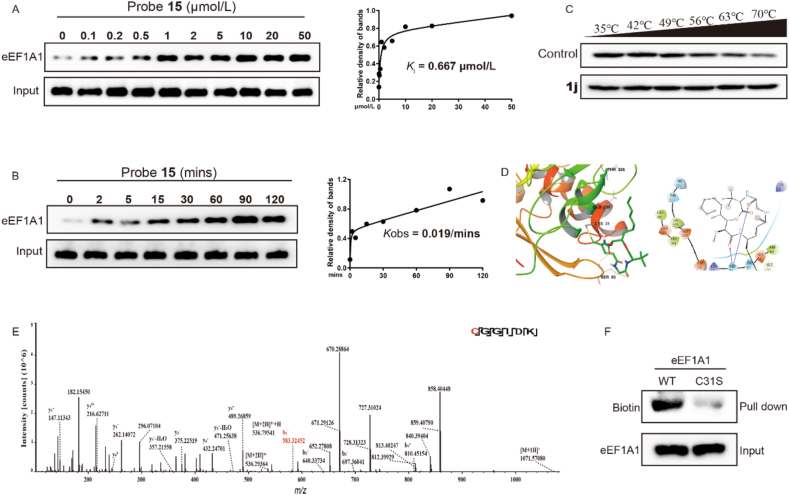
Boholamide A analogue bound to Cys31 of eEF1A1. (A) The specific binding *K*_i_ analysis of probe **15** and eEF1A1 using cell lysates and analyzed by Western blot assay. (B) The *K*_obs_ determination between probe **15** (10 μmol/L) and eEF1A1 protein using cell lysate and analyzed by Western blot assay. (C) The CESTA analysis using cell lysis after treated with **1j**. (D) The covalent docking between **1j** and Cys31 of eEF1A1 protein. (E) LC–MS/MS analysis of recombinant eEF1A1 peptide after incubated with **1j**. (F) Western blot analysis of the binding affinity after incubating probe **15** with wild-type or mutant eEF1A1 protein respectively.

We then performed a pull-down assay with probe **15** at different concentrations in cell lysates, and the binding affinity of probe **15** with eEF1A1 was verified with Western blot. The binding of probe **15** with eEF1A1 exhibited a dose-dependent manner with the *K*_i_ value of 0.667 μmol/L (Fig. 6A). Furthermore, the binding of eEF1A1 with probe **15** indicated a time-dependent saturation curve with the *K*_obs_ value of 0.019/min, which was consistent with an irreversible binding mechanism (Fig. 6B). Cellular thermal shift assay (CETSA) suggested that **1j** increased the thermal stability of eEF1A1 protein, indicating the binding between **1j** and eEF1A1 protein (Fig. 6C). The target of **1j** was consistent with the target of BE-43547A_2_, an APD-containing cyclolipodepsipeptide^18^. Accordingly, we proposed that eEF1A1 might be the stem of hypoxia-selective anti-cancer activity of APD-containing cyclolipodepsipeptides.

Based on preliminary results and relevant study references on BE-43547A_2_, we hypothesized that **1j** covalently reacted with the thiol group of a cysteine residue in eEF1A1^18^. To identify the specific amino acid residue directly bound to **1j**, we co-incubated the water-soluble derivative of **1j** with recombinant eEF1A1 protein and subsequently performed LC‒MS/MS analysis. The results showed that the peptide CGGIDK (*m*/*z* = 536.29) was 479.299 Da larger after incubation, with the mass difference exactly matching the molecular weight of **1j**. Further analysis of this peptide showed the 479.299 Da mass shift occurred from fragment ion b1 to b5, indicating that the Cys31 residue was the site of covalent modification by **1j** (Fig. 6E). The covalent docking assay between **1j** and eEF1A1 protein suggested **1j** could covalently bind Cys31 of eEF1A1 protein as a Michael acceptor and Ser83 promoted the covalent bond formation by establishing hydrogen bonds with **1j** (docking score: −5.138) (Fig. 6D). To further validate the binding of **1j** to Cys31 of eEF1A1 protein, we mutated the Cys31 of eEF1A1 to serine and purified the mutant recombinant eEF1A1 protein. Then the Western blot assay was performed after incubating probe **15** with wild-type or mutant eEF1A1 protein respectively. The results showed the binding affinity of probe **15** was significantly reduced after Cys31 of eEF1A1 mutation which indicated that **1j** covalently bound to the eEF1A1 protein *via* Cys31 (Fig. 6F).

eEF1A1 was recognized as a translation elongation factor which catalyzed the GTP-dependent binding of aminoacyl-tRNA (aa-tRNA) to the ribosomeal A-site during the elongation phase of protein synthesis^38^. In addition to this canonical function, eEF1A1 exhibited noncanonical cellular functions to participate in diverse biological processes beyond translation^39^, ^40^. Until now, eEF1A1 has emerged as a key target for multiple therapeutic agents including compounds with non-APD structure. As reported, the natural product Neferine inhibited macrophage glycolytic reprogramming by directly targeting eEF1A1 and disrupting the eEF1A1/ARID3A/PKC-*δ* complex^41^. *N*-Cinnamoylpyrrole-derived alkaloids targeted eEF1A1 to achieve the anti-neuroinflammatory and antioxidative stress function^42^. Plitidepsin targeted the host protein eEF1A to inhibit SARS-CoV-2 replication and eEF1A could be a druggable target for antiviral research^43^. Besides that, the number of anti-cancer agents targeting eEF1A were growing, indicating that modulating this crucial translation elongation factor could exhibit therapeutic potential in cancer treatment^44^.

### 2.8 Boholamide A analogue disrupted the interaction between eEF1A1 and FoxO1 in hypoxia

**Figure 7:**
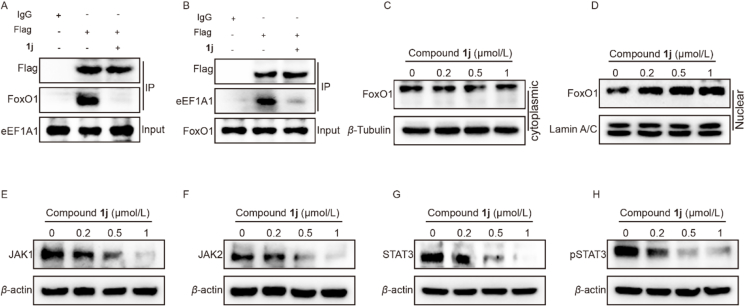
Boholamide A analogue disrupted the interaction between eEF1A1 and FoxO1 under hypoxia. (A) co-IP analysis of the interaction between eEF1A1 and FoxO1 after overexpressing Flag-tagged eEF1A1. (B) co-IP analysis of the interaction between eEF1A1 and FoxO1 after overexpressing Flag-tagged FoxO1. (C) The protein level of FoxO1 in cytoplasmic. (D) The protein level of FoxO1 in nuclear. (E–H) The protein level of JAK1, JAK2, STAT3 and pSTAT3 after treatment of **1j** under hypoxia for 48 h.

As reported, eEF1A1 was upregulated under hypoxic conditions and exhibited strong interaction with FoxO1, which is similarly induced by hypoxia. Furthermore, eEF1A1 inhibits the nuclear transport of FoxO1 by directly binding, thereby mediating the JAK/STAT3 signaling pathway^45^. To investigate whether **1j** disrupted the interaction between eEF1A1 and FoxO1 protein, we performed co-IP assay. First, we overexpressed Flag-tagged eEF1A1 in MDA-MB-231 cells and performed co-immunoprecipitation using an anti-Flag antibody. Treatment of **1j** markedly reduced the amount of FoxO1 co-immunoprecipitated with eEF1A1, indicating that **1j** disrupted the eEF1A1–FoxO1 interaction (Fig. 7A). Conversely, when Flag-tagged FoxO1 was overexpressed in MDA-MB-231 cells and subjected to anti-Flag co-immunoprecipitation, treatment of **1j** similarly led to a significant decrease in the amount of eEF1A1 pulled down with FoxO1 (Fig. 7B). These co-immunoprecipitation experiments collectively demonstrated that compound **1j** inhibited the association between eEF1A1 and FoxO1.

To verify whether compound **1j** promoted FoxO1 nuclear translocation by disrupting the eEF1A1/FoxO1 interaction and consequently suppressed the JAK/STAT3 pathway, we extracted nuclear proteins and cytoplasmic proteins separately from MDA-MB-231 cells with nuclear and cytoplasmic protein extraction kits to analyze the nuclear translocation of FoxO1. And the results suggested compound **1j** increased the level of FoxO1 in the nucleus and decreased the level of FoxO1 in the cytoplasm, indicating that compound **1j** significantly increased the nuclear translocation of FoxO1 (Fig. 7C and D).

We further investigated the effects of compound **1j** on the downstream JAK/STAT3 signal pathway. The results showed that **1j** significantly suppressed levels of JAK1, JAK2, STAT3 and phosphorylation of STAT3 in a dose-dependent manner (Fig. 7E–H). Collectively, these findings suggested **1j** inhibited the association between eEF1A1 and FoxO1, promoted FoxO1 nuclear translocation and consequently suppressed the JAK/STAT3 pathway.

### 2.9 The toxicity evaluation of boholamide A analogue

Since **1j** showed significant anti-TNBC activity *in vitro*, we decided to determine the anti-TNBC effect of **1j** *in vivo*. However, compound **1j** exhibited low water solubility, and then compound **1j** was transformed to its prodrug **16** following our previously reported strategy (Fig. S1A)^35^, ^36^. The results showed that the water solubility was clearly improved and the activity of compound **16** was maintained (Fig. S1B). The preliminary toxicity of compound **16** was evaluated in Balb/c mice. The results suggested no significant alterations were observed in body weight and organ coefficients including liver, spleen, lung, kidney, brain and heart compared with vehicle group after intraperitoneal administration of compound **16** at doses of 20, 40 and 80 mg/kg respectively (Fig. S1C and S1D). Furthermore, serum levels of aspartate aminotransferase (AST), alanine aminotransferase (ALT), and creatinine (CR) which were key indicators of hepatic and kidney function were not clearly increased after intraperitoneal administration of compound **16** at all tested doses, indicating no hepatotoxicity and nephrotoxicity (Fig. S1E). We performed histopathological analyses of major organs (including liver, kidney, heart, spleen, brain and lung) using hematoxylin and eosin (H&E) staining (Supporting Information Fig. S2). The results indicated no significant pathological abnormalities in any examined tissues at the tested doses, further supporting the safety profile of the compound **1j**. Collectively, these findings suggested that compound **16** exhibited no obvious toxicity *in vivo* at the tested doses.

### 2.10 Compound **16** inhibited tumor growth in xenografts

**Figure 8:**
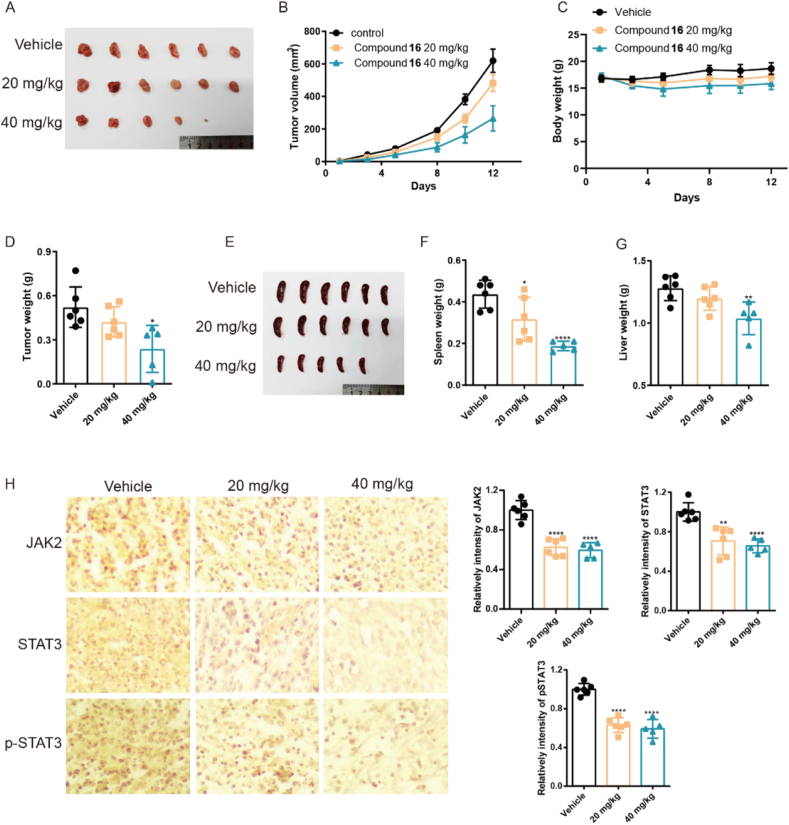
Compound **16** inhibited TNBC tumor growth in xenografts. (A–D) The tumor picture, tumor volume, body weight and tumor weight of mice after administration of prodrug **16**. (E–G) The spleen picture, spleen weight and liver weight of mice after administration of prodrug **16**. (H) The level of JAK2, STAT3 and pSTAT3 in tumor tissues. ∗*P* < 0.05, ∗∗*P* < 0.01, ∗∗∗*P* < 0.005, ∗∗∗∗*P* < 0.001. Data presented as means ± SD (*n* = 6 in vehicle and 20 mg/kg groups, *n* = 5 in 40 mg/kg group).

These results prompted us to evaluate the anti-TNBC efficacy of compound **16** *in vivo*. Firstly, mouse breast cancer cells 4T1 were cultured and transplanted into the mammary fat pad of Bal-b/c mice to establish breast cancer model. After the model was established, compound **16** was intraperitoneally administrated at different doses. The results indicated that compound **16** significantly inhibited the tumor volume and tumor weights in a dose-dependent manner (Fig. 8A, B, D). Hepatosplenomegaly is commonly associated with breast cancer metastasis. Notably, compound **16** significantly reduced the weight of both spleen and livers, which may suggest compound **16** could inhibit breast cancer metastasis *in vivo* (Fig. 8E–G). Moreover, although the mice exhibited a reduction in body weight after repeated administration of compound **16**, the toxicity remained manageable *in vivo* (Fig. 8C).

Furthermore, immunohistochemical analysis revealed that treatment with compound **16** significantly decreased the protein levels of JAK2, STAT3 and pSTAT3 in tumor tissues (Fig. 8H). All these results suggested that compound **16** inhibited tumor growth and metastasis *in vivo* through inhibiting JAK/STAT3 signal pathway.

## 3 Conclusions

In conclusion, boholamide A was identified as a lead compound for the discovery of potential hypoxia-selective anti-TNBC agents. A library of boholamide A analogues was synthesized through a highly efficient combinatorial strategy. Some preliminary structure‒activity relationships were concluded: (1) the C2 methoxyl group is important for the anti-TNBC activity; (2) the C6 methyl group is not essential, and relocating it to the terminus of the lipophilic chain retains potency; (3) the C11 position accommodates diverse modification. Among these, the most promising compound **1j** exhibited an IC_50_ value of 0.15 μmol/L, which was more potent than the parent natural product boholamide A for TNBC cells. Compound **1j** could selectively killed TNBC cells without apparent inhibitory effect on normal cells. Moreover, **1j** induced apoptosis and inhibited migration of TNBC cells. Meanwhile, eEF1A1 which was identified as the target of **1j** might be the stem of anticancer activity and hypoxia selectivity of boholamide A analogues. Furthermore, the binding site analysis suggested **1j** covalently bound Cys31 of eEF1A1 protein. The mechanism research showed **1j** significantly inhibited the association between eEF1A1 and FoxO1, promoted FoxO1 nuclear translocation and consequently suppressed the JAK/STAT3 pathway. These results suggested that boholamide A analogues might be a good source for discovery of hypoxia-selective anti-TNBC agents. Compound **16**, a prodrug of **1j**, exhibited no observable toxicity *in vivo* at the 20‒80 mg/kg doses. The *in vivo* efficacy research indicated compound **16** significantly inhibited the tumor volume, tumor weight and metastasis of TNBC with a dose-dependent manner in xenografts. These findings indicated that compound **16** may deserve further investigation as a promising lead compound for ultimate discovery of anti-TNBC drug.

## 4 Experimental

### 4.1 Chemistry

*General*. Unless otherwise mentioned, all reactions were carried out under an argon atmosphere with dry solvents under anhydrous conditions. The used solvents were purified and dried according to common procedures. Yields refer to chromatographically and spectroscopically (^1^H NMR) homogeneous materials, unless otherwise stated. Reagents were purchased at the highest commercial quality and used without further purification, unless otherwise stated. ^1^H NMR and ^13^C NMR were recorded on Bruker AV 400 and calibrated using internal references and solvent signals CDCl_3_ (*δ*_H_ = 7.26 ppm, *δ*_C_ = 77.16 ppm), DMSO-*d*_6_ (*δ*_H_ = 2.50 ppm, *δ*_C_ = 39.52 ppm). ^1^H NMR data are reported as follows: chemical shift, multiplicity (s = singlet, d = doublet, t = triplet, q = quartet, br = broad, m = multiplet), coupling constants and integration. High-resolution mass spectra (HRMS) were detected by FTICR-MS (Ion spec 7.0T) spectrometer.

*(2S,3S,4R)-1-((3aR,6R)-8,8-Dimethyl-2,2-dioxidotetrahydro-3H-3a,6-methanobenzo[c]isothiazol-1(4H)-yl)-3-hydroxy-2-methoxy-4-methylnonan-1-one(****9a****).* To a solution of compound **12a** (10 g, 34.80 mmol, 2.0 equiv) in CH_2_Cl_2_ (170 mL) was added triethylamine (9.86 g, 97.43 mmol, 2.8 equiv) and TBSOTf (23.90 g, 90.47 mmol, 2.6 equiv). The reaction mixture was stirred at room temperature for 12 h. All of the volatile materials were evaporated and extracted with hexane (3 × 40 mL). The combined organic phases were concentrated under reduced pressure to afford the desired compound **11a** as a yellow solid, which was dissolved in CH_2_Cl_2_ (50 mL) at–78 °C under argon atmosphere was added aldehyde **10a** (2.23 g, 1.0 equiv).

After stirred for 30 min, BF_3_•Et_2_O (2.72 g, 19.14 mmol, 1.1 equiv) was added to the reaction mixture. The reaction was stirred at −78 °C for 5 h before saturated aqueous Na_2_CO_3_ (50 mL) was added. The aqueous phase was extracted with ethyl acetate (2 × 50 mL). The combined organic phase was washed with brine (2 × 10 mL), dried over Na_2_SO_4_, filtered and concentrated under reduced pressure. The crude product was purified by column chromatography on silica gel (petroleum ether/ethyl acetate = 4:1) to obtain **9a** (3.83 g, 53% for 2 steps) as a white solid. ^1^H NMR (400 MHz, CDCl_3_) *δ* 4.42 (d, *J* = 8.9 Hz, 1H), 3.99 (dd, *J* = 7.7, 5.0 Hz, 1H), 3.71 (ddd, *J* = 11.4, 8.8, 2.2 Hz, 1H), 3.60–3.46 (m, 2H), 3.45 (s, 1H), 3.35 (s, 3H), 2.20 (dt, *J* = 11.7, 4.0 Hz, 1H), 2.12 (dd, *J* = 13.9, 7.8 Hz, 1H), 1.98 (d, *J* = 11.6 Hz, 1H), 1.89 (dd, *J* = 19.6, 6.8 Hz, 3H), 1.40 (dt, *J* = 18.0, 9.2 Hz, 3H), 1.27 (td, *J* = 11.7, 10.5, 5.1 Hz, 7H), 1.16 (s, 3H), 0.96 (s, 3H), 0.90–0.81 (m, 6H). ^13^C NMR (100 MHz, CDCl_3)_ *δ* 172.2, 81.0, 76.2, 65.5, 58.2, 53.4, 48.8, 47.9, 44.6, 38.2, 34.3, 34.0, 33.0, 32.2, 27.1, 26.6, 22.8, 20.7, 20.1, 14.2, 12.0. HRMS (ESI) *m/z*: [M + Na]^+^ calcd for C_21_H_37_NO_5_SNa^+^ 438.2285, found 438.2288.

*(2S,3S)-1-((3aR,6R)-8,8-Dimethyl-2,2-dioxidotetrahydro-3H-3a,6-methanobenzo[c]isothiazol-1(4H)-yl)-3-hydroxy-2-methoxynonan-1-one (****9b****)*. The titled compound **9b** was obtained following the general procedure described for **9a**. The residue was purified by column chromatography on silica gel (petroleum ether/ethyl acetate = 15:1) to obtain compound **9b** (3.92 g, 57% for 2 steps) as a white solid. ^1^H NMR (400 MHz, CDCl_3_) *δ* 4.35 (d, *J* = 6.4 Hz, 1H), 3.97 (t, *J* = 6.3 Hz, 1H), 3.77 (tdd, *J* = 9.2, 6.2, 2.5 Hz, 1H), 3.49 (d, *J* = 12.3 Hz, 2H), 3.37 (s, 3H), 2.18–1.99 (m, 3H), 1.97–1.82 (m, 3H), 1.66-1.60 (m, 1H), 1.58–1.47 (m, 1H), 1.45–1.33 (m, 3H), 1.32–1.19 (m, 7H), 1.12 (s, 3H), 0.95 (s, 3H), 0.89–0.77 (m, 3H). ^13^C NMR (100 MHz, CDCl_3_) *δ* 171.1, 83.7, 73.5, 65.4, 58.3, 53.3, 48.7, 47.9, 44.6, 38.2, 33.9, 33.0, 31.8, 29.2, 26.5, 25.4, 22.7, 20.6, 20.0, 14.2. HRMS (ESI) *m/z*: [M + H]^+^ calcd for C_20_H_36_NO_5_S^+^ 402.2309, found 402.2314.

*(2S,3S,4R)-1-((3aR,6R)-8,8-Dimethyl-2,2-dioxidotetrahydro-3H-3a,6-methanobenzo[c]isothiazol-1(4H)-yl)-3-hydroxy-2-methoxy-4-methyldecan-1-one (****9c****).* The titled compound **9c** was obtained following the general procedure described for **9a**. The residue was purified by column chromatography on silica gel (petroleum ether/ethyl acetate = 15:1) to obtain compound **9c** (4.13 g, 62% for 2 steps) as a white solid. ^1^H NMR (400 MHz, CDCl_3_) *δ* 4.40 (d, *J* = 8.9 Hz, 1H), 3.98 (dd, *J* = 7.7, 5.0 Hz, 1H), 3.70 (ddd, *J* = 11.3, 8.9, 2.2 Hz, 1H), 3.57–3.39 (m, 2H), 3.34 (s, 3H), 2.23–2.14 (m, 1H), 2.10 (dd, *J* = 13.9, 7.8 Hz, 1H), 1.98 (d, *J* = 11.7 Hz, 1H), 1.95–1.78 (m, 4H), 1.39 (dt, *J* = 18.2, 8.7 Hz, 3H), 1.27–1.20 (m, 9H), 1.14 (s, 3H), 0.94 (s, 3H), 0.90–0.80 (m, 6H). ^13^C NMR (100 MHz, CDCl_3_) *δ* 172.2, 80.9, 76.2, 65.5, 58.1, 53.4, 48.7, 47.9, 44.6, 38.1, 34.2, 34.0, 33.0, 31.9, 29.6, 27.3, 26.5, 22.7, 20.6, 20.0, 14.2, 12.0. HRMS (ESI) *m/z*: [M + Na]^+^ calcd for C_22_H_39_NO_5_Na^+^ 452.2442, found 452.2438.

*(2S,3S)-3-((tert-Butyldimethylsilyl)oxy)-1-((3aR,6R)-8,8-dimethyl-2,2-dioxidotetrahydro-3H-3a,6-methanobenzo[c]iso-thiazol-1(4H)-yl)-2-methoxyundecan-1-one (****9d*^*1*^***)*. The titled compound **9d** was obtained following the general procedure described for **9a**. The residue was purified by column chromatography on silica gel (petroleum ether/ethyl acetate = 15:1) to obtain crude compound **9d** as a white solid.

Since compound **9d** and raw material **12d** had similar polarity, they could not be separated by conventional methods, so the obtained **9d** was a mixture, but it did not affect the purification of the next reaction. We protected the hydroxyl group of **9d** with TBS to separate the two and collect its data.

To a solution of crude compound **9d** (426 mg, 1.13 mmol, 1.0 equiv) in CH_2_Cl_2_ (10 mL) was added 2,6-lutidine (566 mg, 3.39 mmol, 3.0 equiv) and TBSOTf (597.0 mg, 2.26 mmol, 2.0 equiv). The reaction mixture was stirred at room temperature for 3 h. The aqueous phase was extracted with ethyl acetate (2 × 10 mL). The combined organic phase was washed with brine (2 × 5 mL), dried over Na_2_SO_4_, filtered and concentrated under reduced pressure. The crude product was purified by column chromatography on silica gel (petroleum ether/ethyl acetate = 2:1) to obtain **9d^1^** (237 mg) as a white solid. Supporting Information is visible for reaction equations. ^1^H NMR (400 MHz, CDCl_3_) *δ* 4.47 (d*, J* = 3.0 Hz, 1H), 4.03 (dt*, J* = 9.5, 2.8 Hz, 1H), 3.96 (dd*, J* = 7.7, 4.4 Hz, 1H), 3.53–3.39 (m, 2H), 3.39 (s, 3H), 2.08 (dd*, J* = 13.5, 7.7 Hz, 1H), 1.97–1.82 (m, 4H), 1.62 (dtd*, J* = 13.8, 9.3, 5.1 Hz, 1H), 1.54–1.38 (m, 2H), 1.36 (d*, J* = 7.4 Hz, 1H), 1.31–1.14 (m, 12H), 1.12 (s, 3H), 0.96 (s, 3H), 0.88 (s, 12H), 0.14 (s, 3H), 0.07 (s, 3H). ^13^C NMR (100 MHz, CDCl_3_) *δ* 170.6, 85.3, 73.4, 65.2, 59.0, 53.2, 48.6, 47.9, 44.7, 38.3, 32.9, 32.0, 31.3, 29.6, 29.5, 29.3, 26.6, 26.1 (3C), 25.8, 22.8, 20.8, 20.0, 18.3, 14.2, −4.2, −4.9. HRMS (ESI) *m/z*: [M + H]^+^ calcd for C_28_H_54_NO_5_SSi^+^ 544.3487, found 544.3491.

*(2S,3S)-1-((3aR,6R)-8,8-Dimethyl-2,2-dioxidotetrahydro-3H-3a,6-methanobenzo[c]isothiazol-1(4H)-yl)-3-hydroxy-2-methylnonan-1-one (****9e****).* The titled compound **9e** was obtained following the general procedure described for **9a**. The residue was purified by column chromatography on silica gel (petroleum ether/ethyl acetate = 15:1) to obtain compound **9e** (4.22 g, 65% for 2 steps) as a white solid. ^1^H NMR (400 MHz, CDCl_3_) *δ* 3.80 (dd, *J* = 7.7, 5.0 Hz, 1H), 3.58–3.51 (m, 1H), 3.49–3.33 (m, 2H), 3.07 (p, *J* = 6.5 Hz, 1H), 2.49 (s, 1H), 2.01 (qd, *J* = 13.7, 5.6 Hz, 2H), 1.90–1.74 (m, 3H), 1.43 (ddd, *J* = 21.3, 8.6, 5.4 Hz, 2H), 1.28 (dt, *J* = 16.4, 8.4 Hz, 5H), 1.20–1.14 (m, 5H), 1.11 (d, *J* = 6.9 Hz, 3H), 1.08 (s, 3H), 0.88 (s, 3H), 0.77 (t, *J* = 6.5 Hz, 3H). ^13^C NMR (100 MHz, CDCl_3_) *δ* 175.3, 75.2, 65.2, 53.0, 48.2, 47.6, 45.2, 44.6, 38.4, 35.5, 32.7, 31.6, 29.1, 26.3, 25.3, 22.5, 20.6, 19.8, 14.0 (2C). HRMS (ESI) *m/z*: [M + H]^+^ calcd for C_20_H_36_NO_4_S^+^ 386.2360, found 386.2358.

*(2S,3S,4R)-3-((tert-Butyldimethylsilyl)oxy)-1-((3aR,6R)-8,8-dimethyl-2,2-dioxidotetrahydro-3H-3a,6-methanobenzo[c]iso-thiazol-1(4H)-yl)-2,4-dimethyldecan-1-one (****9f*^*1*^***).* The titled compound **9f^1^** was obtained following the general procedure described for **9d^1^**. The residue was purified by column chromatography on silica gel (petroleum ether/ethyl acetate = 15:1) to obtain compound **9f^1^** as a white solid. ^1^H NMR (400 MHz, CDCl_3_) *δ* 4.04 (dd, *J* = 6.2, 1.8 Hz, 1H), 3.88 (t, *J* = 6.3 Hz, 1H), 3.51–3.40 (m, 2H), 3.35 (p, *J* = 6.7 Hz, 1H), 2.04 (dd, *J* = 6.4, 1.9 Hz, 2H), 1.94–1.80 (m, 3H), 1.54–1.44 (m, 1H), 1.44–1.33 (m, 2H), 1.29–1.11 (m, 16H), 0.96 (s, 3H), 0.92–0.83 (m, 15H), 0.07 (d, *J* = 9.9 Hz, 6H). ^13^C NMR (100 MHz, CDCl_3_) *δ* 174.3, 75.5, 65.7, 53.3, 48.1, 47.8, 46.8, 44.9, 38.8, 36.6, 35.6, 33.1, 32.0, 29.7, 27.8, 26.6, 26.1 (3C), 22.8, 21.1, 20.0, 18.4, 14.7, 14.2, 12.2, −3.9, −4.8. HRMS (ESI) *m/z*: [M + H]^+^ calcd for C_28_H_54_NO_4_SSi^+^ 528.3538, found 528.3539.

*(2S,3S,4R)-3-((tert-Butyldimethylsilyl)oxy)-1-((3aR,6R)-8,8-dimethyl-2,2-dioxidotetrahydro-3H-3a,6-methanobenzo[c]isothi-azol-1(4H)-yl)-2-ethyl-4-methyldecan-1-one (****9g*^*1*^***).* The titled compound **9g^1^** was obtained following the general procedure described for **9d**. The residue was purified by column chromatography on silica gel (petroleum ether/ethyl acetate = 15:1) to obtain compound **9g^1^** as a white solid. ^1^H NMR (400 MHz, CDCl_3_) *δ* 4.09 (dd, *J* = 5.4, 1.6 Hz, 1H), 3.93 (dd, *J* = 7.7, 4.8 Hz, 1H), 3.53–3.39 (m, 2H), 3.22 (ddd, *J* = 10.8, 5.3, 2.5 Hz, 1H), 2.11–2.01 (m, 1H), 1.99–1.84 (m, 5H), 1.79 (ddd, *J* = 13.4, 7.7, 2.5 Hz, 1H), 1.46–1.34 (m, 3H), 1.29–1.11 (m, 13H), 0.96 (s, 3H), 0.93–0.80 (m, 18H), 0.13 (s, 3H), 0.07 (s, 3H). ^13^C NMR (100 MHz, CDCl_3_) *δ* 173.3, 74.3, 65.5, 56.3, 53.4, 48.1, 47.9, 44.8, 38.7, 36.6, 36.1, 33.0, 32.0, 29.7, 27.8, 26.7, 26.1 (3C), 22.8, 21.0, 20.0, 18.6, 18.4, 14.6, 14.2, 13.5, −4.0, −5.3. HRMS(ESI) *m/z*: [M + H]^+^ calcd for C_29_H_56_NO_4_SSi^+^ 542.3694, found 542.3699.

*Benzyl (2S,3S,4R)-3-hydroxy-2-methoxy-4-methylnonanoate (****8a****).* To a solution of **9a** (3.00 g, 7.22 mmol, 1.0 equiv) in THF/MeOH/H_2_O (21 mL/7 mL/7 mL) was added LiOH•H_2_O (363 mg, 8.66 mmol, 1.2 equiv) at room temperature. After being stirred for 4 h at this temperature, the reaction mixture acidified to pH = 3.0 with aqueous 10% NaHSO_4_ and extracted with ethyl acetate (2 × 300 mL). The solvent was evaporated, and the resulting mixture was directly used for the next step without further purification.

The above colorless oil was dissolved in DMF (35 mL) was added K_2_CO_3_ (2.00g, 14.44 mmol, 2.0 equiv) and BnBr (1.84 g, 10.83 mmol, 1.5 equiv) at room temperature. The mixture was stirred at room temperature for 10 h, and then H_2_O (10 mL) was added, and the resultant mixture was extracted with ethyl acetate (3 × 50 mL). The organic phase was washed by brine (4 × 100 mL). Dried over MgSO_4_, filtered and concentrated under reduced pressure. The residue was purified with column chromatography on silica gel (petroleum ether: ethyl acetate = 6:1) to afford compound **8a** (1.45 g, 65% for 2 steps) as a colorless oil. ^1^H NMR (400 MHz, CDCl_3_) *δ* 7.40–7.30 (m, 5H), 5.23 (d, *J* = 3.6 Hz, 2H), 3.86 (d, *J* = 6.4 Hz, 1H), 3.80–3.74 (m, 1H), 3.40 (s, 3H), 2.21 (d, *J* = 5.7 Hz, 1H), 1.69 (tt*, J* = 10.2, 5.9 Hz, 1H), 1.39 (ddt*, J* = 9.4, 6.0, 3.4 Hz, 1H), 1.33–1.15 (m, 7H), 0.92–0.84 (m, 6H). ^13^C NMR (100 MHz, CDCl_3_) *δ* 171.5, 135.5, 128.7 (2C), 128.5, 128.5 (2C), 82.3, 74.8, 66.9, 58.7, 34.35, 33.6, 32.1, 26.8, 22.7, 14.2, 13.6. HRMS (ESI) *m/z*: [M + Na]^+^ calcd for C_18_H_28_O_4_Na^+^ 331.1880, found 331.1878.

*Benzyl (2S,3S)-3-hydroxy-2-methoxynonanoate (****8b****).* The titled compound **8b** was obtained following the general procedure described for **8a**. The residue was purified by column chromatography on silica gel (petroleum ether/ethyl acetate = 35:1) to obtain compound **8b** (1.24 g, 62% for 2 steps) as a colorless oil. ^1^H NMR (400 MHz, CDCl_3_) *δ* 7.37–7.27 (m, 5H), 5.29–5.12 (m, 2H), 3.85 (dt, *J* = 13.5, 4.2 Hz, 2H), 3.42 (s, 3H), 2.39 (s, 1H), 1.52–1.39 (m, 3H), 1.29–1.20 (m, 7H), 0.96–0.81 (m, 3H). ^13^C NMR (100 MHz, CDCl_3_) *δ* 170.5, 135.5, 128.6 (2C), 128.5, 128.4 (2C), 84.1, 72.3, 66.7, 58.8, 32.3, 31.7, 29.2, 25.6, 22.6, 14.1. HRMS (ESI) *m/z*: [M + Na]^+^ calcd for C_17_H_26_O_4_Na^+^ 317.1724, found 317.1720.

*Benzyl (2S,3S,4R)-3-hydroxy-2-methoxy-4-methyldecanoate (****8c****).* The titled compound **8c** was obtained following the general procedure described for **8a**. The residue was purified by column chromatography on silica gel (petroleum ether/ethyl acetate = 35:1) to obtain compound **8c** (1.73 g, 63% for 2 steps) as a colorless oil. ^1^H NMR (400 MHz, CDCl_3_) *δ* 7.41–7.28 (m, 5H), 5.52–4.90 (m, 2H), 3.86 (d, *J* = 6.4 Hz, 1H), 3.81–3.73 (m, 1H), 3.40 (s, 3H), 2.20 (d, *J* = 5.8 Hz, 1H), 1.69 (dq, *J* = 12.5, 6.6, 5.4 Hz, 1H), 1.39 (qd, *J* = 9.1, 8.4, 6.1 Hz, 1H), 1.26–1.17 (m, 9H), 0.93–0.83 (m, 6H). ^13^C NMR (100 MHz, CDCl_3_) *δ* 171.6, 135.5, 128.7 (2C), 128.6, 128.5 (2C), 82.4, 74.9, 66.9, 58.7, 34.4, 33.7, 32.0, 29.6, 27.1, 22.7, 14.2, 13.6. HRMS (ESI) *m/z*: [M + Na]^+^ calcd for C_19_H_30_O_4_Na^+^ 345.2037, found 345.2037.

*Benzyl (2S,3S)-3-hydroxy-2-methoxyundecanoate (****8d****).* The titled compound **8d** was obtained following the general procedure described for **8a**. The residue was purified by column chromatography on silica gel (petroleum ether/ethyl acetate = 35:1) to obtain compound **8d** (0.983 g, 49% for 2 steps) as a colorless oil. ^1^H NMR (400 MHz, CDCl_3_) *δ* 7.34 (tdd, *J* = 10.2, 5.6, 4.1 Hz, 5H), 5.39–5.01 (m, 2H), 3.86 (dt, *J* = 14.7, 4.4 Hz, 2H), 3.43 (s, 3H), 2.41 (d, *J* = 6.1 Hz, 1H), 1.43 (ddt, *J* = 19.4, 10.0, 6.2 Hz, 3H), 1.33–1.15 (m, 11H), 0.87 (t, *J* = 6.8 Hz, 3H). ^13^C NMR (100 MHz, CDCl_3_) *δ* 170.5, 135.5, 128.7 (2C), 128.5, 128.5 (2C), 84.0, 72.3, 66.7, 58.8, 32.3, 31.9, 29.5, 29.5, 29.3, 25.7, 22.7, 14.2. HRMS (ESI) *m/z*: [M + Na]^+^ calcd for C_19_H_30_O_4_Na^+^ 345.2037, found 345.2036.

*Benzyl (2S,3S)-3-hydroxy-2-methylnonanoate (****8e****).* The titled compound **8e** was obtained following the general procedure described for **8a**. The residue was purified by column chromatography on silica gel (petroleum ether/ethyl acetate = 35:1) to obtain compound **8e** (1.27 g, 62% for 2 steps) as a colorless oil. ^1^H NMR (400 MHz, CDCl_3_) *δ* 7.43–7.27 (m, 5H), 5.15 (d, *J* = 1.7 Hz, 2H), 3.71–3.64 (m, 1H), 2.68–2.45 (m, 2H), 1.53–1.24 (m, 10H), 1.22 (d, *J* = 7.2 Hz, 3H), 0.88 (t, *J* = 6.6 Hz, 3H). ^13^C NMR (100 MHz, CDCl_3_) *δ* 176.0, 136.0, 128.7 (2C), 128.4, 128.3 (2C), 73.5, 66.5, 45.4, 34.9, 31.9, 29.4, 25.6, 22.7, 14.4, 14.2. HRMS (ESI) *m/z*: [M + Na]^+^ calcd for C_17_H_26_O_3_Na^+^ 301.1775, found 301.1776.

*Benzyl (2S,3S,4R)-3-hydroxy-2,4-dimethyldecanoate (****8f****).* The titled compound **8f** was obtained following the general procedure described for **8a**. The residue was purified by column chromatography on silica gel (petroleum ether/ethyl acetate = 35:1) to obtain compound **8f** (1.68 g, 67% for 2 steps) as a colorless oil. ^1^H NMR (400 MHz, CDCl_3_) *δ* 7.41–7.28 (m, 5H), 5.15 (s, 2H), 3.62 (dd, *J* = 7.8, 3.8 Hz, 1H), 2.70 (p, *J* = 7.3 Hz, 1H), 2.49 (s, 1H), 1.57–1.50 (m, 1H), 1.46–1.35 (m, 1H), 1.27 (h, *J* = 6.4, 5.1 Hz, 9H), 1.18 (d, *J* = 7.2 Hz, 3H), 0.91–0.85 (m, 6H). ^13^C NMR (100 MHz, CDCl_3_) *δ* 176.4, 135.9, 128.7 (2C), 128.4, 128.2 (2C), 76.1, 66.5, 43.3, 35.2, 34.0, 32.0, 29.6, 27.3, 22.8, 14.6, 14.2, 13.0. HRMS (ESI) *m/z*: [M + Na]^+^ calcd for C_19_H_30_O_3_Na^+^ 329.2088, found 329.2088.

*Benzyl (2S,3S,4R)-2-ethyl-3-hydroxy-4-methyldecanoate (****8g****).* The titled compound **8g** was obtained following the general procedure described for **8a**. The residue was purified by column chromatography on silica gel (petroleum ether/ethyl acetate = 35:1) to obtain compound **8g** (1.49 g, 71% for 2 steps) as a colorless oil. ^1^H NMR (400 MHz, CDCl_3_) *δ* 7.29–7.14 (m, 5H), 5.07 (s, 2H), 3.52 (q, *J* = 6.3 Hz, 1H), 2.54–2.39 (m, 2H), 1.58 (dtd, *J* = 15.3, 13.5, 7.3 Hz, 2H), 1.41 (ddd, *J* = 12.3, 7.3, 5.2 Hz, 1H), 1.36–1.27 (m, 1H), 1.23–1.15 (m, 9H), 0.80 (td, *J* = 7.7, 7.2, 4.5 Hz, 9H). ^13^C NMR (100 MHz, CDCl_3_) *δ* 175.9, 135.9, 128.6 (2C), 128.3 (2C), 128.3, 75.2, 66.3, 50.3, 36.1, 33.7, 31.9, 29.6, 27.1, 22.9, 22.7, 14.2, 13.6, 11.7. HRMS(ESI) *m/z*: [M + Na]^+^ calcd for C_20_H_32_O_3_Na^+^ 343.2244, found 343.2243.

*Benzyl (2S,3S,4R)-3-(((tert-butoxycarbonyl)-l-valyl)oxy)-2-methoxy-4-methylnonanoate (****6a****).* To a solution of compound **7** (2.11 g, 9.73 mmol, 3.0 equiv) and **8a** (1.00 g, 3.24 mmol, 1.0 equiv) in CH_2_Cl_2_ (3.2 mL) was added DMAP (39.6 mg, 0.32 mmol, 0.1 equiv) and DIC (1.35 g, 10.69 mmol, 3.3 equiv) under argon atmosphere at 0 °C. The reaction mixture was stirred for 1.5 h, and diluted with CH_2_Cl_2_ (50 mL). The suspension was filtered through a pad of silica gel and the filter cake was washed with CH_2_Cl_2_ (3 × 10 mL) and concentrated under reduced pressure. The residue was purified by column chromatography on silica gel (petroleum ether/ethyl acetate = 40:1 to 30:1) to obtain compound **6a** (1.33 g, 81%) as colorless oil. ^1^H NMR (400 MHz, CDCl_3_) *δ* 7.36 (d, *J* = 4.1 Hz, 5H), 5.17 (d, *J* = 3.5 Hz, 3H), 4.99 (d, *J* = 9.4 Hz, 1H), 4.19 (dd, *J* = 9.6, 4.6 Hz, 1H), 3.96 (d, *J* = 4.9 Hz, 1H), 3.34 (t, *J* = 2.4 Hz, 3H), 2.13–2.08 (m, 1H), 1.86 (d, *J* = 9.7 Hz, 1H), 1.47–1.39 (m, 10H), 1.34–1.06 (m, 7H), 0.99–0.88 (m, 6H), 0.84 (dt, *J* = 6.2, 2.4 Hz, 6H). ^13^C NMR (100 MHz, CDCl_3_) *δ* 171.9, 170.0, 155.8, 135.3, 128.7 (2C), 128.6 (3C), 80.6, 79.7, 76.7, 67.1, 58.6, 58.5, 33.6, 33.3, 32.0, 30.9, 28.4 (3C), 26.6, 22.7, 19.4, 17.1, 14.4, 14.2. HRMS (ESI) *m/z*: [M + Na]^+^ calcd for C_28_H_45_NO_7_Na^+^ 530.3089, found 530.3090.

*Benzyl (2S,3S)-3-(((tert-butoxycarbonyl)-l-valyl)oxy)-2-methoxynonanoate (****6b****).* The titled compound **6b** was obtained following the general procedure described for **6a**. Flash column chromatography eluent (petroleum ether/ethyl acetate = 9:1 to 5:1) to obtain **6b** (1.1 g, 87%) as a colorless oil. ^1^H NMR (400 MHz, CDCl_3_) *δ* 7.30 (dtd, *J* = 13.7, 7.3, 6.5, 3.3 Hz, 5H), 5.26–5.08 (m, 3H), 5.01 (d, *J* = 9.2 Hz, 1H), 4.18 (dd, *J* = 9.2, 4.6 Hz, 1H), 3.93 (d, *J* = 3.7 Hz, 1H), 3.35 (s, 3H), 2.14–2.05 (m, 1H), 1.66 (tt, *J* = 10.3, 6.3 Hz, 1H), 1.40 (s, 10H), 1.24–1.13 (m, 8H), 0.90 (d, *J* = 6.8 Hz, 3H), 0.81 (t, *J* = 6.7 Hz, 6H). ^13^C NMR (100 MHz, CDCl_3_) *δ* 171.9, 169.3, 155.6, 135.3, 128.6 (2C), 128.5, 128.5 (2C), 81.5, 79.6, 74.5, 66.8, 58.7, 58.5, 31.5, 31.0, 29.3, 28.8, 28.3 (3C), 25.2, 22.5, 19.1, 17.2, 14.0. HRMS (ESI) *m/z*: [M + Na]^+^ calcd for C_27_H_43_NO_7_Na^+^ 516.2932, found 516.2930.

*Benzyl (2S,3S,4R)-3-(((tert-butoxycarbonyl)-l-valyl)oxy)-2-methoxy-4-methyldecanoate (****6c****)*. The titled compound **6c** was obtained following the general procedure described for **6a**. Flash column chromatography eluent (petroleum ether/ethyl acetate = 9:1 to 5:1) to obtain **6c** (0.942 g, 72%) as a colorless oil. ^1^H NMR (400 MHz, CDCl_3_) *δ* 7.43–7.28 (m, 5H), 5.19 (q, *J* = 4.2, 3.7 Hz, 3H), 5.03 (d, *J* = 9.3 Hz, 1H), 4.20 (dt, *J* = 8.3, 3.4 Hz, 1H), 3.99 (t, *J* = 4.0 Hz, 1H), 3.34 (s, 3H), 2.12 (q, *J* = 6.4 Hz, 1H), 1.92–1.84 (m, 1H), 1.44 (s, 9H), 1.37–1.08 (m, 10H), 0.98–0.78 (m, 12H). ^13^C NMR (100 MHz, CDCl_3_) *δ* 171.7, 169.8, 155.6, 135.2, 128.6 (2C), 128.5 (3C), 80.5, 79.5, 76.5, 66.9, 58.5, 58.3, 33.5, 33.2, 31.7, 30.7, 29.3, 28.3 (3C), 26.8, 22.6, 19.2, 17.0, 14.3, 14.0. HRMS (ESI) *m/z*: [M + Na]^+^ calcd for C_29_H_47_NO_7_Na^+^ 544.3245, found 544.3244.

*Benzyl (2S,3S)-3-(((tert-butoxycarbonyl)-l-valyl)oxy)-2-methoxyundecanoate (****6d****).* The titled compound **6d** was obtained following the general procedure described for **6a**. Flash column chromatography eluent (petroleum ether/ethyl acetate = 9:1 to 5:1) to obtain **6d** (0.912 g, 76%) as a colorless oil. ^1^H NMR (400 MHz, CDCl_3_) *δ* 7.38–7.28 (m, 5H), 5.28–5.10 (m, 3H), 5.00 (d, *J* = 9.2 Hz, 1H), 4.21 (dd, *J* = 9.2, 4.5 Hz, 1H), 3.96 (d, *J* = 3.7 Hz, 1H), 3.38 (s, 3H), 2.17–2.08 (m, 1H), 1.68 (ddt, *J* = 13.2, 8.9, 5.8 Hz, 1H), 1.43 (s, 10H), 1.35–1.11 (m, 12H), 0.93 (d, *J* = 6.8 Hz, 3H), 0.90–0.76 (m, 6H). ^13^C NMR (100 MHz, CDCl_3_) *δ* 172.0, 169.4, 155.7, 135.4, 128.7 (2C), 128.6, 128.6 (2C), 81.6, 79.7, 74.6, 67.0, 58.8, 58.6, 31.9, 31.2, 29.4, 29.4, 29.3, 29.3, 28.4 (3C), 25.4, 22.7, 19.2, 17.2, 14.2. HRMS (ESI) *m/z*: [M + Na]^+^ calcd for C_29_H_47_NO_7_Na^+^ 544.3245, found 544.3244.

*Benzyl (2S,3S)-3-(((tert-butoxycarbonyl)-l-valyl)oxy)-2-methylnonanoate (****6e****).* The titled compound **6e** was obtained following the general procedure described for **6a**. Flash column chromatography eluent (petroleum ether/ethyl acetate = 9:1 to 5:1) to obtain **6e** (1.08 g, 82%) as a colorless oil. ^1^H NMR (400 MHz, CDCl_3_) *δ* 7.34 (d, *J* = 3.2 Hz, 5H), 5.18 (d, *J* = 5.8 Hz, 1H), 5.16–5.04 (m, 2H), 5.00 (d, *J* = 9.2 Hz, 1H), 4.18 (dt, *J* = 8.8, 3.8 Hz, 1H), 2.84 (t, *J* = 7.1 Hz, 1H), 2.06 (dt, *J* = 17.2, 5.7 Hz, 1H), 1.54 (s, 2H), 1.43 (s, 9H), 1.28–1.21 (m, 8H), 1.15 (dd, *J* = 7.6, 2.8 Hz, 3H), 0.95–0.89 (m, 3H), 0.88–0.78 (m, 6H). ^13^C NMR (100 MHz, CDCl_3_) *δ* 173.2, 171.9, 155.8, 135.1, 128.6 (2C), 128.4, 128.3 (2C), 79.7, 75.7, 66.5, 58.7, 42.9, 31.7, 31.2, 30.8, 29.1, 28.4 (3C), 24.9, 22.6, 19.3, 17.2, 14.1, 12.7. HRMS (ESI) *m/z*: [M + Na]^+^ calcd for C_27_H_43_NO_6_Na^+^ 500.2983, found 500.2982.

*Benzyl (2S,3S,4R)-3-(((tert-butoxycarbonyl)-l-valyl)oxy)-2,4-dimethyldecanoate (****6f****).* The titled compound **6f** was obtained following the general procedure described for **6a**. Flash column chromatography eluent (petroleum ether/ethyl acetate = 9:1 to 5:1) to obtain **6f** (1.35 g, 69%) as a colorless oil. ^1^H NMR (400 MHz, CDCl_3_) *δ* 7.48–7.28 (m, 5H), 5.19 (dd, *J* = 8.4, 3.9 Hz, 1H), 5.06 (s, 2H), 4.99 (d, *J* = 9.4 Hz, 1H), 4.17 (dd, *J* = 9.4, 4.4 Hz, 1H), 2.86 (dd, *J* = 8.2, 7.0 Hz, 1H), 2.05 (d, *J* = 4.4 Hz, 1H), 1.74 (dq, *J* = 11.6, 5.6, 4.9 Hz, 1H), 1.42 (s, 9H), 1.32–1.19 (m, 9H), 1.14 (d, *J* = 7.1 Hz, 3H), 1.09 (dd, *J* = 8.4, 1.5 Hz, 1H), 0.92 (d, *J* = 6.8 Hz, 3H), 0.88–0.79 (m, 9H). ^13^C NMR (100 MHz, CDCl_3_) *δ* 173.7, 171.8, 155.8, 135.8, 128.6 (2C), 128.3, 128.2 (2C), 79.6, 78.3, 66.5, 58.7, 42.1, 34.3, 33.5, 31.8, 30.8, 29.4, 28.4 (3C), 27.2, 22.7, 19.4, 17.1, 14.2, 14.1, 13.5. HRMS (ESI) *m/z*: [M + Na]^+^ calcd for C_29_H_47_NO_6_Na^+^ 528.3296, found 528.3295.

*Benzyl (2S,3S,4R)-3-(((tert-butoxycarbonyl)-l-valyl)oxy)-2-ethyl-4-methyldecanoate (****6g****).* The titled compound **6g** was obtained following the general procedure described for **6a**. Flash column chromatography eluent (petroleum ether/ethyl acetate = 9:1 to 5:1) to obtain **6g** (0.893 g, 68%) as a colorless oil. ^1^H NMR (400 MHz, CDCl_3_) *δ* 7.40–7.26 (m, 5H), 5.21 (dd, *J* = 8.0, 4.1 Hz, 1H), 5.04 (dd, *J* = 32.5, 6.9 Hz, 3H), 4.17 (dd, *J* = 9.4, 4.4 Hz, 1H), 2.72 (ddd, *J* = 9.8, 7.9, 4.8 Hz, 1H), 2.13–1.99 (m, 1H), 1.72 (td, *J* = 7.5, 4.2 Hz, 1H), 1.56 (dtdd, *J* = 23.8, 14.0, 7.5, 2.8 Hz, 2H), 1.43 (s, 9H), 1.38–1.20 (m, 9H), 1.10 (ddd, *J* = 12.5, 10.3, 5.8 Hz, 1H), 1.00–0.89 (m, 3H), 0.85 (ddd, *J* = 11.1, 5.4, 3.0 Hz, 12H). ^13^C NMR (100 MHz, CDCl_3_) *δ* 173.1, 171.8, 155.8, 135.8, 128.6 (2C), 128.4 (2C), 128.4, 79.7, 77.5, 66.5, 58.8, 49.6, 34.7, 33.6, 31.9, 30.9, 29.5, 28.4 (3C), 27.2, 22.7, 22.4, 19.5, 17.2, 14.2, 13.8, 11.6. HRMS(ESI) *m/z*: [M + Na]^+^ calcd for C_30_H_49_NO_6_Na^+^ 542.3453, found 542.3454.

*(2S,3S,4R)-2-Methoxy-4-methyl-1-oxo-1-(((R,E)-2,2,14,14-tetramethyl-9,12-dioxo-3,3-diphenyl-4,13-dioxa-10-aza-3-silapentadec-7-en-6-yl)amino)nonan-3-yl* *(tert-butoxycarbonyl)-L-valinate (****4a****).* To a solution of compound **6a** (0.8 g, 1.58 mmol, 1.0 equiv) in MeOH (16 mL) was added Pd/C (10%, 50 mg) under Ar. The suspension was degassed under vacuum and purged with H_2_ three times. The mixture was stirred under H_2_ balloon at 20 °C for 5 h. The resulting mixture was filtered through a short pad of silica gel, washed with MeOH (2 × 30 mL) and concentrated under reduced pressure. The crude product was used directly for the next step without further purification.

To a solution of above acid and **5** (1.53 g, 3.16 mmol, 2.0 equiv) in CH_2_Cl_2_ (5 mL) was added EDCI (364 mg, 1.90 mmol, 1.2 equiv) and HOBt (235 mg, 1.74 mmol, 1.1 equiv) under argon atmosphere at 20 °C. The reaction mixture was stirred for 12 h, and then quenched with 1% HCl (100 mL). The aqueous phase was extracted with ethyl acetate (2 × 80 mL). The combined organic phases were dried (Na_2_SO_4_) and concentrated under reduced pressure. The crude product was purified by column chromatography on silica gel (petroleum ether/ethyl acetate = 9:1 to 5:1) to obtain **4a** (920 mg, 66% for 2 steps) as a colorless oil. ^1^H NMR (400 MHz, CDCl_3_) *δ* 7.66–7.57 (m, 4H), 7.39 (dtt*, J* = 16.3, 6.3, 3.1 Hz, 6H), 7.20 (d*, J* = 8.2 Hz, 1H), 6.81 (dd*, J* = 15.3, 6.0 Hz, 1H), 6.11 (t*, J* = 4.9 Hz, 1H), 5.95 (dd*, J* = 15.4, 1.5 Hz, 1H), 5.20 (t*, J* = 5.2 Hz, 1H), 5.02 (d*, J* = 9.3 Hz, 1H), 4.60–4.56 (m, 1H), 4.18 (dd*, J* = 9.3, 4.6 Hz, 1H), 4.00 (dd*, J* = 5.1, 1.7 Hz, 2H), 3.84 (d*, J* = 4.8 Hz, 1H), 3.76 (qd*, J* = 10.2, 4.2 Hz, 2H), 3.46 (s, 3H), 2.13 (h*, J* = 6.6 Hz, 1H), 2.02 (q*, J* = 6.6, 6.1 Hz, 1H), 1.47 (s, 10H), 1.42 (s, 10H), 1.29–1.16 (m, 6H), 1.05 (s, 9H), 0.90 (t*, J* = 6.4 Hz, 6H), 0.86–0.75 (m, 6H). ^13^C NMR (100 MHz, CDCl_3_) *δ* 172.0, 169.1, 168.9, 165.0, 155.8, 141.3, 135.7 (2C), 135.7 (2C), 132.8, 132.6, 130.0 (2C), 128.0 (4C), 124.7, 82.5, 81.9, 79.7, 76.9, 65.0, 59.2, 58.7, 51.5, 42.2, 33.5, 32.1 (2C), 30.8, 28.4 (3C), 28.1 (3C), 26.9 (3C), 26.5, 22.7, 19.4, 19.3, 17.2, 14.8, 14.2. HRMS (ESI) *m/z*: [M + H]^+^ calcd for C_48_H_76_N_3_O_10_Si^+^ 882.5295, found 882.5295.

*(2S,3S)-2-Methoxy-1-oxo-1-(((R,E)-2,2,14,14-tetramethyl-9,12-dioxo-3,3-diphenyl-4,13-dioxa-10-aza-3-silapentadec-7-en-6-yl)amino)nonan-3-y*l *(tert-butoxycarbonyl)-l-valinate (****4b****).* The titled compound **4b** was obtained following the general procedure described for **4a**. Flash column chromatography eluent (petroleum ether/ethyl acetate = 9:1 to 5:1) to obtain **4b** (1.13 g, 81% for 2 steps) as a colorless oil. ^1^H NMR (400 MHz, CDCl_3_) *δ* 7.60 (ddt, *J* = 6.5, 4.7, 1.8 Hz, 4H), 7.44–7.31 (m, 6H), 7.27 (d, *J* = 9.1 Hz, 1H), 6.80 (dd, *J* = 15.3, 6.2 Hz, 1H), 6.28 (t, *J* = 5.1 Hz, 1H), 5.98 (dd, *J* = 15.4, 1.4 Hz, 1H), 5.35 (dt, *J* = 9.7, 3.4 Hz, 1H), 5.05 (d, *J* = 9.2 Hz, 1H), 4.62–4.58 (m, 1H), 4.20 (dd, *J* = 9.3, 4.8 Hz, 1H), 3.97 (d, *J* = 5.1 Hz, 2H), 3.85 (d, *J* = 2.6 Hz, 1H), 3.72 (qt, *J* = 6.7, 3.0 Hz, 2H), 3.48 (s, 3H), 2.11 (td, *J* = 14.5, 12.2, 6.2 Hz, 1H), 1.74 (ddt, *J* = 14.0, 9.5, 5.2 Hz, 1H), 1.43 (d, *J* = 13.3 Hz, 18H), 1.39–1.34 (m, 1H), 1.26–1.16 (m, 8H), 1.03 (d, *J* = 6.7 Hz, 9H), 0.92 (d, *J* = 6.7 Hz, 3H), 0.82 (dd, *J* = 15.2, 7.0 Hz, 6H). ^13^C NMR (100 MHz, CDCl_3_) *δ* 171.9, 169.1, 168.4, 165.0, 155.7, 141.0, 135.6 (2C), 135.6 (2C), 132.7, 132.5, 130.0 (2C), 127.9 (4C), 124.9, 83.1, 82.3, 79.7, 75.4, 65.1, 59.7, 58.7, 51.4, 42.1, 31.7, 31.1 (2C), 29.0, 28.3 (3C), 28.0 (3C), 26.8 (3C), 25.4, 22.5, 19.3, 19.2, 17.4, 14.0. HRMS (ESI) *m/z*: [M + Na]^+^ calcd for C_47_H_73_N_3_O_10_SiNa^+^ 890.4958, found 890.4954.

*(2S,3S,4R)-2-Methoxy-4-methyl-1-oxo-1-(((R,E)-2,2,14,14-tetramethyl-9,12-dioxo-3,3-diphenyl-4,13-dioxa-10-aza-3-silapentadec-7-en-6-yl)amino)decan-3-yl* *(tert-butoxycarbonyl)-l-valinate (****4c****).* The titled compound **4c** was obtained following the general procedure described for **4a**. Flash column chromatography eluent (petroleum ether/ethyl acetate = 9:1 to 5:1) to obtain **4c** (1.62 g, 86% for 2 steps) as a colorless oil. ^1^H NMR (400 MHz, CDCl_3_) *δ* 7.61 (td, *J* = 8.0, 1.7 Hz, 4H), 7.38 (ttd, *J* = 8.9, 6.4, 6.0, 2.7 Hz, 6H), 7.19 (d, *J* = 8.2 Hz, 1H), 6.81 (ddd, *J* = 15.4, 6.1, 1.6 Hz, 1H), 6.14 (t, *J* = 5.2 Hz, 1H), 5.95 (dd, *J* = 15.4, 1.8 Hz, 1H), 5.19 (t, *J* = 5.2 Hz, 1H), 5.02 (d, *J* = 9.3 Hz, 1H), 4.57 (t, *J* = 7.2 Hz, 1H), 4.17 (dd, *J* = 9.4, 4.6 Hz, 1H), 3.99 (d, *J* = 4.8 Hz, 2H), 3.84 (d, *J* = 4.8 Hz, 1H), 3.75 (qd, *J* = 10.2, 4.0 Hz, 2H), 3.45 (s, 3H), 2.13 (q, *J* = 6.3 Hz, 1H), 2.01 (h, *J* = 6.5, 5.7 Hz, 1H), 1.44 (dd, *J* = 18.6, 1.6 Hz, 18H), 1.29–1.16 (m, 10H), 1.05 (s, 9H), 0.90 (t, *J* = 6.3 Hz, 6H), 0.86–0.77 (m, 6H). ^13^C NMR (100 MHz, CDCl_3_) *δ* 172.0, 169.0, 168.8, 165.0, 155.8, 141.2, 135.7 (2C), 135.6 (2C), 132.7, 132.6, 130.0 (2C), 127.9 (4C), 124.7, 82.4, 81.9, 79.7, 76.8, 65.0, 59.1, 58.7, 51.5, 42.2, 33.5, 33.5, 31.8, 30.7, 29.5, 28.4 (3C), 28.0 (3C), 26.9 (3C), 26.8, 22.7, 19.4, 19.3, 17.2, 14.8, 14.1. HRMS (ESI) *m/z*: [M + H]^+^ calcd for C_49_H_78_N_3_O_10_Si^+^ 896.5451, found 896.5454.

*(2S,3S)-2-Methoxy-1-oxo-1-(((R,E)-2,2,14,14-tetramethyl-9,12-dioxo-3,3-diphenyl-4,13-dioxa-10-aza-3-silapentadec-7-en-6-yl)amino)undecan-3-yl* *(tert-butoxycarbonyl)-l-valinate (****4d****).* The titled compound **4d** was obtained following the general procedure described for **4a**. Flash column chromatography eluent (petroleum ether/ethyl acetate = 9:1 to 5:1) to obtain **4d** (643 mg, 63% for 2 steps) as a colorless oil. ^1^H NMR (400 MHz, CDCl_3_) *δ* 7.61 (ddd, *J* = 8.0, 4.4, 1.6 Hz, 4H), 7.46–7.33 (m, 6H), 7.27–7.22 (m, 1H), 6.81 (dd, *J* = 15.4, 6.2 Hz, 1H), 6.08 (t, *J* = 5.0 Hz, 1H), 5.95 (dd, *J* = 15.4, 1.5 Hz, 1H), 5.42–5.33 (m, 1H), 5.02 (d, *J* = 9.2 Hz, 1H), 4.62 (dqd, *J* = 8.8, 4.4, 2.1 Hz, 1H), 4.22 (dd, *J* = 9.3, 4.7 Hz, 1H), 3.99 (d, *J* = 5.0 Hz, 2H), 3.86 (d, *J* = 2.6 Hz, 1H), 3.80–3.67 (m, 2H), 3.50 (s, 3H), 2.12 (h, *J* = 6.6 Hz, 1H), 1.74 (tt, *J* = 9.3, 4.5 Hz, 1H), 1.45 (d, *J* = 15.7 Hz, 19H), 1.28–1.18 (m, 12H), 1.05 (s, 9H), 0.93 (d, *J* = 6.8 Hz, 3H), 0.91–0.81 (m, 6H). ^13^C NMR (100 MHz, CDCl_3_) *δ* 171.9, 169.1, 168.3, 164.9, 155.7, 141.2, 135.7 (2C), 135.6 (2C), 132.7, 132.6, 130.0 (2C), 128.0 (4C), 124.8, 83.2, 82.4, 79.8, 75.4, 65.2, 59.7, 58.7, 51.3, 42.2, 31.9, 31.2, 29.5, 29.5, 29.3, 28.9, 28.4 (3C), 28.1 (3C), 26.9 (3C), 25.6, 22.7, 19.3, 19.3, 17.4, 14.2. HRMS (ESI) *m/z*: [M + H]^+^ calcd for C_49_H_78_N_3_O_10_Si^+^ 896.5451, found 896.5454.

*(2S,3S)-2-Methyl-1-oxo-1-(((R,E)-2,2,14,14-tetramethyl-9,12-dioxo-3,3-diphenyl-4,13-dioxa-10-aza-3-silapentadec-7-en-6-yl)amino)nonan-3-yl* *(tert-butoxycarbonyl)-l-valinate (****4e****)*. The titled compound **4e** was obtained following the general procedure described for **4a**. Flash column chromatography eluent (petroleum ether/ethyl acetate = 9:1 to 5:1) to obtain **4e** (1.395 g, 79% for 2 steps) as a colorless oil. ^1^H NMR (400 MHz, CDCl_3_) *δ* 7.61 (dt, *J* = 8.0, 1.8 Hz, 4H), 7.45–7.32 (m, 6H), 6.82 (dd, *J* = 15.4, 5.2 Hz, 1H), 6.53 (t, *J* = 5.3 Hz, 1H), 6.12 (d, *J* = 8.4 Hz, 1H), 6.01 (dd, *J* = 15.4, 1.7 Hz, 1H), 5.17–4.95 (m, 2H), 4.70 (q, *J* = 4.8 Hz, 1H), 4.10 (dd, *J* = 8.7, 5.2 Hz, 1H), 4.04 (dd, *J* = 18.3, 5.4 Hz, 1H), 3.91 (dd, *J* = 18.3, 5.0 Hz, 1H), 3.78–3.66 (m, 2H), 2.50 (p, *J* = 7.0 Hz, 1H), 2.09 (h, *J* = 6.6 Hz, 1H), 1.63–1.55 (m, 2H), 1.43 (d, *J* = 11.1 Hz, 18H), 1.24 (q, *J* = 7.2 Hz, 8H), 1.10 (d, *J* = 7.0 Hz, 3H), 1.03 (s, 9H), 0.90 (d, *J* = 6.8 Hz, 3H), 0.88–0.79 (m, 6H). ^13^C NMR (100 MHz, CDCl_3_) *δ* 172.6, 172.0, 169.0, 165.0, 156.0, 141.6, 135.7 (2C), 135.6 (2C), 133.0, 132.8, 130.0 (2C), 127.9 (4C), 124.3, 82.2, 79.9, 76.5, 65.4, 59.0, 51.6, 44.7, 42.1, 31.7, 30.7, 30.7, 29.2, 28.4 (3C), 28.1 (3C), 26.9 (3C), 24.9, 22.6, 19.3 (2C), 17.4, 14.1, 13.4. HRMS (ESI) *m/z*: [M + H]^+^ calcd for C_47_H_74_N_3_O_9_Si^+^ 852.5189, found 852.5189.

*(2S,3S,4R)-2,4-Dimethyl-1-oxo-1-(((R,E)-2,2,14,14-tetramethyl-9,12-dioxo-3,3-diphenyl-4,13-dioxa-10-aza-3-silapentadec-7-en-6-yl)amino)decan-3-yl* *(tert-butoxycarbonyl)-l-valinate (****4f****).* The titled compound **4f** was obtained following the general procedure described for **4a**. Flash column chromatography eluent (petroleum ether/ethyl acetate = 9:1 to 5:1) to obtain **4f** (1.12 g, 85% for 2 steps) as a colorless oil. ^1^H NMR (400 MHz, CDCl_3_) *δ* 7.64–7.58 (m, 4H), 7.45–7.32 (m, 6H), 6.78 (dd, *J* = 15.5, 5.3 Hz, 1H), 6.73–6.66 (m, 1H), 6.28 (d, *J* = 8.2 Hz, 1H), 6.04 (dd, *J* = 15.4, 1.7 Hz, 1H), 5.17 (dd, *J* = 9.2, 2.6 Hz, 1H), 4.92 (d, *J* = 9.4 Hz, 1H), 4.69 (s, 1H), 4.15–3.98 (m, 2H), 3.89 (dd, *J* = 18.2, 5.0 Hz, 1H), 3.79–3.68 (m, 2H), 2.40 (dt, *J* = 14.0, 7.1 Hz, 1H), 2.19 (dq, *J* = 11.5, 7.1 Hz, 1H), 1.76–1.71 (m, 1H), 1.43 (d, *J* = 11.1 Hz, 18H), 1.28–1.19 (m, 10H), 1.08 (d, *J* = 6.9 Hz, 3H), 1.03 (s, 9H), 0.91–0.77 (m, 12H). ^13^C NMR (100 MHz, CDCl_3_) *δ* 173.2, 171.3, 168.9, 165.3, 156.4, 141.0, 135.6 (2C), 135.6 (2C), 133.1, 133.0, 129.9 (2C), 127.9 (4C), 124.6, 82.0, 79.9, 78.0, 65.3, 59.1, 51.9, 43.8, 42.0, 34.0, 33.9, 31.8, 29.7, 29.5, 28.4 (3C), 28.1 (3C), 27.2, 26.9 (3C), 22.7, 19.5, 19.3, 17.3, 14.6, 14.1, 13.2. HRMS (ESI) *m/z*: [M + H]^+^ calcd for C_49_H_78_N_3_O_9_Si^+^ 880.5502, found 880.5507.

*(3S,4S,5R)-5-Methyl-3-(((R,E)-2,2,14,14-tetramethyl-9,12-dioxo-3,3-diphenyl-4,13-dioxa-10-aza-3-silapentadec-7-en-6-yl)carbamoyl)undecan-4-yl* *(tert-butoxycarbonyl)-l-valinate (****4g****).* The titled compound **4g** was obtained following the general procedure described for **4a**. Flash column chromatography eluent (petroleum ether/ethyl acetate = 9:1 to 5:1) to obtain **4g** (529 mg, 62% for 2 steps) as a colorless oil. ^1^H NMR (400 MHz, CDCl_3_) *δ* 7.62 (dq, *J* = 8.0, 1.9 Hz, 4H), 7.38 (dtd, *J* = 14.1, 6.8, 3.3 Hz, 7H), 6.79 (dd, *J* = 15.4, 5.2 Hz, 1H), 6.68 (t, *J* = 5.4 Hz, 1H), 6.25 (d, *J* = 8.2 Hz, 1H), 6.06 (dd, *J* = 15.5, 1.7 Hz, 1H), 5.20 (dd, *J* = 8.7, 2.9 Hz, 1H), 4.93 (d, *J* = 9.5 Hz, 1H), 4.73 (ddt, *J* = 10.1, 5.2, 2.5 Hz, 1H), 4.13–3.99 (m, 2H), 3.89 (dd, *J* = 18.2, 4.9 Hz, 1H), 3.74 (d, *J* = 4.8 Hz, 2H), 2.32–2.11 (m, 2H), 1.79–1.56 (m, 2H), 1.45 (d, *J* = 9.5 Hz, 18H), 1.25 (t, *J* = 11.0 Hz, 10H), 1.04 (s, 9H), 0.86 (ddd, *J* = 25.5, 12.3, 6.6 Hz, 15H). ^13^C NMR (100 MHz, CDCl_3_) *δ* 172.3, 171.4, 169.0, 165.3, 156.4, 141.2, 135.7 (2C), 135.7 (2C), 133.0, 132.9, 130.0 (2C), 128.0 (4C), 127.9, 124.7, 82.1, 80.0, 77.7, 65.5, 59.2, 52.0, 51.9, 42.1, 34.3, 34.1, 31.9, 29.5, 28.5 (3C), 28.2 (3C), 27.2, 27.0 (3C), 22.7, 22.3, 19.6, 19.4, 17.4, 14.2, 13.5, 12.1. HRMS(ESI) *m/z*: [M + H]^+^ calcd for C_50_H_80_N_3_O_9_Si^+^ 894.5659, found 894.5662.

*(3S,11R,14S,15S,E)-11-(((tert-Butyldiphenylsilyl)oxy)methyl)-15-((R)-heptan-2-yl)-3-isopropyl-14-methoxy-1-oxa-4,7,12-triazacyclopentadec-9-ene-2,5,8,13-tetraone (****3a****)*. To a solution of **4a** (771 mg, 0.874 mmol, 1.0 equiv) in CH_2_Cl_2_ (7.0 mL) was added trifluoroacetic acid (1.7 mL) at 0 °C. After stirred for 4 h, the reaction solution was diluted with toluene (7 mL). And the resulting solution was concentrated under reduced pressure. The resulting white solid was dissolved in THF (20 mL), and the solution was added slowly to a suspension of HATU (4.98 g, 13.11 mmol, 15.0 equiv) and DIPEA (3.39 g, 26.22 mmol, 30.0 equiv) in THF (900 mL) over 30 h at room temperature and then continued to stirred another 12 h at room temperature. The solvent was evaporated, diluted with MeOH and EtOAc (*v/v* = 2:1, 300 mL) and filtered through a short pad of silica gel. The filtrate was concentrated under reduced pressure. The residue was dissolved in EtOAc (300 mL), washed with 1% HCl (80 mL), saturated aqueous solution of NaHCO_3_ (60 mL), and brine (60 mL). The organic phase was dried (Na_2_SO_4_) and concentrated under reduced pressure. The crude product was purified by column chromatography on silica gel (dichloromethane/methanol = 50:1 to 9:1) to obtain **3a** (460 mg, 67% for 2 steps) as a white solid. ^1^H NMR (400 MHz, DMSO-*d*_6_) *δ* 8.41 (d, *J* = 9.0 Hz, 1H), 8.22 (d, *J* = 9.8 Hz, 1H), 7.59 (ddd, *J* = 7.8, 3.5, 1.6 Hz, 4H), 7.48–7.35 (m, 6H), 6.71 (dd, *J* = 15.1, 3.0 Hz, 1H), 5.92 (dd, *J* = 15.1, 2.2 Hz, 1H), 5.31–5.20 (m, 1H), 4.70 (dtt, *J* = 8.9, 6.0, 2.4 Hz, 1H), 4.25 (dd, *J* = 9.8, 6.9 Hz, 1H), 4.06–3.95 (m, 1H), 3.88 (d, *J* = 9.7 Hz, 1H), 3.58 (dd, *J* = 19.4, 8.3 Hz, 3H), 3.33 (d, *J* = 1.2 Hz, 1H), 3.20 (s, 3H), 2.02–1.90 (m, 1H), 1.84 (q, *J* = 7.1, 6.6 Hz, 1H), 1.28–1.13 (m, 8H), 0.95 (d, *J* = 10.1 Hz, 12H), 0.88–0.76 (m, 9H). ^13^C NMR (100 MHz, DMSO-*d*_6_) *δ* 169.1, 168.8, 167.7, 166.5, 141.5, 135.2 (2C), 135.1 (2C), 132.7, 132.6, 130.0, 130.0, 128.0 (4C), 119.5, 78.4, 73.4, 65.5, 57.4, 56.7, 51.5, 44.1, 33.1, 32.9, 31.5, 31.4, 26.6 (3C), 26.6, 22.1, 19.3 (2C), 18.8, 18.3, 14.0. HRMS (ESI) *m/z*: [M + H]^+^ calcd for C_39_H_58_N_3_O_7_Si^+^ 708.4039, found 708.4039.

*(3S,11R,14S,15S,E)-11-(((tert-Butyldiphenylsilyl)oxy)methyl)-15-hexyl-3-isopropyl-14-methoxy-1-oxa-4,7,12-triazacyclopen-tadec-9-ene-2,5,8,13-tetraone (****3b****).* The titled compound **3b** was obtained following the general procedure described for **3a**. Flash column chromatography eluent (dichloromethane/methanol = 50:1 to 9:1) to obtain **3b** (870 mg, 82% for 2 steps) as a white solid. ^1^H NMR (400 MHz, DMSO-*d*_6_) *δ* 8.46 (d, *J* = 8.9 Hz, 1H), 8.26 (d, *J* = 9.4 Hz, 1H), 7.66–7.60 (m, 4H), 7.57 (t, *J* = 6.8 Hz, 1H), 7.45 (qd, *J* = 6.7, 2.5 Hz, 6H), 6.78 (dd, *J* = 15.1, 2.9 Hz, 1H), 5.92 (dd, *J* = 15.1, 2.3 Hz, 1H), 5.16 (dt, *J* = 9.3, 4.4 Hz, 1H), 4.76 (ddt, *J* = 9.6, 6.8, 3.9 Hz, 1H), 4.33 (dd, *J* = 9.4, 5.7 Hz, 1H), 4.03 (dd, *J* = 17.7, 5.9 Hz, 1H), 3.81 (d, *J* = 9.7 Hz, 1H), 3.69–3.56 (m, 3H), 3.26 (s, 3H), 2.09 (dt, *J* = 13.1, 6.6 Hz, 1H), 1.64 (ddt, *J* = 15.2, 10.4, 4.9 Hz, 2H), 1.45–1.31 (m, 2H), 1.25 (d, *J* = 4.4 Hz, 6H), 1.00 (s, 9H), 0.87 (t, *J* = 7.1 Hz, 9H). ^13^C NMR (100 MHz, DMSO-*d*_6_) *δ* 169.6, 168.9, 167.7, 166.4, 141.6, 135.2 (2C), 135.1 (2C), 132.6, 132.6, 130.0, 130.0, 127.9 (4C), 119.4, 79.4, 71.7, 65.3, 56.9, 56.8, 51.5, 43.9, 31.2 (2C), 30.1, 28.8, 26.6 (3C), 23.1, 22.0, 19.0, 18.8, 17.7, 13.9. HRMS (ESI) *m/z*: [M + H]^+^ calcd for C_38_H_56_N_3_O_7_Si^+^ 694.3883, found 694.3882.

*(3S,11R,14S,15S,E)-11-(((tert-Butyldiphenylsilyl)oxy)methyl)-3-isopropyl-14-methoxy-15-((R)-octan-2*-*yl)-1-oxa-4,7,12-triazacyclopentadec-9-ene-2,5,8,13-tetraone (****3c****)*. The titled compound **3c** was obtained following the general procedure described for **3a**. Flash column chromatography eluent (dichloromethane/methanol = 50:1 to 9:1) to obtain **3c** (501 mg, 67% for 2 steps) as a white solid. ^1^H NMR (400 MHz, DMSO-*d*_6_) *δ* 8.41 (d, *J* = 9.0 Hz, 1H), 8.21 (d, *J* = 9.8 Hz, 1H), 7.69–7.54 (m, 5H), 7.44 (qdd, *J* = 8.4, 3.3, 1.8 Hz, 6H), 6.74 (dd, *J* = 15.1, 3.0 Hz, 1H), 5.95 (dd, *J* = 15.2, 2.2 Hz, 1H), 5.26 (dd, *J* = 9.8, 1.7 Hz, 1H), 4.73 (ddt, *J* = 9.5, 6.7, 3.8 Hz, 1H), 4.28 (dd, *J* = 9.7, 6.9 Hz, 1H), 4.03 (dd, *J* = 17.5, 5.8 Hz, 1H), 3.90 (d, *J* = 9.7 Hz, 1H), 3.67–3.53 (m, 3H), 3.23 (s, 3H), 2.03–1.90 (m, *J* = 7.3 Hz, 1H), 1.87 (q, *J* = 6.8 Hz, 1H), 1.32–1.20 (m, 9H), 1.17–1.02 (m, 1H), 0.98 (d, *J* = 11.4 Hz, 12H), 0.91–0.81 (m, 9H). ^13^C NMR (100 MHz, DMSO-*d*_6_) *δ* 169.0, 168.7, 167.6, 166.5, 141.4, 135.1 (2C), 135.0 (2C), 132.6, 132.6, 130.0, 130.0, 127.9 (4C), 119.5, 78.4, 73.4, 65.4, 57.4, 56.6, 51.4, 44.0, 33.1, 32.9, 31.3, 31.2, 28.9, 26.8, 26.6 (3C), 22.0, 19.2, 18.8, 18.2, 13.9, 13.9. HRMS (ESI) *m/z*: [M + H]^+^ calcd for C_40_H_60_N_3_O_7_Si^+^ 722.4196, found 722.4195.

*(3S,11R,14S,15S,E)-11-(((tert-Butyldiphenylsilyl)oxy)methyl)-3-isopropyl-14-methoxy-15-octyl-1-oxa-4,7,12-triazacyclopentadec-9-ene-2,5,8,13-tetraone (****3d****).* The titled compound **3d** was obtained following the general procedure described for **3a**. Flash column chromatography eluent (dichloromethane/methanol = 50:1 to 9:1) to obtain **3d** (216 mg, 62% for 2 steps) as a white solid. ^1^H NMR (400 MHz, DMSO-*d*_6_) *δ* 8.55 (d, *J* = 8.9 Hz, 1H), 8.32 (d, *J* = 9.4 Hz, 1H), 7.59 (s, 4H), 7.56 (t, *J* = 6.8 Hz, 1H), 7.44 (dtdd, *J* = 11.6, 8.6, 6.0, 1.9 Hz, 6H), 6.76 (dd, *J* = 15.1, 3.0 Hz, 1H), 5.98 (dd, *J* = 15.2, 2.3 Hz, 1H), 5.14 (dt, *J* = 9.2, 4.5 Hz, 1H), 4.76 (qd, *J* = 6.6, 3.7 Hz, 1H), 4.32 (dd, *J* = 9.4, 5.8 Hz, 1H), 4.10 (dd, *J* = 17.6, 6.0 Hz, 1H), 3.87 (d, *J* = 9.7 Hz, 1H), 3.71–3.54 (m, 3H), 3.38 (d, *J* = 7.4 Hz, 3H), 2.12–1.99 (m, *J* = 7.2 Hz, 1H), 1.62 (ddt, *J* = 20.3, 14.6, 6.9 Hz, 2H), 1.24 (s, 12H), 0.99 (s, 9H), 0.90–0.77 (m, 9H). ^13^C NMR (100 MHz, DMSO-*d*_6_) *δ* 169.5, 169.0, 167.8, 166.4, 141.4, 135.1 (2C), 135.0 (2C), 132.6, 132.6, 129.9, 129.9, 127.9 (4C), 119.5, 79.3, 71.8, 65.4, 57.0, 56.8, 51.6, 43.9, 31.2, 31.2, 30.1, 29.0, 28.8, 28.5, 26.6 (3C), 23.1, 22.1, 19.0, 18.8, 17.7, 13.9. HRMS (ESI) *m/z*: [M + H]^+^ calcd for C_40_H_60_N_3_O_7_Si^+^ 722.4196, found 722.4200.

*(3S,11R,14S,15S,E)-11-(((tert-Butyldiphenylsilyl)oxy)methyl)-15-hexyl-3-isopropyl-14-methyl-1-oxa-4,7,12-triazacyclopentadec-9-ene-2,5,8,13-tetraone (****3e****).* The titled compound **3e** was obtained following the general procedure described for **3a**. Flash column chromatography eluent (dichloromethane/methanol = 50:1 to 9:1) to obtain **3e** (362 mg, 66% for 2 steps) as a white solid. ^1^H NMR (400 MHz, DMSO-*d*_6_) *δ* 8.23 (d, *J* = 9.5 Hz, 1H), 7.95 (d, *J* = 8.7 Hz, 1H), 7.62 (td, *J* = 6.1, 3.0 Hz, 4H), 7.56–7.36 (m, 7H), 6.75 (dd, *J* = 15.0, 2.8 Hz, 1H), 5.91 (dd, *J* = 15.0, 2.3 Hz, 1H), 5.13 (dt, *J* = 10.8, 4.2 Hz, 1H), 4.64 (dtt, *J* = 8.2, 5.4, 2.7 Hz, 1H), 4.31 (dd, *J* = 9.5, 5.5 Hz, 1H), 4.04 (dd, *J* = 17.7, 6.1 Hz, 1H), 3.66 (dd, *J* = 10.1, 5.8 Hz, 1H), 3.63–3.49 (m, 2H), 2.72 (dq, *J* = 10.3, 6.9 Hz, 1H), 2.06 (dq, *J* = 13.3, 6.7 Hz, 1H), 1.54 (ddt, *J* = 24.6, 15.0, 4.1 Hz, 2H), 1.38 (p, *J* = 5.1, 4.5 Hz, 2H), 1.30–1.21 (m, 9H), 1.00 (d, *J* = 10.9 Hz, 12H), 0.86 (dt, *J* = 7.3, 3.7 Hz, 6H). ^13^C NMR (100 MHz, DMSO-*d*_6_) *δ* 173.0, 170.0, 169.0, 166.3, 142.4, 135.1 (2C), 135.1 (2C), 132.8, 132.7, 129.9 (2C), 127.9 (4C), 119.1, 74.8, 65.1, 56.8, 51.4, 43.8, 42.0, 31.2, 31.2, 30.1, 28.8, 26.6 (3C), 22.6, 22.0, 19.0, 18.8, 17.6, 15.5, 13.9. HRMS (ESI) *m/z*: [M + Na]^+^ calcd for C_38_H_55_N_3_O_6_SiNa^+^ 700.3753, found 700.3747.

*(3S,11R,14S,15S,E)-11-(((tert-Butyldiphenylsilyl)oxy)methyl)-3-isopropyl-14-methyl-15-((R)-octan-2*-*yl)-1-oxa-4,7,12-triazacyclopentadec-9-ene-2,5,8,13-tetraone (****3f****)*. The titled compound **3f** was obtained following the general procedure described for **3a**. Flash column chromatography eluent (dichloromethane/methanol = 50:1 to 9:1) to obtain **3f** (332 mg, 69% for 2 steps) as a white solid. ^1^H NMR (400 MHz, DMSO-*d*_6_) *δ* 8.21 (d, *J* = 9.8 Hz, 1H), 7.92 (d, *J* = 8.8 Hz, 1H), 7.62 (ddd, *J* = 8.1, 4.9, 1.8 Hz, 4H), 7.54 (t, *J* = 6.8 Hz, 1H), 7.44 (dtt, *J* = 10.5, 6.6, 2.2 Hz, 6H), 6.72 (dd, *J* = 15.0, 2.7 Hz, 1H), 5.94 (dd, *J* = 15.1, 2.3 Hz, 1H), 5.26 (dd, *J* = 10.4, 1.6 Hz, 1H), 4.67–4.61 (m, 1H), 4.25 (dd, *J* = 9.8, 6.9 Hz, 1H), 4.03 (dd, *J* = 17.4, 5.9 Hz, 1H), 3.68–3.49 (m, 3H), 2.79 (dt, *J* = 14.6, 7.2 Hz, 1H), 1.93 (h, *J* = 6.9 Hz, 1H), 1.71 (q, *J* = 7.3, 6.8 Hz, 1H), 1.24 (d, *J* = 10.0 Hz, 10H), 1.02 (d, *J* = 7.0 Hz, 3H), 0.98 (d, *J* = 2.1 Hz, 9H), 0.93 (d, *J* = 6.8 Hz, 3H), 0.90–0.81 (m, 9H). ^13^C NMR (100 MHz, DMSO-*d*_6_) *δ* 173.0, 169.1, 168.6, 166.5, 142.2, 135.1 (2C), 135.1 (2C), 132.8, 132.7, 129.9 (2C), 127.9 (4C), 119.3, 76.4, 65.2, 57.5, 51.2, 44.0, 41.7, 33.5, 33.4, 31.4, 31.2, 28.9, 26.8, 26.6 (3C), 22.1, 19.3, 18.8, 18.3, 16.0, 13.9, 13.3. HRMS (ESI) *m/z*: [M + H]^+^ calcd for C_40_H_60_N_3_O_6_Si^+^ 706.4246, found 706.4251.

*(3S,11R,14S,15S,E)-11-(((tert-Butyldiphenylsilyl)oxy)methyl)-14-ethyl-3-isopropyl-15-((R)-octan-2-yl)-1-oxa-4,7,12-triazacy-clopentadec-9-ene-2,5,8,13-tetraone (****3g****).* The titled compound **3g** was obtained following the general procedure described for **3a**. Flash column chromatography eluent (dichloromethane/methanol = 50:1 to 9:1) to obtain **3g** (289 mg, 58% for 2 steps) as a white solid. ^1^H NMR (400 MHz, DMSO-*d*_6_) *δ* 8.18 (d, *J* = 9.9 Hz, 1H), 7.90 (d, *J* = 9.0 Hz, 1H), 7.64–7.52 (m, 4H), 7.47–7.38 (m, 6H), 6.76 (dd, *J* = 15.1, 2.8 Hz, 1H), 5.98 (dd, *J* = 15.1, 2.3 Hz, 1H), 5.34–5.27 (m, 1H), 4.69 (ddt, *J* = 9.6, 6.8, 3.9 Hz, 1H), 4.30–4.21 (m, 1H), 4.09–3.98 (m, 1H), 3.61–3.50 (m, 3H), 2.69 (dt, *J* = 10.0, 4.7 Hz, 1H), 1.99–1.90 (m, 1H), 1.70 (q, *J* = 6.9 Hz, 1H), 1.51 (ddd, *J* = 11.9, 7.2, 4.1 Hz, 1H), 1.37 (td, *J* = 8.7, 3.9 Hz, 2H), 1.24 (d, *J* = 9.0 Hz, 10H), 0.99 (s, 9H), 0.93 (d, *J* = 6.8 Hz, 3H), 0.90–0.81 (m, 9H), 0.76 (t, *J* = 7.4 Hz, 3H). ^13^C NMR (100 MHz, DMSO-*d*_6_) *δ* 171.4, 169.1, 168.6, 166.6, 142.2, 135.1 (2C), 135.1 (2C), 132.6, 132.6, 130.0 (2C), 127.9 (4C), 119.4, 75.4, 65.5, 57.4, 51.1, 47.8, 44.1, 33.6, 33.4, 31.5, 31.1, 28.9, 26.8, 26.6 (3C), 22.4, 22.0, 19.4, 18.8, 18.3, 13.9, 13.4, 10.7. HRMS(ESI) *m/z*: [M + H]^+^ calcd for C_41_H_62_N_3_O_6_Si^+^ 720.4403, found 720.4404.

*(3S,14S,15S,E)-15-((R)-Heptan-2-yl)-3-isopropyl-14-methoxy-11-methylene-1-oxa-4,7,12-triazacyclopentadec-9-ene-2,5,8,13-tetraone (****1a****).* To a solution of compound **3a** (460 mg, 0.650 mmol, 1.0 equiv) in THF (10 mL) were added HOAc (97.5 mg, 1.63 mmol, 2.5 equiv) and TBAF (1.63 ml, 1 mol/L in THF, 1.63 mmol, 3.0 equiv). The mixture was stirred at room temperature for 24 h, and then concentrated. The residue was purified by column chromatography on silica gel (dichloromethane/methanol = 20:1 to 9:1) to obtain **2a** as a white powder.

To a solution of compound **2a** (292 mg, 0.622 mmol, 1.0 equiv) in THF (6.2 mL), then triethylamine (157 mg, 1.56 mmol, 3.0 equiv) and ethanesulfonyl chloride (160 mg, 1.24 mmol, 2.0 equiv) were added at 0 °C. After stirred for 2 h, the reaction solution was quenched by addition of H_2_O (0.1 mL), dried over Na_2_SO_4_, filtered and concentrated under reduced pressure. The resulting crude product was dissolved in THF (6.2 mL). To the resulting solution was added DBU (806 mg, 5.3 mmol, 8 equiv) at 20 °C. After stirred for 2.5 h, the solution was dissolved in ethyl acetate (80 mL) and washed successively with 1% HCl (30 mL), saturated aqueous NaHCO_3_ (30 mL), brine (30 mL), dried over Na_2_SO_4_ and concentrated under reduced pressure carefully. The crude product was purified by column chromatography on silica gel (dichloromethane/methanol = 100:1 to 100:6), *t*-BuOH (3 mL) was added to the eluent and concentrated to remove CH_2_Cl_2_ and MeOH. The residual solution was lyophilized to give **1a** (155 mg, 52% for 2 steps) as a white solid. ^1^H NMR (400 MHz, DMSO-*d*_6_) *δ* 9.17 (s, 1H), 8.24 (d*, J* = 9.8 Hz, 1H), 7.68 (dd*, J* = 7.7, 5.3 Hz, 1H), 6.89 (d*, J* = 15.2 Hz, 1H), 6.11 (d*, J* = 15.2 Hz, 1H), 5.59 (s, 1H), 5.46 (s, 1H), 5.18 (dd*, J* = 8.7, 3.0 Hz, 1H), 4.31 (dd*, J* = 9.8, 7.0 Hz, 1H), 4.13–3.90 (m, 2H), 3.59 (dd*, J* = 17.2, 7.7 Hz, 1H), 3.30 (s, 3H), 1.98 (tt*, J* = 15.4, 7.3 Hz, 2H), 1.25 (tdd*, J* = 16.1, 7.7, 4.1 Hz, 8H), 0.95 (d*, J* = 6.8 Hz, 3H), 0.89–0.82 (m, 9H). ^13^C NMR (100 MHz, DMSO-*d*_6_) *δ* 169.2, 168.9, 168.0, 167.0, 138.1, 137.4, 119.0, 116.3, 79.0, 74.2, 57.4, 56.8, 44.3, 33.1, 32.7, 31.4, 31.3, 26.3, 22.0, 19.2, 18.2, 14.0, 13.9. HRMS (ESI) *m/z*: [M + H]^+^ calcd for C_23_H_38_N_3_O_6_^+^ 452.2756, found 452.2758.

*(3S,14S,15S,E)-15-Hexyl-3-isopropyl-14-methoxy-11-methylene-1-oxa-4,7,12-triazacyclopentadec-9-ene-2,5,8,13-tetraone (****1b****).* The titled compound **1b** was obtained following the general procedure described for **1a**. The crude product was purified by column chromatography on silica gel (dichloromethane/methanol = 100:1 to 100:6), *t-*BuOH (3 mL) was added to the eluent and concentrated to remove CH_2_Cl_2_ and MeOH. The residual solution was lyophilized to give **1b** (123 mg, 57% for 2 steps) as a white solid. ^1^H NMR (400 MHz, DMSO-*d*_6_) *δ* 9.13 (s, 1H), 8.33 (d, *J* = 9.5 Hz, 1H), 7.65 (t, *J* = 6.5 Hz, 1H), 6.94 (d, *J* = 15.2 Hz, 1H), 6.03 (d, *J* = 15.2 Hz, 1H), 5.51 (d, *J* = 6.3 Hz, 2H), 5.16 (q, *J* = 5.9 Hz, 1H), 4.40 (dd, *J* = 9.5, 5.6 Hz, 1H), 4.00 (dd, *J* = 17.4, 5.4 Hz, 1H), 3.91 (d, *J* = 7.7 Hz, 1H), 3.58 (dd, *J* = 17.6, 7.8 Hz, 1H), 3.32 (s, 3H), 2.10 (q, *J* = 6.5 Hz, 1H), 1.96 (ddd, *J* = 28.6, 13.9, 7.4 Hz, 1H), 1.79 (q, *J* = 12.3 Hz, 1H), 1.64 (dt, *J* = 14.7, 7.0 Hz, 1H), 1.46 (d, *J* = 9.1 Hz, 1H), 1.39–1.26 (m, 6H), 0.85 (q, *J* = 6.5 Hz, 9H). ^13^C NMR (100 MHz, DMSO-*d*_6_) *δ* 169.8, 169.1, 167.8, 167.0, 138.3, 137.6, 119.3, 116.9, 80.4, 72.3, 57.2, 56.8, 44.0, 31.1, 31.1, 30.2, 28.6, 23.7, 22.0, 19.0, 17.6, 13.1. HRMS (ESI) *m/z*: [M + Na]^+^ calcd for C_22_H_35_N_3_O_6_Na^+^ 460.2419, found 460.2423.

*(3S,14S,15S,E)-3-Isopropyl-14-methoxy-11-methylene-15-((R)-octan-2-yl)-1-oxa-4,7,12-triazacyclopentadec-9-ene-2,5,8,13-tetraone (****1c****).* The titled compound **1c** was obtained following the general procedure described for **1a**. The crude product was purified by column chromatography on silica gel (dichloromethane/methanol = 100:1 to 100:6), *t*-BuOH (3 mL) was added to the eluent and concentrated to remove CH_2_Cl_2_ and MeOH. The residual solution was lyophilized to give **1c** (109 mg, 47% for 2 steps) as a white solid. ^1^H NMR (400 MHz, DMSO-*d*_6_) *δ* 9.23 (s, 1H), 8.28 (d, *J* = 9.8 Hz, 1H), 7.68 (dd, *J* = 7.7, 5.3 Hz, 1H), 6.87 (d, *J* = 15.2 Hz, 1H), 6.19 (d, *J* = 15.3 Hz, 1H), 5.61 (s, 1H), 5.45 (s, 1H), 5.16 (dd, *J* = 8.7, 2.9 Hz, 1H), 4.30 (dd, *J* = 9.7, 7.1 Hz, 1H), 4.19–4.05 (m, 2H), 3.62–3.54 (m, 1H), 3.34 (s, 3H), 1.98 (dq, *J* = 13.6, 7.0 Hz, 2H), 1.31–1.21 (m, 9H), 1.16–1.05 (m, 1H), 0.95 (dd, *J* = 7.2, 2.9 Hz, 3H), 0.88–0.81 (m, 9H). ^13^C NMR (100 MHz, DMSO-*d*_6_) *δ* 169.1, 169.0, 168.1, 167.0, 138.1, 137.4, 119.1, 116.1, 78.9, 74.3, 57.5, 56.8, 44.2, 33.1, 32.7, 31.4, 31.2, 28.9, 26.6, 22.1, 19.2, 18.2, 14.0, 13.9. HRMS (ESI) *m/z*: [M + H]^+^ calcd for C_24_H_40_N_3_O_6_^+^ 466.2912, found 466.2911.

*(3S,14S,15S,E)-3-Isopropyl-14-methoxy-11-methylene-15-octyl-1-oxa-4,7,12-triazacyclopentadec-9-ene-2,5,8,13-tetraone (****1d****).* The titled compound **1d** was obtained following the general procedure described for **1a**. The crude product was purified by column chromatography on silica gel (dichloromethane/methanol = 100:1 to 100:6), *t-*BuOH (3 mL) was added to the eluent and concentrated to remove CH_2_Cl_2_ and MeOH. The residual solution was lyophilized to give **1d** (87 mg, 67% for 2 steps) as a white solid. ^1^H NMR (400 MHz, DMSO-*d*_6_) *δ* 9.23 (s, 1H), 8.38 (d, *J* = 9.5 Hz, 1H), 7.66 (dd, *J* = 7.6, 5.4 Hz, 1H), 6.92 (d, *J* = 15.2 Hz, 1H), 6.12 (d, *J* = 15.2 Hz, 1H), 5.54 (s, 1H), 5.49 (s, 1H), 5.14 (dt, *J* = 7.7, 5.1 Hz, 1H), 4.38 (dd, *J* = 9.5, 5.7 Hz, 1H), 4.09 (dd, *J* = 17.3, 5.4 Hz, 1H), 3.99 (d, *J* = 8.0 Hz, 1H), 3.57 (dd, *J* = 17.4, 7.7 Hz, 1H), 3.31 (s, 3H), 2.08 (p, *J* = 6.7 Hz, 1H), 1.65 (p, *J* = 6.4 Hz, 2H), 1.56 (dt, *J* = 15.3, 7.9 Hz, 2H), 1.35–1.25 (m, 10H), 0.93 (t, *J* = 7.3 Hz, 3H), 0.85 (q, *J* = 6.1 Hz, 6H). ^13^C NMR (100 MHz, DMSO-*d*_6_) *δ* 169.7, 169.2, 168.0, 167.0, 138.3, 137.6, 119.3, 116.7, 80.3, 72.4, 57.1, 56.9, 44.0, 31.2 (2C), 30.2, 28.9, 28.9, 28.6, 23.6, 22.1, 19.0, 17.6, 14.0. HRMS (ESI) *m/z*: [M + H]^+^ calcd for C_24_H_40_N_3_O_6_^+^ 466.2912, found 466.2912.

*(3S,14S,15S,E)-15-Hexyl-3-isopropyl-14-methyl-11-methylene-1-oxa-4,7,12-triazacyclopentadec-9-ene-2,5,8,13-tetraone (****1e****)*. The titled compound **1e** was obtained following the general procedure described for **1a**. The crude product was purified by column chromatography on silica gel (dichloromethane/methanol = 100:1 to 100:6), *t*-BuOH (3 mL) was added to the eluent and concentrated to remove CH_2_Cl_2_ and MeOH. The residual solution was lyophilized to give **1e** (93 mg, 61% for 2 steps) as a white solid. ^1^H NMR (400 MHz, DMSO-*d*_6_) *δ* 9.18 (s, 1H), 8.38 (d, *J* = 9.4 Hz, 1H), 7.64 (t, *J* = 6.7 Hz, 1H), 6.95 (d, *J* = 15.0 Hz, 1H), 6.16 (d, *J* = 15.0 Hz, 1H), 5.45 (s, 1H), 5.33 (s, 1H), 5.04 (dt, *J* = 10.6, 4.1 Hz, 1H), 4.26 (dd, *J* = 9.4, 6.1 Hz, 1H), 4.19 (dd, *J* = 17.2, 5.7 Hz, 1H), 3.52 (dd, *J* = 17.3, 7.6 Hz, 1H), 2.91 (dt, *J* = 10.3, 7.0 Hz, 1H), 2.00 (dt, *J* = 14.8, 7.1 Hz, 2H), 1.59–1.47 (m, 2H), 1.27–1.21 (m, 7H), 1.05 (d, *J* = 6.9 Hz, 3H), 0.94–0.80 (m, 9H). ^13^C NMR (100 MHz, DMSO-*d*_6_) *δ* 172.6, 169.7, 169.0, 166.8, 138.8, 138.6, 118.8, 117.5, 75.1, 57.2, 43.9, 41.9, 31.3, 31.1, 30.1, 28.8, 22.5, 23.0, 19.0, 17.8, 15.0, 13.9. HRMS (ESI) *m/z*: [M + H]^+^ calcd for C_22_H_36_N_3_O_5_^+^ 422.2650, found 422.2652.

*(3S,14S,15S,E)-3-Isopropyl-14-methyl-11-methylene-15-((R)-octan-*2-yl*)-1-oxa-4,7,12-triazacyclopentadec-9-ene-2,5,8,13-tetraone (****1f****).* The titled compound **1f** was obtained following the general procedure described for **1a**. The crude product was purified by column chromatography on silica gel (dichloromethane/methanol = 100:1 to 100:6), *t-*BuOH (3 mL) was added to the eluent and concentrated to remove CH_2_Cl_2_ and MeOH. The residual solution was lyophilized to give **1f** (118 mg, 59% for 2 steps) as a white solid. ^1^H NMR (400 MHz, DMSO-*d*_6_) *δ* 8.94 (s, 1H), 8.24 (d, *J* = 9.8 Hz, 1H), 7.66 (t, *J* = 6.6 Hz, 1H), 6.91 (d, *J* = 15.1 Hz, 1H), 6.13 (d, *J* = 15.1 Hz, 1H), 5.44 (s, 1H), 5.37 (s, 1H), 5.18 (d, *J* = 10.2 Hz, 1H), 4.27–4.18 (m, 1H), 4.08 (dd, *J* = 17.2, 5.6 Hz, 1H), 3.55 (dd, *J* = 17.2, 7.8 Hz, 1H), 2.89 (dt, *J* = 13.8, 7.2 Hz, 1H), 1.92 (h, *J* = 6.5 Hz, 1H), 1.73 (q, *J* = 6.9 Hz, 1H), 1.62–1.50 (m, 1H), 1.39–1.25 (m, 9H), 1.06 (d, *J* = 7.0 Hz, 3H), 0.94 (d, *J* = 6.6 Hz, 3H), 0.86 (dd, *J* = 10.9, 6.7 Hz, 9H). ^13^C NMR (100 MHz, DMSO-*d*_6_) *δ* 172.5, 169.0, 168.7, 166.9, 138.8, 138.2, 118.6, 117.1, 76.7, 57.7, 44.2, 41.8, 33.5, 31.4, 31.2, 28.9, 26.8, 23.1, 22.0, 19.3, 18.4, 15.4, 13.9, 13.2. HRMS (ESI) *m/z*: [M + H]^+^ calcd for C_24_H_40_N_3_O_5_^+^450.2963, found 450.2966.

*(3S,14S,15S,E)-14-Ethyl-3-isopropyl-11-methylene-15-((R)-octan-2-yl)-1-oxa-4,7,12-triazacyclopentadec-9-ene-2,5,8,13-tetraone (****1g****).* The titled compound **1g** was obtained following the general procedure described for **1a**. The crude product was purified by column chromatography on silica gel (dichloromethane/methanol = 100:1 to 100:6), *t-*BuOH (3 mL) was added to the eluent and concentrated to remove CH_2_Cl_2_ and MeOH. The residual solution was lyophilized to give **1g** (93 mg, 56% for 2 steps) as a white solid. ^1^H NMR (400 MHz, DMSO-*d*_6_) *δ* 8.92 (s, 1H), 8.18 (d, *J* = 9.9 Hz, 1H), 7.66 (dd, *J* = 7.7, 5.4 Hz, 1H), 6.87 (d, *J* = 15.1 Hz, 1H), 6.16 (d, *J* = 15.1 Hz, 1H), 5.42 (s, 2H), 5.23 (dd, *J* = 10.1, 1.7 Hz, 1H), 4.25 (dd, *J* = 9.9, 7.3 Hz, 1H), 4.07 (dd, *J* = 17.2, 5.4 Hz, 1H), 3.57 (dd, *J* = 17.2, 7.8 Hz, 1H), 2.77 (td, *J* = 10.2, 4.2 Hz, 1H), 1.93 (h, *J* = 8.0, 6.8 Hz, 1H), 1.72 (h, *J* = 6.9 Hz, 1H), 1.53 (ddt, *J* = 12.1, 7.2, 4.1 Hz, 2H), 1.46–1.20 (m, 8H), 1.05 (dtd, *J* = 18.1, 11.7, 10.8, 5.2 Hz, 2H), 0.98–0.92 (m, 3H), 0.85 (dd, *J* = 10.3, 6.7 Hz, 12H). ^13^C NMR (100 MHz, DMSO-*d*_6_) *δ* 171.4, 169.0, 168.7, 167.0, 138.6, 137.7, 118.7, 116.3, 76.1, 57.6, 48.8, 44.3, 33.6, 33.5, 31.5, 31.2, 28.9, 26.7, 22.4, 22.1, 19.3, 18.4, 13.9, 13.3, 11.1. HRMS(ESI) *m/z*: [M + H]^+^ calcd for C_25_H_42_N_3_O_5_^+^ 464.3119, found 464.3120.

*Benzyl (2S,3S,4R)-3-(((tert-butoxycarbonyl)-l-phenylalanyl)oxy)-2-methoxy-4-methyldecanoate (****6h****)*. To a solution of compound **7h** (1.08 g, 4.65 mmol, 2.5 equiv) and **8c** (600 mg, 1.86 mmol, 1.0 equiv) in CH_2_Cl_2_ (3.12 mL) was added DMAP (22.7 mg, 0.186 mmol, 0.1 equiv) and DIC (658 mg, 52.1 mmol, 2.8 equiv) under argon atmosphere at 0 °C. The reaction mixture was stirred for 1.5 h, and diluted with CH_2_Cl_2_ (50 mL). The suspension was filtered through a pad of silica gel and the filter cake was washed with CH_2_Cl_2_ (3 × 10 mL) and concentrated under reduced pressure. The residue was purified by column chromatography on silica gel (petroleum ether/ethyl acetate = 40:1 to 30:1) to obtain compound **6h** (879 mg, 83%) as colorless oil. ^1^H NMR (400 MHz, CDCl_3_) *δ* 7.43–7.09 (m, 10H), 5.17 (d, *J* = 11.1 Hz, 3H), 4.84 (d, *J* = 8.6 Hz, 1H), 4.49 (q, *J* = 7.1 Hz, 1H), 3.94 (d, *J* = 5.8 Hz, 1H), 3.37 (s, 3H), 3.08 (dd, *J* = 14.1, 5.8 Hz, 1H), 2.84 (dd, *J* = 14.1, 7.1 Hz, 1H), 1.93–1.86 (m, 1H), 1.38 (s, 9H), 1.30–1.04 (m, 10H), 0.91–0.82 (m, 6H). ^13^C NMR (100 MHz, CDCl_3_) *δ* 171.4, 170.0, 155.1, 136.4, 135.3, 129.5 (2C), 128.6 (4C), 128.6, 128.4 (2C), 126.8, 80.5, 79.7, 76.8, 67.0, 58.4, 54.4, 37.7, 33.5, 33.2, 31.8, 29.4, 28.3 (3C), 26.9, 22.7, 14.2, 14.1. HRMS (ESI) *m/z*: [M + Na]^+^ calcd for C_33_H_47_NO_7_Na^+^ 592.3245, found 592.3240.

*Benzyl (2S,3S,4R)-3-(((tert-butoxycarbonyl)-l-alanyl)oxy)-2-methoxy-4-methyldecanoate (****6i****).* The titled compound **6i** was obtained following the general procedure described for **6h**. Flash column chromatography eluent (petroleum ether/ethyl acetate = 9:1 to 5:1) to obtain **6i** (799 mg, 87%) as a colorless oil. ^1^H NMR (400 MHz, CDCl_3_) *δ* 7.40–7.24 (m, 5H), 5.18–5.11 (m, 3H), 4.99 (d, *J* = 8.0 Hz, 1H), 4.22 (p, *J* = 7.4 Hz, 1H), 3.90 (d, *J* = 6.8 Hz, 1H), 3.34 (s, 3H), 1.95–1.86 (m, 1H), 1.42 (s, 9H), 1.32–1.06 (m, 13H), 0.92–0.78 (m, 6H). ^13^C NMR (100 MHz, CDCl_3_) *δ* 172.6, 170.2, 155.1, 135.3, 128.7 (4C), 128.6, 80.4, 79.7, 76.2, 67.1, 58.5, 49.3, 33.4, 33.2, 31.8, 29.4, 28.4 (3C), 26.9, 22.7, 18.4, 14.1, 13.9. HRMS (ESI) *m/z*: [M + Na]^+^ calcd for C_27_H_43_NO_7_Na^+^ 516.2932, found 516.2931.

*Benzyl (2S,3S,4R)-3-(((S)-2-((tert-butoxycarbonyl)amino)-3,3-dimethylbutanoyl)oxy)-2-methoxy-4-methyldecanoate (****6j****).* The titled compound **6j** was obtained following the general procedure described for **6h**. Flash column chromatography eluent (petroleum ether/ethyl acetate = 9:1 to 5:1) to obtain **6j** (787 mg, 79%) as a colorless oil. ^1^H NMR (400 MHz, CDCl_3_) *δ* 7.43–7.27 (m, 5H), 5.24–5.16 (m, 3H), 5.15–5.09 (m, 1H), 4.16–4.05 (m, 1H), 4.00 (d, *J* = 5.8 Hz, 1H), 3.35 (s, 3H), 1.95–1.79 (m, 1H), 1.44 (s, 9H), 1.39–1.16 (m, 10H), 0.97 (s, 9H), 0.92 (d, *J* = 6.8 Hz, 3H), 0.87 (t, *J* = 6.9 Hz, 3H). ^13^C NMR (100 MHz, CDCl_3_) *δ* 171.5, 169.9, 155.5, 135.3, 128.6 (2C), 128.5 (2C), 128.5, 80.6, 79.6, 76.7, 66.9, 62.0, 58.5, 34.3, 33.6, 33.4, 31.8, 29.3, 28.3 (3C), 26.9, 26.4 (3C), 22.6, 14.4, 14.1. HRMS (ESI) *m/z*: [M + Na]^+^ calcd for C_30_H_49_NO_7_Na^+^ 558.3402, found 558.3399.

*Benzyl (2S,3S,4R)-3-(((tert-butoxycarbonyl)-l-leucyl)oxy)-2-methoxy-4-methyldecanoate (****6k****).* The titled compound **6k** was obtained following the general procedure described for **6h**. Flash column chromatography eluent (petroleum ether/ethyl acetate = 9:1 to 5:1) to obtain **6k** (847 mg, 85%) as a colorless oil. ^1^H NMR (400 MHz, CDCl_3_) *δ* 7.42–7.28 (m, 5H), 5.15 (s, 3H), 4.81 (d, *J* = 9.0 Hz, 1H), 4.25 (td, *J* = 9.3, 5.1 Hz, 1H), 3.91 (d, *J* = 6.6 Hz, 1H), 3.34 (s, 3H), 1.95–1.87 (m, 1H), 1.74–1.58 (m, 1H), 1.52 (ddd, *J* = 13.8, 8.6, 5.2 Hz, 1H), 1.42 (s, 9H), 1.37–1.03 (m, 11H), 0.87 (dd, *J* = 21.2, 6.9 Hz, 12H). ^13^C NMR (100 MHz, CDCl_3_) *δ* 172.7, 170.2, 155.4, 135.4, 128.7 (2C), 128.6 (2C), 128.5, 80.5, 79.7, 76.2, 67.0, 58.6, 52.3, 41.6, 33.5, 33.3, 31.8, 29.4, 28.4 (3C), 27.0, 24.8, 23.0, 22.7, 21.8, 14.2, 14.0. HRMS (ESI) *m/z*: [M + Na]^+^ calcd for C_30_H_49_NO_7_Na^+^ 558.3402, found 558.3398.

*Benzyl (2S,3S,4R)-3-(((tert-butoxycarbonyl)glycyl)oxy)-2-methoxy-4-methyldecanoate (****6l****).* The titled compound **6l** was obtained following the general procedure described for **6h**. Flash column chromatography eluent (petroleum ether/ethyl acetate = 9:1 to 5:1) to obtain **6l** (722 mg, 81%) as a colorless oil. ^1^H NMR (400 MHz, CDCl_3_) *δ* 7.40–7.25 (m, 5H), 5.21–5.09 (m, 3H), 4.81 (t, *J* = 5.6 Hz, 1H), 3.87 (d, *J* = 7.2 Hz, 1H), 3.77 (dd, *J* = 18.1, 6.1 Hz, 1H), 3.58 (dd, *J* = 18.3, 5.3 Hz, 1H), 3.37 (s, 3H), 1.97–1.88 (m, 1H), 1.43 (s, 9H), 1.30–1.18 (m, 10H), 0.90–0.79 (m, 6H). ^13^C NMR (100 MHz, CDCl_3_) *δ* 170.4, 169.7, 155.5, 135.3, 128.9 (2C), 128.7 (2C), 128.6, 80.1, 79.8, 76.2, 67.1, 58.6, 42.2, 34.7, 33.3, 31.8, 29.4, 28.4 (3C), 26.9, 22.6, 14.1, 13.7. HRMS (ESI) *m/z*: [M + Na]^+^ calcd for C_26_H_41_NO_7_Na^+^ 502.2776, found 502.2774.

*(2S,3S,4R)-2-Methoxy-4-methyl-1-oxo-1-(((R,E)-2,2,14,14-tetramethyl-9,12-dioxo-3,3-diphenyl-4,13-dioxa-10-aza-3-silapentadec-7-en-6-yl)amino)decan-3-yl* *(tert-butoxycarbonyl)-l-phenylalaninate (****4h****)*. To a solution of compound **6h** (569.7 mg, 1.0 mmol, 1.0 equiv) in MeOH (10 mL) was added Pd/C (10%, 30 mg) under N_2_. The suspension was degassed under vacuum and purged with H_2_ three times. The mixture was stirred under H_2_ balloon at 20 °C for 2 h. The resulting mixture was filtered through a short pad of silica gel, washed with MeOH (2 × 30 mL) and concentrated under reduced pressure. The crude product was used directly for the next step without further purification.

To a solution of above acid and **5** (728 mg, 1.5 mmol, 1.5 equiv) in CH_2_Cl_2_ (5 mL) was added EDCI (230 mg, 1.2 mmol, 1.2 equiv) and HOBt (148.6 mg, 1.1 mmol, 1.1 equiv) under argon atmosphere at 20 °C. The reaction mixture was stirred for 12 h, and then quenched with 1% HCl (100 mL). The aqueous phase was extracted with ethyl acetate (2 × 80 mL). The combined organic phases were dried (Na_2_SO_4_) and concentrated under reduced pressure. The crude product was purified by column chromatography on silica gel (petroleum ether/ethyl acetate = 9:1 to 5:1) to obtain **4h** (840 mg, 89% for 2 steps) as a colorless oil. ^1^H NMR (400 MHz, CDCl_3_) *δ* 7.61 (d*, J* = 7.1 Hz, 4H), 7.39 (ddd*, J* = 17.3, 7.3, 2.9 Hz, 6H), 7.30–7.09 (m, 6H), 6.82 (ddd*, J* = 15.4, 5.8, 2.2 Hz, 1H), 6.26 (d*, J* = 5.6 Hz, 1H), 5.97 (d*, J* = 15.4 Hz, 1H), 5.19 (td*, J* = 5.3, 2.1 Hz, 1H), 5.06 (d*, J* = 8.6 Hz, 1H), 4.52 (q*, J* = 7.5, 6.9 Hz, 2H), 4.01 (qdd*, J* = 18.3, 6.0, 3.0 Hz, 2H), 3.86–3.71 (m, 3H), 3.43–3.38 (m, 3H), 3.15 (dd*, J* = 14.3, 6.1 Hz, 1H), 2.92 (dd*, J* = 13.8, 7.2 Hz, 1H), 2.08–1.94 (m, 1H), 1.48 (d*, J* = 2.2 Hz, 9H), 1.41–1.35 (m, 9H), 1.24 (d*, J* = 13.4 Hz, 10H), 1.05 (d*, J* = 2.3 Hz, 9H), 0.92–0.82 (m, 6H). ^13^C NMR (100 MHz, CDCl_3_) *δ* 171.9, 169.2, 169.1, 165.1, 155.3, 141.0, 136.5, 135.7 (2C), 135.6 (2C), 132.8, 132.7, 130.0, 130.0, 129.5 (2C), 128.5 (2C), 127.9 (4C), 126.9, 124.6, 82.4, 81.6, 79.8, 77.0, 64.9, 59.1, 54.8, 52.0, 42.2, 37.8, 33.5 (2C), 31.9, 29.5, 28.3 (3C), 28.2 (3C), 26.9 (4C), 22.7, 19.3, 14.3, 14.2. HRMS (ESI) *m/z*: [M + Na]^+^ calcd for C_53_H_77_N_3_O_10_SiNa^+^ 966.5271, found 966.5268.

*(2S,3S,4R)-2-Methoxy-4-methyl-1-oxo-1-(((R,E)-2,2,14,14-tetramethyl-9,12-dioxo-3,3-diphenyl-4,13-dioxa-10-aza-3-silapentadec-7-en-6-yl)amino)decan-3-yl* *(tert-butoxycarbonyl)-l-alaninate (****4i****).* The titled compound **4i** was obtained following the general procedure described for **4h**. Flash column chromatography eluent (petroleum ether/ethyl acetate = 9:1 to 5:1) to obtain **4i** (965 mg, 82% for 2 steps) as a colorless oil. ^1^H NMR (400 MHz, CDCl_3_) *δ* 7.62 (ddt, *J* = 8.4, 7.0, 1.6 Hz, 4H), 7.39 (dddd, *J* = 12.5, 8.5, 5.9, 2.4 Hz, 6H), 7.14 (d, *J* = 8.1 Hz, 1H), 6.81 (dd, *J* = 15.4, 5.9 Hz, 1H), 6.20 (t, *J* = 5.1 Hz, 1H), 5.95 (dd, *J* = 15.4, 1.5 Hz, 1H), 5.13 (t, *J* = 5.4 Hz, 2H), 4.57–4.48 (m, 1H), 4.24 (p, *J* = 8.5, 7.9 Hz, 1H), 3.99 (qd, *J* = 18.5, 5.1 Hz, 2H), 3.84–3.71 (m, 3H), 3.44 (s, 3H), 2.02 (dd, *J* = 12.5, 6.2 Hz, 1H), 1.44 (d, *J* = 18.3 Hz, 18H), 1.31–1.19 (m, 13H), 1.05 (s, 9H), 0.91 (d, *J* = 6.8 Hz, 3H), 0.84 (t, *J* = 6.7 Hz, 3H). ^13^C NMR (100 MHz, CDCl_3_) *δ* 173.0, 169.2, 169.2, 165.1, 155.2, 141.1, 135.7 (2C), 135.6 (2C), 132.8, 132.7, 130.0 (2C), 128.0 (4C), 124.6, 82.5, 81.5, 79.7, 76.5, 65.0, 59.1, 51.7, 49.4, 42.2, 33.4, 33.4, 31.9, 29.5, 28.4 (3C), 28.1 (3C), 26.9 (4C), 22.7, 19.3, 18.4, 14.3, 14.2. HRMS (ESI) *m/z*: [M + Na]^+^ calcd for C_47_H_73_N_3_O_10_SiNa^+^ 890.4958, found 590.4952.

*(2S,3S,4R)-2-Methoxy-4-methyl-1-oxo-1-(((R,E)-2,2,14,14-tetramethyl-9,12-dioxo-3,3-diphenyl-4,13-dioxa-10-aza-3-silapentadec-7-en-6-yl)amino)decan-3-yl* *(S)-2-((tert-butoxycarbonyl)amino)-3,3-dimethylbutanoate (****4j****).* The titled compound **4j** was obtained following the general procedure described for **4h**. Flash column chromatography eluent (petroleum ether/ethyl acetate = 9:1 to 5:1) to obtain **4j** (1.02 g, 81% for 2 steps) as a colorless oil. ^1^H NMR (400 MHz, CDCl_3_) *δ* 7.62 (tt, *J* = 7.7, 1.6 Hz, 4H), 7.46–7.33 (m, 6H), 7.21 (d, *J* = 8.2 Hz, 1H), 6.82 (dd, *J* = 15.4, 6.1 Hz, 1H), 6.11 (t, *J* = 5.0 Hz, 1H), 5.97 (dd, *J* = 15.3, 1.5 Hz, 1H), 5.22 (t, *J* = 4.8 Hz, 1H), 5.10 (d, *J* = 9.9 Hz, 1H), 4.57 (s, 1H), 4.08 (d, *J* = 9.9 Hz, 1H), 3.99 (d, *J* = 5.1 Hz, 2H), 3.88 (d, *J* = 4.4 Hz, 1H), 3.78 (q, *J* = 5.4, 4.4 Hz, 2H), 3.47 (s, 3H), 1.99 (s, 1H), 1.47 (s, 9H), 1.41 (s, 9H), 1.27–1.18 (m, 10H), 1.06 (s, 9H), 0.93 (d, *J* = 18.1 Hz, 12H), 0.84 (t, *J* = 6.7 Hz, 3H). ^13^C NMR (100 MHz, CDCl_3_) *δ* 171.8, 169.1, 168.9, 165.1, 155.7, 141.4, 135.8 (2C), 135.7 (2C), 132.9, 132.7, 130.1, 130.0, 128.0 (2C), 128.0 (2C), 124.7, 82.5, 82.4, 79.8, 77.2, 65.1, 62.2, 59.3, 51.6, 42.3, 34.4, 33.8, 33.7, 31.9, 29.6, 28.4 (3C), 28.2 (3C), 26.9((3C), 26.8, 26.6 (3C), 22.7, 19.4, 14.9, 14.2. HRMS (ESI) *m/z*: [M + H]^+^ calcd for C_50_H_80_N_3_O_10_Si^+^ 910.5608, found 910.5605.

*(2S,3S,4R)-2-Methoxy-4-methyl-1-oxo-1-(((R,E)-2,2,14,14-tetramethyl-9,12-dioxo-3,3-diphenyl-4,13-dioxa-10-aza-3-silapentadec-7-en-6-yl)amino)decan-3-yl* *(tert-butoxycarbonyl)-l-leucinate (****4k****)*. The titled compound **4k** was obtained following the general procedure described for **4h**. Flash column chromatography eluent (petroleum ether/ethyl acetate = 9:1 to 5:1) to obtain **4k** (802 mg, 86% for 2 steps) as a colorless oil. ^1^H NMR (400 MHz, CDCl_3_) *δ* 7.68–7.58 (m, 4H), 7.39 (tt, *J* = 8.8, 4.6 Hz, 6H), 7.12 (d, *J* = 8.1 Hz, 1H), 6.81 (dd, *J* = 15.4, 5.8 Hz, 1H), 6.18 (t, *J* = 5.1 Hz, 1H), 5.96 (dd, *J* = 15.5, 1.3 Hz, 1H), 5.16 (t, *J* = 5.2 Hz, 1H), 4.98 (d, *J* = 8.9 Hz, 1H), 4.62–4.47 (m, 1H), 4.25 (td, *J* = 9.3, 4.7 Hz, 1H), 4.00 (qd, *J* = 18.5, 5.0 Hz, 2H), 3.79 (dtd, *J* = 15.0, 10.4, 4.5 Hz, 3H), 3.45 (s, 3H), 1.99–1.94 (m, 1H), 1.74–1.55 (m, 3H), 1.47 (s, 9H), 1.42 (s, 9H), 1.32–1.18 (m, 10H), 1.06 (s, 9H), 0.96–0.80 (m, 12H). ^13^C NMR (100 MHz, CDCl_3_) *δ* 172.9, 169.3, 169.2, 165.1, 155.6, 141.1, 135.7 (2C), 135.7 (2C), 132.9, 132.8, 130.0 (2C), 128.0 (4C), 124.7, 82.5, 82.0, 79.8, 76.6, 65.0, 59.4, 52.5, 51.9, 42.22, 41.2, 33.6, 33.6, 31.9, 30.0, 28.5 (3C), 28.2 (3C), 27.0 (4C), 24.9, 23.0, 22.7, 21.9, 19.4, 14.4, 14.2. HRMS (ESI) *m/z*: [M + H]^+^ calcd for C_50_H_80_N_3_O_10_Si^+^ 910.5608, found 910.5612.

*(2S,3S,4R)-1-(((R,E)-5-((2-(tert-Butoxy)-2-oxoethyl)amino)-1-((tert-butyldiphenylsilyl)oxy)-5-oxopent-3-en-2-yl)amino)-2-methoxy-4-methyl-1-oxodecan-3-yl* *(tert-butoxycarbonyl)glycinate (****4l****).* The titled compound **4l** was obtained following the general procedure described for **4h**. Flash column chromatography eluent (petroleum ether/ethyl acetate = 9:1 to 5:1) to obtain **4l** (985 mg, 79% for 2 steps) as a colorless oil. ^1^H NMR (400 MHz, CDCl_3_) *δ* 7.62 (t, *J* = 7.0 Hz, 4H), 7.50–7.32 (m, 6H), 7.17 (d, *J* = 8.2 Hz, 1H), 6.81 (dd, *J* = 15.3, 6.0 Hz, 1H), 6.23 (t, *J* = 5.2 Hz, 1H), 5.96 (d, *J* = 15.3 Hz, 1H), 5.11 (q, *J* = 7.0, 6.3 Hz, 2H), 4.54 (d, *J* = 8.0 Hz, 1H), 3.98 (dd, *J* = 5.1, 3.3 Hz, 2H), 3.89–3.69 (m, 5H), 3.43 (s, 3H), 2.00–1.96 (m, 1H), 1.46 (s, 9H), 1.41 (s, 9H), 1.23 (d, *J* = 8.3 Hz, 9H), 1.05 (s, 10H), 0.90 (d, *J* = 6.7 Hz, 3H), 0.84 (t, *J* = 6.6 Hz, 3H). ^13^C NMR (100 MHz, CDCl_3_) *δ* 170.0, 169.3, 169.1, 165.1, 155.7, 141.0, 135.6 (2C), 135.5 (2C), 132.8, 132.7, 129.9 (2C), 127.9 (4C), 124.7, 82.2, 81.3, 79.7, 76.6, 65.0, 59.1, 51.5, 42.5, 42.1, 33.4, 33.2, 31.8, 29.4, 28.3 (3C), 28.0 (3C), 26.8 (3C), 26.8, 22.6, 19.3, 14.2, 14.1. HRMS (ESI) *m/z*: [M + Na]^+^ calcd for C_46_H_71_N_3_O_10_SiNa^+^ 876.4801, found 876.4797.

*(3S,11R,14S,15S,E)-3-Benzyl-11-(((tert-butyldiphenylsilyl)oxy)methyl)-14-methoxy-15-((R)-octan-2*-*yl)-1-oxa-4,7,12-triazacyclopentadec-9-ene-2,5,8,13-tetraone (****3h****).* To a solution of **4h** (815 mg, 0.863 mmol, 1.0 equiv) in CH_2_Cl_2_ (7 mL) was added trifluoroacetic acid (1.7 mL) at 0 °C. After stirred for 4 h, the reaction solution was diluted with toluene (7 mL). And the resulting solution was concentrated under reduced pressure. The resulting white solid was dissolved in THF (20 mL), and the solution was added slowly to a suspension of HATU (4.92 g, 12.95 mmol, 15.0 equiv) and DIPEA (4.1 mL, 25.9 mmol, 30.0 equiv) in THF (900 mL) over 18 h at room temperature and then continued to stirred another 12h at room temperature. The solvent was evaporated, diluted with MeOH and EtOAc (*v/v* = 2:1, 300 mL) and filtered through a short pad of silica gel. The filtrate was concentrated under reduced pressure. The residue was dissolved in EtOAc (300 mL), washed with 1% HCl (80 mL), saturated aqueous solution of NaHCO_3_ (60 mL), and brine (60 mL). The organic phase was dried (Na_2_SO_4_) and concentrated under reduced pressure. The crude product was purified by column chromatography on silica gel (dichloromethane/methanol = 50:1 to 9:1) to obtain **3h** (458 mg, 69% for 2 steps) as a white solid. ^1^H NMR (400 MHz, DMSO-*d*_6_) *δ* 8.45 (dd, *J* = 9.2, 6.1 Hz, 2H), 7.64–7.59 (m, 4H), 7.44 (ddt, *J* = 13.5, 10.2, 4.6 Hz, 6H), 7.32–7.16 (m, 5H), 6.73 (dd, *J* = 15.1, 3.0 Hz, 1H), 5.91 (dd, *J* = 15.2, 2.3 Hz, 1H), 5.27 (dd, *J* = 9.7, 1.8 Hz, 1H), 4.78–4.72 (m, 1H), 4.65 (td, *J* = 9.8, 4.0 Hz, 1H), 3.97–3.88 (m, 2H), 3.64 (d, *J* = 6.6 Hz, 2H), 3.36 (s, 2H), 3.25 (s, 3H), 3.02 (dd, *J* = 13.9, 4.1 Hz, 1H), 2.85 (dd, *J* = 13.8, 10.5 Hz, 1H), 1.89 (q, *J* = 7.1 Hz, 1H), 1.30–1.21 (m, 10H), 0.98 (d, *J* = 11.7 Hz, 12H), 0.85 (t, *J* = 6.7 Hz, 3H). ^13^C NMR (100 MHz, DMSO-*d*_6_) *δ* 169.5, 168.3, 167.7, 166.4, 141.3, 136.8, 135.1 (2C), 135.0 (2C), 132.6, 132.6, 129.9, 129.9, 128.9 (2C), 128.2 (2C), 127.9 (4C), 126.6, 119.5, 78.4, 73.6, 65.5, 56.7, 53.5, 51.4, 43.9, 38.7, 33.1, 33.0, 31.2, 28.8, 26.7, 26.6 (3C), 22.1, 18.8, 13.9, 13.7. HRMS (ESI) *m/z*: [M + H]^+^ calcd for C_44_H_60_N_3_O_7_Si^+^ 770.4196, found 770.4194.

*(3S,11R,14S,15S,E)-11-(((tert-Butyldiphenylsilyl)oxy)methyl)-14-methoxy-3-methyl-15-((R)-octan-2*-*yl)-1-oxa-4,7,12-triazacyclopentadec-9-ene-2,5,8,13-tetraone (****3i****).* The titled compound **3i** was obtained following the general procedure described for **3h**. Flash column chromatography eluent (dichloromethane/methanol = 50:1 to 9:1) to obtain **3i** (501 mg, 72% for 2 steps) as a white solid. ^1^H NMR (400 MHz, DMSO-*d*_6_) *δ* 8.46 (dd, *J* = 8.8, 3.3 Hz, 2H), 7.62 (dd, *J* = 7.3, 4.0 Hz, 4H), 7.52 (t, *J* = 6.6 Hz, 1H), 7.44 (qq, *J* = 8.6, 4.5, 3.0 Hz, 6H), 6.75 (dd, *J* = 15.1, 3.0 Hz, 1H), 5.90 (dd, *J* = 15.1, 2.2 Hz, 1H), 5.23 (d, *J* = 9.6 Hz, 1H), 4.78–4.70 (m, 1H), 4.43 (p, *J* = 7.4 Hz, 1H), 3.91 (q, *J* = 7.6, 6.5 Hz, 2H), 3.71–3.52 (m, 3H), 3.24 (s, 3H), 1.88 (q, *J* = 7.0 Hz, 1H), 1.38–1.17 (m, 13H), 0.98 (d, *J* = 11.9 Hz, 12H), 0.86 (t, *J* = 6.7 Hz, 3H). ^13^C NMR (100 MHz, DMSO-*d*_6_) *δ* 170.8, 168.0, 167.7, 166.5, 141.2, 135.1 (2C), 135.0 (2C), 132.7, 132.6, 130.0, 130.0, 127.9 (4C), 119.5, 78.4, 73.2, 65.5, 56.7, 51.4, 48.0, 44.0, 33.3, 32.9, 31.1, 28.8, 26.7, 26.6 (3C), 22.0, 19.2, 18.8, 13.9, 13.6. HRMS (ESI) *m/z*: [M + H]^+^ calcd for C_38_H_56_N_3_O_7_Si^+^ 694.3883, found 694.3881.

*(3S,11R,14S,15S,E)-3-(tert-Butyl)-11-(((tert-butyldiphenylsilyl)oxy)methyl)-14-methoxy-15-((R)-octan-2*-*yl)-1-oxa-4,7,12-triazacyclopentadec-9-ene-2,5,8,13-tetraone (****3j****).* The titled compound **3j** was obtained following the general procedure described for **3h**. Flash column chromatography eluent (dichloromethane/methanol = 50:1 to 9:1) to obtain **3j** (609 mg, 77% for 2 steps) as a white solid. ^1^H NMR (400 MHz, DMSO-*d*_6_) *δ* 8.30 (d, *J* = 8.8 Hz, 1H), 8.07 (d, *J* = 10.2 Hz, 1H), 7.63–7.56 (m, 5H), 7.50–7.41 (m, 6H), 6.72 (dd, *J* = 15.1, 3.0 Hz, 1H), 5.95 (d, *J* = 15.2 Hz, 1H), 5.38 (d, *J* = 9.7 Hz, 1H), 4.71 (d, *J* = 7.8 Hz, 1H), 4.33 (d, *J* = 10.1 Hz, 1H), 4.12 (dd, *J* = 17.5, 5.9 Hz, 1H), 3.91 (d, *J* = 9.7 Hz, 1H), 3.65 (d, *J* = 6.6 Hz, 2H), 3.54 (dd, *J* = 17.5, 7.6 Hz, 1H), 3.24 (s, 3H), 1.87 (q, *J* = 6.9 Hz, 1H), 1.39–1.20 (m, 8H), 1.12 (d, *J* = 11.3 Hz, 2H), 1.03–0.90 (m, 21H), 0.85 (t, *J* = 6.6 Hz, 3H). ^13^C NMR (100 MHz, DMSO-*d*_6_) *δ* 168.9, 167.7, 167.5, 166.3, 141.5, 135.1 (2C), 135.0 (2C), 132.6, 132.6, 130.0 (2C), 128.0 (4C), 119.5, 78.0, 73.2, 65.3, 60.2, 56.4, 51.6, 44.0, 34.9, 33.0, 32.9, 31.1, 28.9, 26.75, 26.6 (6C), 22.0, 18.8, 14.0, 13.9. HRMS (ESI) *m/z*: [M + H]^+^ calcd for C_41_H_62_N_3_O_7_Si^+^ 736.4352, found 736.4354.

*(3S,11R,14S,15S,E)-11-(((tert-Butyldiphenylsilyl)oxy)methyl)-3-isobutyl-14-methoxy-15-((R)-octan-2*-*yl)-1-oxa-4,7,12-triazacyclopentadec-9-ene-2,5,8,13-tetraone (****3k****).* The titled compound **3k** was obtained following the general procedure described for **3h**. Flash column chromatography eluent (dichloromethane/methanol = 50:1 to 9:1) to obtain **3k** (442 mg, 74% for 2 steps) as a white solid. ^1^H NMR (400 MHz, DMSO-*d*_6_) *δ* 8.44 (d, *J* = 9.1 Hz, 1H), 8.35 (d, *J* = 9.1 Hz, 1H), 7.61 (ddd, *J* = 7.9, 3.8, 1.7 Hz, 4H), 7.52 (dd, *J* = 7.5, 5.9 Hz, 1H), 7.49–7.38 (m, 6H), 6.74 (dd, *J* = 15.1, 3.0 Hz, 1H), 5.89 (dd, *J* = 15.2, 2.3 Hz, 1H), 5.24 (dd, *J* = 9.7, 1.8 Hz, 1H), 4.73 (dp, *J* = 9.3, 3.8 Hz, 1H), 4.44 (td, *J* = 9.6, 5.0 Hz, 1H), 3.95–3.85 (m, 2H), 3.66–3.53 (m, 3H), 3.23 (s, 3H), 1.86 (q, *J* = 7.5, 6.9 Hz, 1H), 1.63–1.50 (m, 2H), 1.43 (ddd, *J* = 13.5, 8.9, 5.1 Hz, 1H), 1.35–1.15 (m, *J* = 6.4, 5.8 Hz, 10H), 0.99 (s, 9H), 0.92 (dd, *J* = 20.1, 6.6 Hz, 6H), 0.85 (q, *J* = 6.6 Hz, 6H). ^13^C NMR (100 MHz, DMSO-*d*_6_) *δ* 170.3, 168.3, 167.7, 166.4, 141.4, 135.1 (2C), 135.0 (2C), 132.6, 132.6, 129.9, 129.9, 127.9 (4C), 119.4, 78.3, 73.0, 65.5, 56.7, 51.4, 50.6, 44.0, 42.0, 33.1, 32.9, 31.1, 28.8, 26.6, 26.6 (3C), 24.1, 22.6, 22.0, 21.1, 18.8, 13.9, 13.6. HRMS (ESI) *m/z*: [M + H]^+^ calcd for C_41_H_62_N_3_O_7_Si^+^ 736.4352, found 736.4347.

*(11R,14S,15S,E)-11-(((tert-Butyldiphenylsilyl)oxy)methyl)-14-methoxy-15-((R)-octan-2-yl)-1-oxa-4,7,12-triazacyclopentadec-9-ene-2,5,8,13-tetraone (****3l****).* The titled compound **3l** was obtained following the general procedure described for **3h**. Flash column chromatography eluent (dichloromethane/methanol = 50:1 to 9:1) to obtain **3l** (533 mg, 83% for 2 steps) as a white solid. ^1^H NMR (400 MHz, DMSO-*d*_6_) *δ* 8.48 (d, *J* = 8.9 Hz, 1H), 8.40 (t, *J* = 6.2 Hz, 1H), 7.62 (ddd, *J* = 8.0, 4.5, 1.6 Hz, 4H), 7.54 (t, *J* = 6.6 Hz, 1H), 7.43 (dddd, *J* = 15.8, 8.4, 4.2, 1.9 Hz, 6H), 6.78 (dd, *J* = 15.1, 3.3 Hz, 1H), 5.99 (dd, *J* = 15.2, 2.2 Hz, 1H), 5.14 (dd, *J* = 9.4, 2.1 Hz, 1H), 4.73 (dh, *J* = 6.2, 3.5 Hz, 1H), 3.99–3.82 (m, 4H), 3.73–3.56 (m, 3H), 3.23 (s, 3H), 1.87 (q, *J* = 6.5, 5.3 Hz, 1H), 1.31–1.21 (m, 10H), 0.99 (s, 9H), 0.92 (d, *J* = 6.9 Hz, 3H), 0.89–0.81 (m, 3H). ^13^C NMR (100 MHz, DMSO-*d*_6_) *δ* 168.7, 168.2, 167.7, 166.6, 141.5, 135.1 (2C), 135.1 (2C), 132.7, 132.6, 129.9, 129.9, 127.9 (4C), 120.0, 78.8, 74.2, 65.3, 56.7, 51.6, 44.4, 41.0, 33.6, 33.1, 31.3, 28.9, 26.8, 26.6 (3C), 22.1, 18.8, 13.9, 13.8. HRMS (ESI) *m/z*: [M + H]^+^ calcd for C_37_H_54_N_3_O_7_Si^+^ 680.3726, found 680.3723.

*(3S,14S,15S,E)-3-Benzyl-14-methoxy-11-methylene-15-((R)-octan-2-yl)-1-oxa-4,7,12-triazacyclopentadec-9-ene-2,5,8,13-tetraone (****1h****).* To a solution of compound **3h** (312 mg, 1.0 mmol, 1.0 equiv) in THF (10 mL) were added HOAc (72 mmg, 1.2 mmol, 3.0 equiv) and TBAF (1.2 mL, 1 mol/L in THF, 1.2 mmol, 3.0 equiv). The mixture was stirred at room temperature for 24 h, and then concentrated. The residue was purified by column chromatography on silica gel (dichloromethane/methanol = 100:3 to 9:1) to obtain **2h** as a white powder.

To a solution of compound **2h** (140 mg, 0.263 mmol, 1.0 equiv) in THF (2.48 mL), then triethylamine (80 mg, 0.79 mmol, 3.0 equiv) and ethanesulfonyl chloride (65 mg, 0.658 mmol, 2.5 equiv) were added at 0 °C. After stirred for 2 h, the reaction solution was quenched by addition of water (0.1 mL), dried over Na_2_SO_4_, filtered and concentrated under reduced pressure. The resulting crude product was dissolved in THF (5.0 mL). To the resulting solution was added DBU (321 mg, 2.11 mmol, 8 equiv) at 20 °C. After stirred for 2.5 h, the solution was dissolved in ethyl acetate (80 mL) and washed successively with 1% HCl (3 mL), saturated aqueous NaHCO_3_ (3 mL), brine (3 mL), dried over Na_2_SO_4_ and concentrated under reduced pressure carefully. The crude product was purified by column chromatography on silica gel (dichloromethane/methanol = 100: 1 to 100: 6), *t*-BuOH (3 mL) was added to the eluent and concentrated to remove CH_2_Cl_2_ and MeOH. The residual solution was lyophilized to give **1h** (82 mg, 57% for 3 steps) as a white solid. ^1^H NMR (400 MHz, DMSO-*d*_6_) *δ* 9.29 (s, 1H), 8.57 (d*, J* = 9.5 Hz, 1H), 7.56 (dd*, J* = 7.6, 5.3 Hz, 1H), 7.30–7.15 (m, 5H), 6.85 (d*, J* = 15.3 Hz, 1H), 6.22 (dd*, J* = 15.3, 3.2 Hz, 1H), 5.61 (s, 1H), 5.44 (s, 1H), 5.15 (dd*, J* = 8.5, 3.2 Hz, 1H), 4.68 (td*, J* = 9.8, 4.3 Hz, 1H), 4.19 (d*, J* = 8.9 Hz, 1H), 4.05 (dd*, J* = 17.8, 5.0 Hz, 1H), 3.35 (d*, J* = 3.1 Hz, 1H), 3.30 (s, 3H), 3.03 (dd*, J* = 13.7, 4.4 Hz, 1H), 2.85 (dd*, J* = 13.7, 10.1 Hz, 1H), 1.93 (s, 1H), 1.42–1.21 (m, 10H), 0.94 (d*, J* = 7.1 Hz, 3H), 0.85 (t*, J* = 6.8 Hz, 3H). ^13^C NMR (100 MHz, DMSO-*d*_6_) *δ* 169.6, 168.5, 168.3, 167.0, 138.0, 137.4, 136.8, 129.0 (2C), 128.1 (2C), 126.6, 119.3, 116.0, 78.9, 74.7, 56.9, 53.5, 44.1, 38.8, 33.1, 32.7, 31.2, 28.8, 26.5, 22.0, 13.9, 13.8. HRMS (ESI) *m/z*: [M + H]^+^ calcd for C_28_H_40_N_3_O_6_Si^+^ 514.2912, found 514.2907.

*(3S,14S,15S,E)-14-Methoxy-3-methyl-11-methylene-15-((R)-octan-2-yl)-1-oxa-4,7,12-triazacyclopentadec-9-ene-2,5,8,13-tetraone (****1i****)*. The titled compound **1i** was obtained following the general procedure described for **1h**. The crude product was purified by column chromatography on silica gel (dichloromethane/methanol = 100: 1 to 100: 6), *t-*BuOH (3 mL) was added to the eluent and concentrated to remove CH_2_Cl_2_ and MeOH. The residual solution was lyophilized to give **1i** (115 mg, 51% for 3 steps) as a white solid. ^1^H NMR (400 MHz, DMSO-*d*_6_) *δ* 9.14 (s, 1H), 8.45 (d, *J* = 8.8 Hz, 1H), 7.65 (dd, *J* = 7.7, 5.2 Hz, 1H), 6.90 (d, *J* = 15.2 Hz, 1H), 6.06 (d, *J* = 15.2 Hz, 1H), 5.57 (s, 1H), 5.48 (s, 1H), 5.14 (dd, *J* = 8.4, 3.3 Hz, 1H), 4.56–4.44 (m, 1H), 4.06–3.94 (m, 1H), 3.89 (dd, *J* = 17.4, 5.2 Hz, 1H), 3.57 (dd, *J* = 17.4, 7.7 Hz, 1H), 3.30 (s, 3H), 1.95–1.90 (m, 1H), 1.33–1.19 (m, 13H), 0.94 (d, *J* = 6.8 Hz, 3H), 0.86 (t, *J* = 6.9 Hz, 3H). ^13^C NMR (100 MHz, DMSO-*d*_6_) *δ* 171.0, 168.2, 168.1, 167.1, 138.1, 137.3, 119.1, 116.6, 79.1, 74.3, 57.0, 47.8, 44.3, 33.3, 32.7, 31.2, 28.9, 26.5, 22.1, 19.4, 14.0, 13.8. HRMS (ESI) *m/z*: [M + H]^+^ calcd for C_22_H_36_N_3_O_6_^+^ 438.2599, found 438.2595.

*(3S,14S,15S,E)-3-(tert-Butyl)-14-methoxy-11-methylene-15-((R)-octan-2-yl)-1-oxa-4,7,12-triazacyclopentadec-9-ene-2,5,8,13-tetraone (****1j****).* The titled compound **1j** was obtained following the general procedure described for **1h**. The crude product was purified by column chromatography on silica gel (dichloromethane/methanol = 100: 1 to 100: 6), *t-*BuOH (3 mL) was added to the eluent and concentrated to remove CH_2_Cl_2_ and MeOH. The residual solution was lyophilized to give **1j** (57 mg, 49% for 3 steps) as a white solid. ^1^H NMR (400 MHz, DMSO-*d*_6_) *δ* 9.23 (s, 1H), 8.11 (d, *J* = 10.3 Hz, 1H), 7.73–7.63 (m, 1H), 6.92 (d, *J* = 15.2 Hz, 1H), 6.11 (d, *J* = 15.2 Hz, 1H), 5.52 (d, *J* = 13.8 Hz, 2H), 5.29 (dd, *J* = 9.2, 2.2 Hz, 1H), 4.37 (d, *J* = 10.3 Hz, 1H), 4.13 (dd, *J* = 17.0, 5.7 Hz, 1H), 4.02 (d, *J* = 8.3 Hz, 1H), 3.55 (dd, *J* = 17.2, 7.7 Hz, 1H), 3.30 (s, 3H), 2.00–1.82 (m, 1H), 1.58 (dddd, *J* = 34.8, 12.4, 8.8, 6.6 Hz, 2H), 1.35–1.22 (m, 8H), 0.98–0.90 (m, 12H), 0.88–0.83 (m, 3H). ^13^C NMR (100 MHz, DMSO-*d*_6_) *δ* 169.1, 167.9, 167.8, 166.7, 138.2, 137.7, 118.8, 117.1, 78.5, 73.9, 60.2, 56.5, 44.2, 35.0, 33.1, 32.7, 31.1, 28.9, 26.7, 26.6 (3C), 22.0, 14.1, 13.9. HRMS (ESI) *m/z*: [M + H]^+^ calcd for C_25_H_42_N_3_O_6_^+^ 480.3069, found 480.3065.

*(3S,14S,15S,E)-3-Isobutyl-14-methoxy-11-methylene-15-((R)-octan-2-yl)-1-oxa-4,7,12-triazacyclopentadec-9-ene-2,5,8,13-tetraone (****1k****).* The titled compound **1k** was obtained following the general procedure described for **1h**. The crude product was purified by column chromatography on silica gel (dichloromethane/methanol = 100: 1 to 100: 6), *t-*BuOH (3 mL) was added to the eluent and concentrated to remove CH_2_Cl_2_ and MeOH. The residual solution was lyophilized to give **1k** (105 mg, 51% for 3 steps) as a white solid. ^1^H NMR (400 MHz, DMSO-*d*_6_) *δ* 9.21 (s, 1H), 8.37 (d, *J* = 9.4 Hz, 1H), 7.63 (dd, *J* = 7.7, 5.3 Hz, 1H), 6.88 (d, *J* = 15.2 Hz, 1H), 6.06 (d, *J* = 15.2 Hz, 1H), 5.59 (s, 1H), 5.47 (s, 1H), 5.15 (dd, *J* = 8.8, 2.9 Hz, 1H), 4.50 (td, *J* = 9.6, 4.9 Hz, 1H), 4.00 (d, *J* = 8.8 Hz, 1H), 3.91 (dd, *J* = 17.4, 5.3 Hz, 1H), 3.59 (dd, *J* = 17.4, 7.8 Hz, 1H), 3.30 (s, 3H), 1.92 (d, *J* = 7.2 Hz, 1H), 1.61–1.50 (m, 2H), 1.45 (dq, *J* = 9.8, 5.6 Hz, 1H), 1.25 (d, *J* = 17.1 Hz, 10H), 0.94 (d, *J* = 6.8 Hz, 3H), 0.92–0.78 (m, 9H). ^13^C NMR (100 MHz, DMSO-*d*_6_) *δ* 170.5, 168.5, 168.0, 167.0, 138.0, 137.2, 119.0, 116.3, 78.9, 73.9, 56.8, 50.5, 44.3, 42.2, 33.2, 32.7, 31.1, 28.8, 26.5, 24.1, 22.6, 22.1, 21.1, 13.9, 13.7. HRMS (ESI) *m/z*: [M + H]^+^ calcd for C_25_H_42_N_3_O_6_^+^ 480.3069, found 480.3063.

*(14S,15S,E)-14-Methoxy-11-methylene-15-((R)-octan-2-yl)-1-oxa-4,7,12-triazacyclopentadec-9-ene-2,5,8,13-tetraone (****1l****).* The titled compound **1l** was obtained following the general procedure described for **1h**. The crude product was purified by column chromatography on silica gel (dichloromethane/methanol = 100: 1 to 100: 6), *t-*BuOH (3 mL) was added to the eluent and concentrated to remove CH_2_Cl_2_ and MeOH. The residual solution was lyophilized to give **1l** (100 mg, 43% for 3 steps) as a white solid. ^1^H NMR (400 MHz, DMSO-*d*_6_) *δ* 8.93 (s, 1H), 8.48 (dd, *J* = 7.1, 5.1 Hz, 1H), 7.66 (q, *J* = 6.1 Hz, 1H), 6.93 (d, *J* = 15.2 Hz, 1H), 6.11 (d, *J* = 15.3 Hz, 1H), 5.52 (d, *J* = 7.7 Hz, 2H), 5.13 (dd, *J* = 6.8, 5.3 Hz, 1H), 4.02 (dd, *J* = 10.5, 6.9 Hz, 2H), 3.87 (ddd, *J* = 36.4, 17.4, 5.4 Hz, 2H), 3.61 (dd, *J* = 17.5, 7.1 Hz, 1H), 3.31 (s, 3H), 1.90–1.80 (m, 1H), 1.26–1.21 (m, 10H), 0.86 (dt, *J* = 7.2, 3.4 Hz, 6H). ^13^C NMR (100 MHz, DMSO-*d*_6_) *δ* 169.0, 168.7, 167.8, 167.5, 138.2, 137.5, 119.8, 116.9, 79.7, 75.3, 57.1, 44.6, 41.0, 33.4, 32.7, 31.2, 28.9, 26.3, 22.1, 14.2, 13.9. HRMS (ESI) *m/z*: [M + H]^+^ calcd for C_21_H_34_N_3_O_6_^+^ 424.2443, found 424.2437.

*Allyl (2S,3S,4R)-3-hydroxy-2-methoxy-4-methyldecanoate (****13****).* To a solution of compound **8c** (1.0 g, 3.10 mmol, 1.0 equiv) in MeOH (30 mL) was added Pd/C (10%, 100 mg) under N_2_. The suspension was degassed under vacuum and purged with H_2_ three times. The mixture was stirred under H_2_ balloon at 20 °C for 5 h. The resulting mixture was filtered through a short pad of silica gel, washed with MeOH (2 × 30 mL) and concentrated under reduced pressure. The crude product was used directly for the next step without further purification.

The above colorless oil was dissolved in DMF (30 mL) was added K_2_CO_3_ (857.0 mg, 6.2 mmol, 2.0 equiv) and allyl bromide (402.0 μL, 4.65 mmol, 1.5 equiv) at room temperature. The mixture was stirred at room temperature for 10 h, and then H_2_O (10 mL) was added, and the resultant mixture was extracted with ethyl acetate (3 × 50 mL). The organic phase was washed by brine (4 × 10 mL). Dried over MgSO_4_, filtered and concentrated under reduced pressure. The residue was purified with column chromatography on silica gel (petroleum ether: ethyl acetate = 10:1 to 6:1) to afford compound **13** (810.0 mg, 96% for 2 steps) as a colorless oil. ^1^H NMR (400 MHz,CDCl_3_) *δ* 5.93 (ddt*, J* = 16.6, 10.4, 5.9 Hz, 1H), 5.35 (dq*, J* = 17.2, 1.6 Hz, 1H), 5.25 (dd*, J* = 10.6, 1.5 Hz, 1H), 4.68 (d*, J* = 5.6 Hz, 2H), 3.81 (d*, J* = 6.5 Hz, 1H), 3.75 (t*, J* = 4.9 Hz, 1H), 3.40 (s, 3H), 2.25 (d*, J* = 5.1 Hz, 1H), 1.76–1.67 (m, 1H), 1.42–1.35 (m, 1H), 1.27–1.22 (m, 9H), 0.89 (d*, J* = 6.8 Hz, 3H), 0.85 (d*, J* = 6.9 Hz, 3H). ^13^C NMR (100 MHz, CDCl_3_) *δ* 171.3, 131.7, 118.9, 82.3, 74.6, 65.6, 58.5, 34.2, 33.6, 31.8, 29.5, 27.0, 22.6, 14.0, 13.4. HRMS (ESI) *m/z*: [M + Na]^+^ calcd for C_15_H_28_O_4_Na^+^ 295.1880, found 295.1878.

*Allyl (2S,3S,4R)-3-(((S)-2-((tert-butoxycarbonyl)amino)pent-4-ynoyl)oxy)-2-methoxy-4-methyldecanoate (****6m****).* The titled compound **6m** was obtained following the general procedure described for **6a**. Flash column chromatography eluent (petroleum ether/ethyl acetate = 9:1 to 5:1) to obtain **6m** (1.21 g, 88%) as a colorless oil. ^1^H NMR (400 MHz, CDCl_3_) *δ* 5.85 (ddt, *J* = 16.5, 9.7, 5.9 Hz, 1H), 5.32–5.23 (m, 2H), 5.18 (d, *J* = 10.3 Hz, 1H), 5.09 (t, *J* = 5.3 Hz, 1H), 4.57 (d, *J* = 5.9 Hz, 2H), 4.33 (dt, *J* = 8.9, 4.8 Hz, 1H), 3.85 (d, *J* = 5.9 Hz, 1H), 3.31 (s, 3H), 2.64 (dd, *J* = 5.1, 2.7 Hz, 2H), 1.98 (d, *J* = 2.6 Hz, 1H), 1.84 (dd, *J* = 9.2, 4.8 Hz, 1H), 1.36 (s, 9H), 1.29–1.11 (m, 9H), 1.09–1.02 (m, 1H), 0.84 (d, *J* = 6.6 Hz, 3H), 0.81–0.74 (m, 3H). ^13^C NMR (100 MHz, CDCl_3_) *δ* 169.8 (2C), 155.0, 131.6, 119.1, 80.3, 79.9, 78.7, 76.7, 71.7, 65.9, 58.5, 51.8, 33.3, 33.1, 31.7, 29.2, 28.2 (3C), 26.8, 22.5, 22.4, 14.2, 14.0. HRMS (ESI) *m/z*: [M + Na]^+^ calcd for C_25_H_41_NO_7_Na^+^ 490.2776, found 490.2775.

*(2S,3S,4R)-2-Methoxy-4-methyl-1-oxo-1-(((R,E)-2,2,14,14-tetramethyl-9,12-dioxo-3,3-diphenyl-4,13-dioxa-10-aza-3-silapentadec-7-en-6-yl)amino)decan-*3-yl *(S)-2-((tert-butoxycarbonyl)amino)pent-4-ynoate (****4m****).* To a solution of compound **6m** (1.21 g, 2.59 mmol, 1.0 equiv) in THF (25 mL) was added *N*-methylaniline (555.1 mg, 5.18 mmol, 2.0 equiv) and Pd(PPh_3_)_4_ (448 mg, 0.388 mmol, 0.15 equiv) under N_2_. After stirred for 3.0 h, the solution was dissolved in ethyl acetate (80 mL) and washed successively with 1% HCl (10 mL), brine (10 mL), dried over Na_2_SO_4_ and concentrated under reduced pressure. The crude product was used directly for the next step without further purification.

To a solution of above acid and **5** (1.88 g, 3.89 mmol, 1.5 equiv) in CH_2_Cl_2_ (25 mL) was added EDCI (596 mg, 3.11 mmol, 1.2 equiv) and HOBt (385 mg, 2.85 mmol, 1.1 equiv) under argon atmosphere at 20 °C. The reaction mixture was stirred for 12 h, and then quenched with 1% HCl (100 mL). The aqueous phase was extracted with ethyl acetate (2 × 80 mL). The combined organic phases were dried (Na_2_SO_4_) and concentrated under reduced pressure. The crude product was purified by column chromatography on silica gel (petroleum ether/ethyl acetate = 9:1 to 5:1) to obtain **4m** (1.49 g, 64% for 2 steps) as a colorless oil. ^1^H NMR (400 MHz, CDCl_3_) *δ* 7.62 (ddd*, J* = 8.3, 5.0, 1.7 Hz, 4H), 7.45–7.33 (m, 6H), 7.21 (d*, J* = 8.3 Hz, 1H), 6.80 (dd*, J* = 15.4, 6.1 Hz, 1H), 6.18 (t*, J* = 5.1 Hz, 1H), 5.95 (dd*, J* = 15.4, 1.5 Hz, 1H), 5.37 (d*, J* = 8.4 Hz, 1H), 5.20 (t*, J* = 5.2 Hz, 1H), 4.63–4.58 (m, 1H), 4.39 (dt*, J* = 9.0, 5.0 Hz, 1H), 3.98 (dd*, J* = 5.1, 2.4 Hz, 2H), 3.84 (d*, J* = 5.2 Hz, 1H), 3.76 (d*, J* = 4.4 Hz, 2H), 3.45 (s, 3H), 2.76–2.61 (m, 2H), 2.00–1.94 (m, 2H), 1.46 (s, 9H), 1.43 (s, 9H), 1.30–1.16 (m, 10H), 1.05 (s, 9H), 0.92 (d*, J* = 6.8 Hz, 3H), 0.83 (t*, J* = 6.6 Hz, 3H). ^13^C NMR (100 MHz, CDCl_3_) *δ* 170.1, 169.1, 169.0, 165.0, 155.2, 141.1, 135.6 (4C), 132.8, 132.7, 130.0 (2C), 127.9 (4C), 124.7, 82.4, 81.8, 80.1, 79.2, 77.4, 71.8, 65.1, 59.2, 52.1, 51.5, 42.2, 33.5, 33.3, 31.8, 29.5, 28.4 (3C), 28.1 (3C), 26.9 (3C), 26.8, 22.7, 22.4, 19.3, 14.6, 14.1. HRMS (ESI) *m/z*: [M + Na]^+^ calcd for C_49_H_73_N_3_O_10_SiNa^+^ 914.4958, found 914.4954.

*(3S,11R,14S,15S,E)-11-(((tert-Butyldiphenylsilyl)oxy)methyl)-14-methoxy-15-((R)-octan-2-yl)-3-(prop-2-yn-1-yl)-1-oxa-4,7,12-triazacyclopentadec-9-ene-2,5,8,13-tetraone (****3m****).* The titled compound **3m** was obtained following the general procedure described for **3a**. Flash column chromatography eluent (dichloromethane/methanol = 50:1 to 9:1) to obtain **3m** (744 mg, 62% for 2 steps) as a white solid. ^1^H NMR (400 MHz, DMSO-*d*_6_) *δ* 8.57 (d, *J* = 9.1 Hz, 1H), 8.46 (d, *J* = 9.0 Hz, 1H), 7.61 (ddt, *J* = 6.0, 4.1, 1.7 Hz, 5H), 7.44 (qdd, *J* = 8.4, 3.3, 1.8 Hz, 6H), 6.74 (dd, *J* = 15.1, 3.0 Hz, 1H), 5.94 (dd, *J* = 15.1, 2.3 Hz, 1H), 5.20 (dd, *J* = 9.7, 1.8 Hz, 1H), 4.73 (ddt, *J* = 9.4, 6.5, 3.7 Hz, 1H), 4.55 (dt, *J* = 9.2, 6.6 Hz, 1H), 4.01 (dd, *J* = 17.6, 5.8 Hz, 1H), 3.92 (d, *J* = 9.7 Hz, 1H), 3.63 (d, *J* = 6.2 Hz, 2H), 3.56 (dd, *J* = 17.6, 7.8 Hz, 1H), 3.23 (s, 3H), 2.99 (t, *J* = 2.6 Hz, 1H), 2.65–2.52 (m, 2H), 1.87 (q, *J* = 6.8 Hz, 1H), 1.26 (d, *J* = 22.4 Hz, 10H), 0.99 (s, 9H), 0.96 (d, *J* = 7.0 Hz, 3H), 0.86 (t, *J* = 6.6 Hz, 3H). ^13^C NMR (100 MHz, DMSO-*d*_6_) *δ* 168.6, 168.4, 167.8, 166.5, 141.5, 135.1 (4C), 132.7, 132.6, 130.0 (2C), 127.9 (4C), 119.5, 79.5, 78.4, 74.1, 74.0, 65.5, 56.7, 51.4, 51.2, 44.1, 33.1, 32.9, 31.3, 28.9, 26.8, 26.6 (3C), 23.1, 22.1, 18.8, 14.0, 13.7. HRMS (ESI) *m/z*: [M + H]^+^ calcd for C_40_H_56_N_3_O_7_Si^+^ 715.3883, found 715.3879.

*(3S,14S,15S,E)-14-Methoxy-11-methylene-15-((R)-octan-2-yl)-3-(prop-2-yn-1-yl)-1-oxa-4,7,12-triazacyclopentadec-9-ene-2,5,8,13-tetraone (****1m****).* The titled compound **1m** was obtained following the general procedure described for **1a**. The crude product was purified by column chromatography on silica gel (dichloromethane/methanol = 100: 1 to 100: 6), *t-*BuOH (3 mL) was added to the eluent and concentrated to remove CH_2_Cl_2_ and MeOH. The residual solution was lyophilized to give **1m** (132 mg, 60% for 2 steps) as a white solid. ^1^H NMR (400 MHz, DMSO-*d*_6_) *δ* 9.13 (s, 1H), 8.56 (d, *J* = 9.1 Hz, 1H), 7.70 (dd, *J* = 7.6, 5.1 Hz, 1H), 6.89 (d, *J* = 15.2 Hz, 1H), 6.14 (d, *J* = 15.2 Hz, 1H), 5.60 (s, 1H), 5.47 (s, 1H), 5.13 (dd, *J* = 8.2, 3.4 Hz, 1H), 4.61 (dt, *J* = 8.7, 6.5 Hz, 1H), 4.09–3.98 (m, 2H), 3.56 (dd, *J* = 17.2, 7.8 Hz, 1H), 3.30 (s, 3H), 2.95 (d, *J* = 2.4 Hz, 1H), 2.59 (m, *J* = 6.6, 2.9 Hz, 2H), 1.92 (m, 1H), 1.40–1.09 (m, 10H), 0.94 (d, *J* = 7.0 Hz, 3H), 0.86 (t, *J* = 6.6 Hz, 3H). ^13^C NMR (100 MHz, DMSO-*d*_6_) *δ* 168.8, 168.5, 168.1, 167.1, 138.3, 137.3, 119.0, 116.3, 79.6, 79.1, 75.2, 73.8, 57.0, 51.1, 44.3, 33.1, 32.6, 31.2, 28.8, 26.6, 23.0, 22.1, 14.0, 13.9. HRMS (ESI) *m/z*: [M + Na]^+^ calcd for C_24_H_35_N_3_O_6_Na^+^ 484.2419, found 484.2421.

*N-(2-(2-(2-(2-(4-(((3S,14S,15S,E)-14-Methoxy-11-methylene-15-((R)-octan-2-yl)-2,5,8,13-tetraoxo-1-oxa-4,7,12-triazacyclo-pentadec-9-en-3-yl)methyl)-1H-1,2,3-triazol-1-yl)ethoxy)ethoxy)ethoxy)ethyl)-5-((3aS,4S,6aR)-2-oxohexahydro-1H-thieno[3,4-d]imidazole-4-yl)pentanamide (****15****).* To a solution of compound **1m** (17.8 mg, 0.039 mmol, 1.0 equiv) in MeCN: MeOH = 2: 1 (3.0 mL in total) were added commercially available **14** (20.6 mg, 0.046 mmol, 1.2 equiv) and Cu_2_O-NP (4.0 mg; for the synthesis, see Adv Synth Catal 2010;**352**:1600–4). The reaction mixture was stirred at RT for 24 h. The mixture was filtered through a pad of silica gel and the filtrate was concentrated under reduced pressure to obtain a residue. The residue was purified by silica gel chromatography (dichloromethane/methanol = 50:1 to 9:1) to obtain **15** (29 mg, 84%) as a white powder. ^1^H NMR (400 MHz, DMSO-*d*_6_) *δ* 9.25 (s, 1H), 8.64 (d, *J* = 9.2 Hz, 1H), 7.84 (s, 2H), 7.63 (t, *J* = 6.4 Hz, 1H), 6.88 (d, *J* = 15.2 Hz, 1H), 6.43 (s, 1H), 6.36 (s, 1H), 6.16 (d, *J* = 15.2 Hz, 1H), 5.60 (s, 1H), 5.46 (s, 1H), 5.14 (dd, *J* = 8.4, 3.3 Hz, 1H), 4.70 (td, *J* = 9.4, 4.7 Hz, 1H), 4.46 (t, *J* = 5.2 Hz, 2H), 4.30 (dd, *J* = 7.8, 5.0 Hz, 1H), 4.16–4.10 (m, 1H), 4.10–4.07 (m, 1H), 3.99 (dd, *J* = 17.3, 5.2 Hz, 1H), 3.76 (t, *J* = 5.2 Hz, 2H), 3.49 (d, *J* = 4.6 Hz, 9H), 3.38 (t, *J* = 6.0 Hz, 2H), 3.30 (s, 3H), 3.17 (q, *J* = 5.8 Hz, 2H), 3.08 (dt, *J* = 14.5, 4.4 Hz, 2H), 2.98 (dd, *J* = 14.7, 9.6 Hz, 1H), 2.81 (dd, *J* = 12.4, 5.0 Hz, 1H), 2.57 (d, *J* = 12.5 Hz, 1H), 2.06 (t, *J* = 7.4 Hz, 2H), 1.92 (s, 1H), 1.65–1.40 (m, 4H), 1.36–1.18 (m, 12H), 0.92 (d, *J* = 6.7 Hz, 3H), 0.85 (t, *J* = 6.9 Hz, 3H). ^13^C NMR (100 MHz, DMSO-*d*_6_) *δ* 172.1, 169.5, 168.6, 168.2, 167.1, 162.7, 142.1, 138.2, 137.4, 123.3, 119.1, 116.2, 79.0, 74.7, 69.7, 69.6 (2C), 69.5, 69.2, 68.8, 61.0, 59.2, 56.9, 55.4, 52.2, 49.3, 44.2, 40.2, 39.9, 38.4, 35.1, 33.1, 32.7, 31.2, 28.8, 28.2, 28.0, 26.5, 25.3, 22.1, 14.0, 13.8. HRMS (ESI) *m/z*: [M + Na]^+^ calcd for C_42_H_67_N_6_O_11_SNa^+^ 928.4573, found 928.4571.

*(3S,14S,15S,Z)-3-(tert-Butyl)-14-methoxy-11-((4-methylpiperazin-1-yl)methyl)-15-((R)-octan-2-yl)-1-oxa-4,7,12-triazacyclopentadec-10-ene-2,5,8,13-tetraone* (16)*.* To a solution of compound **2j** (825.0 mg, 1.66 mmol, 1.0 equiv) in THF (6.2 mL), then triethylamine (336 mg, 3.32 mmol, 2.0 equiv) and ethanesulfonyl chloride (320 mg, 2.49 mmol, 1.5 equiv) were added at 0 °C. After stirred for 2 h, the reaction solution was quenched by addition of water (0.1 mL), dried over Na_2_SO_4_, filtered and concentrated under reduced pressure. The resulting crude product was dissolved in THF (6.2 mL). To the resulting solution was added *N*-methyl piperazine (1.84 mL, 16.6 mmol, 10 equiv) at 20 °C. After stirred for 4 h, the solution was dissolved in ethyl acetate (80 mL) and washed successively with saturated aqueous NaHCO_3_ (30 mL) and brine (30 mL), dried over Na_2_SO_4_ and concentrated under reduced pressure carefully. The crude product was purified by column chromatography on silica gel dichloromethane/methanol = 100: 1 to 8: 1), *t*-BuOH (10 mL) was added to the eluent and concentrated to remove CH_2_Cl_2_ and MeOH. The residual solution was lyophilized to give **16** (452 mg, 47% for 2 steps) as a white solid. ^1^H NMR (400 MHz, CDCl_3_) *δ* 8.29 (s, 1H), 7.36 (d*, J* = 9.8 Hz, 1H), 6.46 (s, 1H), 5.37 (t*, J* = 8.0 Hz, 1H), 5.11 (t*, J* = 6.2 Hz, 1H), 4.52 (dd*, J* = 13.6, 9.2 Hz, 2H), 3.86 (d*, J* = 5.7 Hz, 1H), 3.52 (dd*, J* = 17.3, 7.9 Hz, 1H), 3.46 (s, 3H), 3.38 (dd*, J* = 15.4, 4.3 Hz, 1H), 3.13–3.00 (m, 2H), 2.94 (dd*, J* = 17.2, 8.2 Hz, 1H), 2.47 (s, 8H), 2.30 (s, 3H), 2.18 (p*, J* = 6.6 Hz, 1H), 1.57–1.47 (m, 1H), 1.36–1.20 (m, 9H), 1.02 (s, 9H), 0.94 (d*, J* = 6.8 Hz, 3H), 0.89–0.82 (m, 3H). ^13^C NMR (100 MHz, CDCl_3_) *δ* 171.8, 169.5, 169.2, 167.5, 135.5, 113.4, 80.4, 76.1, 62.9, 61.5, 58.8, 55.3 (2C), 52.7 (2C), 46.0, 44.6, 36.0, 35.5, 33.5, 33.3, 31.9, 29.6, 26.9 (3C), 26.7, 22.7, 15.6, 14.2. HRMS (ESI) *m/z*: [M + H]^+^ calcd for C_30_H_54_N_5_O_6_^+^ 580.4069, found 580.4071.

### 4.2 Cell culture

MDA-MB-231, Huh-7, U118, A549, 4T1 and 3T3 cells were cultured in DMEM medium supplemented with 10% fetal bovine serum and penicillin (100 μg/mL), streptomycin (100 μg/mL) at 37 °C including 5% CO_2_.

### 4.3 MTT assay

Cancer cells including MDA-MB-231, Huh-7, U118 and A549 were seeded into 96 well plate individually. After cell attachment, boholamide A derivatives were added with different concentrations and incubated for 72 h. Then 20 μL MTT solution (5 mg/mL) was added and incubated with additional 4 h. After that the supernatant was discarded, the precipitate was dissolved with DMSO and the absorbance at 570 nm was measured. The IC_50_ was calculated with GraphPad Prism 5 and repeated for 3 times.

### 4.4 Colony formation assay

MDA-MB-231 cells were seeded into 24 well plate with 200 cells per well. After cell attachment, compounds were added with different concentrations and treated for 7–10 days. The cells were fixed with 4% paraformaldehyde for 20 min, then stained with crystal violet for 30 min. After that the number of clones were counted.

### 4.5 Cell apoptosis assay

MDA-MB-231 cells in logarithmic growth phase were digested, counted and seeded into 24 well plate. Compounds were added with different concentrations and treated for 48 h. Then the cells were collected, washed with PBS buffer and resuspended with binding buffer. After that, cells were stained with 5 μL Annexin Ⅴ-FITC and 5 μL propidium iodide (PI) for 15 min at room temperature in the dark. Then the cells were analyzed by flow cytometry.

### 4.6 Trans-well assay

MDA-MB-231 cells treated with compounds for 48 h were collected and counted. Then 3 × 10^4^ cells were resuspended with serum-free medium and seeded into the upper chambers. The lower layer was supplied with culture containing 20% fetal bovine serum. After 48 h, cells in upper chamber were fixed with 4% paraformaldehyde for 20 min and stained with crystal violet for 30 min. Then cells on the inside bottom of the upper chamber were erased with a cotton swab, and the migration cells on the outside bottom were calculated under a microscope.

### 4.7 Transfection

The CDS regions of eEF1A1 and FoxO1 genes were cloned into the pCMV plasmid respectively. Then the cells were prepared and transfected with corresponding plasmid using Lipofectamine 3000 Transfection Reagent.

### 4.8 Western blot assay

MDA-MB-231 cells were collected and lysed with RIPA buffer. Proteins in supernatant were collected, boiled with loading buffer, separated by SDS-PAGE and transferred onto PVDF membranes. After blocking with 5% nonfat milk, the membranes were incubated with primary antibodies (1:1000) and subsequently secondary antibodies (1:5000). Then the membranes were washed with PBST buffer and detected with electrochemiluminescence (ECL) reagent.

### 4.9 Pull-down assay

MDA-MB-231 cell lysates were collected and incubated with probe **15** for 2 h at 4 °C. The protein was precipitated with methanol for 40 min at −20 °C. After centrifugation, then the precipitated proteins were collected, dissolved with PBS containing 0.1% SDS buffer and incubated with streptavidin beads for overnight at 4 °C. After that the streptavidin beads were washed for five times with PBS buffer, and the bead-bound proteins were eluted, separated by SDS-PAGE, and visualized by silver staining or Western blot assay.

### 4.10 Kinetic determination of eEF1A1 binding

MDA-MB-231 cell lysate was incubated with probe **15** at different concentrations for 2 h. Then the pull-down assay was performed and the precipitated proteins were analyzed by Western blot. The gray value of eEF1A1 protein band was analyzed by Image J. The *K*_i_ value was obtained through nonlinear curve fitting. Then MDA-MB-231 cell lysate was incubated with probe **15** at 10 μmol/L for different times. In the following, the pull-down assay was performed and the precipitated proteins were analyzed by Western blot. The gray value of eEF1A1 protein band was analyzed by Image J. And *K*_obs_ in binding eEF1A1 was calculated by Eq. (1):

(1) [eEF1A1]*t* = [ eEF1A1]0 × *E*‒ *K*_obs_ × *t*

where [eEF1A1]0 means the total concentration of input-eEF1A1, and [eEF1A1]*t* means the concentration of eEF1A1 at time *t*.

### 4.11 Molecular docking assay

The crystal structure of eEF1A1 (PDB ID: 6ZMO) was retrieved from the RCSB Protein Data Bank. The three-dimensional structure of compound **1j** was generated using ChemDraw software and subsequently energy-minimized to adopt its lowest-energy conformation. The optimal binding pose of the ligand was selected based on the highest docking score.

### 4.12 Protein purification

pET28a-eEF1A1 and pET28a-eEF1A1 mutation plasmids were transformed into *E. coli* strain BL21 respectively. Then *E. coli* strain BL21 were cultured with LB medium supplement kanamycin at 37 °C. After the OD value at 600 nm reached 0.5–0.8, IPTG (0.5 mmol/L) was added and cultured at 16 °C for another 16–20 h. Then the bacterial cells were collected after centrifuged at 5000 rpm for 30 min, lysed with lysis buffer and cracked with ultrasonic crusher. Then the protein supernatant was obtained, Ni-beads column was used to bind His tagged eEF1A1 protein. After washed with wash buffer, eEF1A1 protein was eluted with elution buffer. In the following the eEF1A1 protein was desalted and quantified with BCA assay.

### 4.13 CESTA assay

MDA-MB-231 cultured in hypoxia was collected and lysed with RIPA buffer. After centrifuged at 12,000×*g* for 15 min, the cell lysate was obtained and incubated with **1j** for 1 h at room temperature. Then, the lysates were heated individually for 3 min at different temperatures (35 to 70 °C). After cooling for 5 min on ice, the lysates were centrifuged at 12,000×*g* for 10 min and the protein supernatant was analyzed by Western blot assay.

### 4.14 Binding site assay

Recombinant human eEF1A1 protein (50 μg) was incubated with compound **1j** at room temperature for 16 h. Then the mixture was separated with SDS-PAGE and the specific eEF1A1 band was excised and digested with trypsin. After that, the binding site was analyzed using Triple-quad Ion-trap and Orbitrap fusion (Thermo Fisher Scientific, USA).

### 4.15 Co-immunoprecipitation assay

MDA-MB-231 cells transfected with flag-labeled pCMV-eEF1A1 or pCMV-FoxO1 plasmid were collected and lysed with RIPA buffer. After centrifuged at 12,000×*g* for 15 min at 4 °C, the cell lysate was obtained and incubated with IgG or Flag antibody at 4 °C overnight. Then 20 μL protein A + G Agarose was added and incubated at 4 °C overnight. After washing with 1 × PBS buffer for 5 times, the Agarose was collected and resuspended with 1 × loading buffer. After boiled for 10 min at 100 °C, the samples were developed to Western blot assay.

### 4.16 Nuclear and cytoplasmic protein extraction

MDA-MB-231 cells were seeded into 6-well plate and incubated with compound **1j** for 48 h. Then the cells were collected and the protein were extracted by nuclear and cytoplasmic protein extraction kit according to the manufacturer’s protocol.

### 4.17 Toxicity assay

Balb/c mice were randomly divided into two groups with 4 mouse each group. Then compound **16** was administrated intraperitoneally at dose of 20, 40 and 80 mg/kg respectively. Then the body weight of mice was recorded every day. After 72 h, the organ weights of mice were measured and the ALT, AST, CR level in plasma were examined using ELISA kit.

### 4.18 Animal model assay

The protocol and experiment were approved by Ethics of Animal Experiments Committee of Nankai University of China. Bal-b/c mice were purchased from Charles River and feed in a standard SPF mouse room. 4T1 cells (2 × 10^5^ cells) were injected into breast fat pad on the right side. After the tumor formation, the mice were randomly divided into 3 groups and intraperitoneally administered with compound **16** every two days. Body weight and tumor volume were monitored throughout the experiment.

### 4.19 Immunohistochemical assay

The tumor tissues were fixed with 4% paraformaldehyde. After gradient dehydration, the tissues were embedded in paraffin and subsequently sectioned into 4 μm-thick slices. Then the slides were deparaffinized, hydrated and incubated with primary antibodies including JAK2, STAT3 and pSTAT3 at 4 °C overnight. After washed with PBST for 3 times, the slides were incubated with corresponding secondary antibody for 1 h at room temperature. In the following, the slides were developed by DAB, counterstained with hematoxylin and sealed with neutral balsam.

### 4.20 H&E staining

The tissues were fixed with 4% paraformaldehyde. After gradient dehydration, the tissues were embedded in paraffin and subsequently sectioned into 4 μm-thick slices. Then the slides were deparaffinized, hydrated and dyed with hematoxylin. After that the slides were immersed in 1% hydrochloric acid alcohol, dyed with eosin, dehydrated and sealed with neutral balsam. Then the slides were observed under microscope.

### 4.21 Statistical analysis

All the data were analyzed using GraphPad Prism. The differences between groups were analyzed using Student *t* test. Data are represented as the mean ± SD. The test results were considered statistically significant at ∗*P* < 0.05, ∗∗*P* < 0.01, ∗∗∗*P* < 0.005, ∗∗∗∗*P* < 0.001.

## Author contributions

Quan Zhang and Yahui Ding designed the research; Guangju Liu, Fengyuan Zhang, Fangzhi Han, Sisi Chen, Junjie Ou, Renfeng Qiao, Yixuan Jiang, Shaojie Miao, Wei Lin, and Yahui Ding performed experiment; Guangju Liu, Fengyuan Zhang, Fangzhi Han, Sisi Chen, Junjie Ou, Yixuan Jiang, Shaojie Miao, Wei Lin, Yahui Ding and Quan Zhang analyzed data; Guangju Liu, Yahui Ding and Quan Zhang wrote the manuscript.

## Conflicts of interest

The authors declare no competing financial interest.

## Appendix A Supporting information

The following is the Supporting Information to this article:
