# Supplementary material for: Identification of boholamide A analogue as a potential hypoxia-selective anti-triple-negative breast cancer agent by targeting eEF1A1
Source: Acta Pharm Sin B. 2026 Feb 11;16(5):3128–56. doi: 10.1016/j.apsb.2026.02.009 (PMC13198255; doi:10.1016/j.apsb.2026.02.009)
Supplement: Multimedia component 1 [file mmc1.pdf]

# Identification of boholamide A analogue as a potential hypoxia-selective anti-triple-negative breast cancer agent by targeting eEF1A1

# Supporting Information

## Table of Contents

**1. The toxicity evaluation of boholamide A analogue**……………………………………S2

**2. Synthesis of Compounds 10** ……………………………………………………………S4

**3. NMR Spectra** ………………………………………………………….…………....……S6

**4. HPLC Spectra** ……………………………………………………….…………....……S77

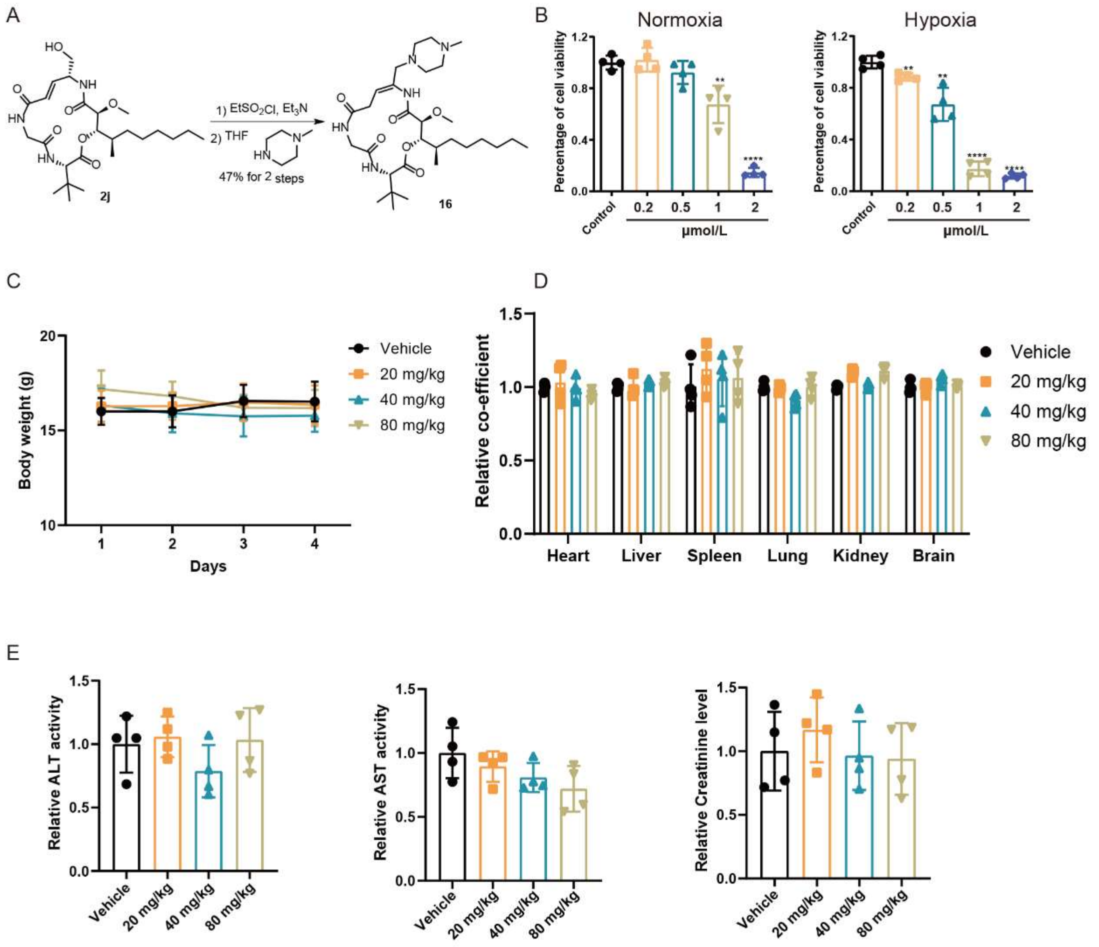


Figure S1 The toxicity evaluation of boholamide A analogue. (A) The synthesis route of prodrug **16**. (B) The antiproliferative activity against MDA-MB-231 cells of prodrug **16** under normoxia and hypoxia. (C-D) The body weight and organ coefficients changes after intraperitoneal administration of compound **16** at different doses. (E) The level of AST, ALT and CR levels after intraperitoneal administration of compound **16** at different doses. **P*<0.05, ** *P*<0.01, *** *P*<0.005, **** *P*<0.001. Data presented as means ± SD (*n* = 4).

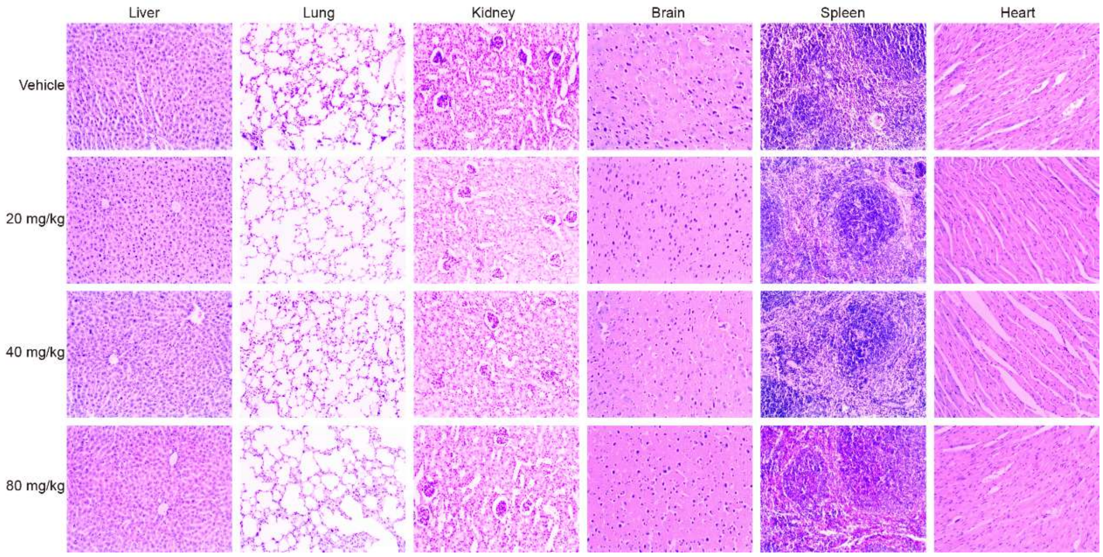


Figure S2 Histopathological analyses of major organs (including liver, kidney, heart, spleen, brain and lung) using hematoxylin and eosin (H&E) staining.

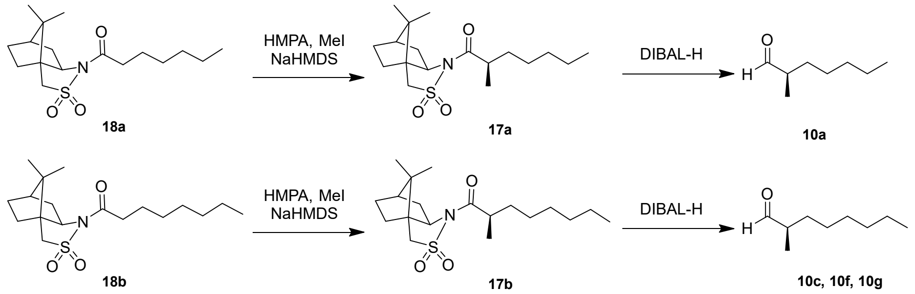


*(2R)-1-((6R,7aR)-8,8-Dimethyl-2,2-dioxidotetrahydro-3H-3a,6-methanobenzo[c]isothiazol-1(4H)-yl)-2-methylheptan-1-one*（***17a***）. To a solution of compound **18a** (10 g, 29.3 mmol, 1.0 equiv) and HMPA (5.8 g, 32.3 mmol, 1.1 equiv) in THF (150 mL) was added NaHMDS (32.3 mL, 32.2 mmol, 1.1 equiv, 1.0 M in THF) at – 78 ºC under argon atmosphere. After the reaction was stirred for 1h, MeI (5.0 g, 35.1 mmol, 1.2 equiv) is added slowly. The reaction was stirred at –78°C for 3 h before saturated aqueous $NH_4Cl$ (50 mL) was added. The aqueous phase was extracted with ethyl acetate (2 × 50 mL). The combined organic phase was washed with brine (2 × 50mL), dried over $Na_2SO_4$, filtered and concentrated under reduced pressure. The crude product was purified by column chromatography on silica gel (petroleum ether / ethyl acetate =4:1) to obtain **17a** (8.5 g, 85 %) as a colorless oily liquid. $^1$H NMR (400 MHz, $CDCl_3$) $\delta$ 3.88 (d, $J$ = 6.9 Hz, 1H), 3.46 (q, $J$ = 9.6, 5.3 Hz, 2H), 3.03 (t, $J$ = 7.6 Hz, 1H), 2.04 (d, $J$ = 6.3 Hz, 2H), 1.87 (d, $J$ = 16.2 Hz, 3H), 1.78 – 1.73 (m, 1H), 1.44 – 1.22 (m, 9H), 1.21 – 1.11 (m, 6H), 0.95 (d, $J$ = 5.5 Hz, 3H), 0.84 (d, $J$ = 7.0 Hz, 3H). $^{13}$C NMR (100 MHz, $CDCl_3$) $\delta$ 176.50, 65.18, 53.29, 48.35, 47.82, 44.70, 40.47, 38.56, 32.90, 32.71, 31.95, 27.05, 26.55, 22.59, 20.93, 19.99, 19.16, 14.17. HRMS (ESI) *m/z*: $[M + H]^+$ Calcd for $C_{18}H_{32}NO_3S^+$ 342.2098; Found 342.2099.

*(R)-2-methylheptanal*（***10a***）.To a solution of compound **17a** (5 g, 14.06 mmol, 1.0 equiv) in THF (70 mL) was added DIBAL-H（15.47 mL, 15.47 mmol, 1.1 equiv, 1.0 M in THF) at – 78 ºC under argon atmosphere. After stirring for 1 h, $H_2O$ (15 mL), 15% NaOH (30 mL), and $H_2O$ (15 mL) were successively added to quench the reaction. Dried over $MgSO_4$ and stirring continued for 20 min. It was filtered, washed with $CH_2Cl_2$, and concentrated at 20°C under reduced pressure. The crude product was

purified by column chromatography on silica gel (hexane) to obtain colorless liquid **10a,** which was directly used in the next reaction.

*(2R)-1-((6R,7aR)-8,8-dimethyl-2,2-dioxidotetrahydro-3H-3a,6-methanobenzo[c]isothiazol-1(4H)-yl)-2-methyloctan-1-one* （ ***17b*** ） . The titled compound **17b** was obtained following the general procedure described for **17a**. The residue was purified by column chromatography on silica gel (petroleum ether / ethyl acetate =4:1) to obtain compound **17b** (23.7 g, 84 %) as a colorless oily liquid. $^{1}$H NMR (400 MHz, $CDCl_3$) $\delta$ 3.89 (t, $J$ = 6.2 Hz, 1H), 3.46 (q, $J$ = 13.7 Hz, 2H), 3.04 (h, $J$ = 7.0 Hz, 1H), 2.05 (d, $J$ = 6.4 Hz, 2H), 1.88 (dd, $J$ = 16.6, 6.8 Hz, 3H), 1.83 – 1.76 (m, 1H), 1.60 (s, 1H), 1.45 – 1.25 (m, 10H), 1.19 (d, $J$ = 6.9 Hz, 3H), 1.15 (s, 3H), 0.96 (s, 3H), 0.85 (t, $J$ = 6.4 Hz, 3H). $^{13}$C NMR (100 MHz, $CDCl_3$) $\delta$ 176.56, 65.24, 53.35, 48.39, 47.87, 44.76, 40.52, 38.61, 32.96, 32.82, 31.82, 29.47, 27.38, 26.60, 22.75, 20.97, 20.03, 19.19, 14.24. HRMS (ESI) *m/z*: $[M + H]^+$ Calcd for $C_{22}H_{35}N_3O_6Na^+$ 356.2245; Found 356.2243.

*(R)-2-methyloctanal* （***10c, 10f, 10g***）. The titled compound **10c (10f, 10g)** was obtained following the general procedure described for **10a**. The crude product was purified by column chromatography on silica gel (hexane) to obtain colorless liquid **10a,** which was directly used in the next reaction.

## 3. NMR Spectra

$^{1}$H NMR spectrum of **17a**

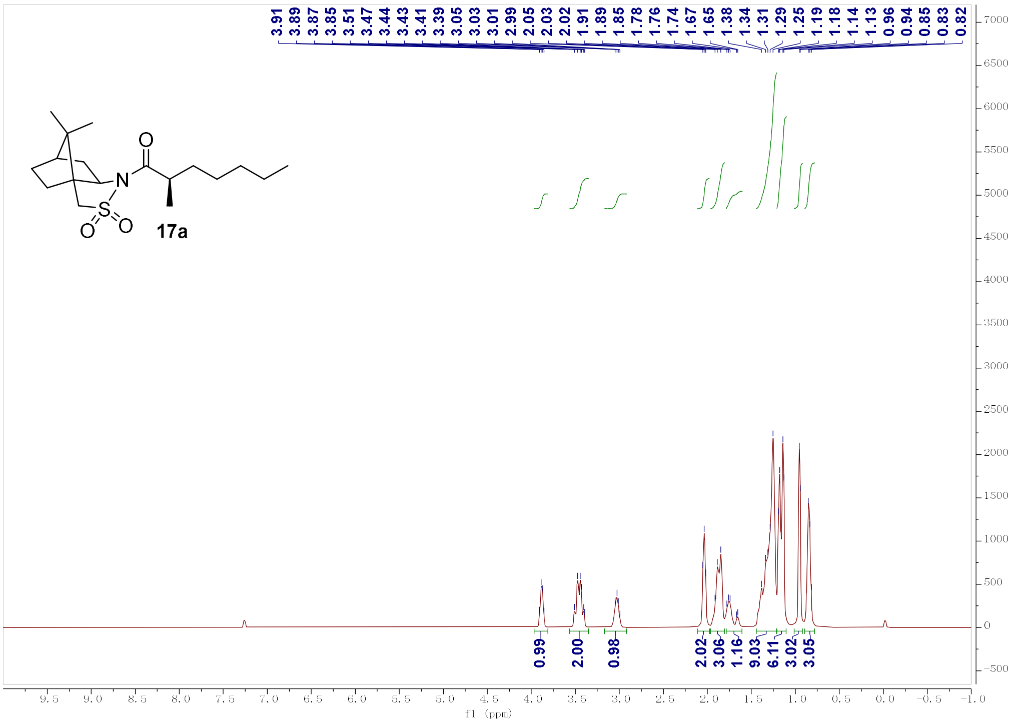


$^{13}$C NMR spectrum of **17a**

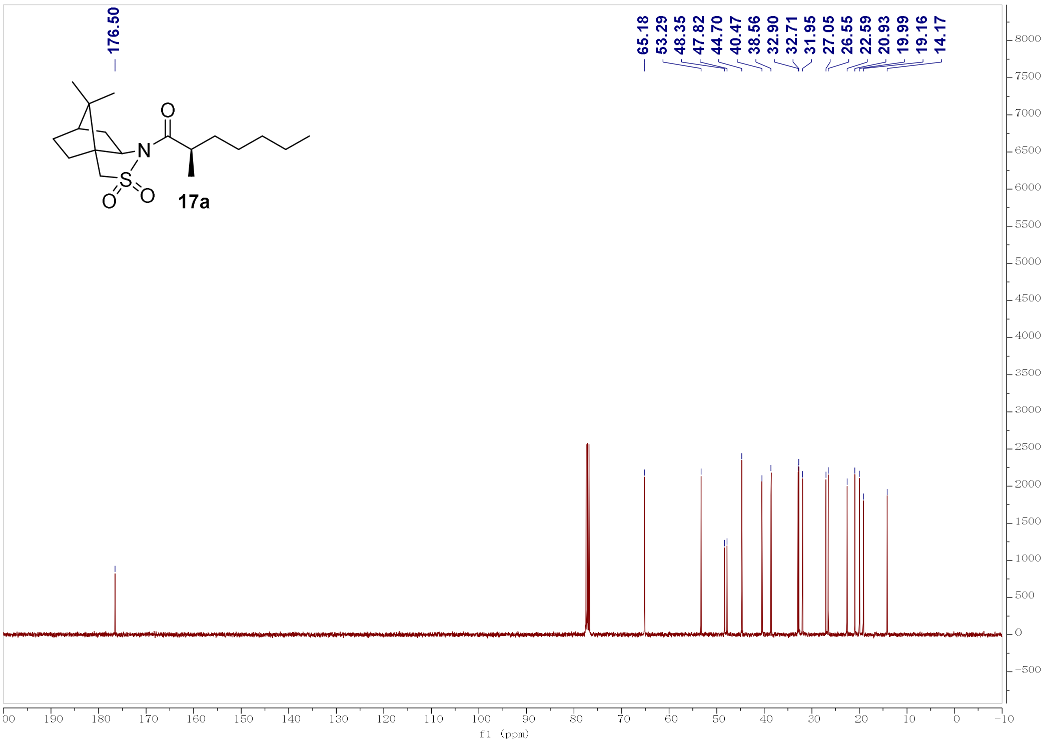

$^{1}$H NMR spectrum of **17b**

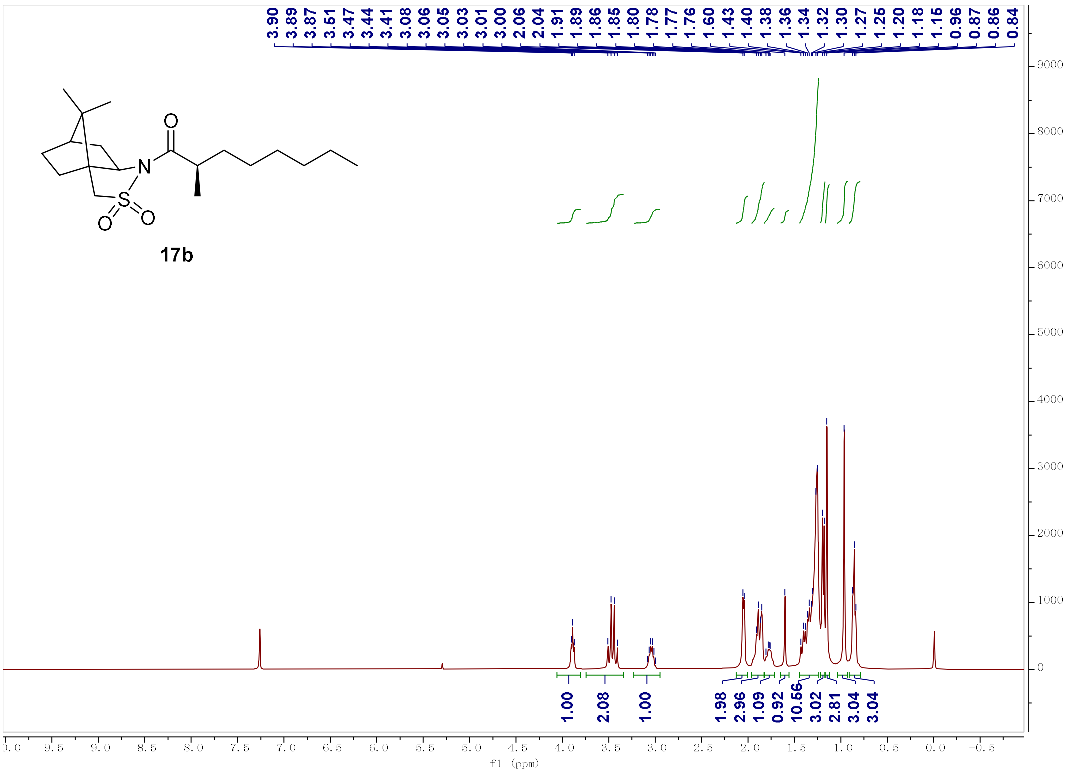

$^{13}$C NMR spectrum of **17b**

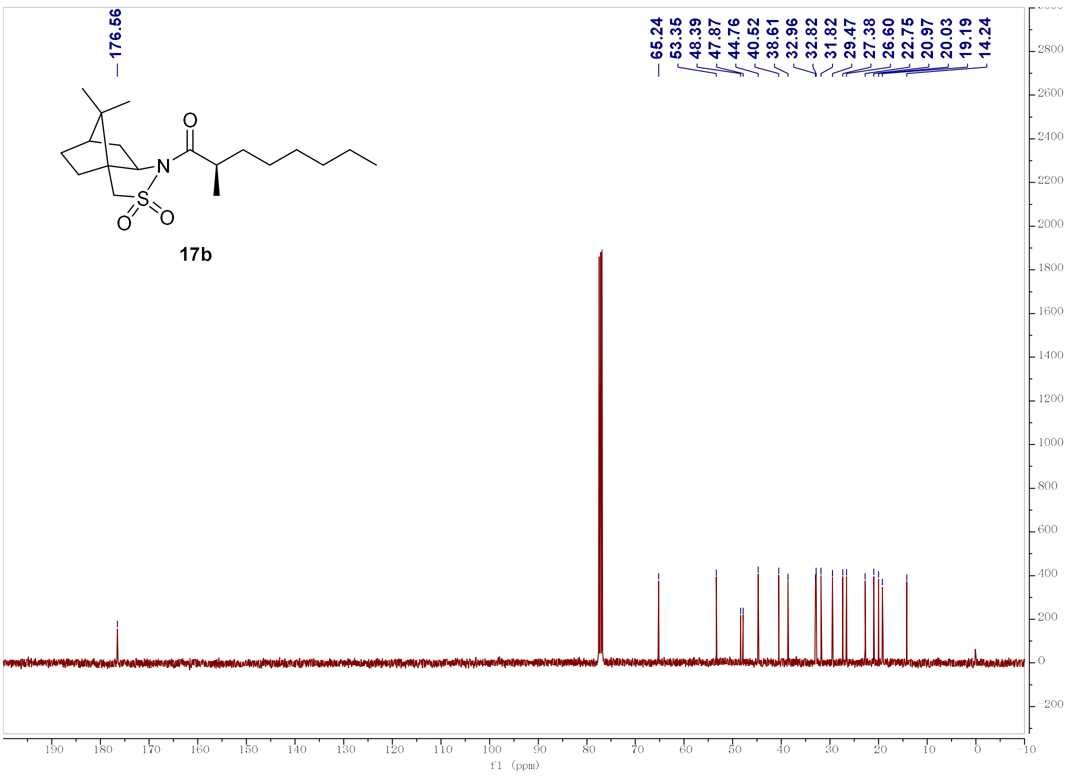

$^{1}$H NMR spectrum of **9a**

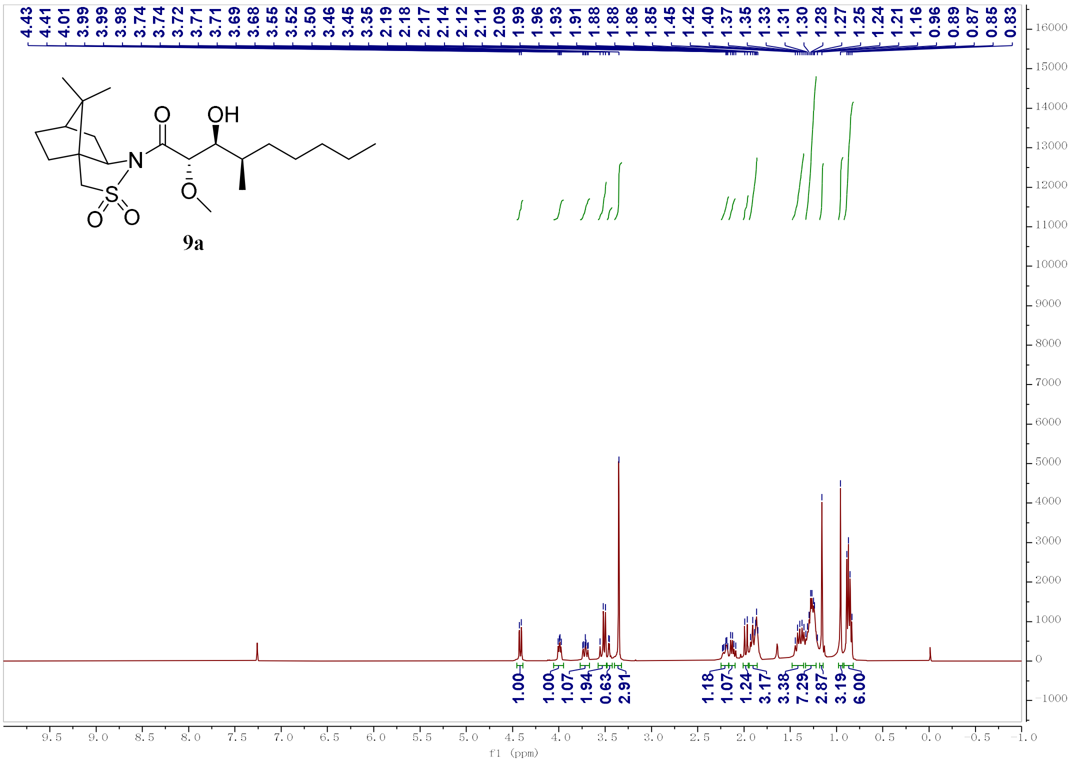


$^{13}$C NMR spectrum of **9a**

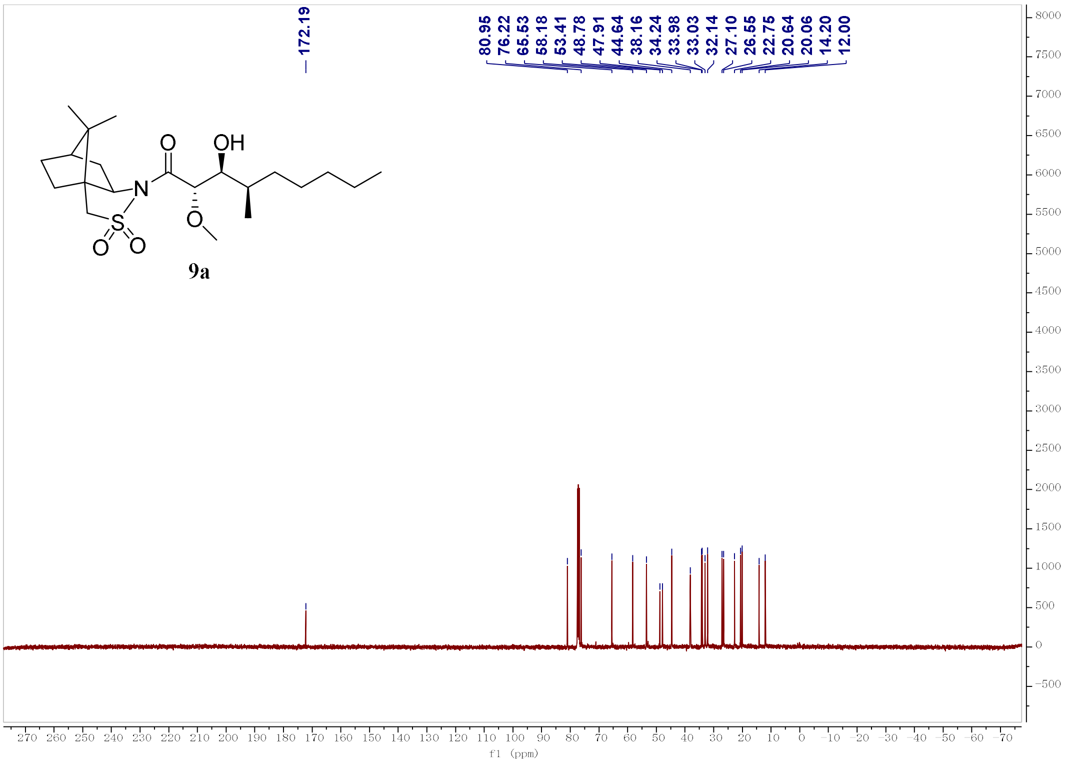

$^{1}$H NMR spectrum of **9b**

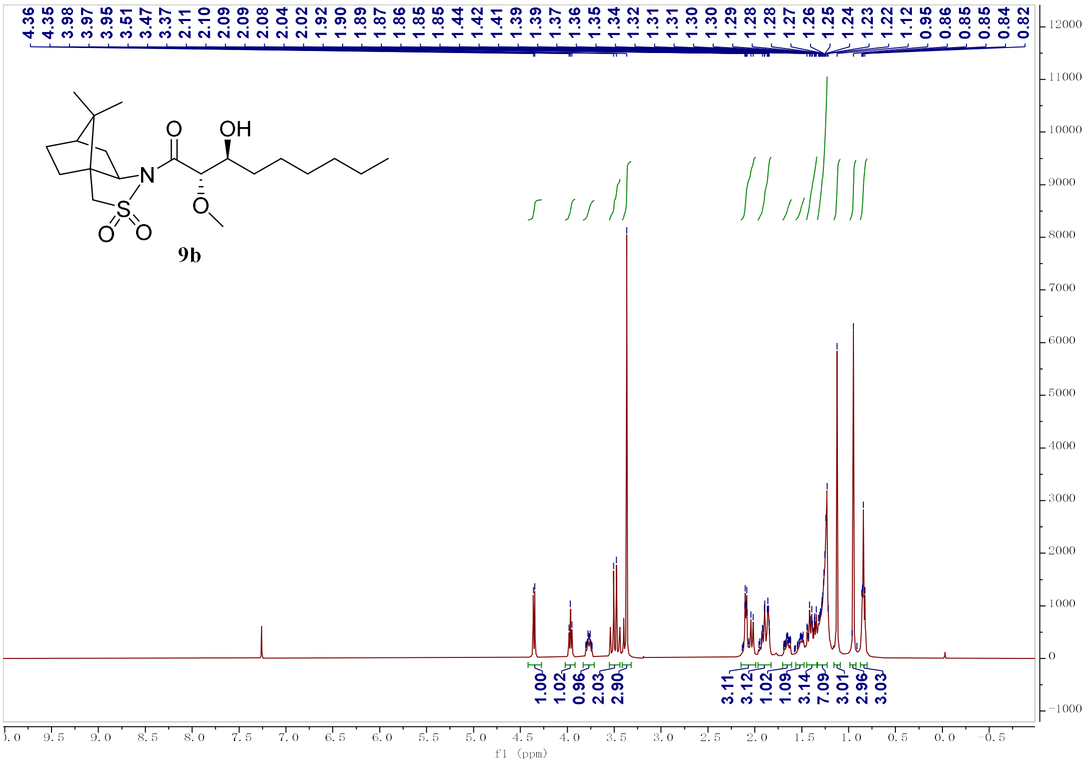

$^{13}$C NMR spectrum of **9b**

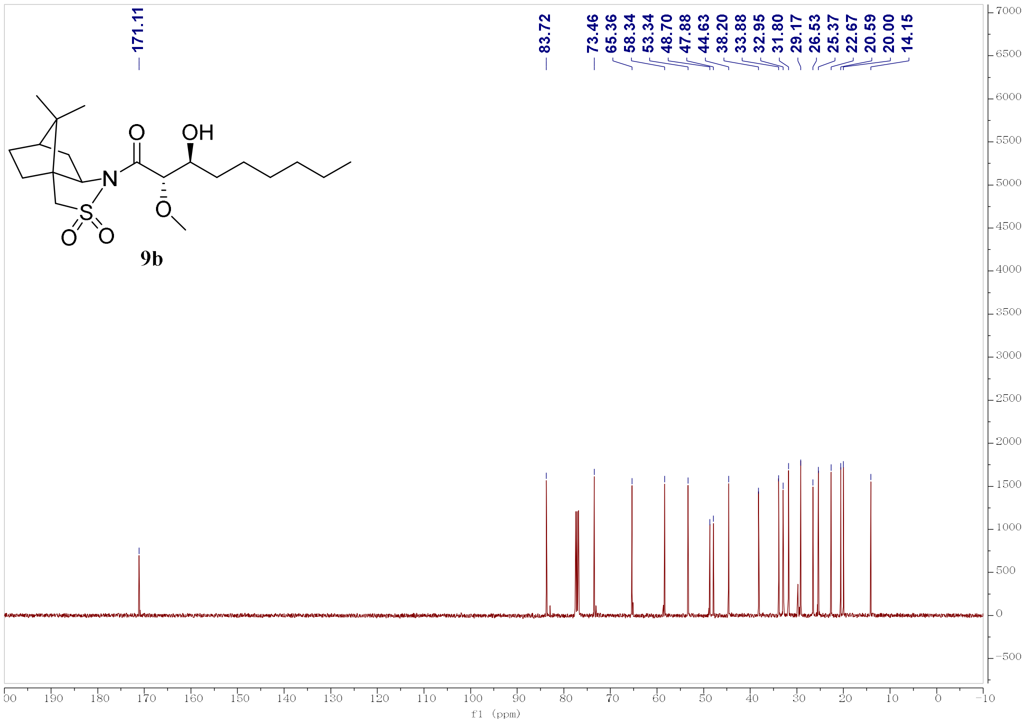

$^{1}$H NMR spectrum of **9c**

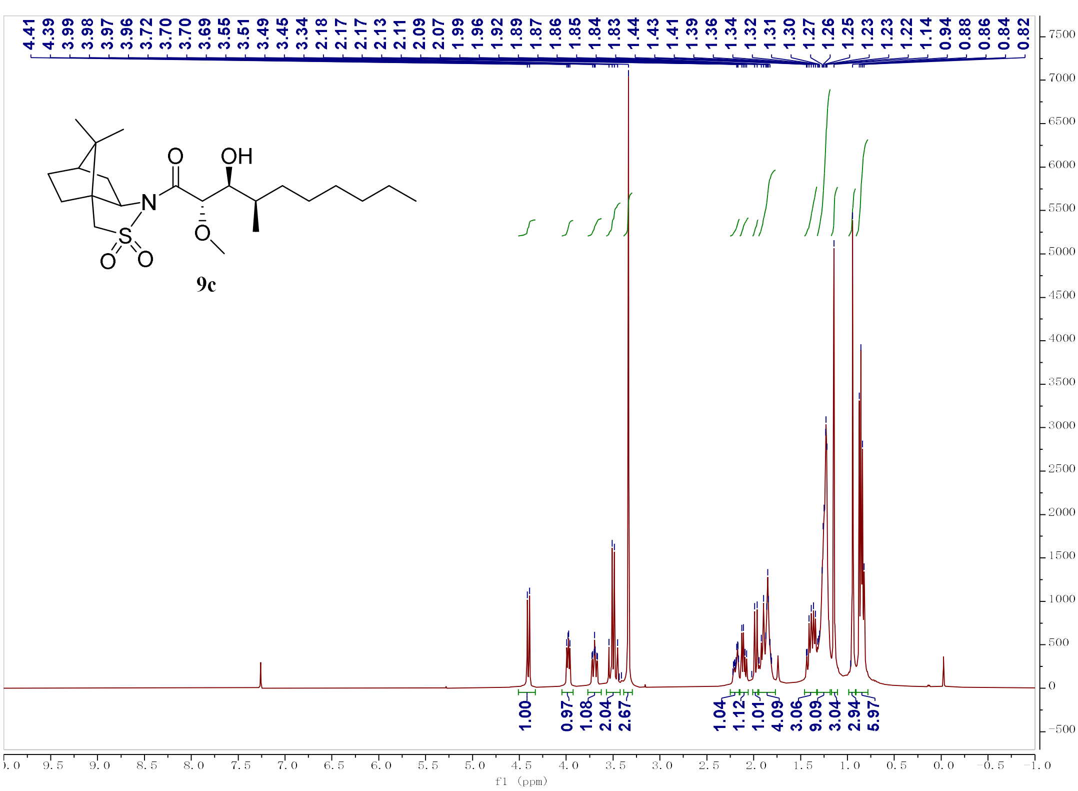

$^{13}$C NMR spectrum of **9c**

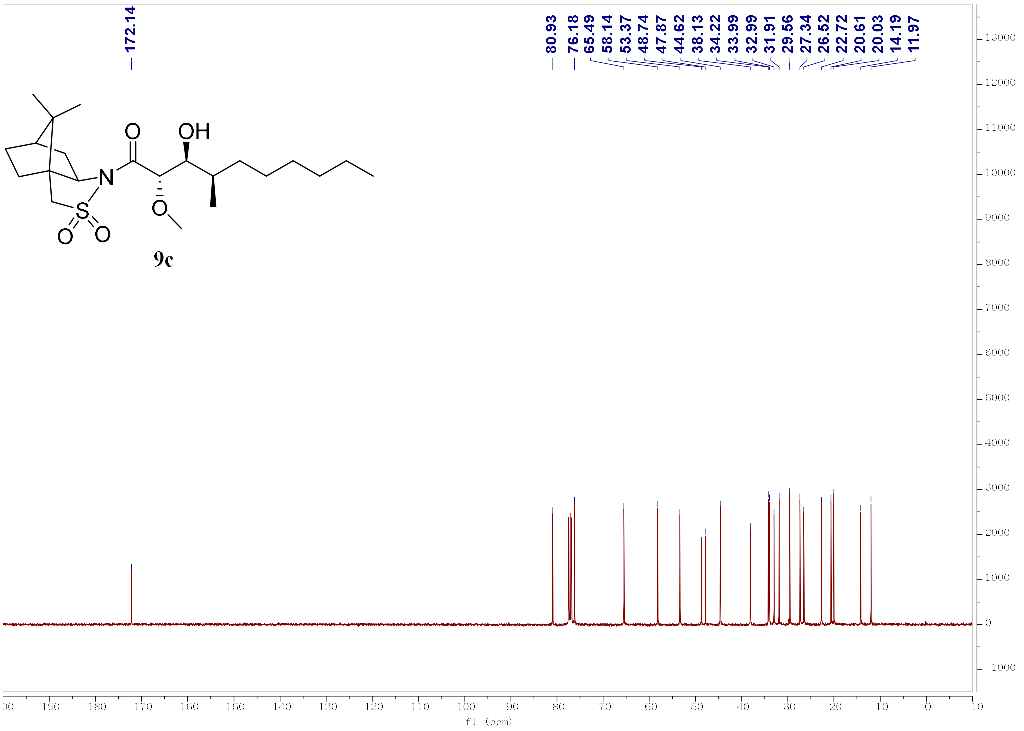

$^{1}$H NMR spectrum of **9d[1]**

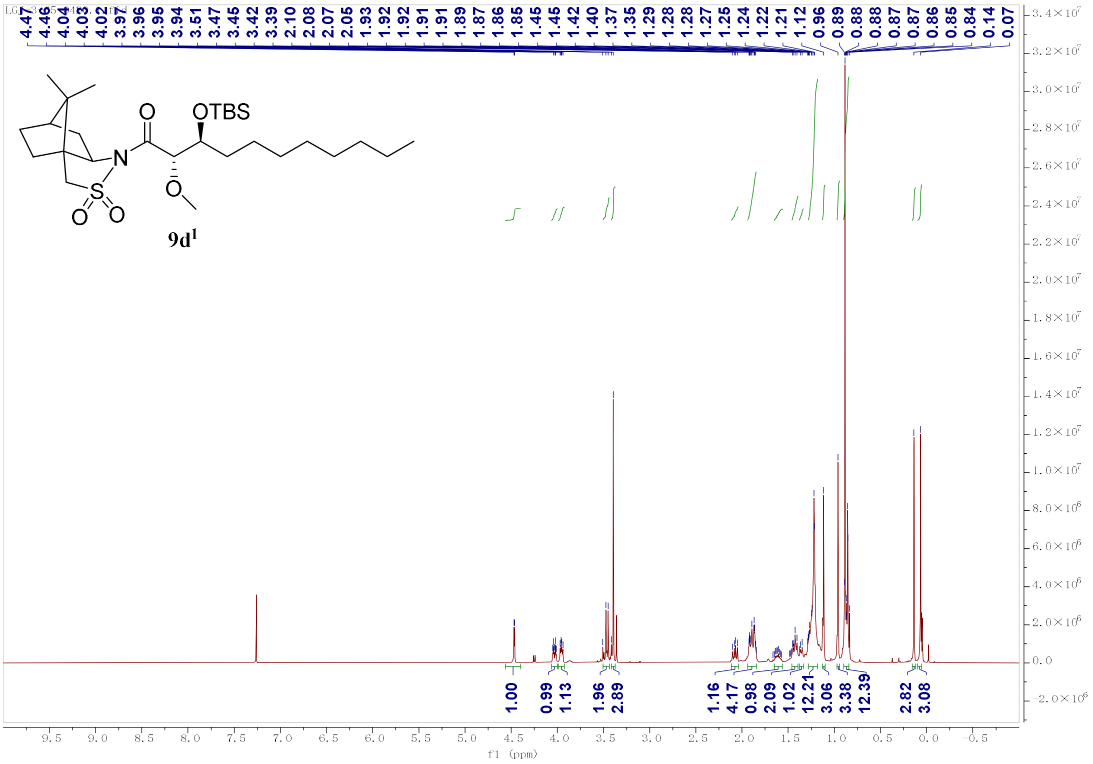

$^{13}$C NMR spectrum of **9d[1]**

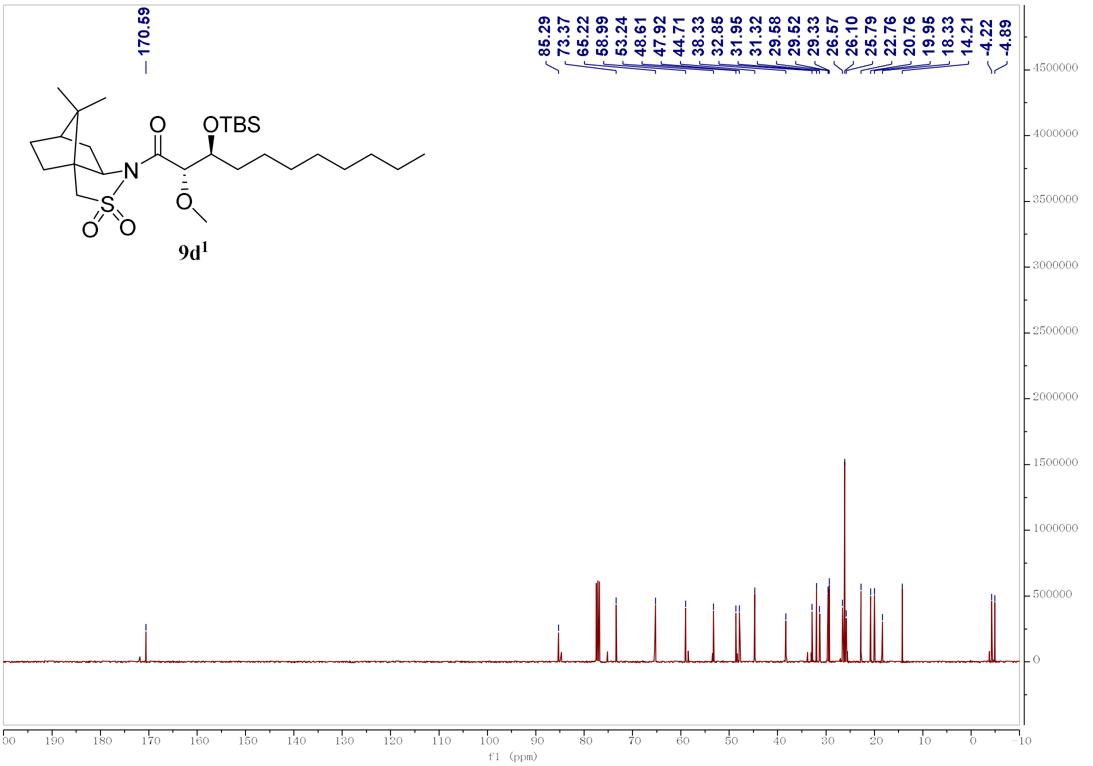

## $^1$H NMR spectrum of **9e**

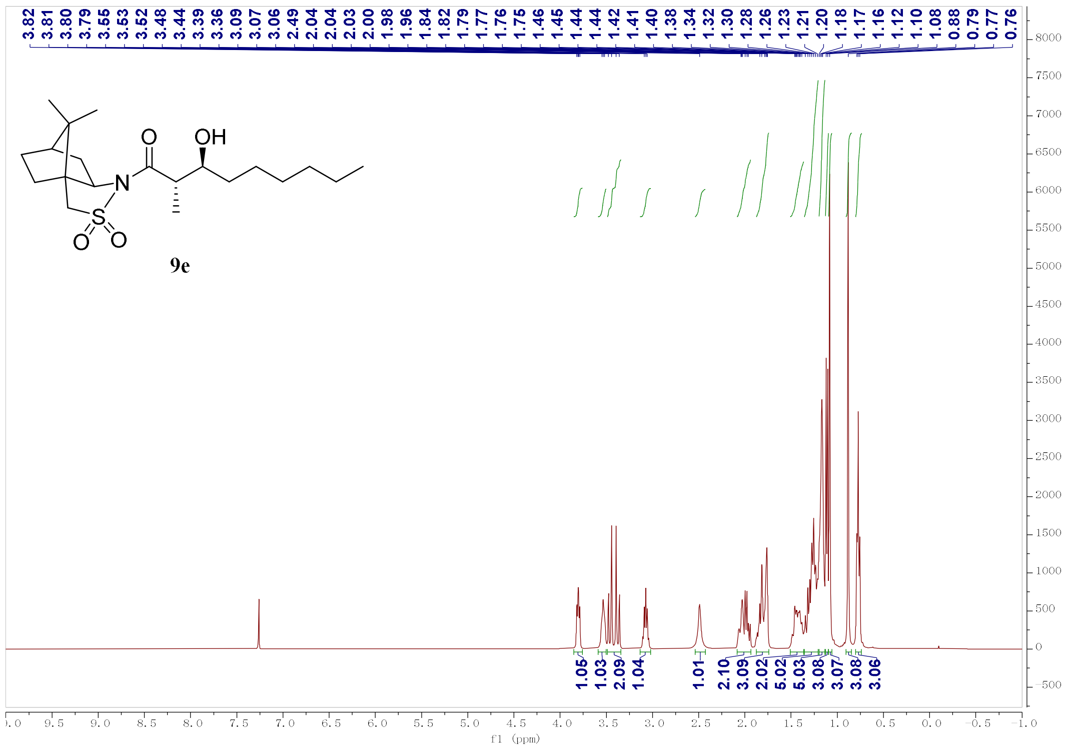

## $^{13}$C NMR spectrum of **9e**

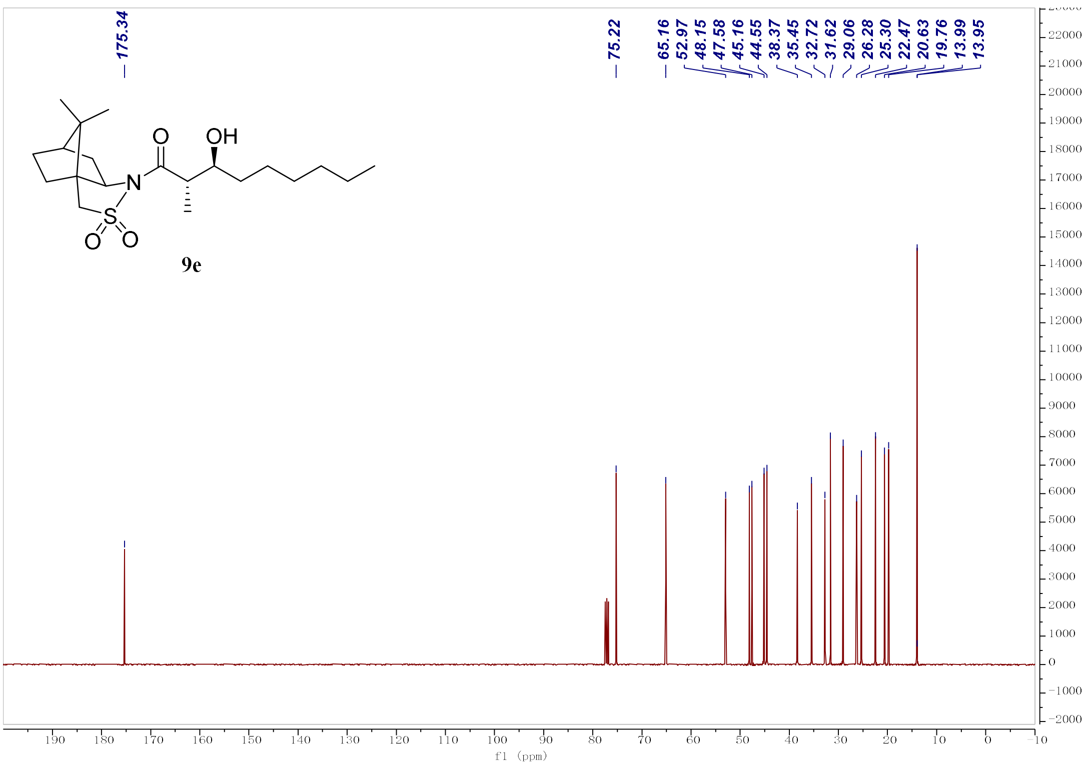

## $^{1}$H NMR spectrum of **9f[1]**

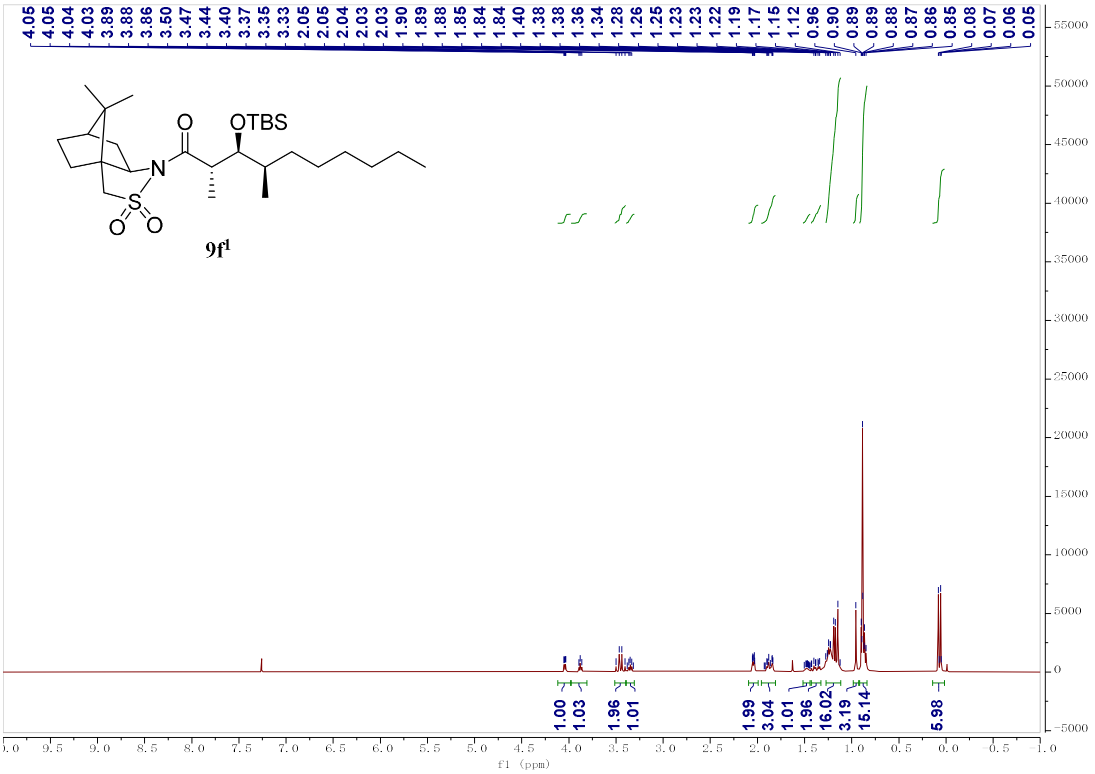


## $^{13}$C NMR spectrum of **9f[1]**

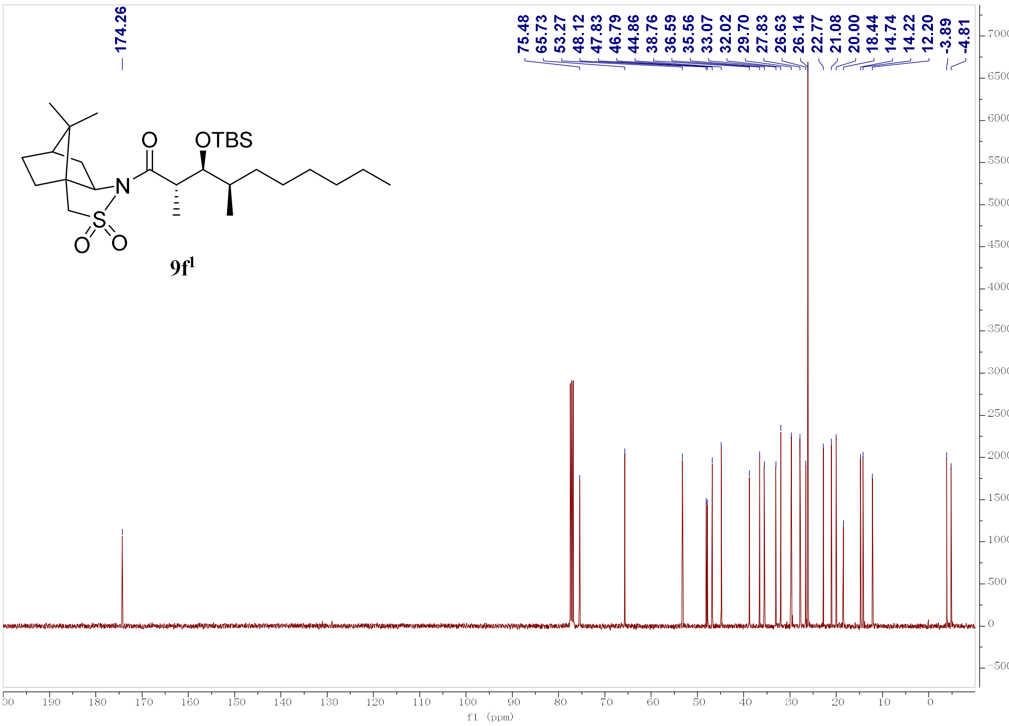

$^{1}H$ NMR spectrum of **9g[1]**

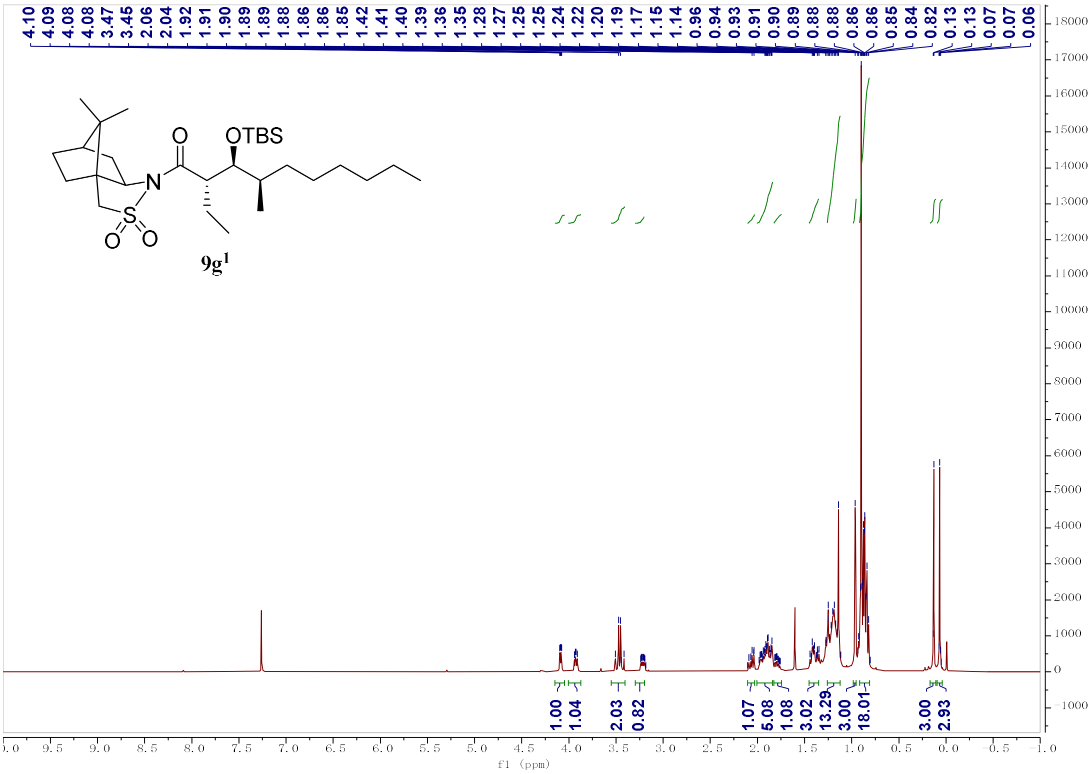


$^{13}C$ NMR spectrum of **9g[1]**

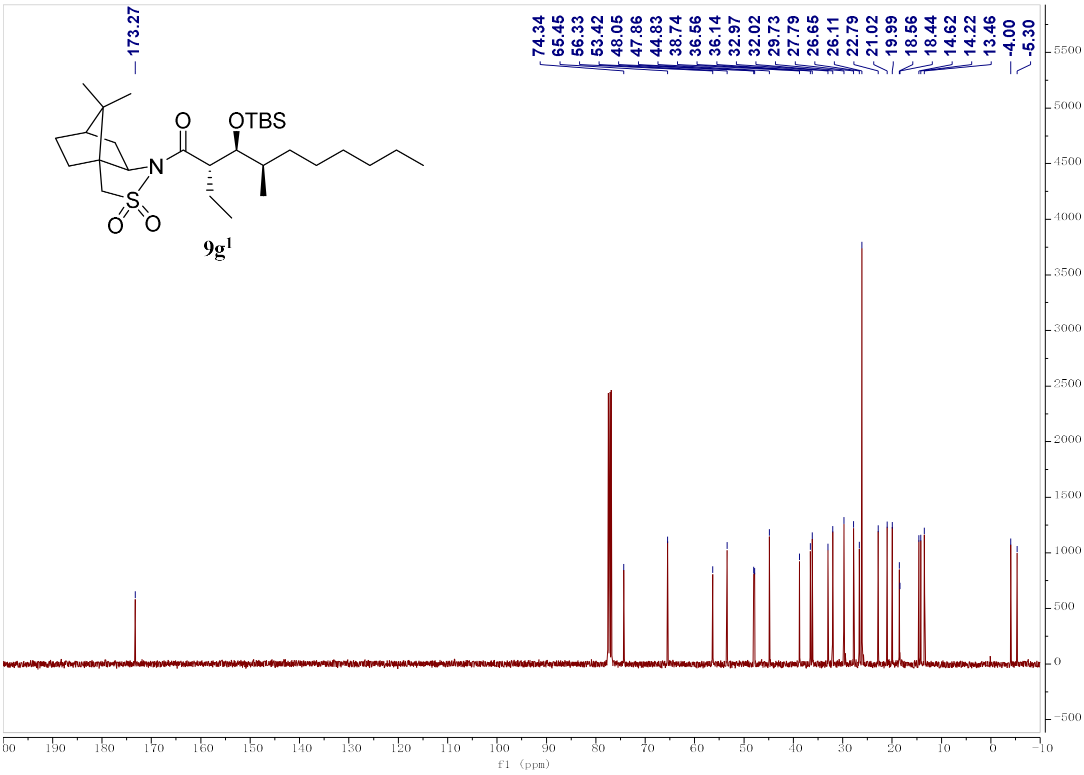

$^{1}$H NMR spectrum of **8a**

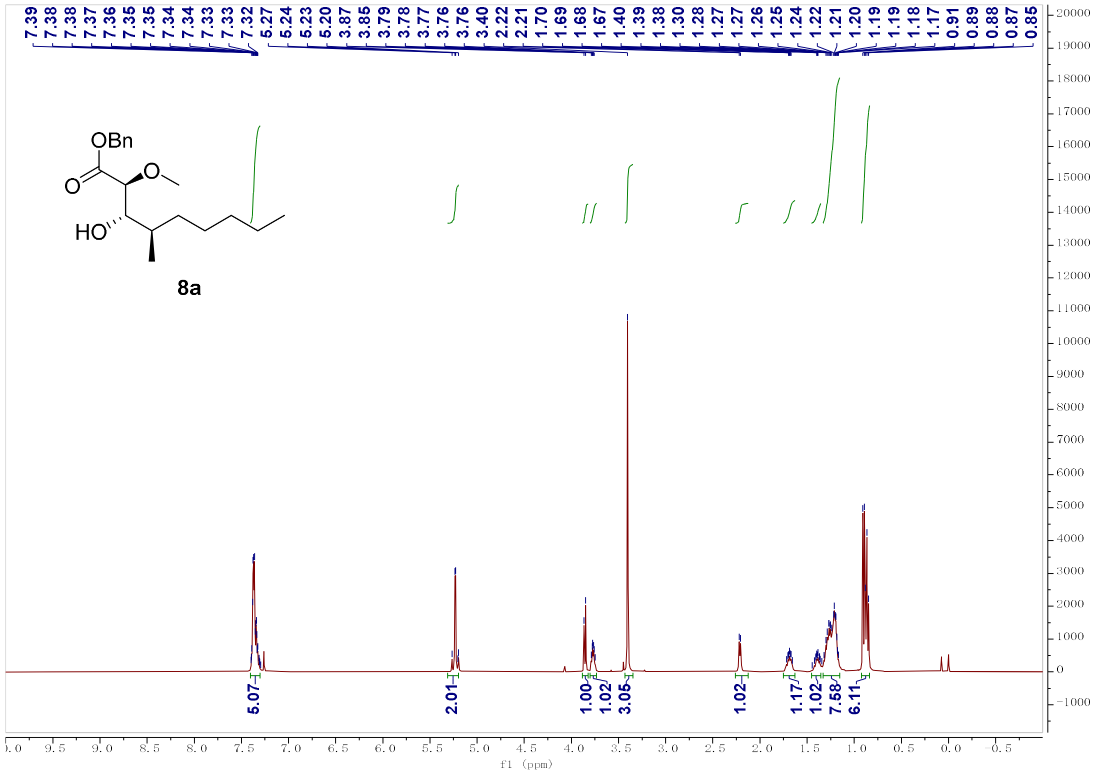

$^{13}$C NMR spectrum of **8a**

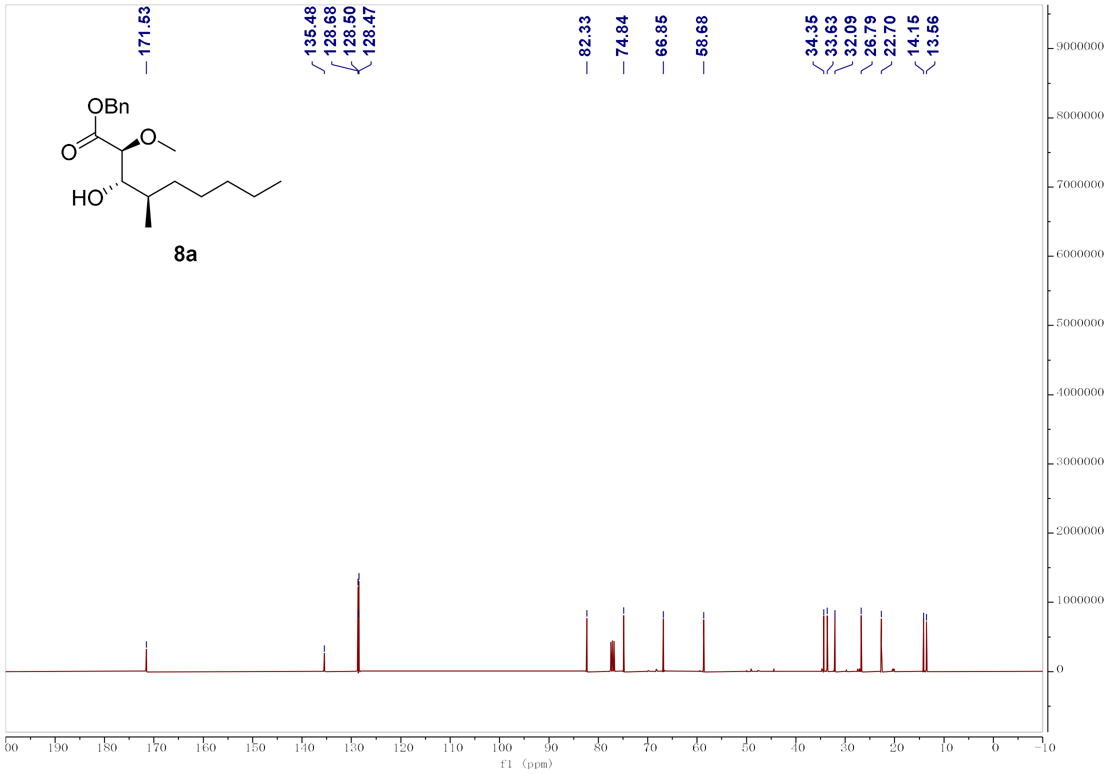

$^{1}$H NMR spectrum of **8b**

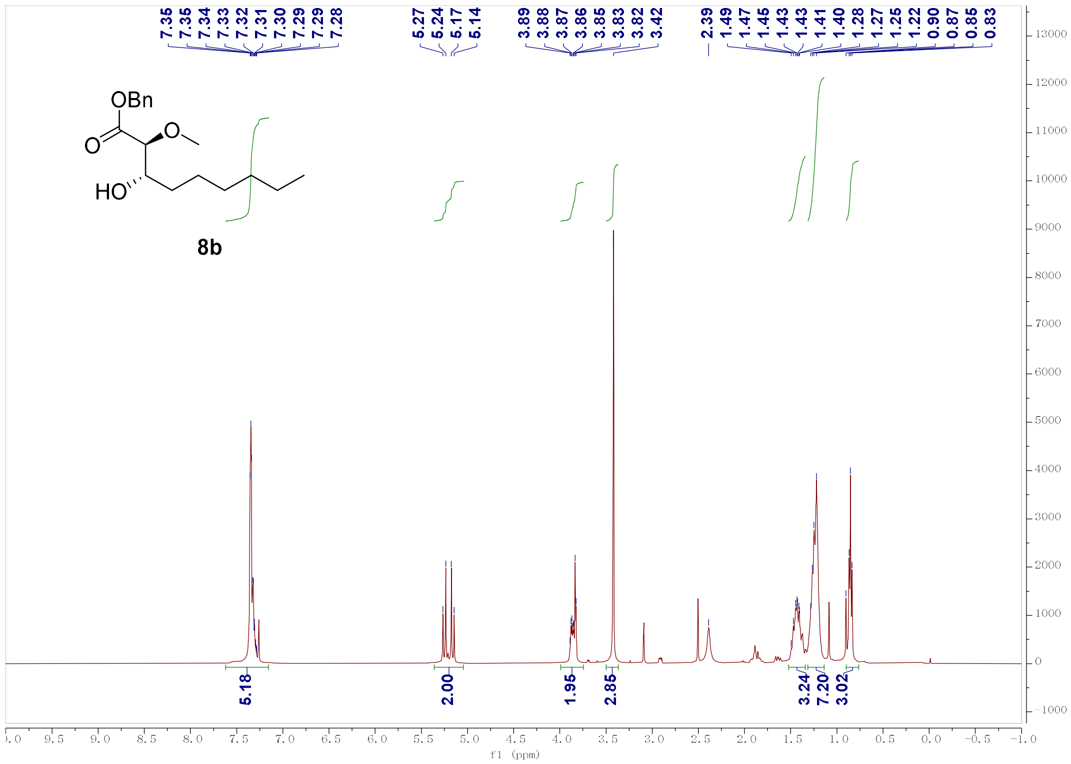


$^{13}$C NMR spectrum of **8b**

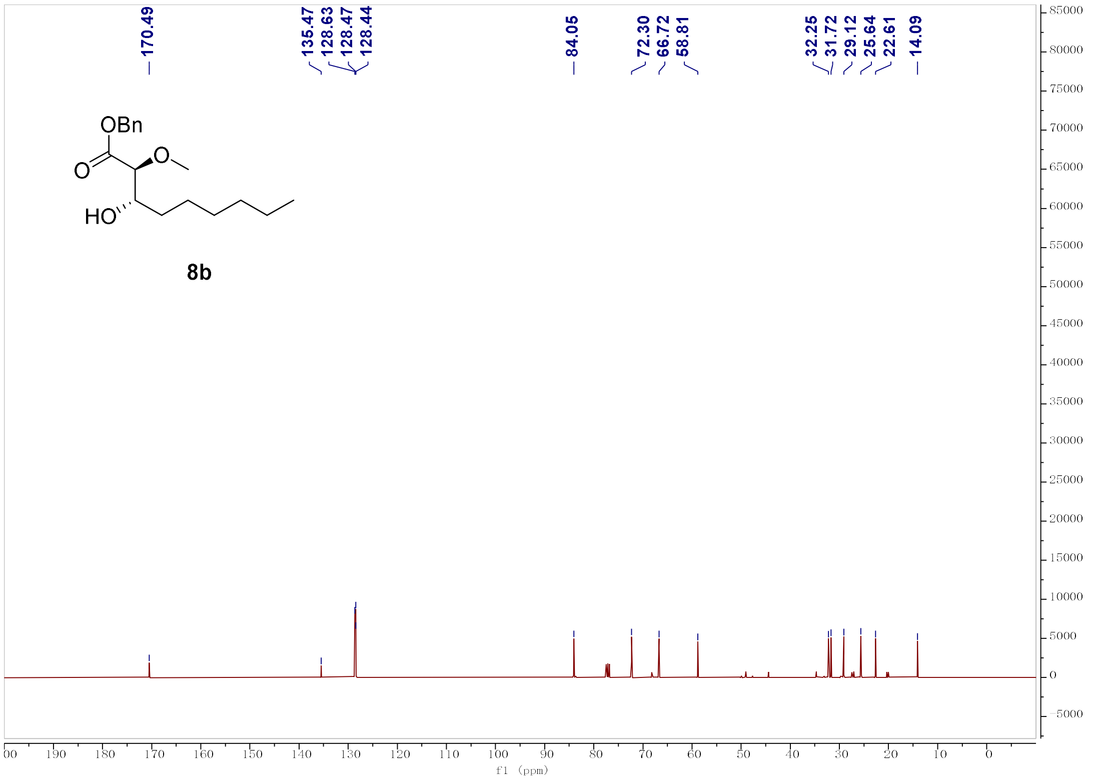

$^{1}$H NMR spectrum of **8c**

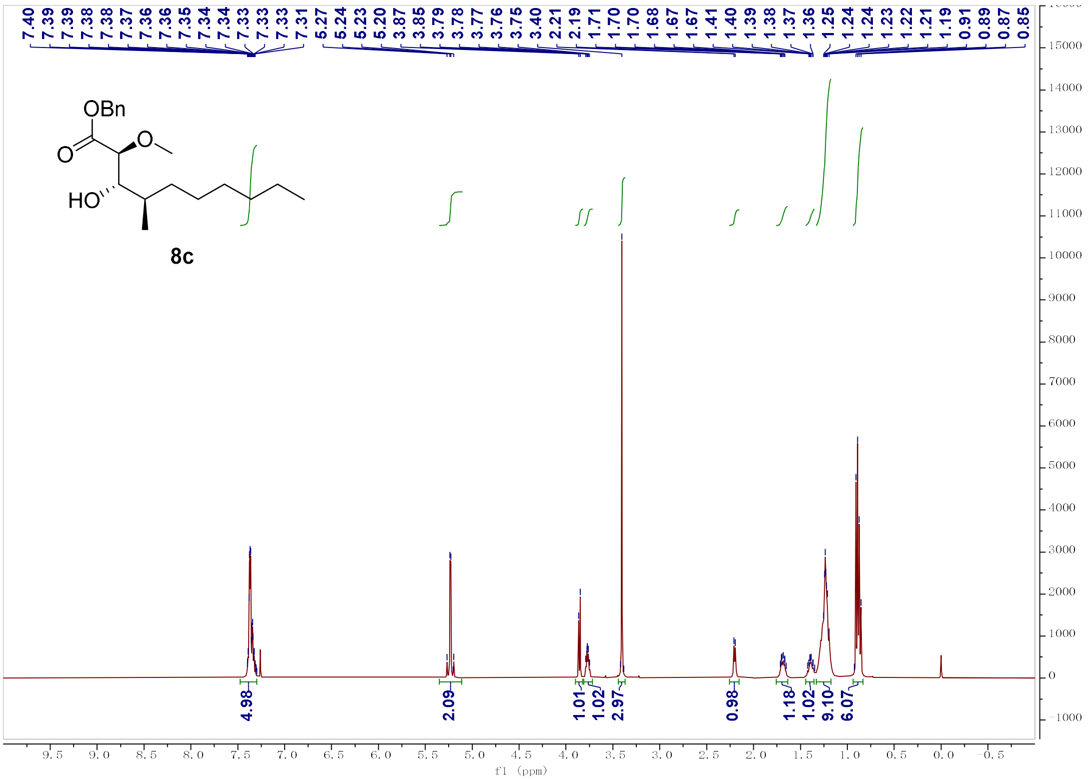

$^{13}$C NMR spectrum of **8c**

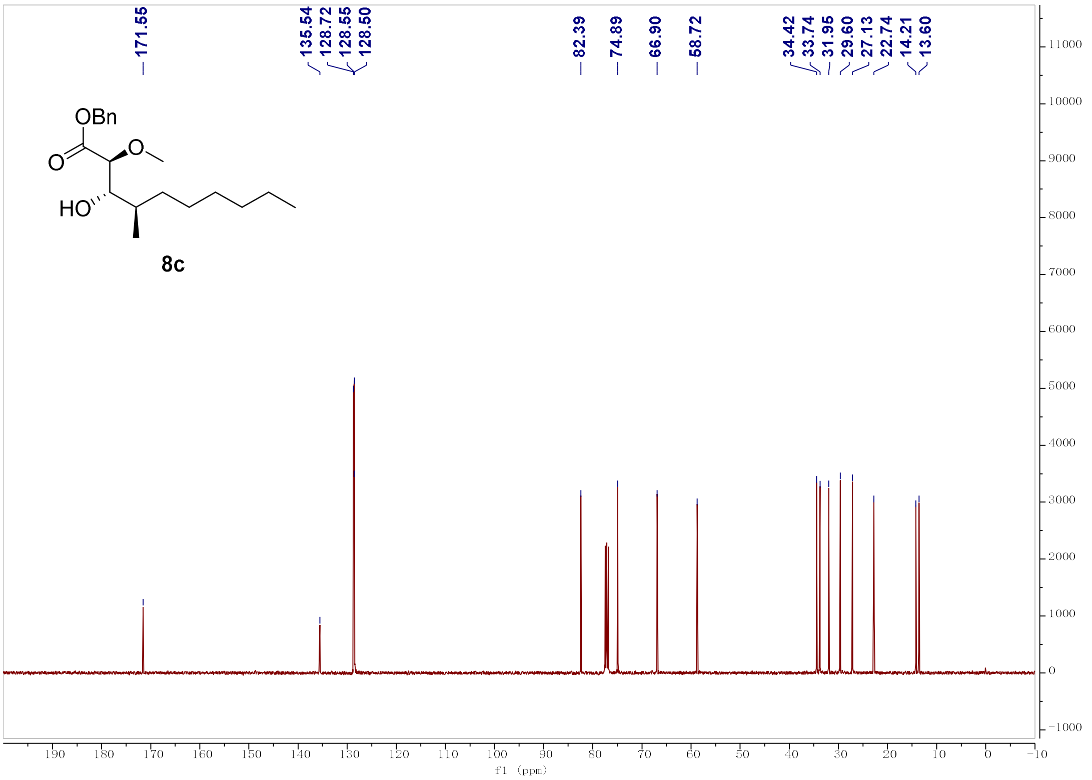

$^{1}$H NMR spectrum of **8d**

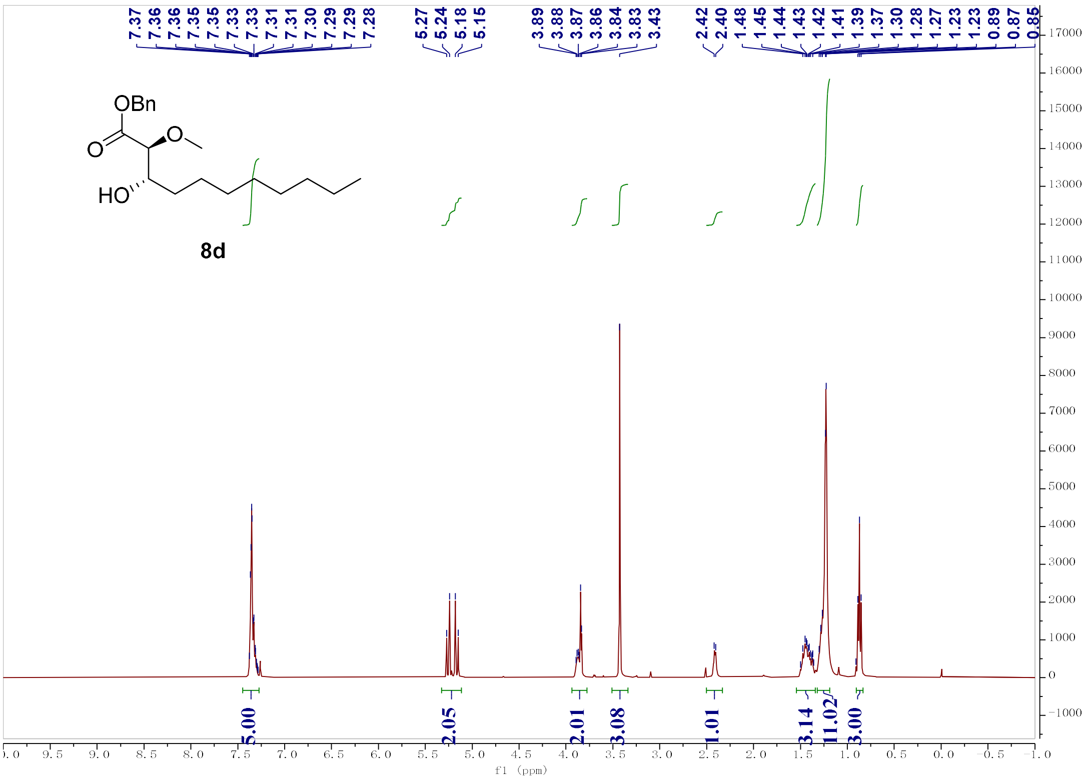

$^{13}$C NMR spectrum of **8d**

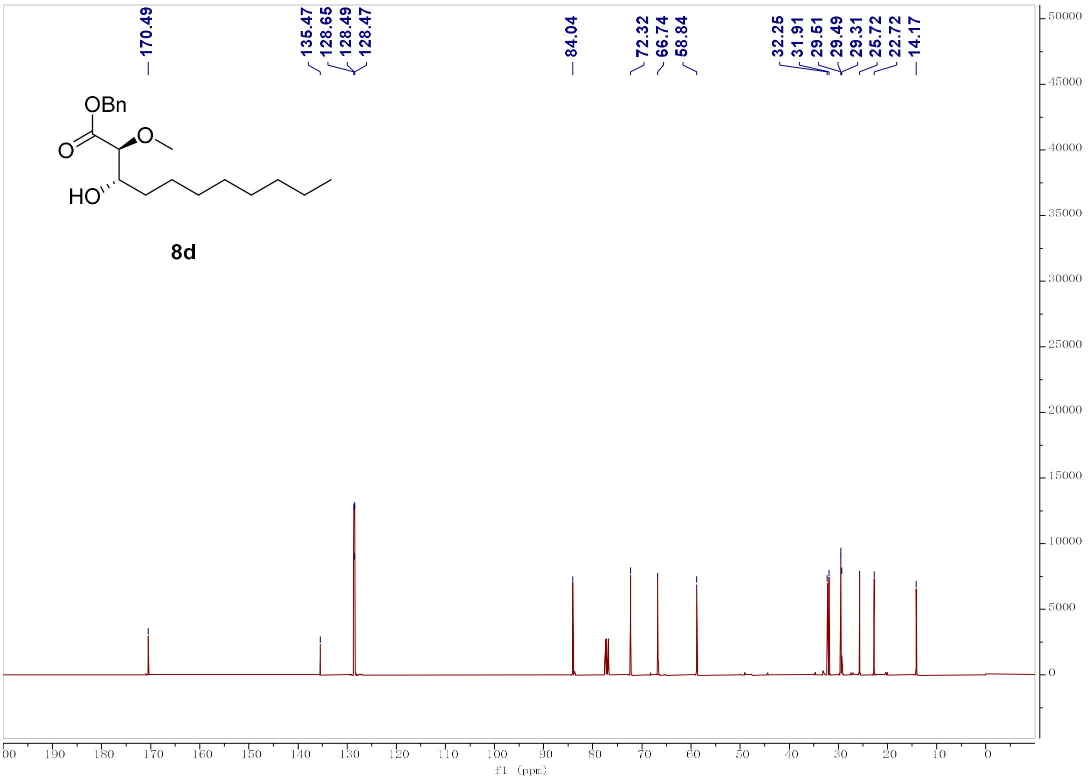

$^{1}$H NMR spectrum of **8e**

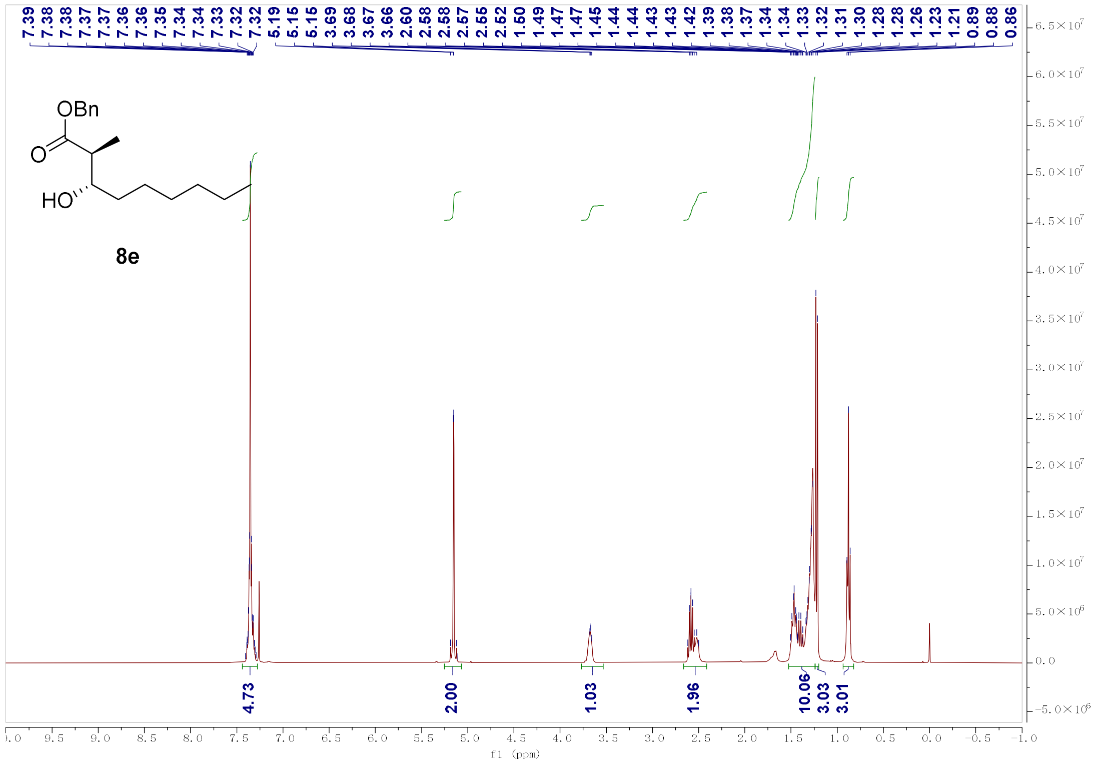

$^{13}$C NMR spectrum of **8e**

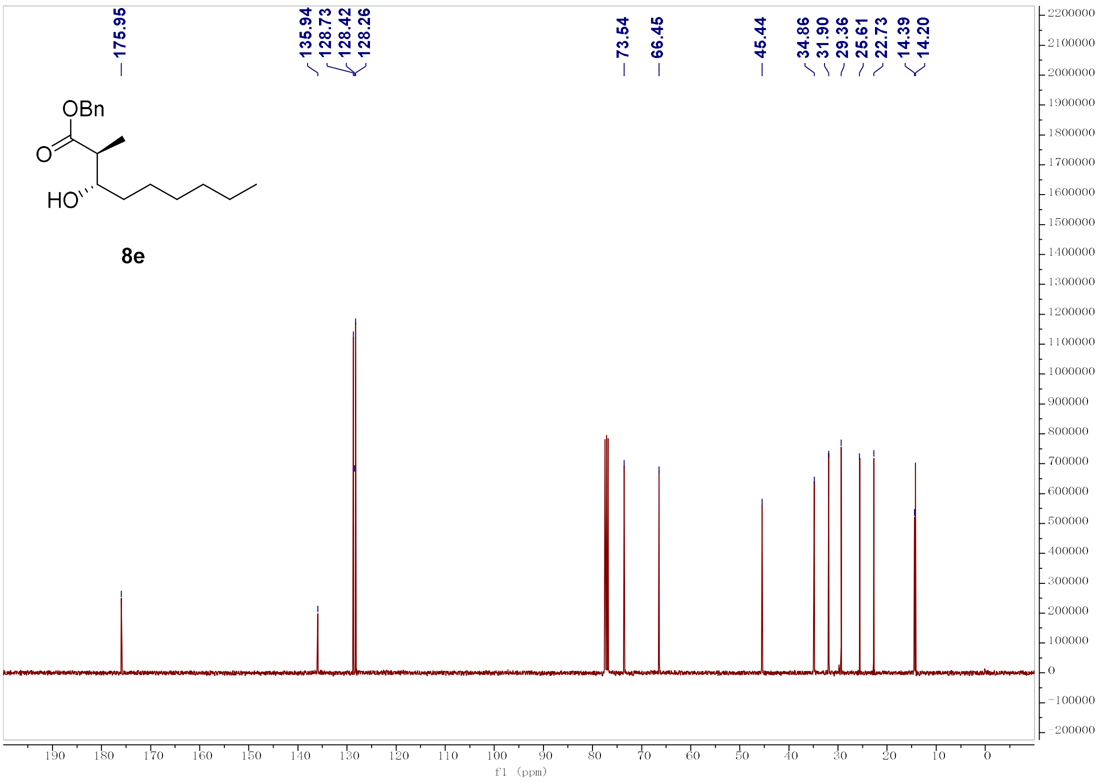

$^{1}$H NMR spectrum of **8f**

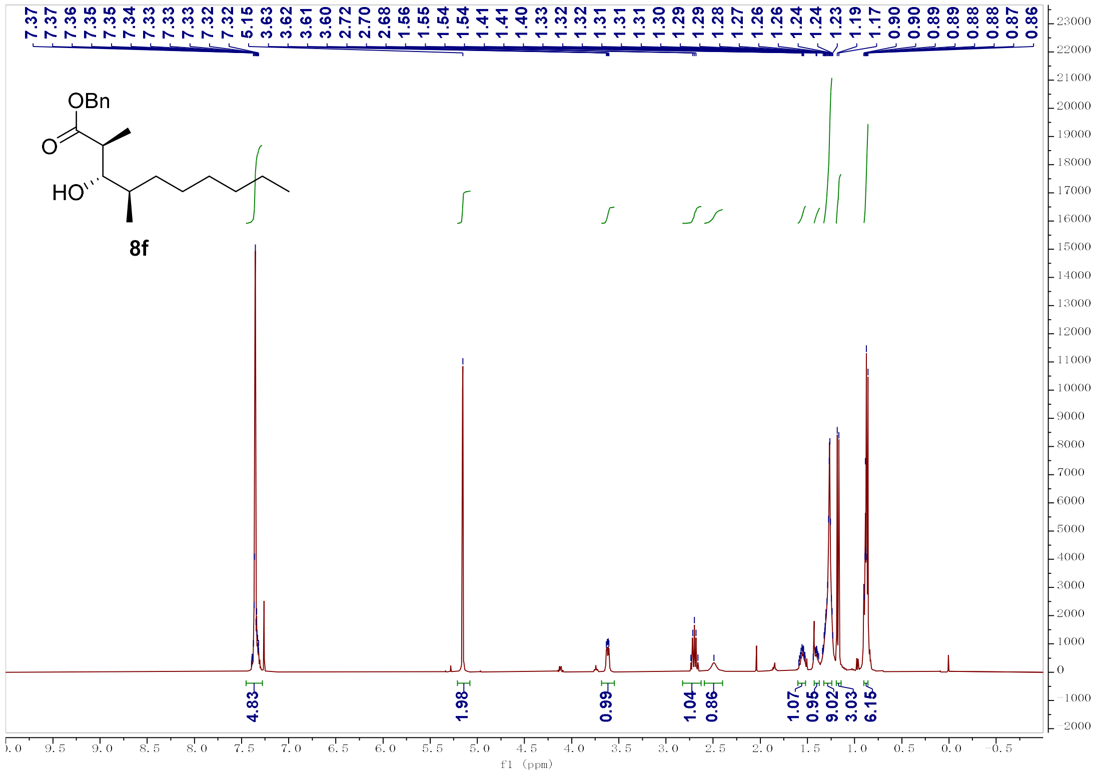

$^{13}$C NMR spectrum of **8f**

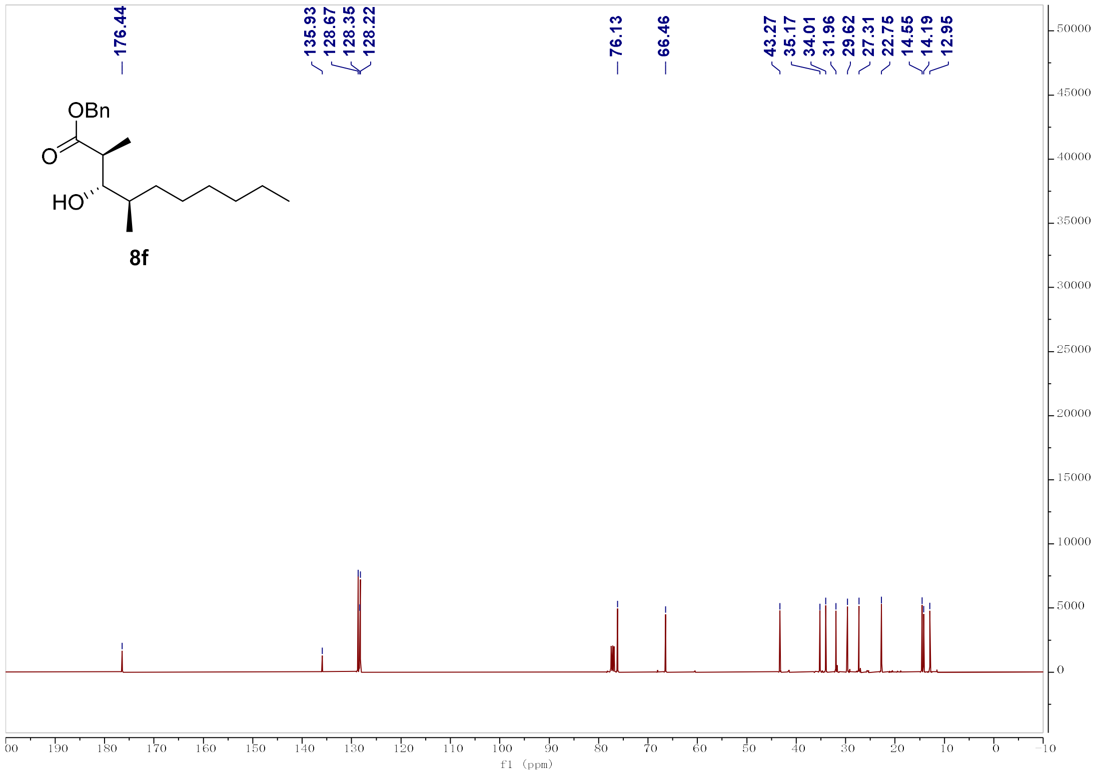

$^{1}$H NMR spectrum of **8g**

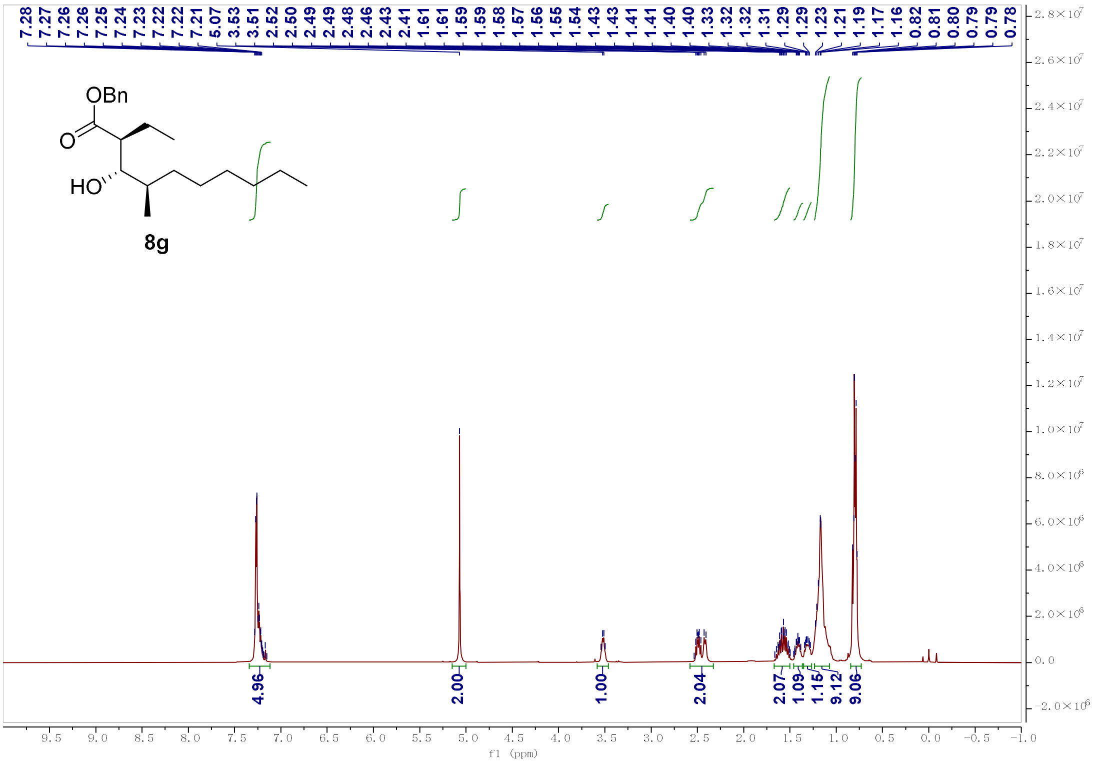

[113]C NMR spectrum of **8g**

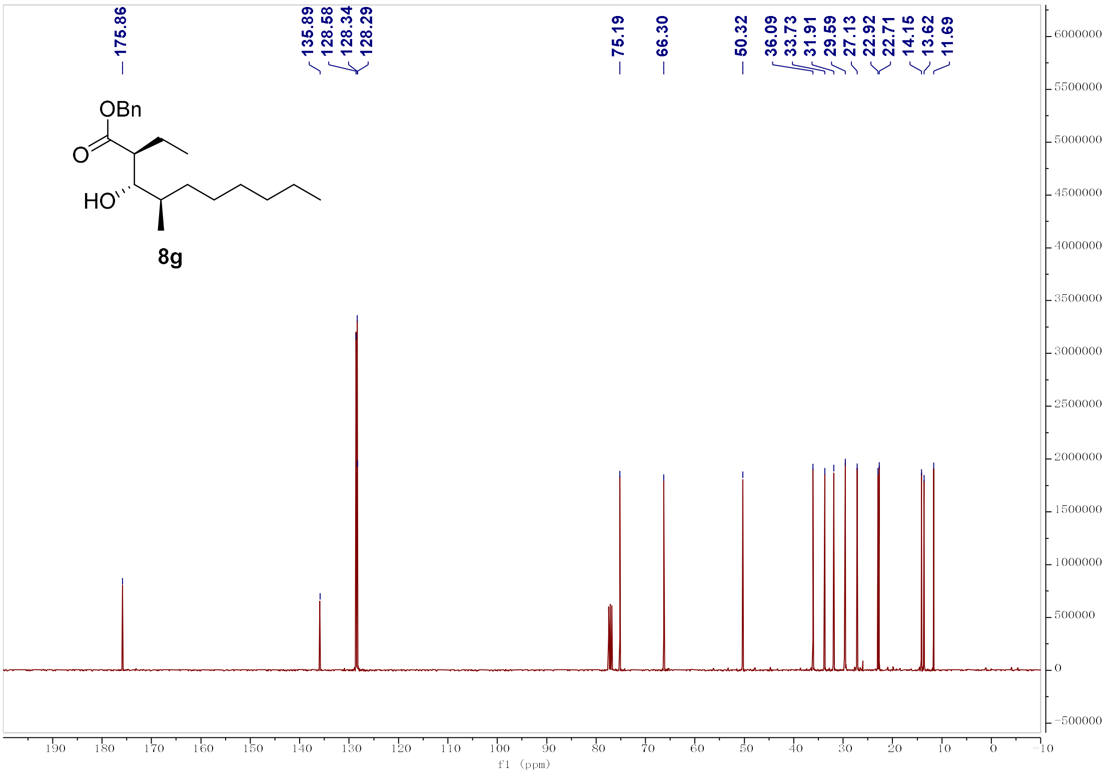

# $^{1}$H NMR spectrum of **6a**

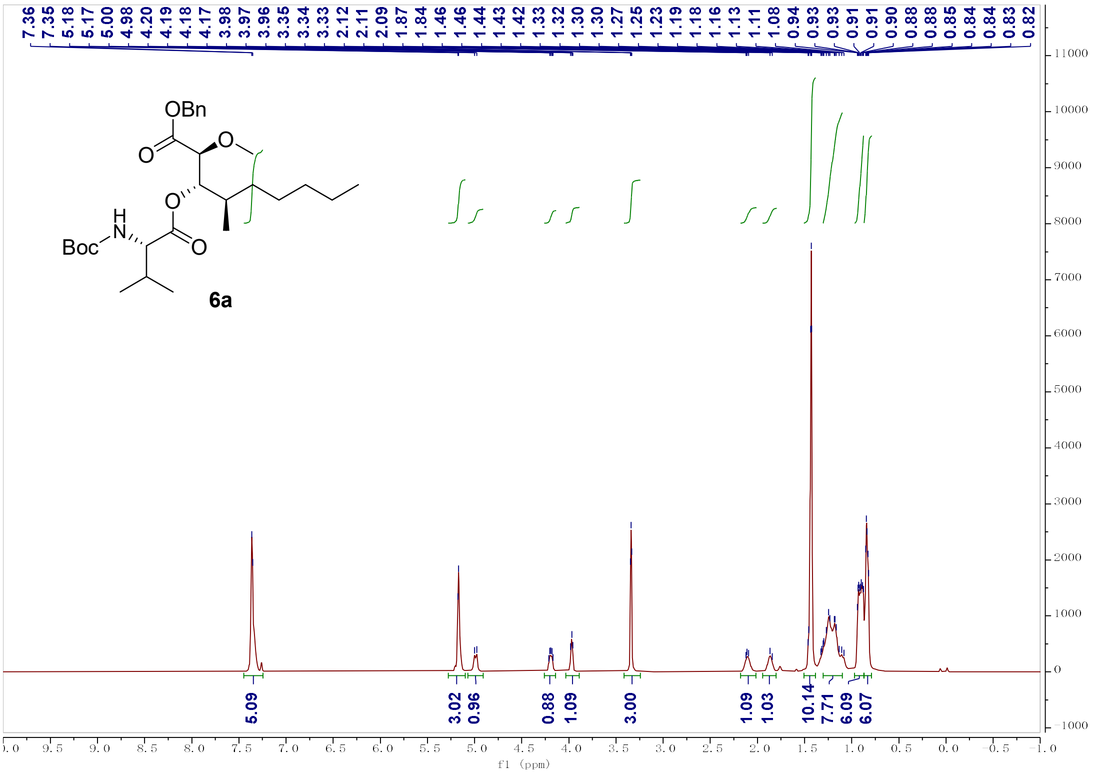

# $^{13}$C NMR spectrum of **6a**

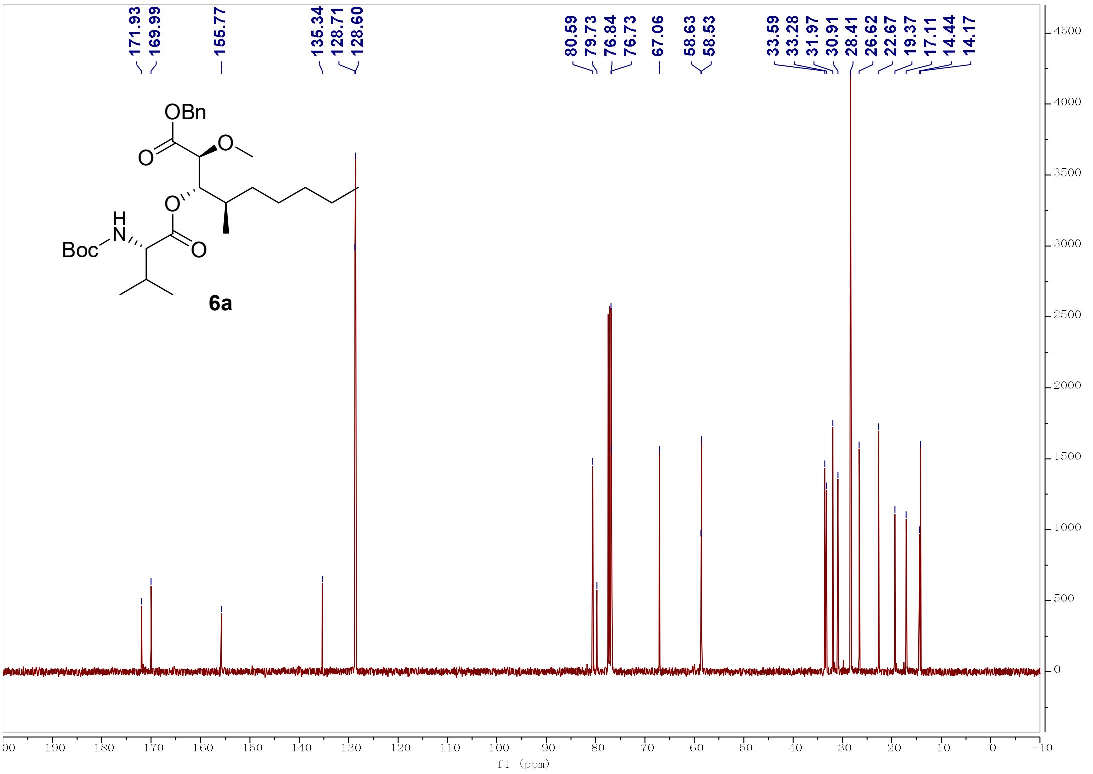

$^{1}$H NMR spectrum of **6b**

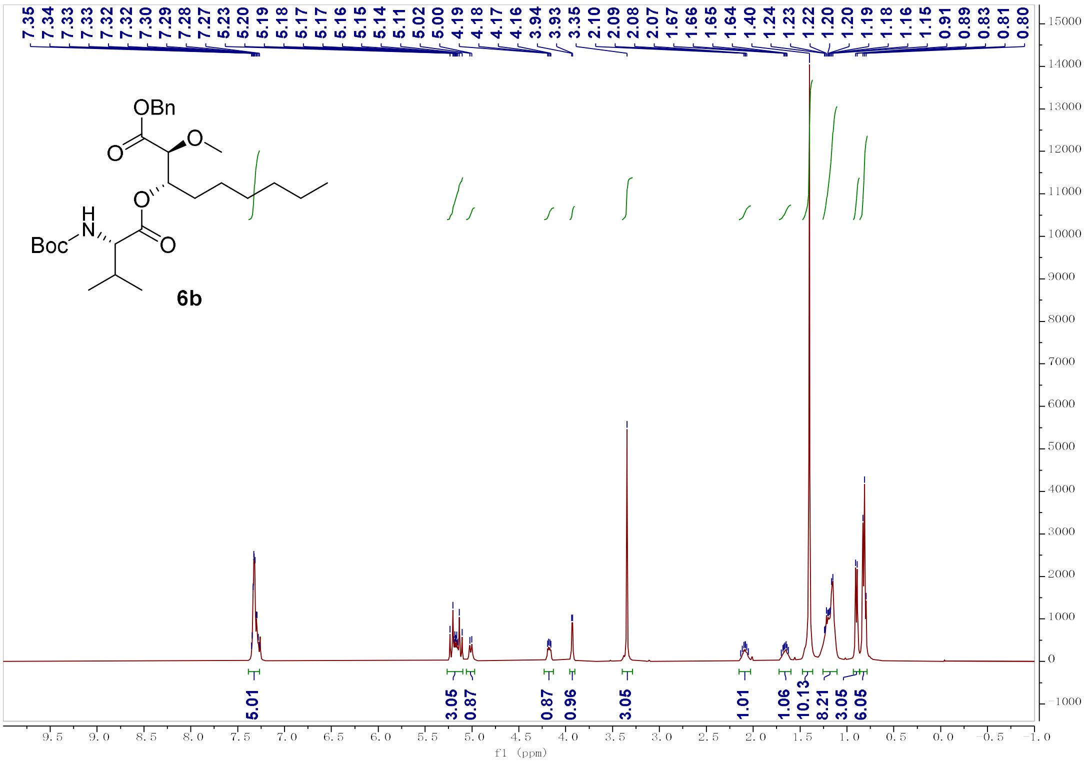


$^{13}$C NMR spectrum of **6b**

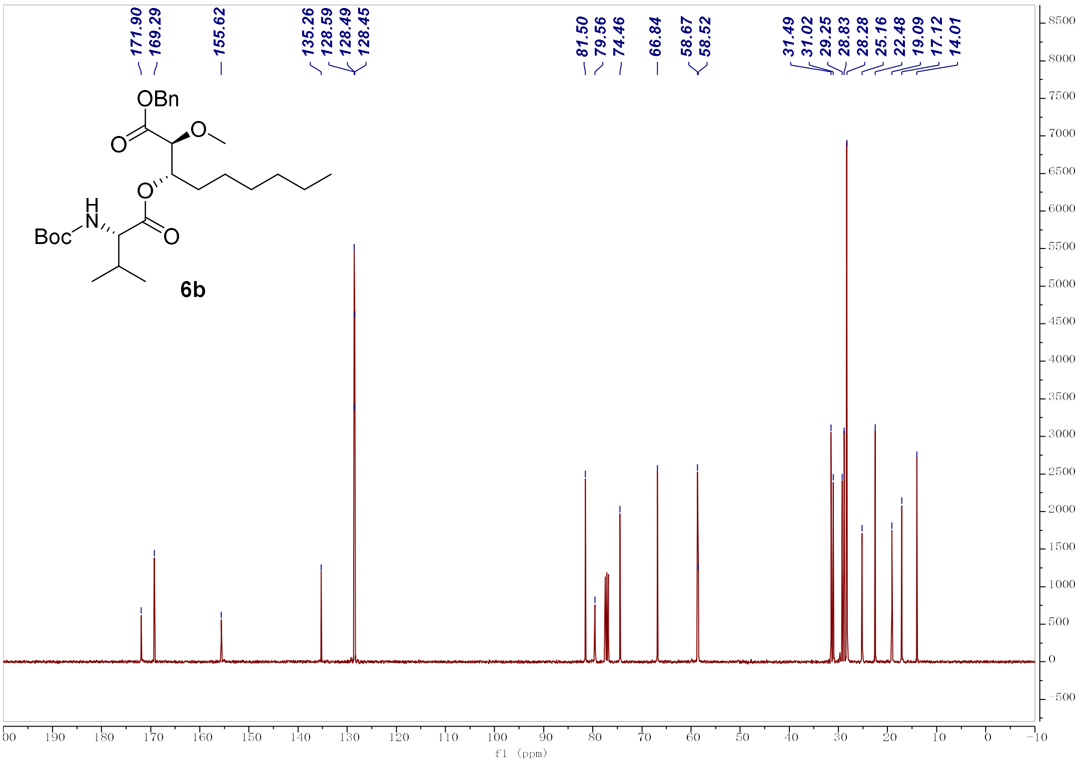

$^{1}$H NMR spectrum of **6c**

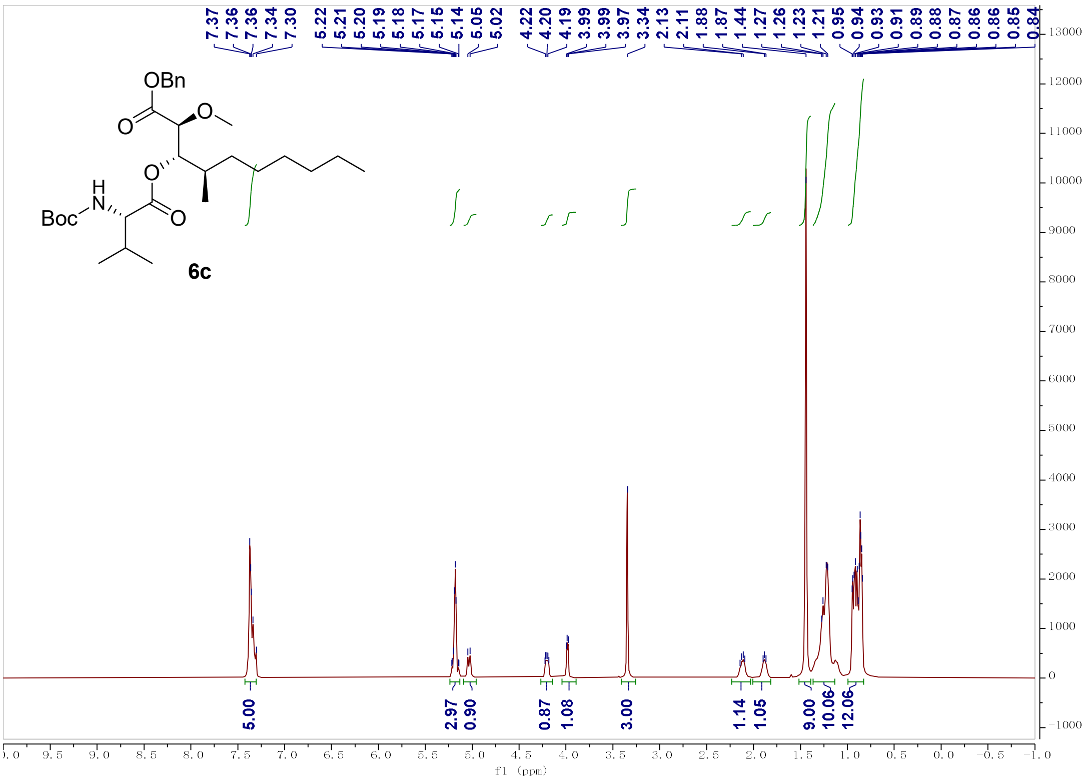

$^{13}$C NMR spectrum of **6c**

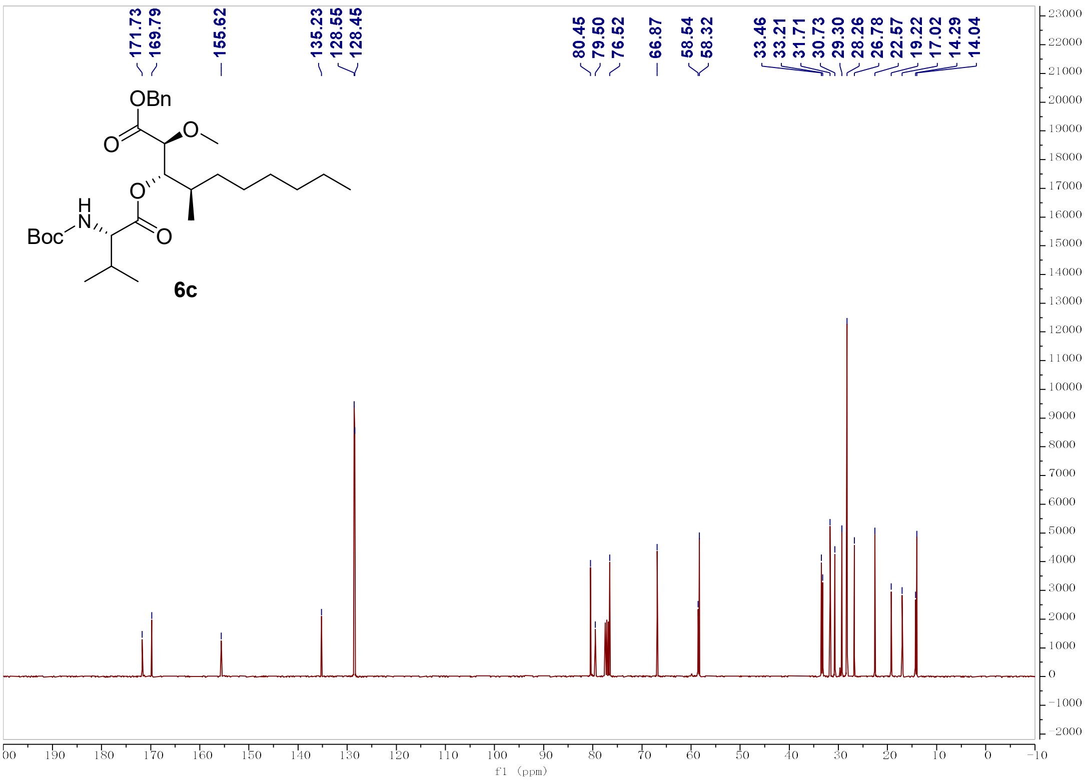

## $^{1}$H NMR spectrum of **6d**

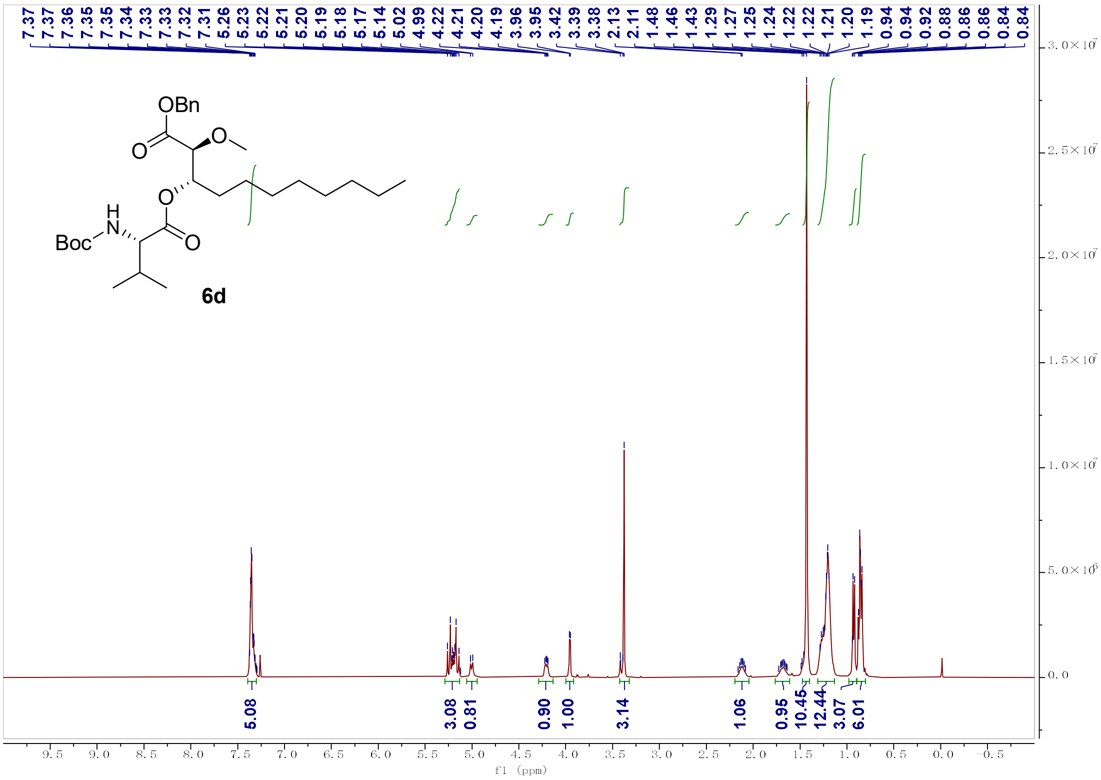

## $^{13}$C NMR spectrum of **6d**

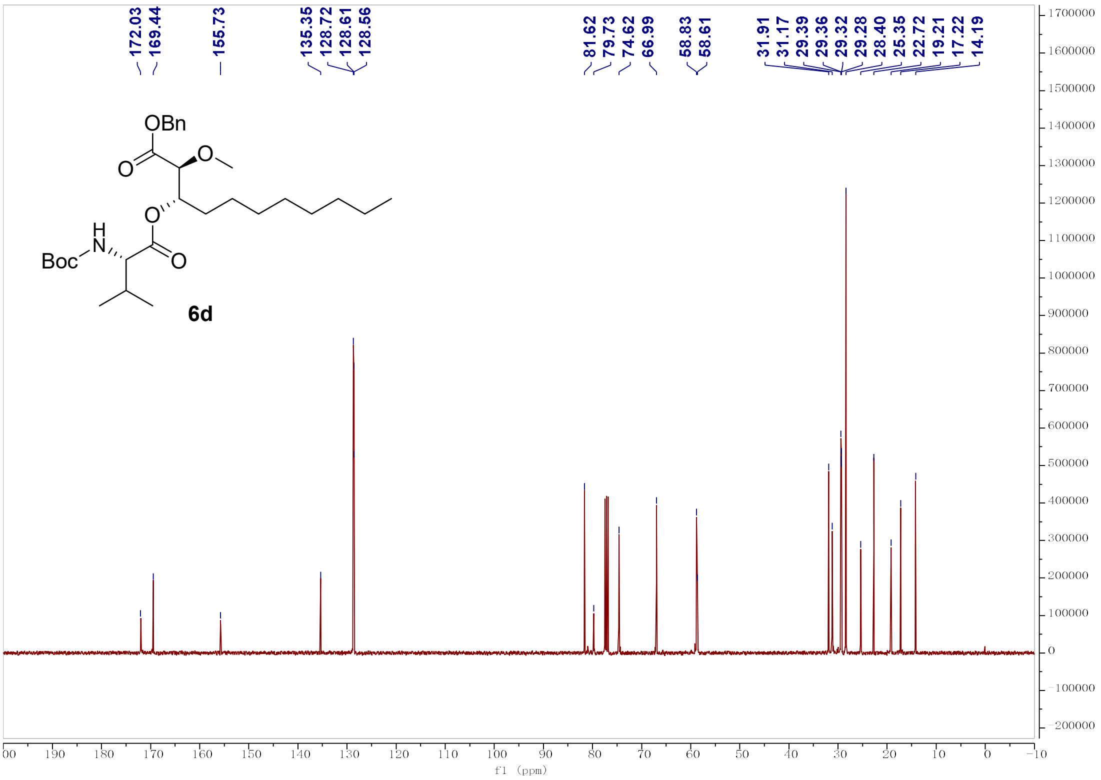

<sup></sup>

## $^{1}$H NMR spectrum of **6e**

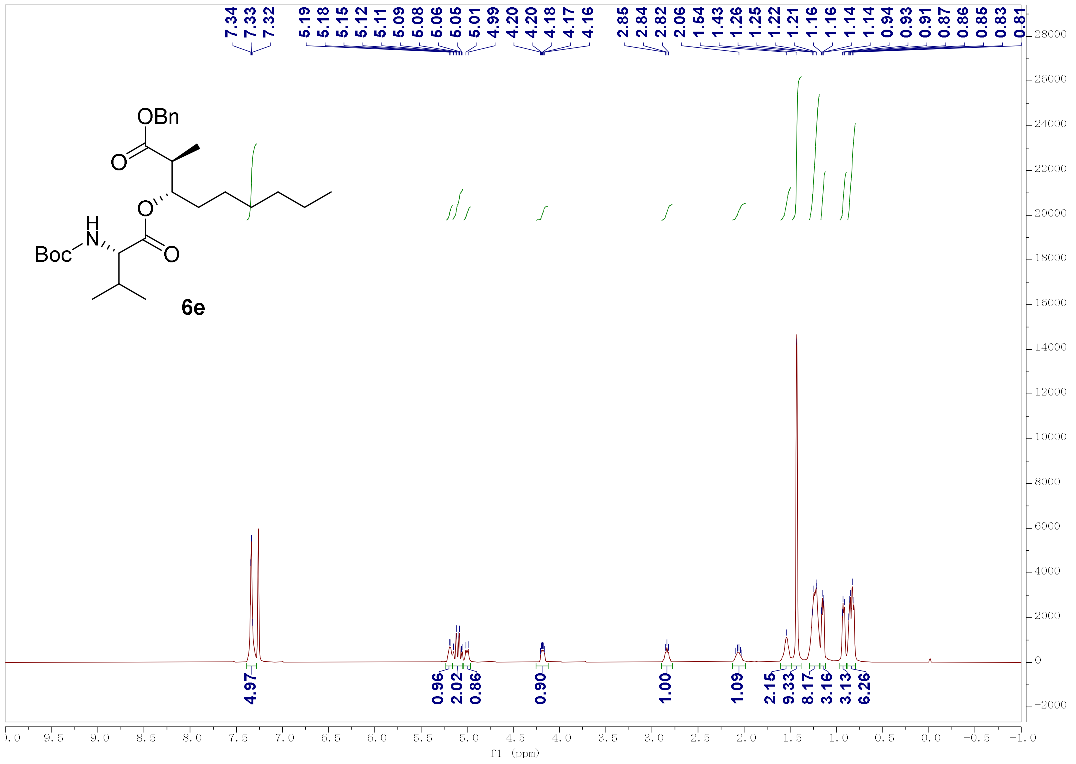

## $^{13}$C NMR spectrum of **6e**

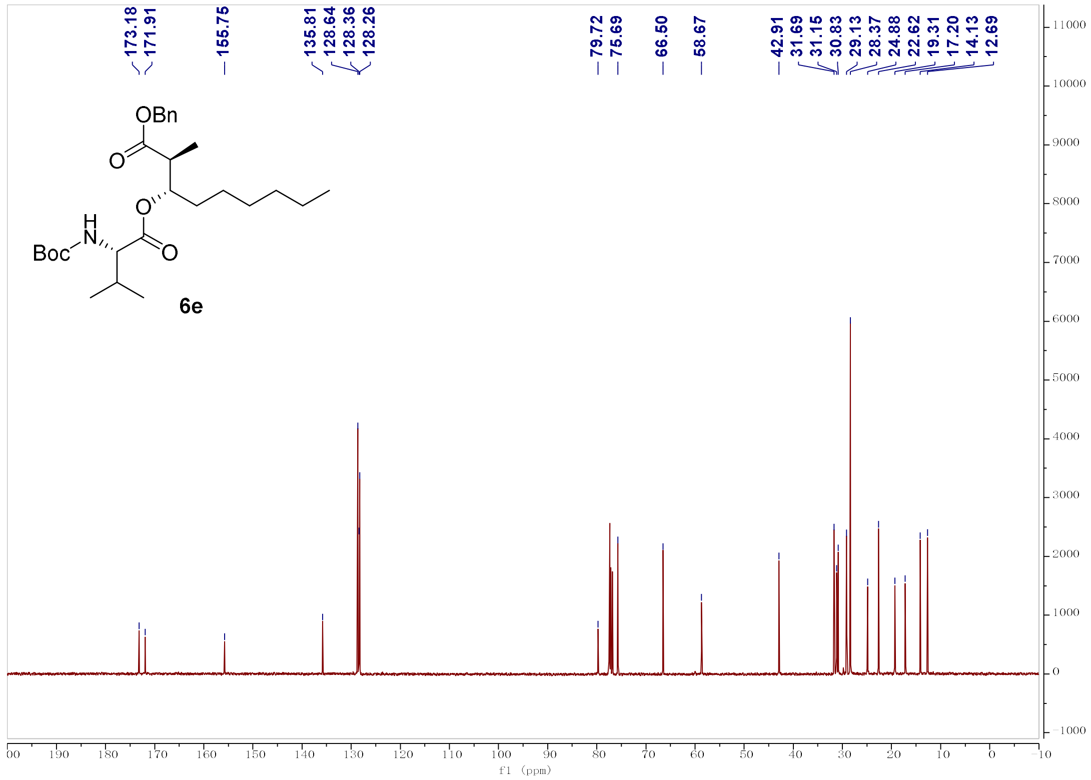

$^{1}$H NMR spectrum of **6f**

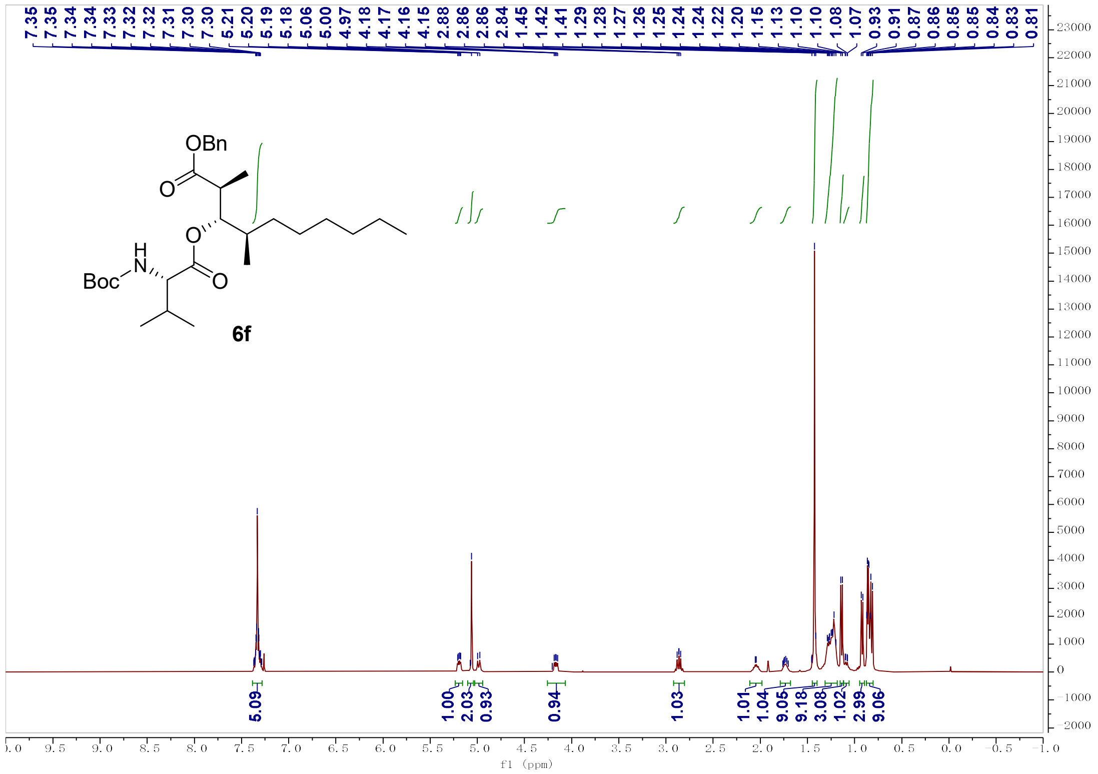

$^{13}$C NMR spectrum of **6f**

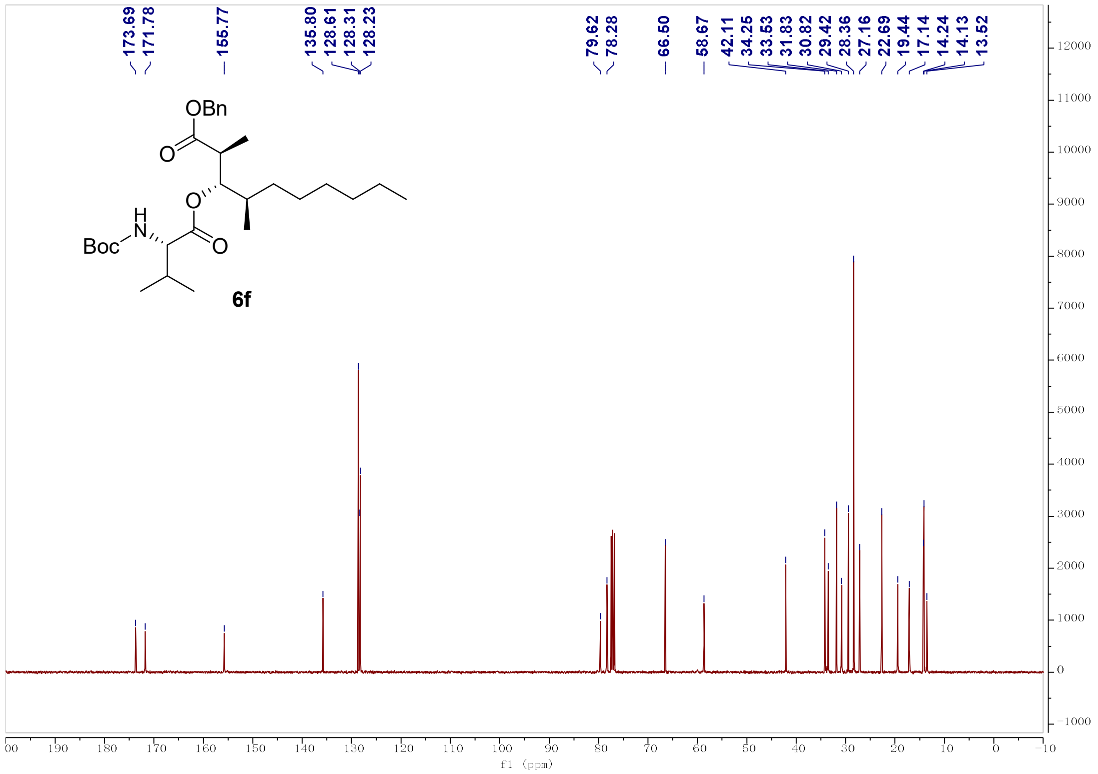

$^{1}$H NMR spectrum of **6g**

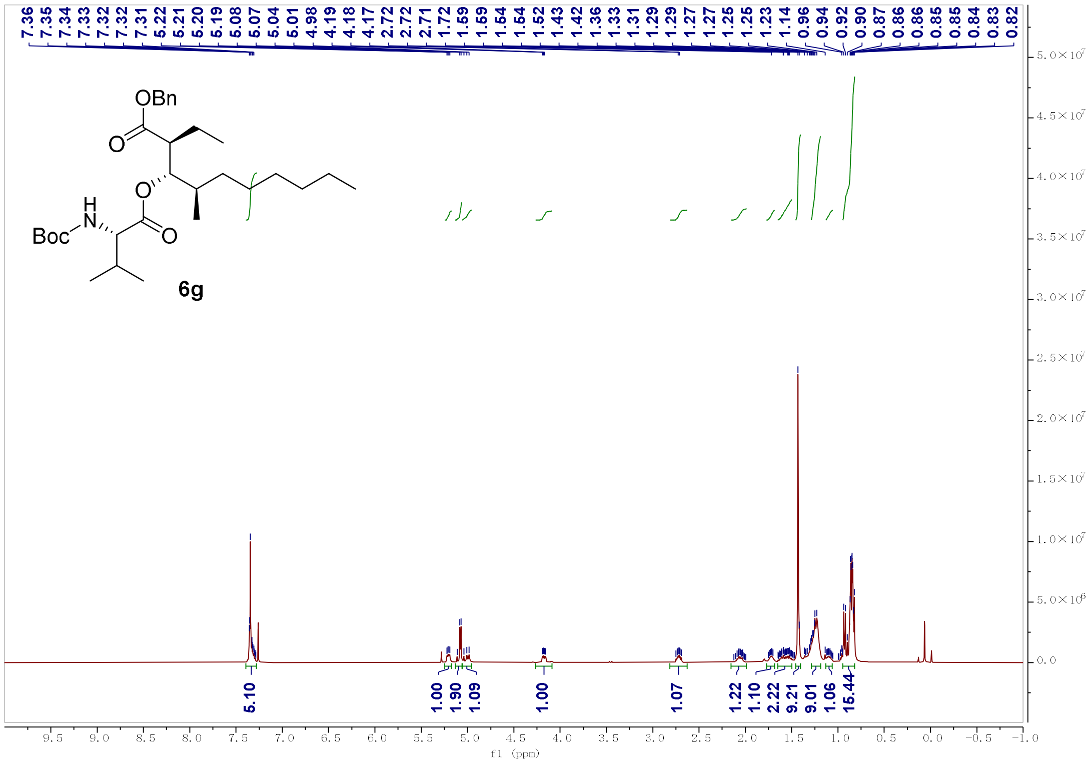

$^{13}$C NMR spectrum of **6g**

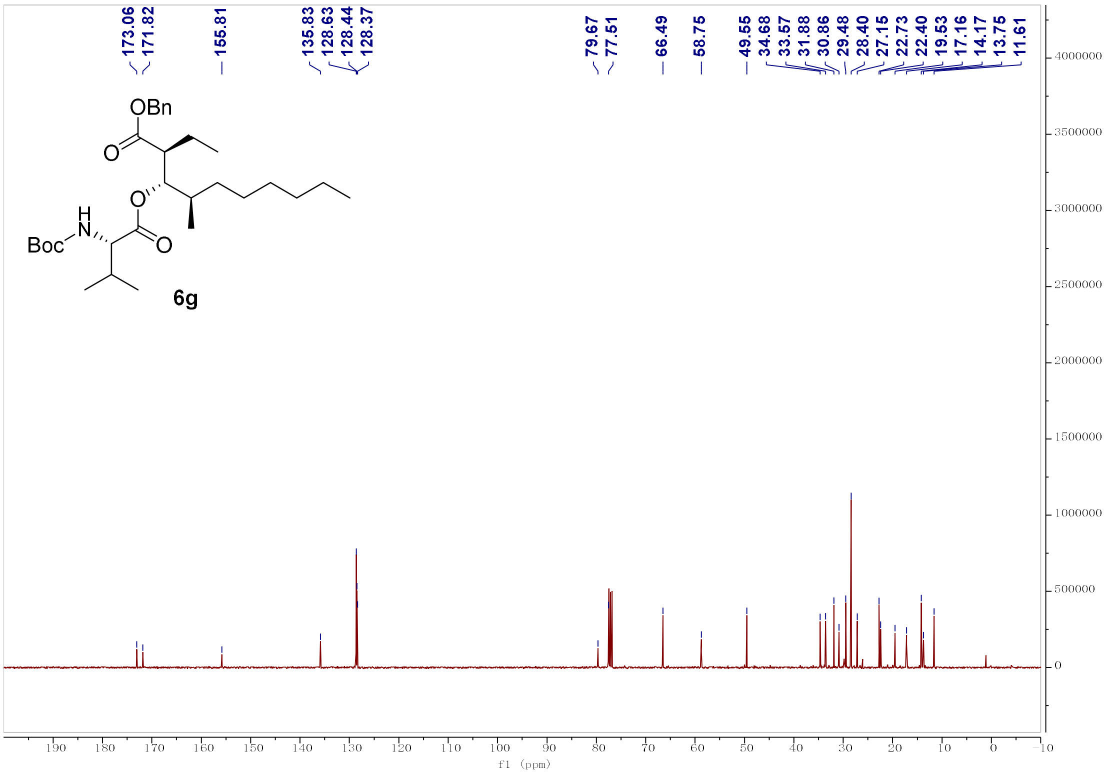

$^{1}$H NMR spectrum of **4a**

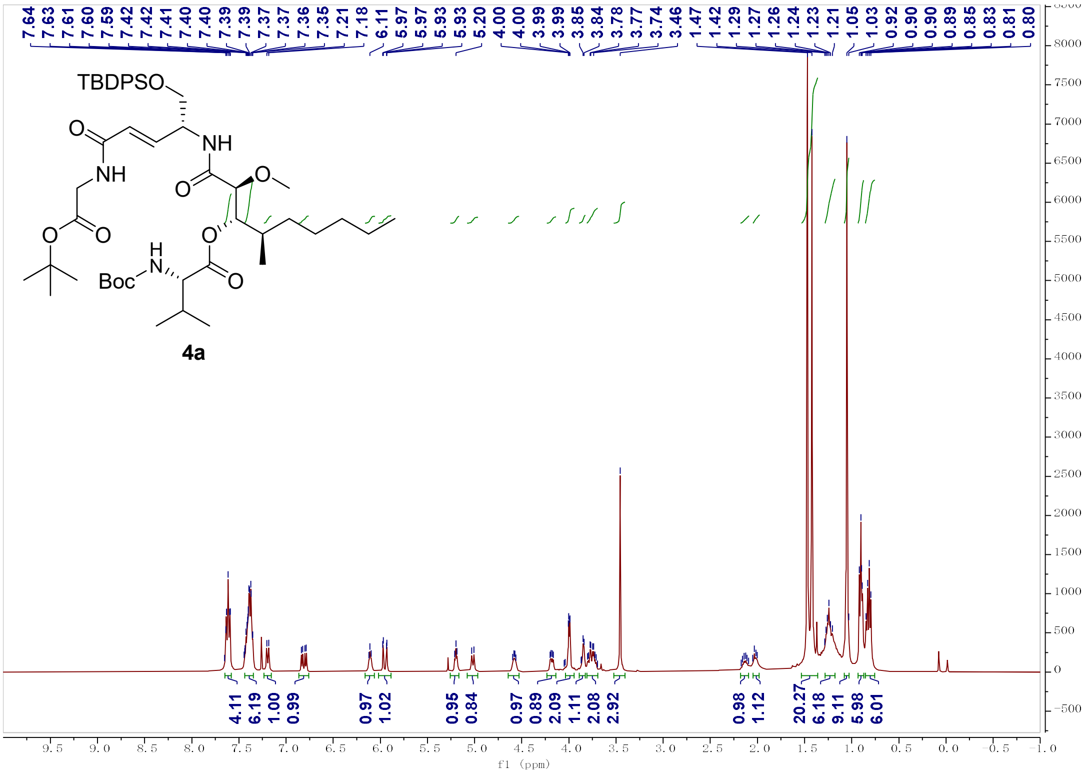

$^{13}$C NMR spectrum of **4a**

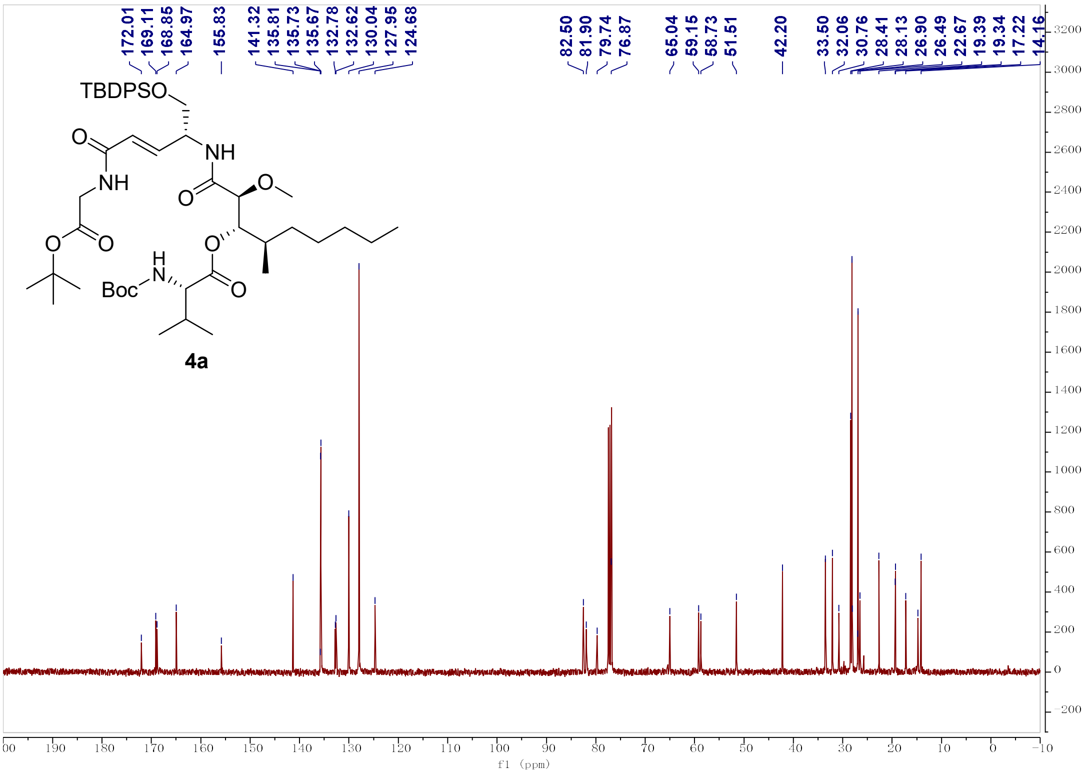

$^{1}$H NMR spectrum of **4b**

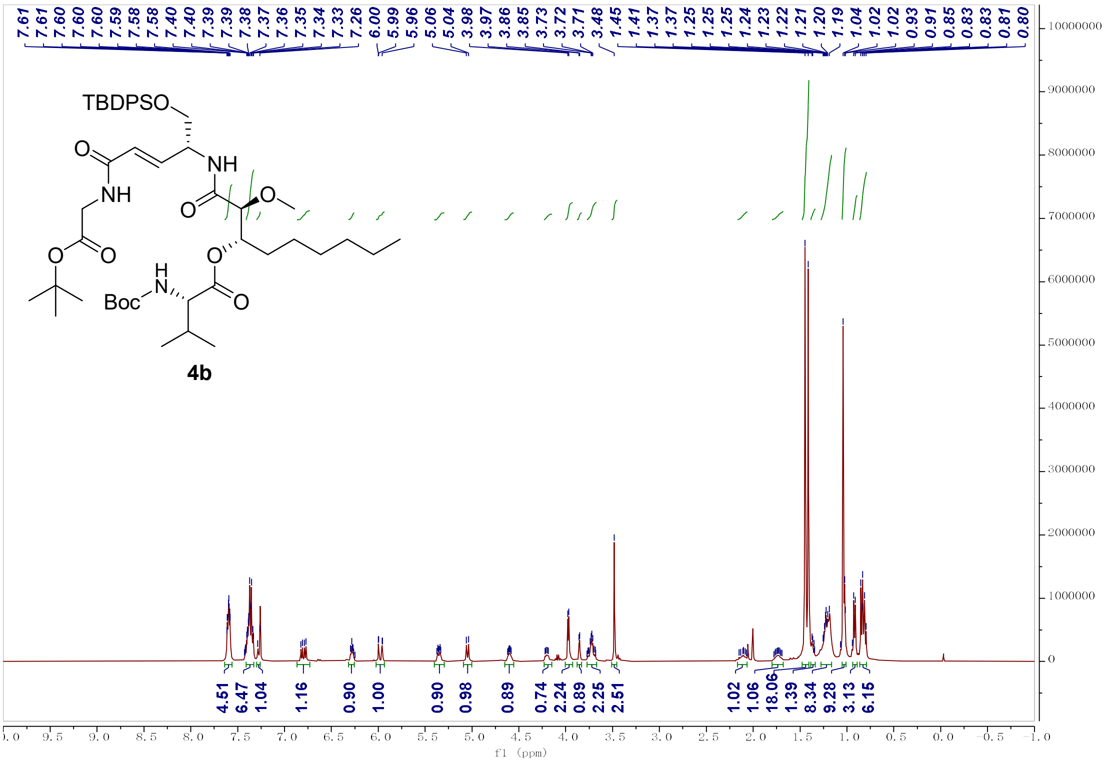

$^{13}$C NMR spectrum of **4b**

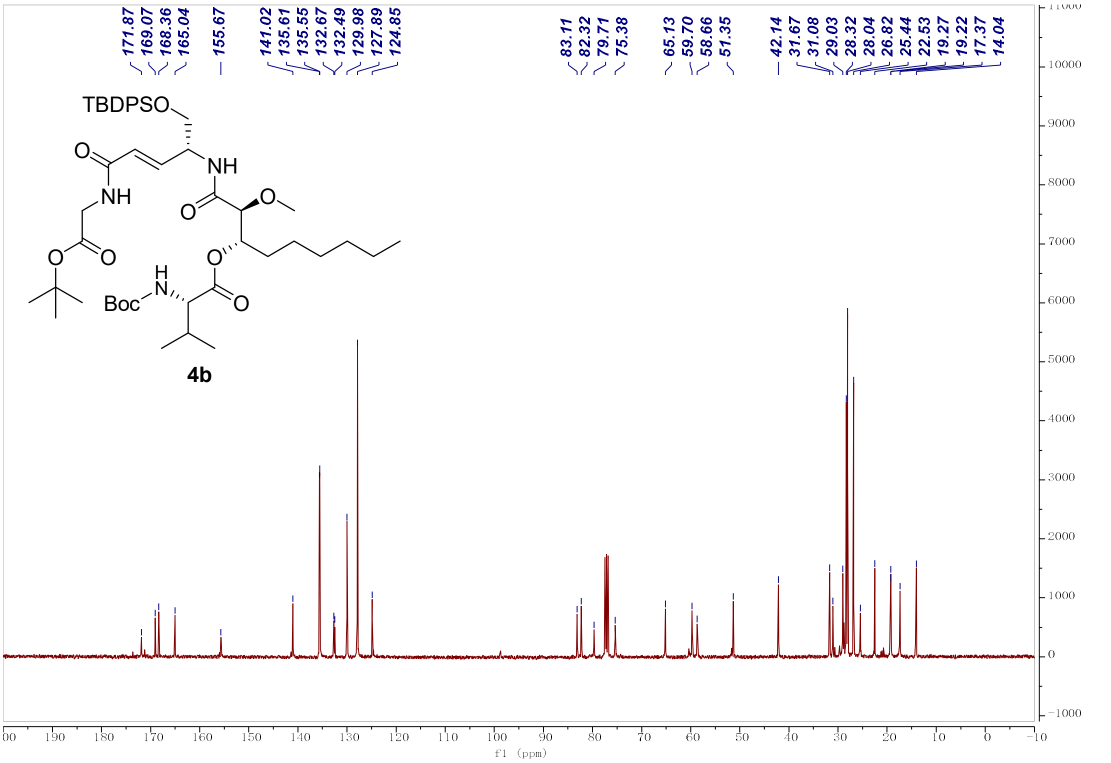

$^{1}$H NMR spectrum of **4c**

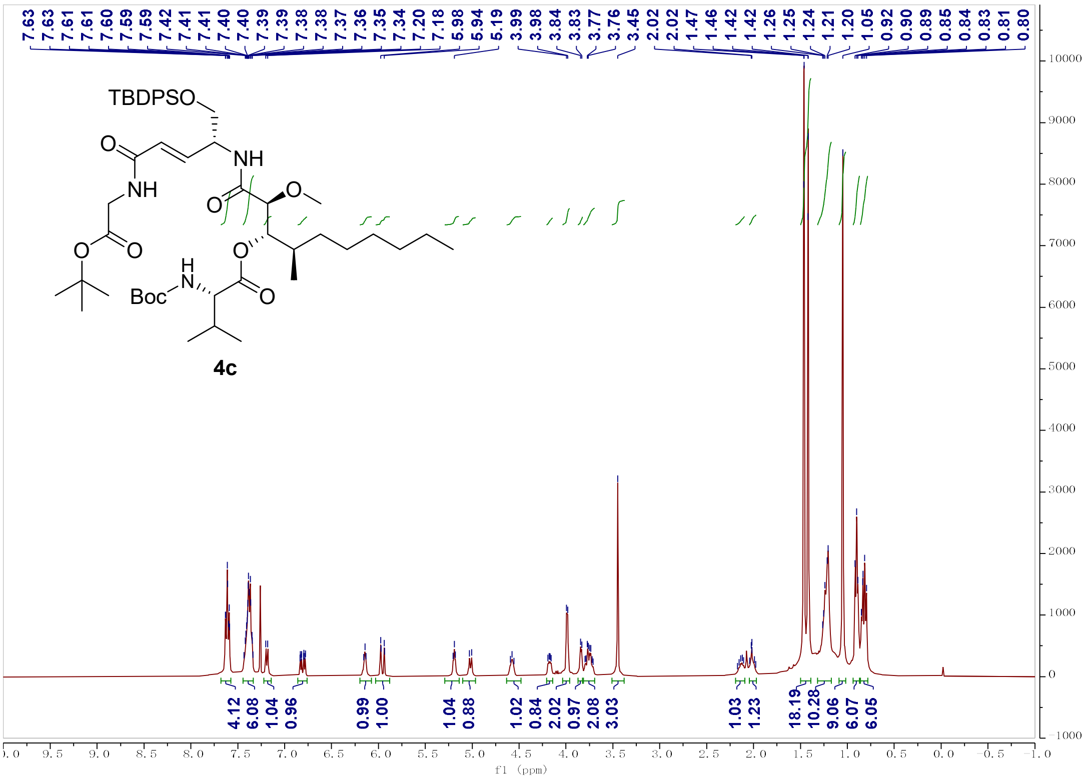

$^{13}$C NMR spectrum of **4c**

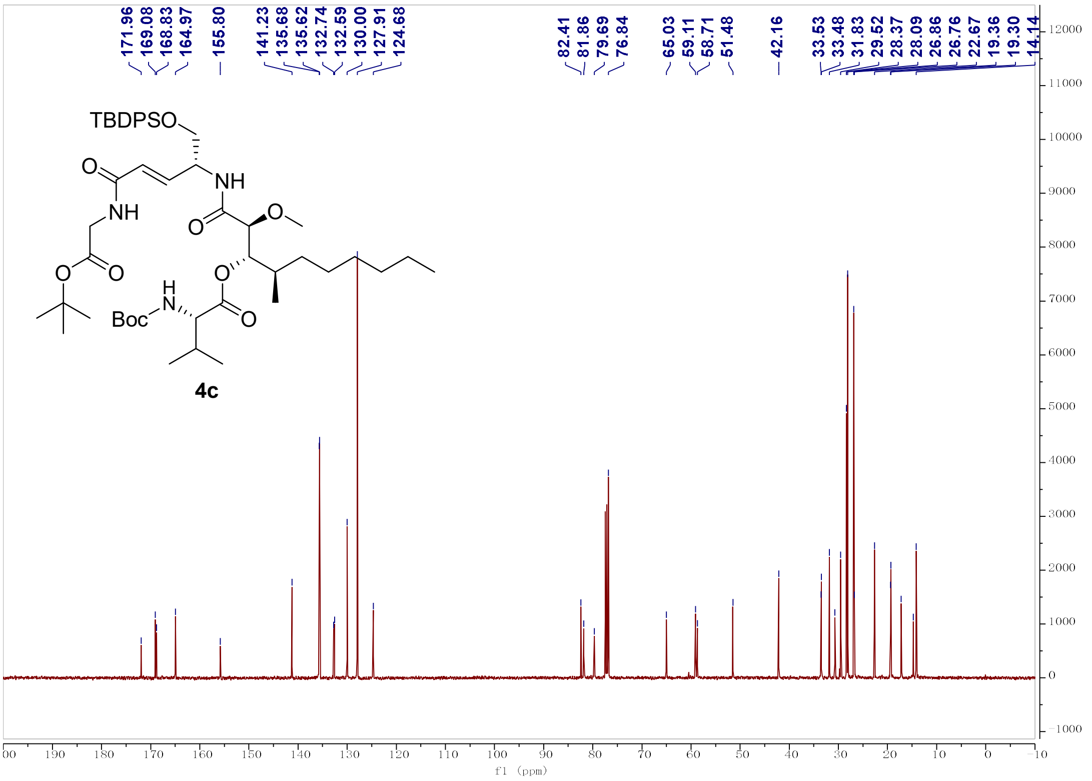

$^{1}H$ NMR spectrum of **4d**

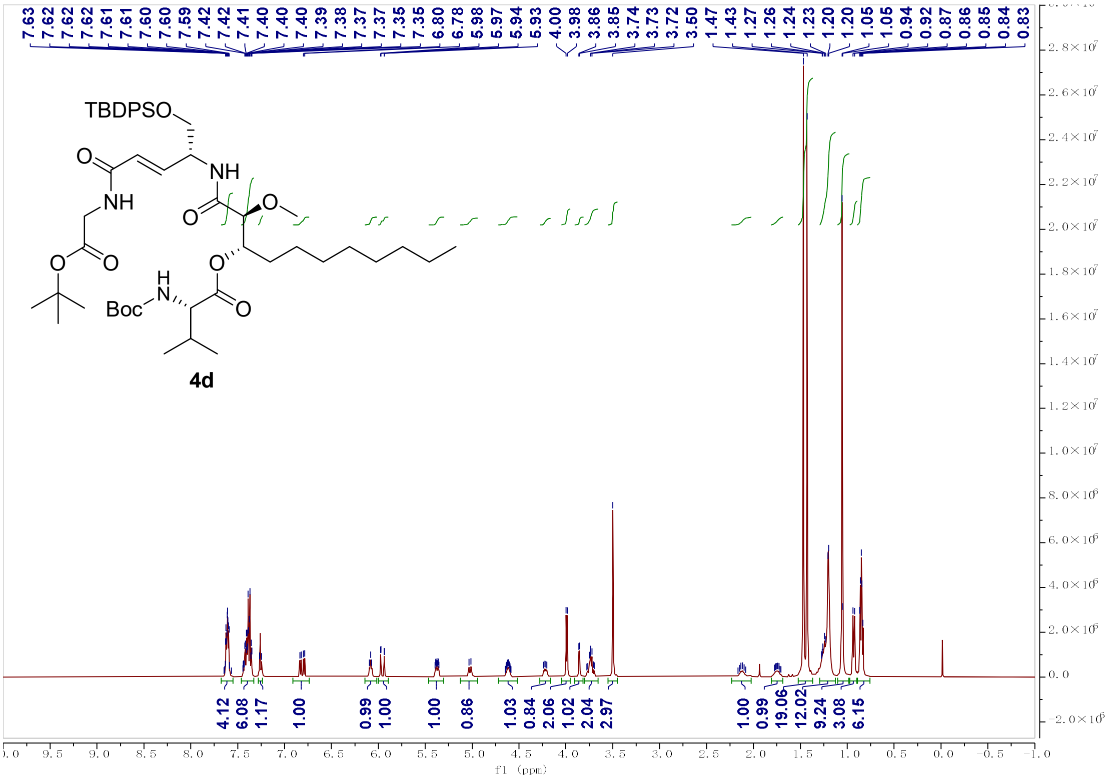


$^{13}C$ NMR spectrum of **4d**

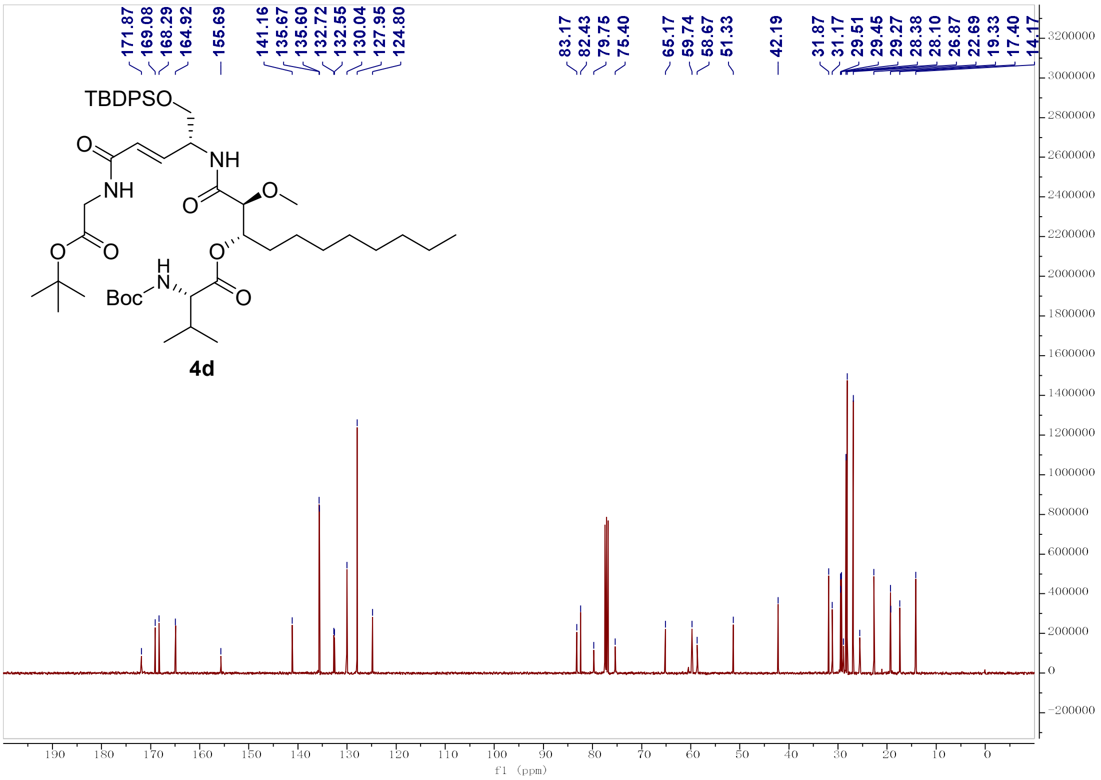

$^{1}$H NMR spectrum of **4e**

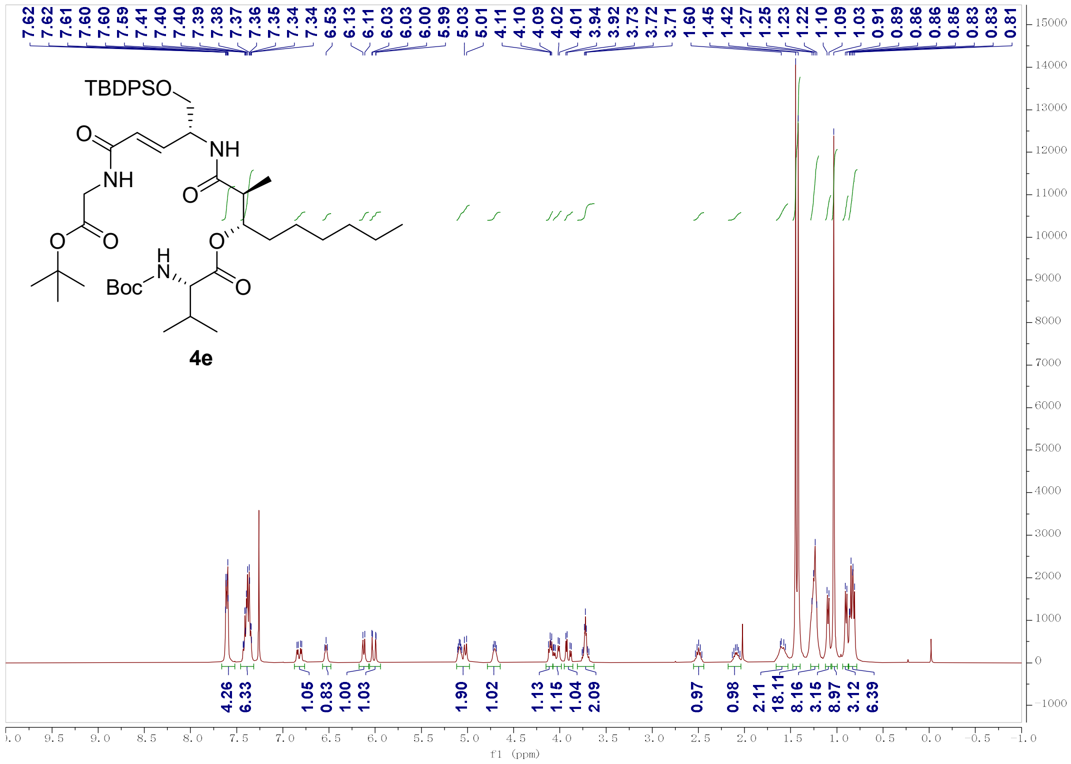

$^{13}$C NMR spectrum of **4e**

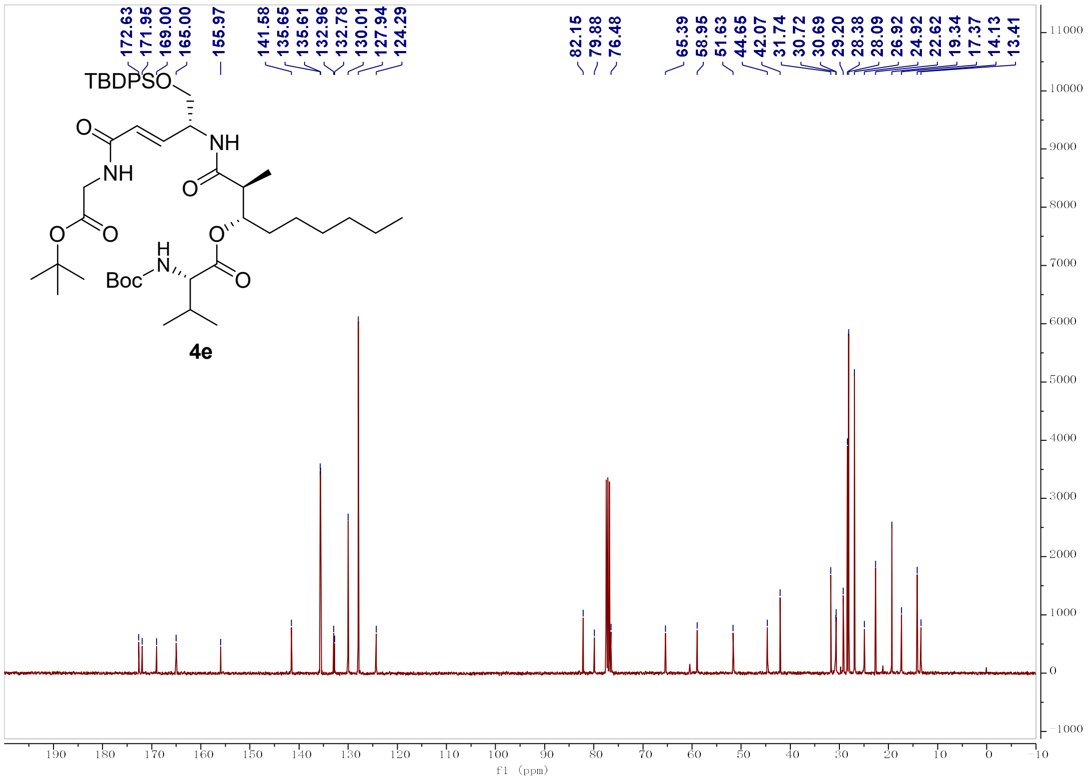

$^{1}H$ NMR spectrum of **4f**

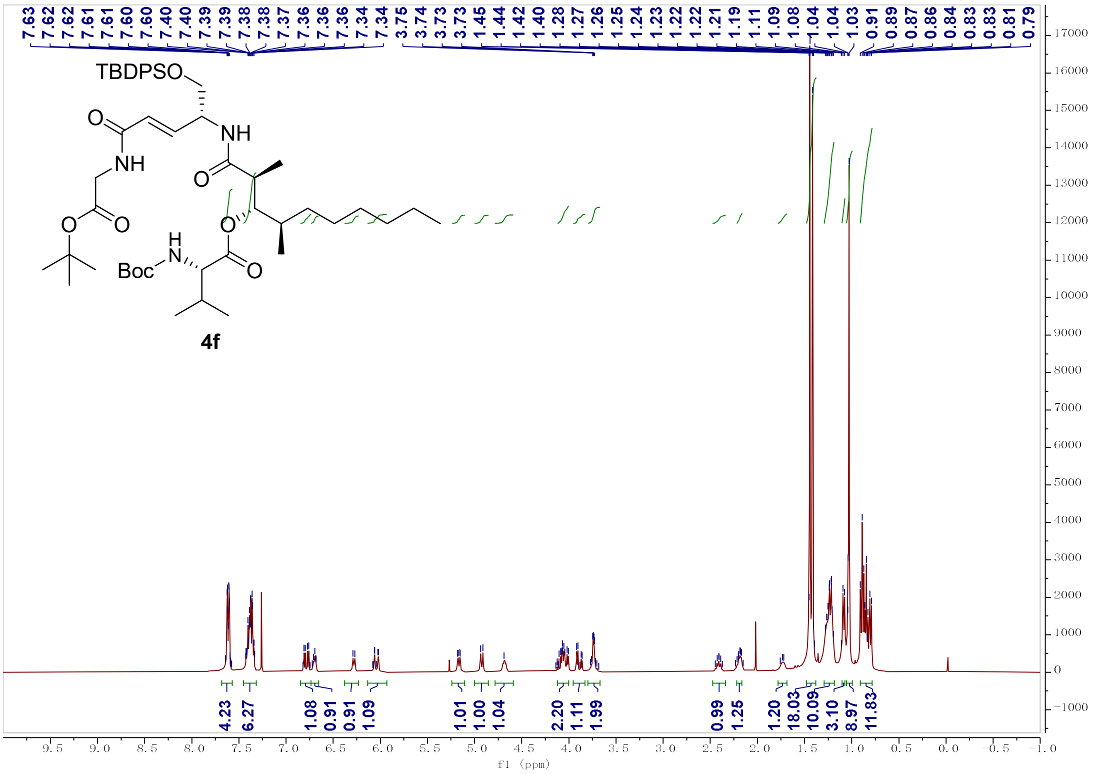

$^{13}C$ NMR spectrum of **4f**

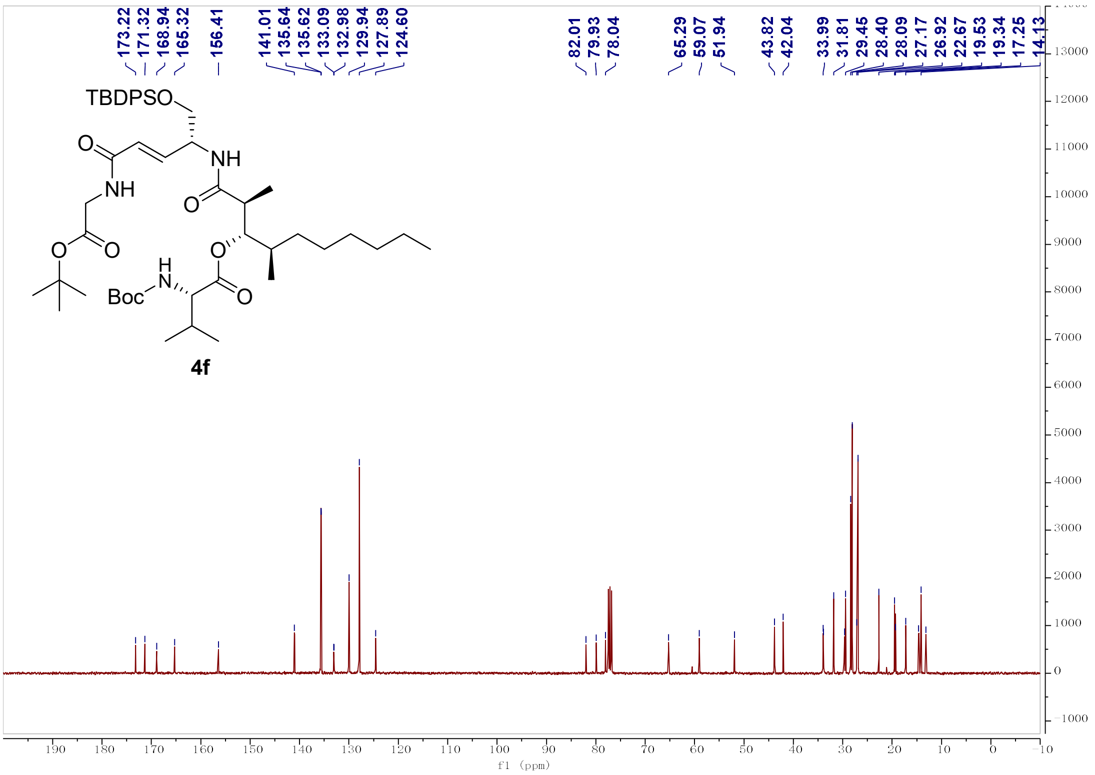

$^1$H NMR spectrum of **4g**

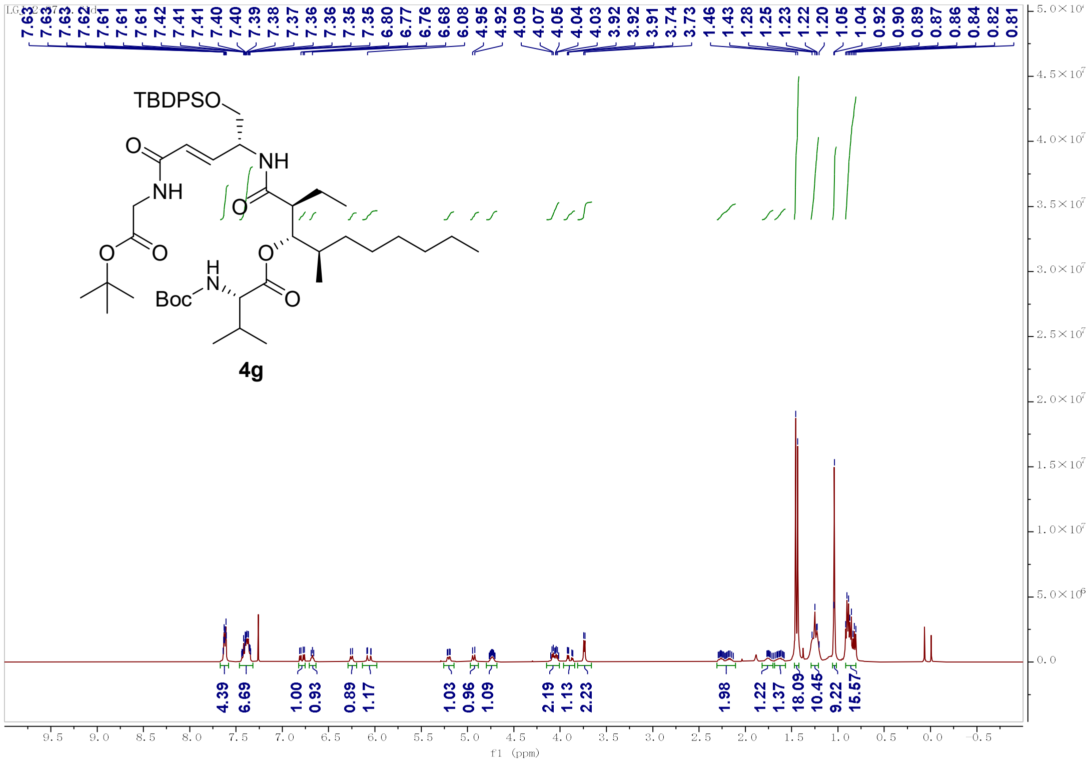


$^{13}$C NMR spectrum of **4g**

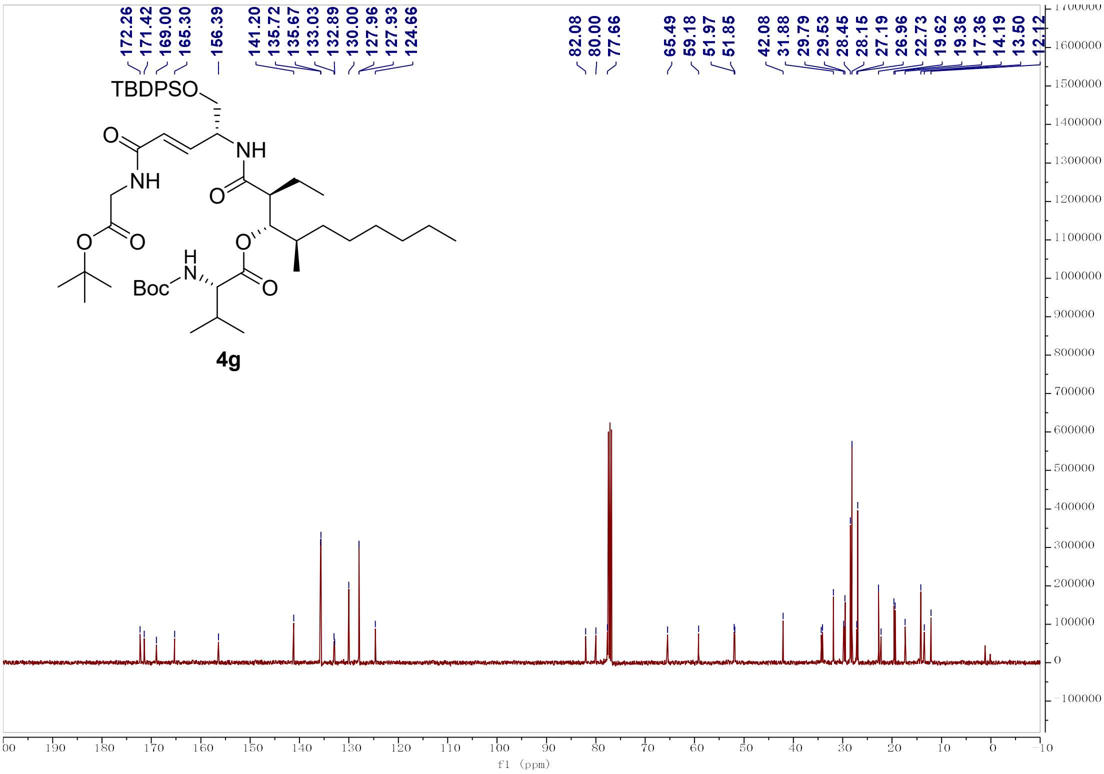

$^{1}H$ NMR spectrum of **3a**

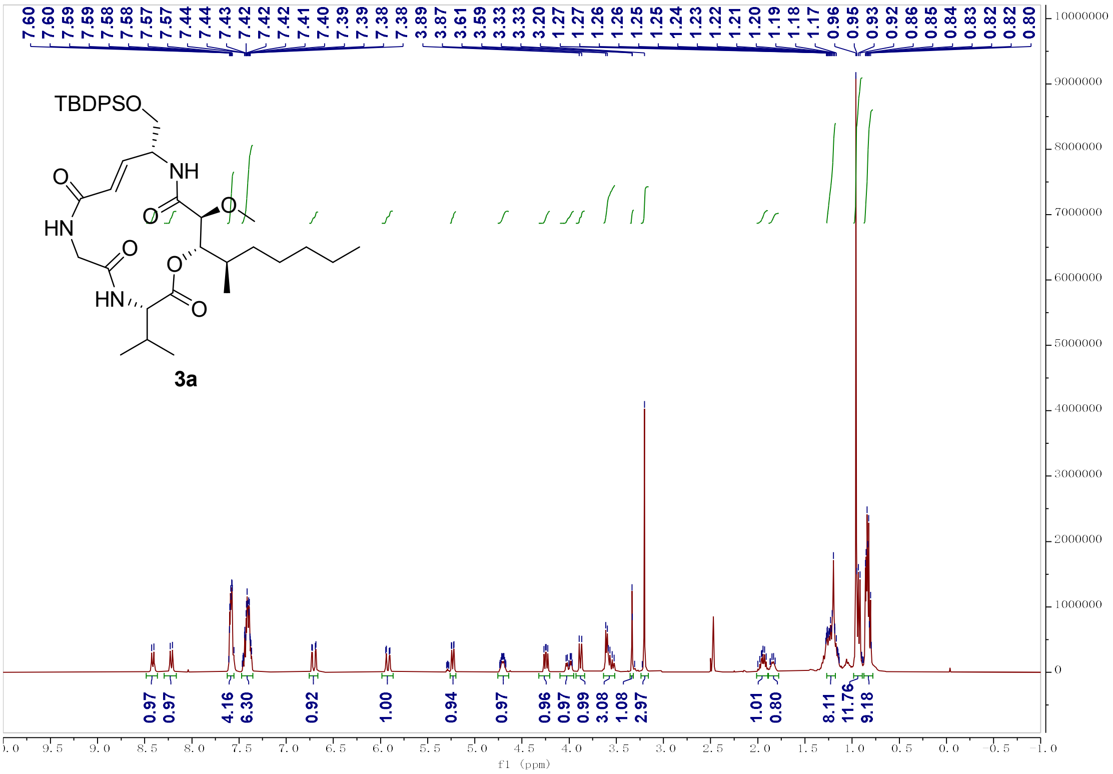

$^{13}C$ NMR spectrum of **3a**

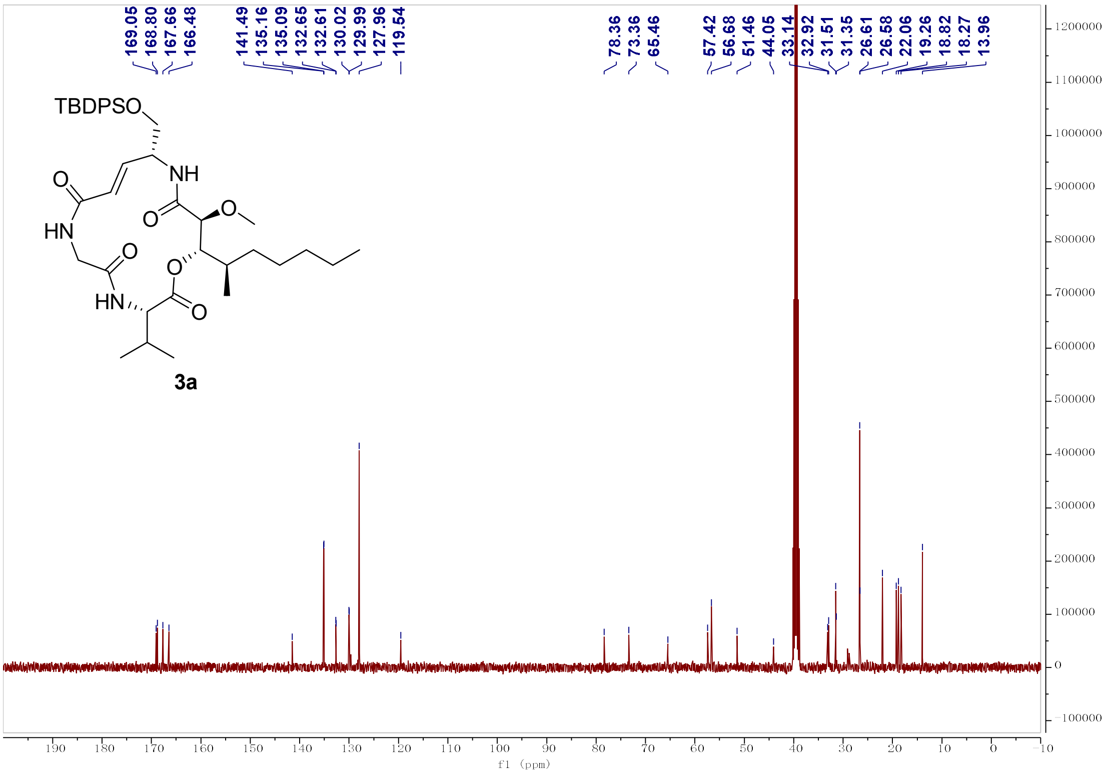

$^{1}$H NMR spectrum of **3b**

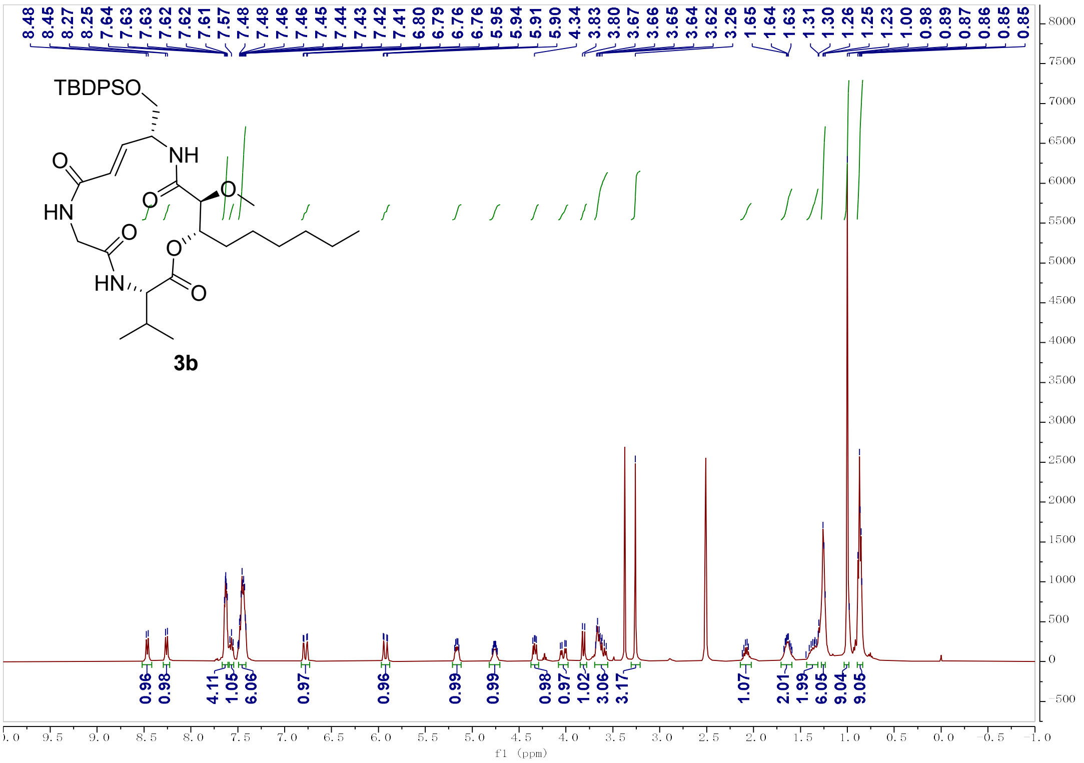


$^{13}$C NMR spectrum of **3b**

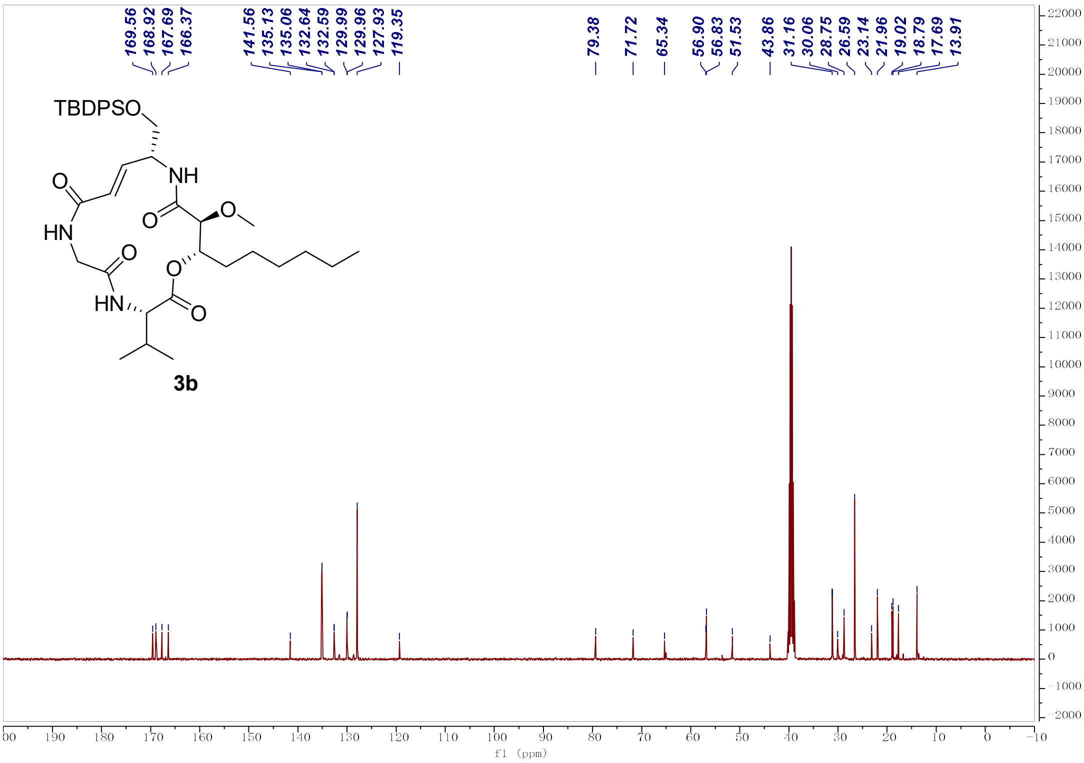

$^{1}$H NMR spectrum of **3c**

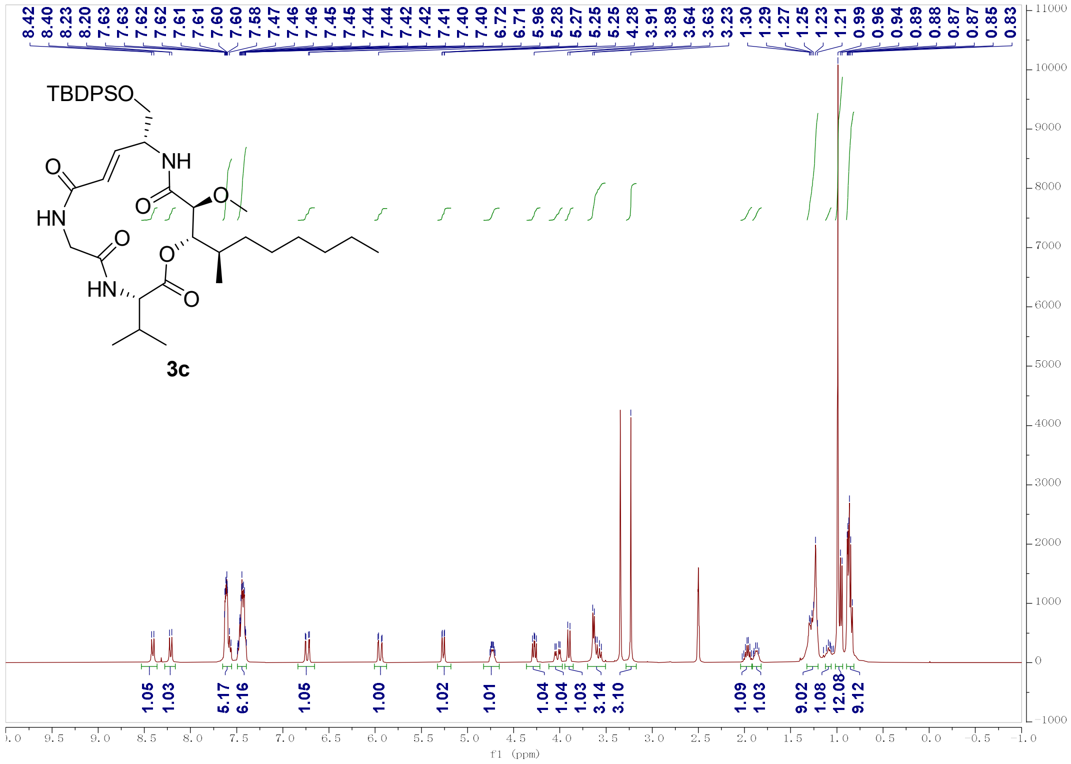

$^{13}$C NMR spectrum of **3c**

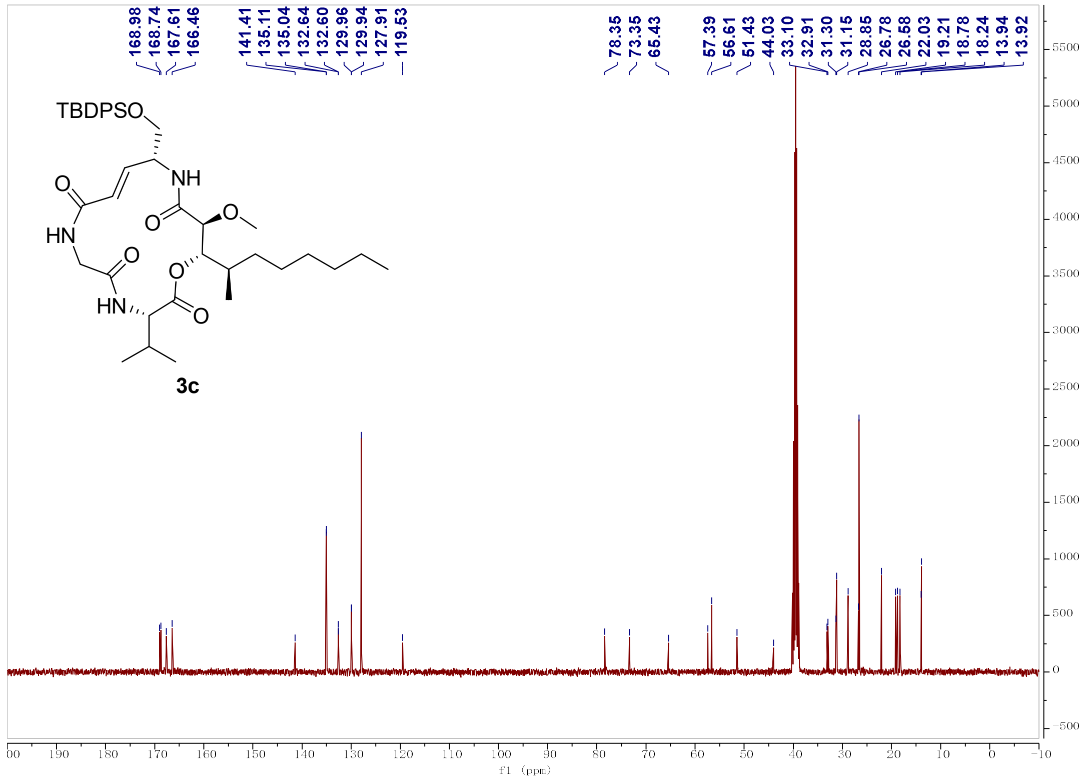

$^{1}$H NMR spectrum of **3d**

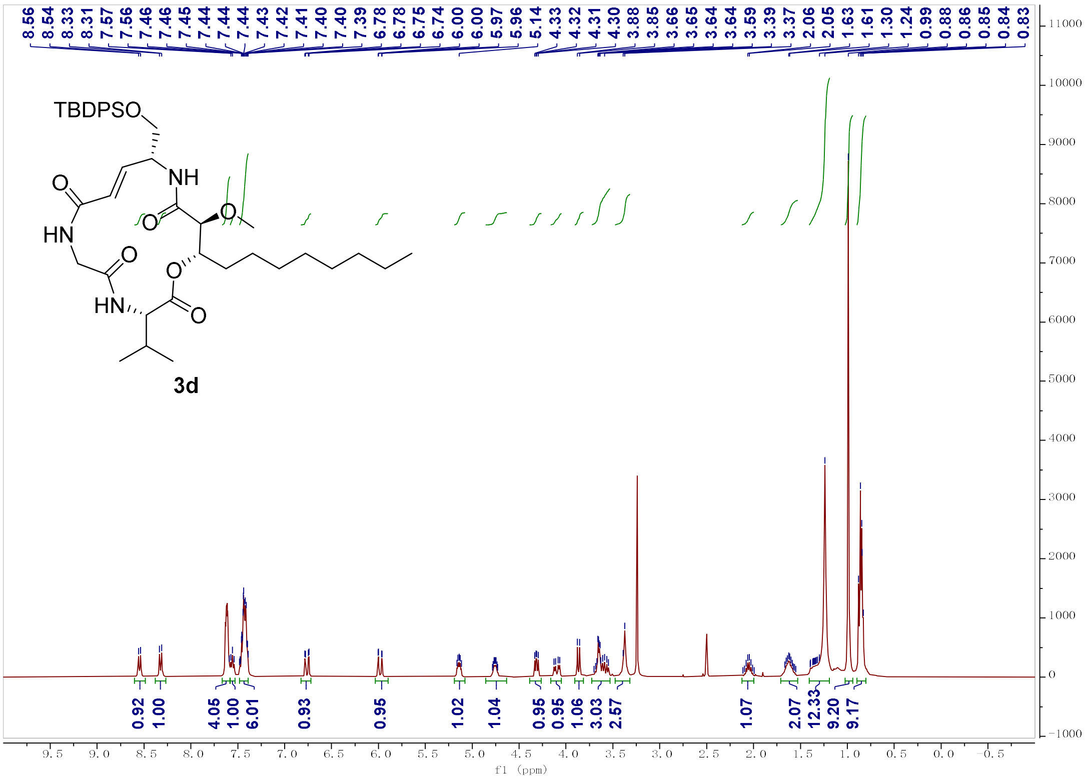

$^{13}$C NMR spectrum of **3d**

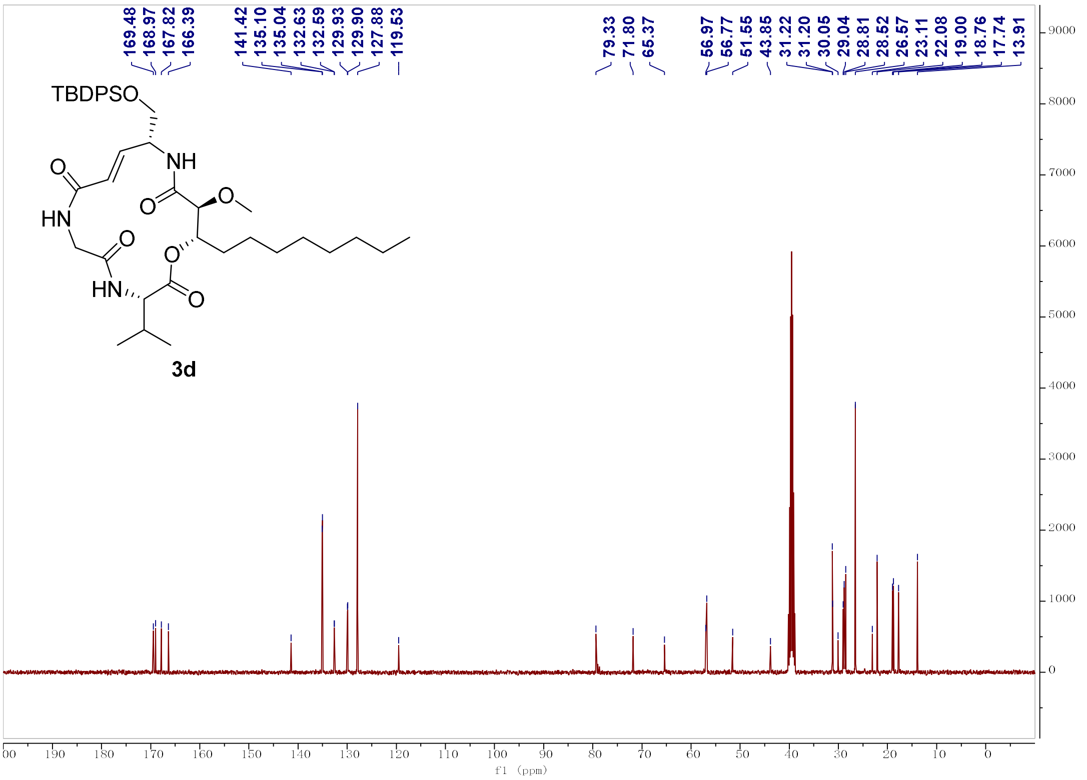

$^{1}$H NMR spectrum of **3e**

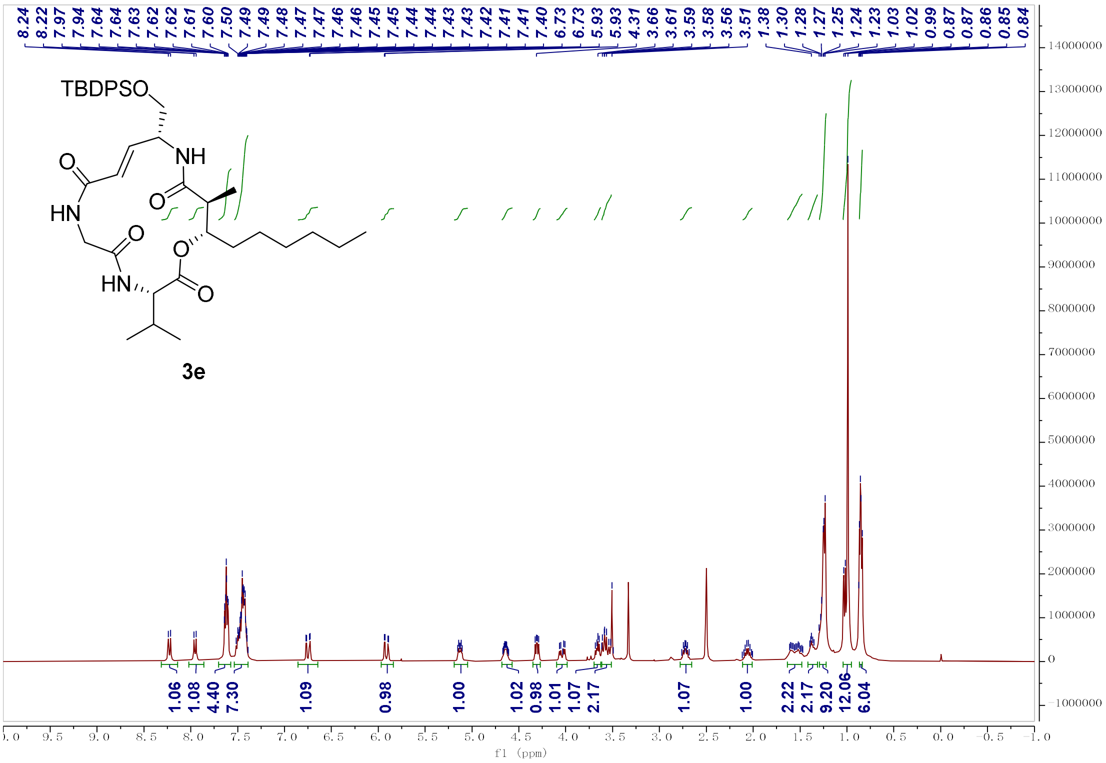

$^{13}$C NMR spectrum of **3e**

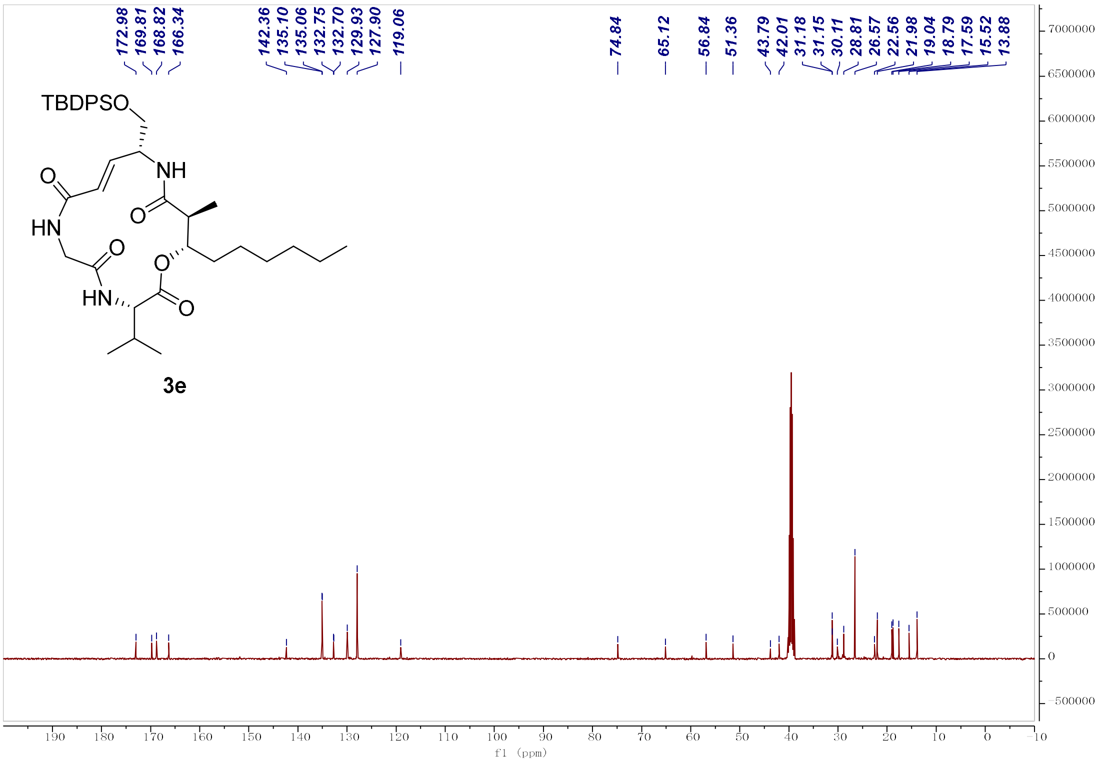

$^{1}$H NMR spectrum of **3f**

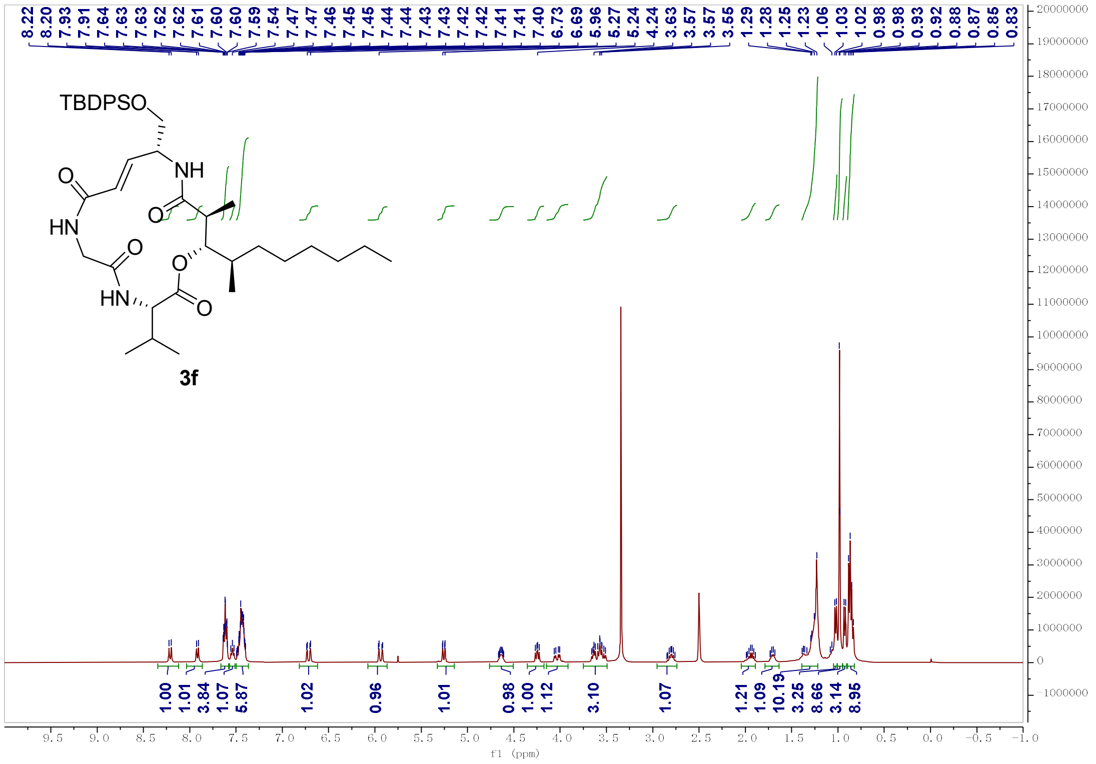

$^{13}$C NMR spectrum of **3f**

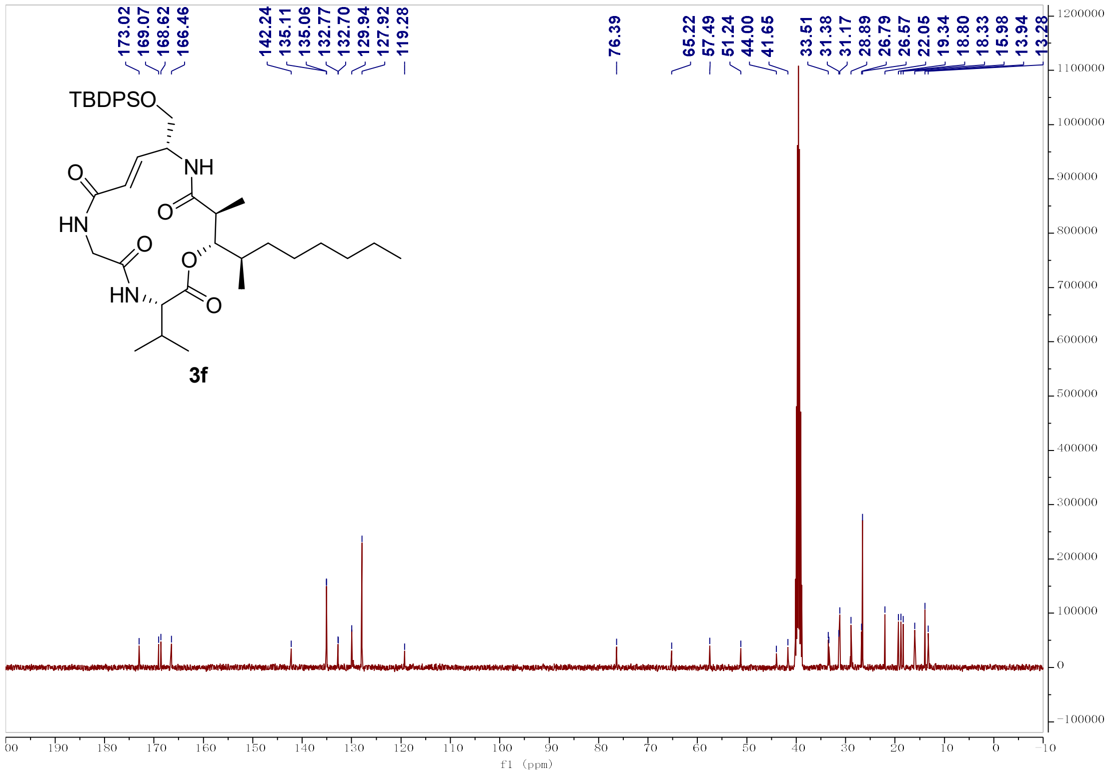

$^{1}$H NMR spectrum of **3g**

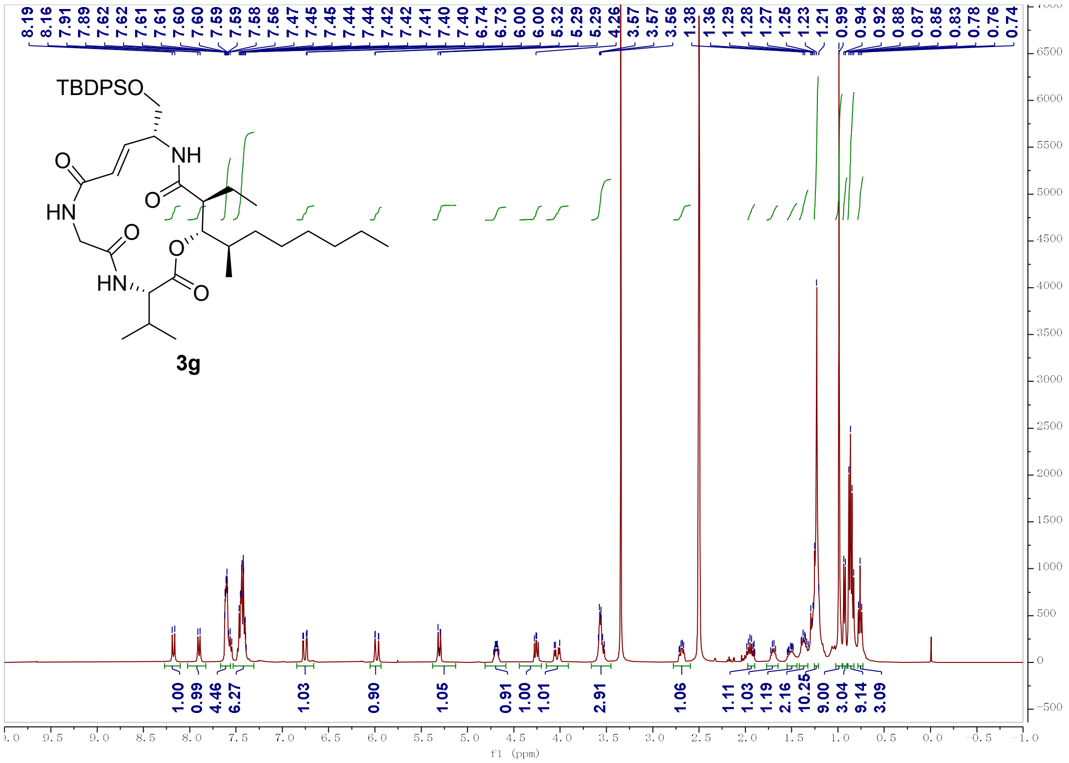


$^{13}$C NMR spectrum of **3g**

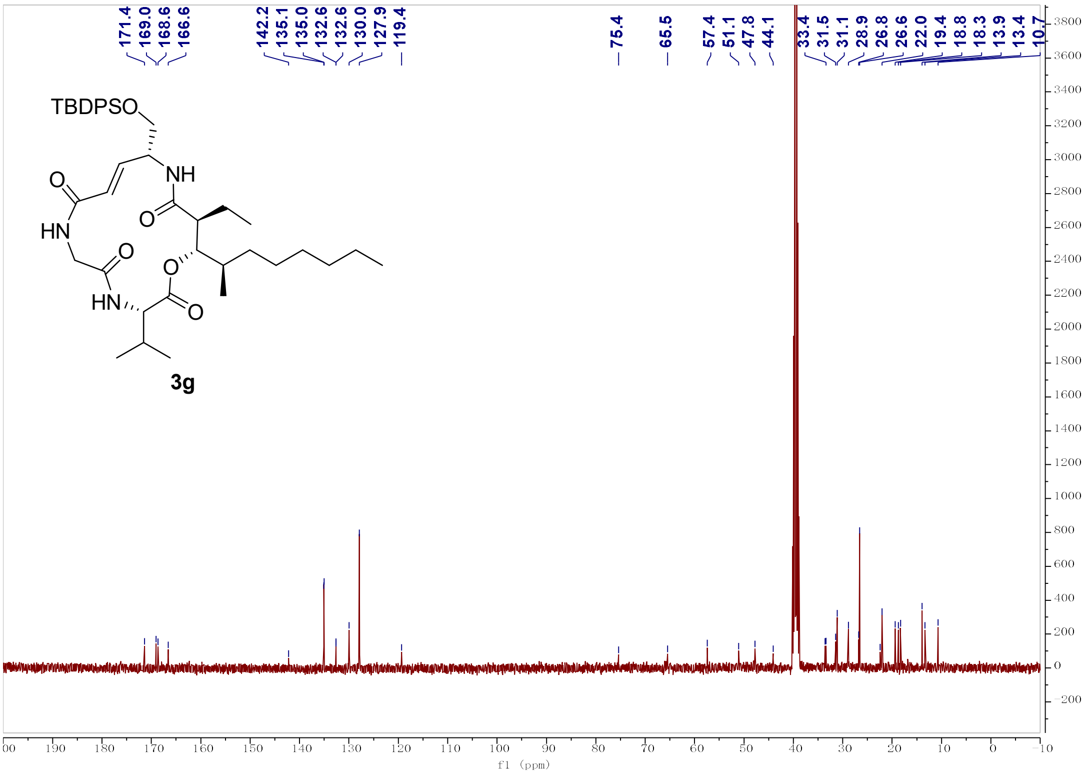

$^{1}H$ NMR spectrum of **1a**

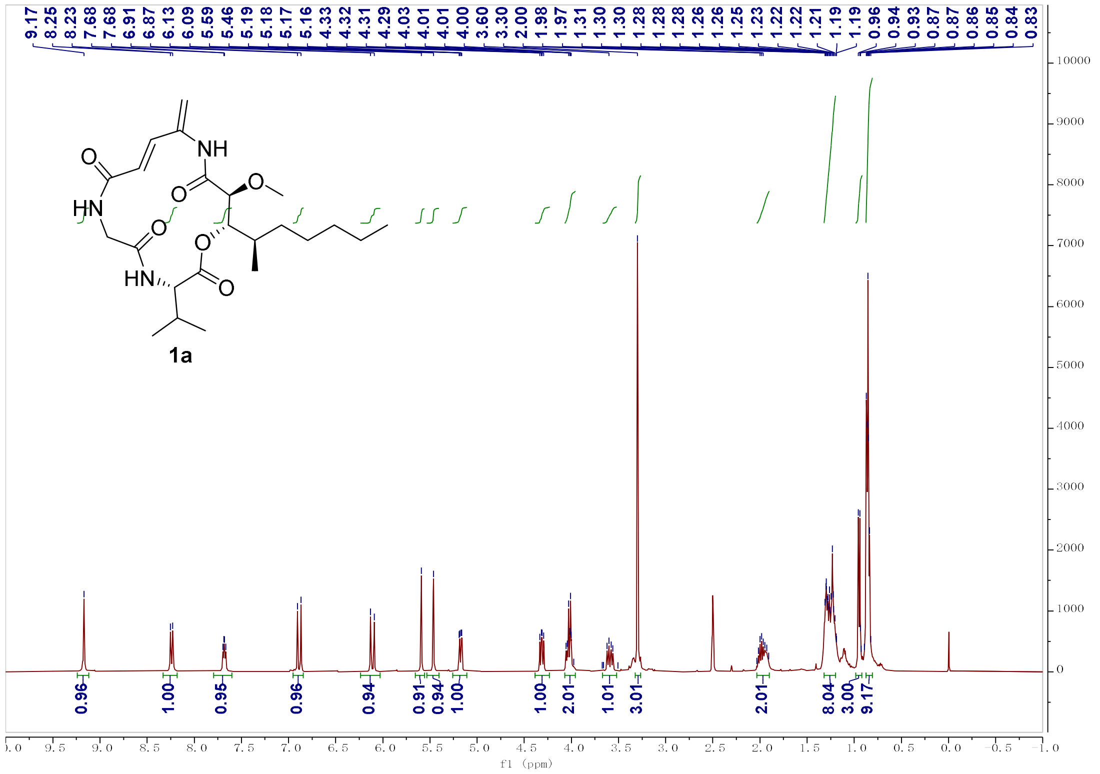

$^{13}C$ NMR spectrum of **1a**

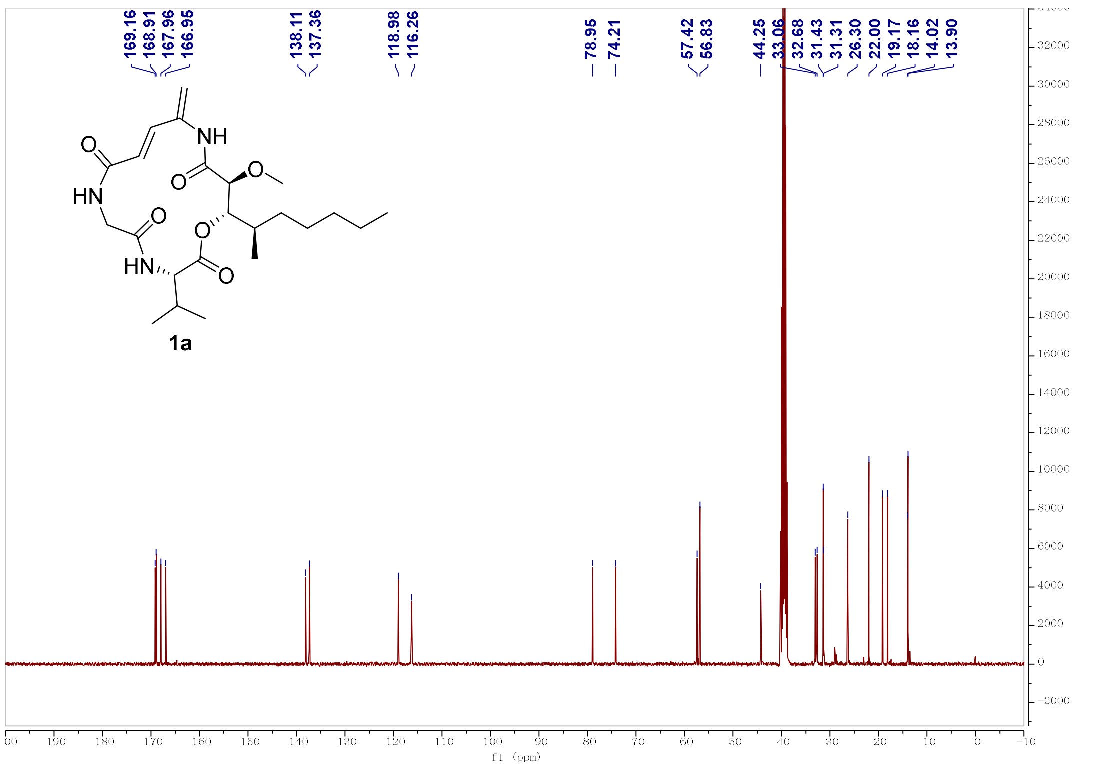

$^{1}$H NMR spectrum of **1b**

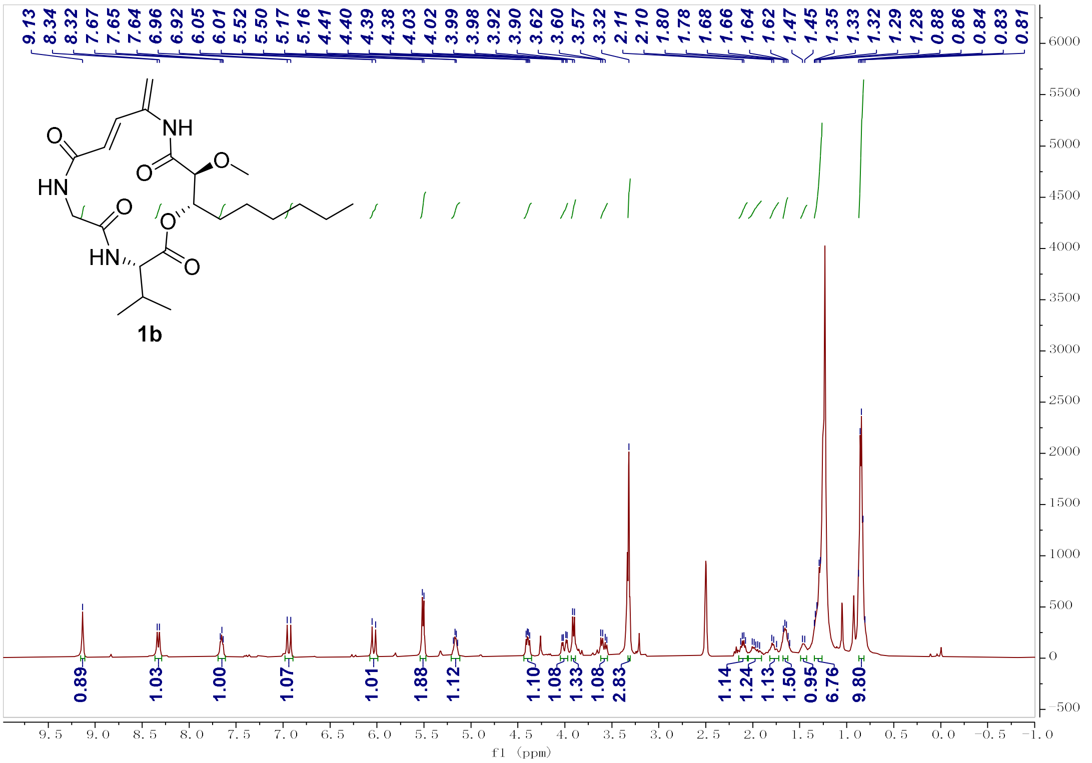

$^{13}$C NMR spectrum of **1b**

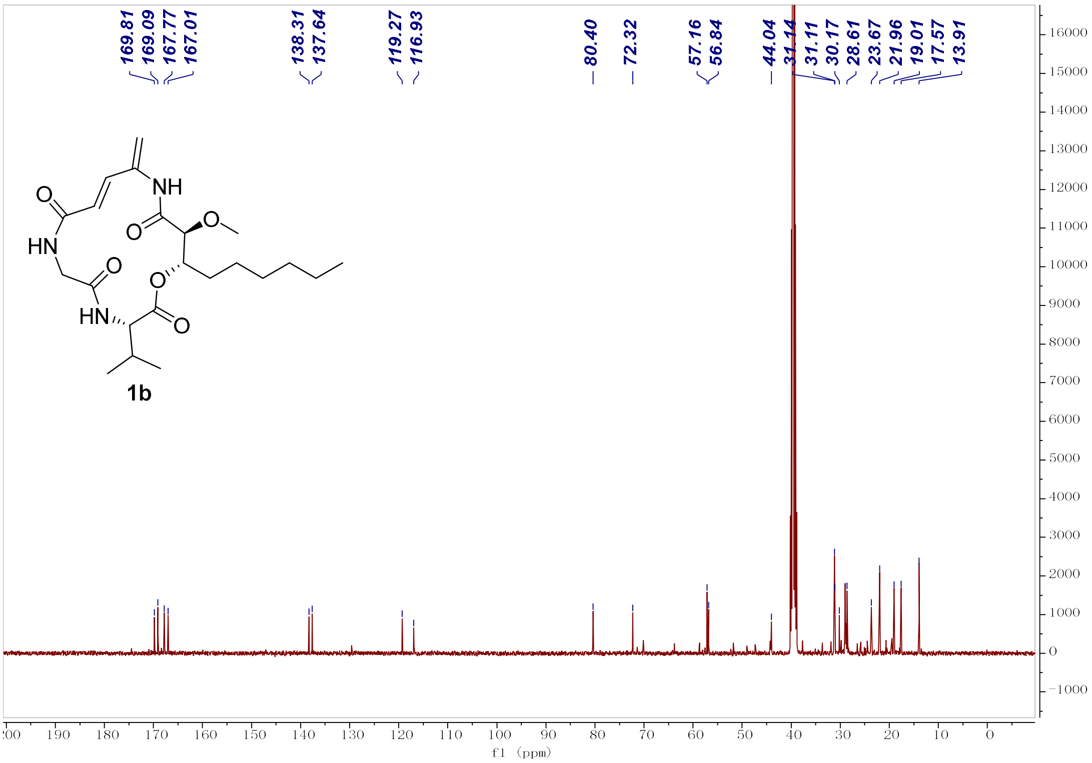

$^{1}$H NMR spectrum of **1c**

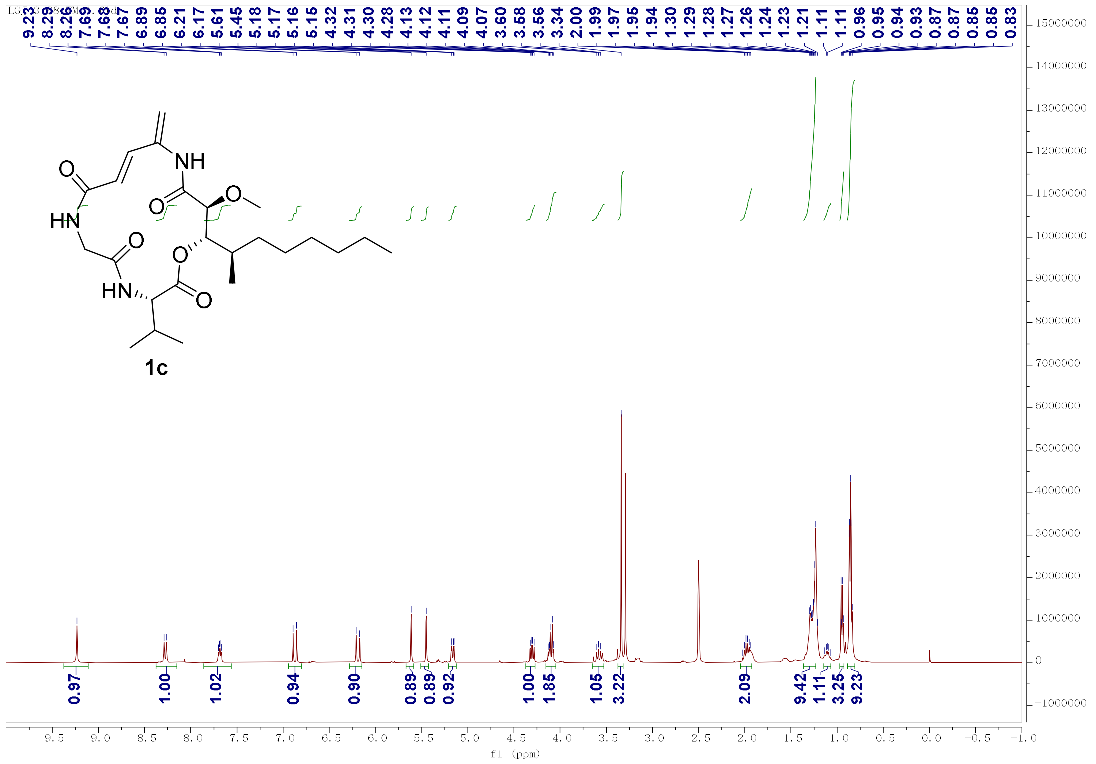

$^{13}$C NMR spectrum of **1c**

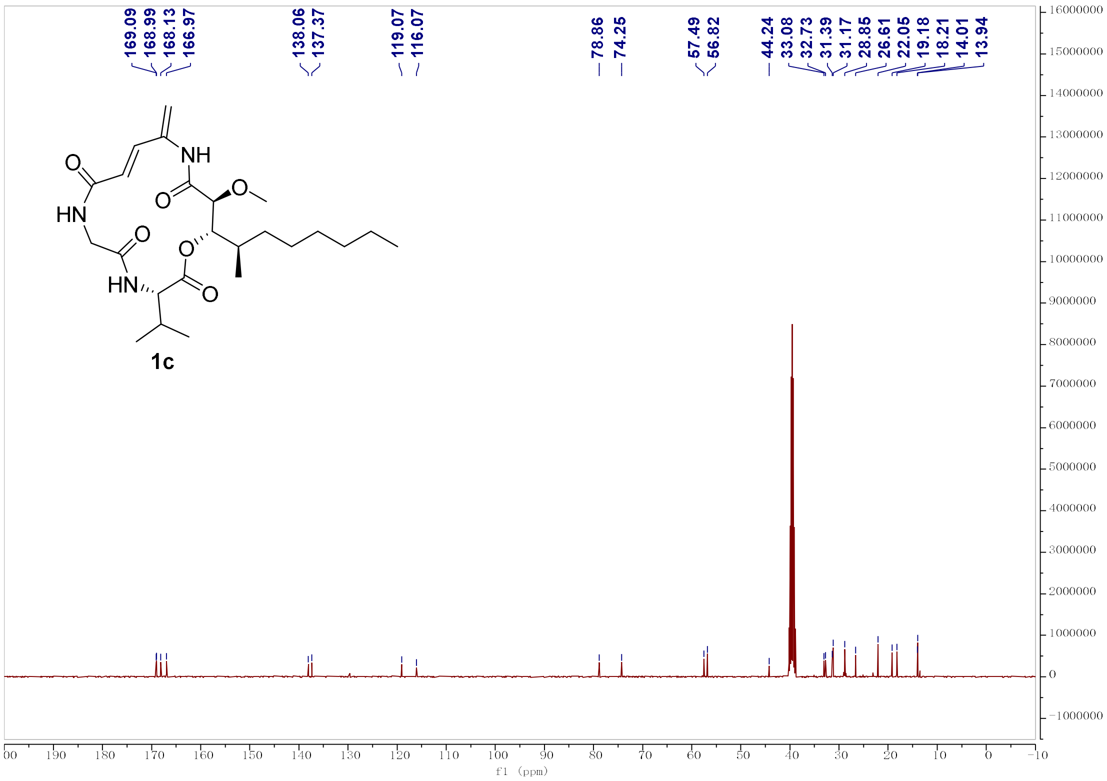

$^{1}$H NMR spectrum of **1d**

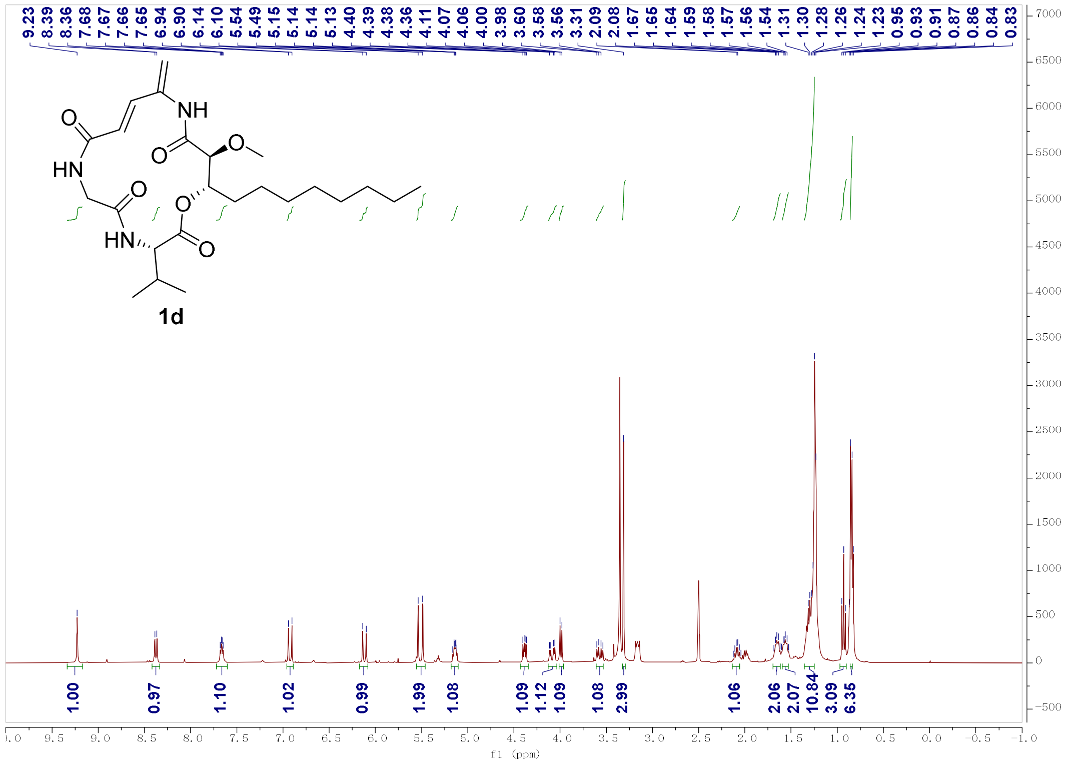


$^{13}$C NMR spectrum of **1d**

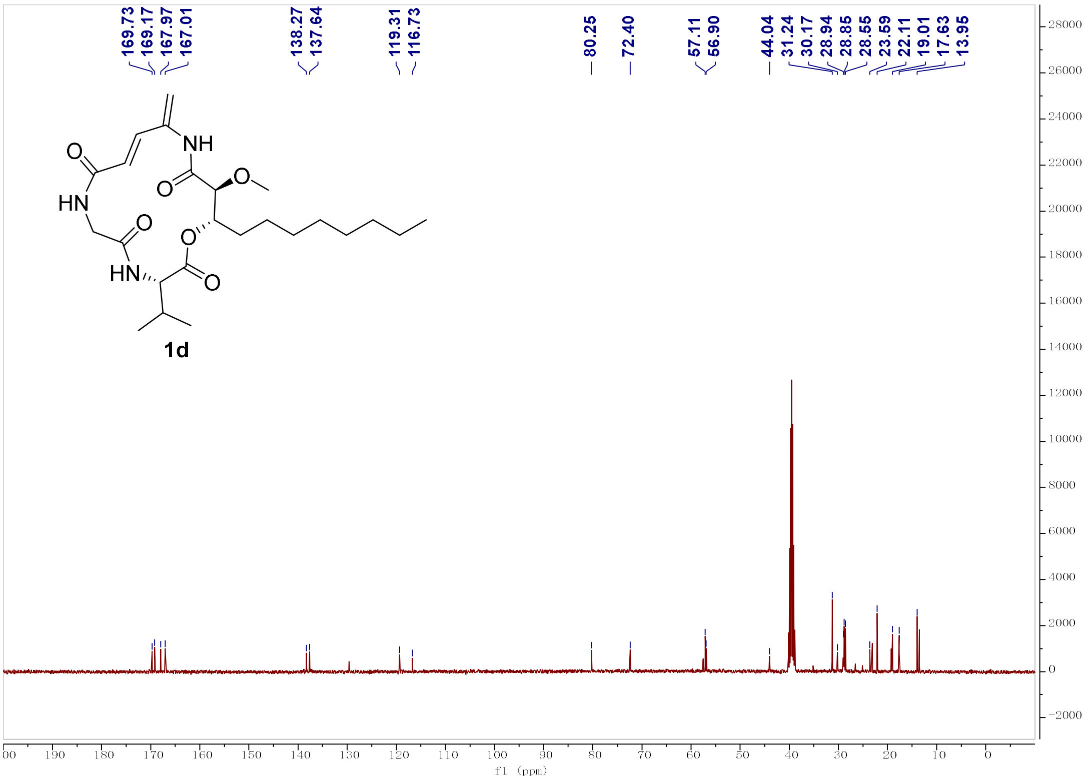

$^{1}$H NMR spectrum of **1e**

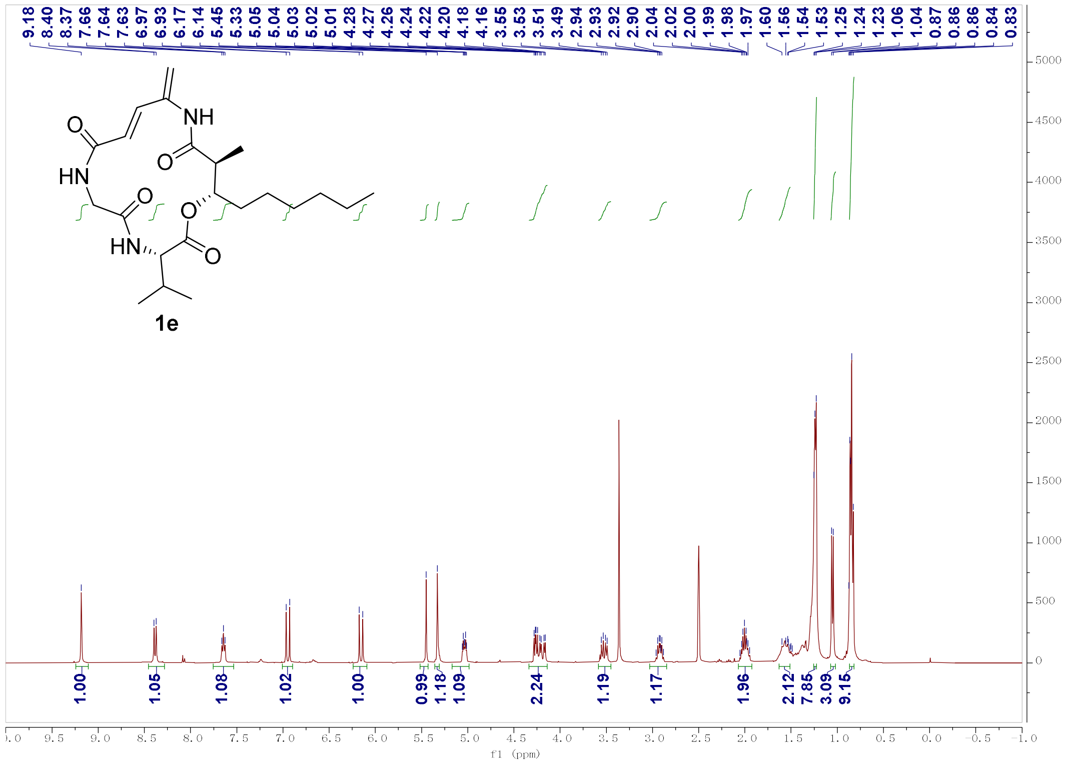

$^{13}$C NMR spectrum of **1e**

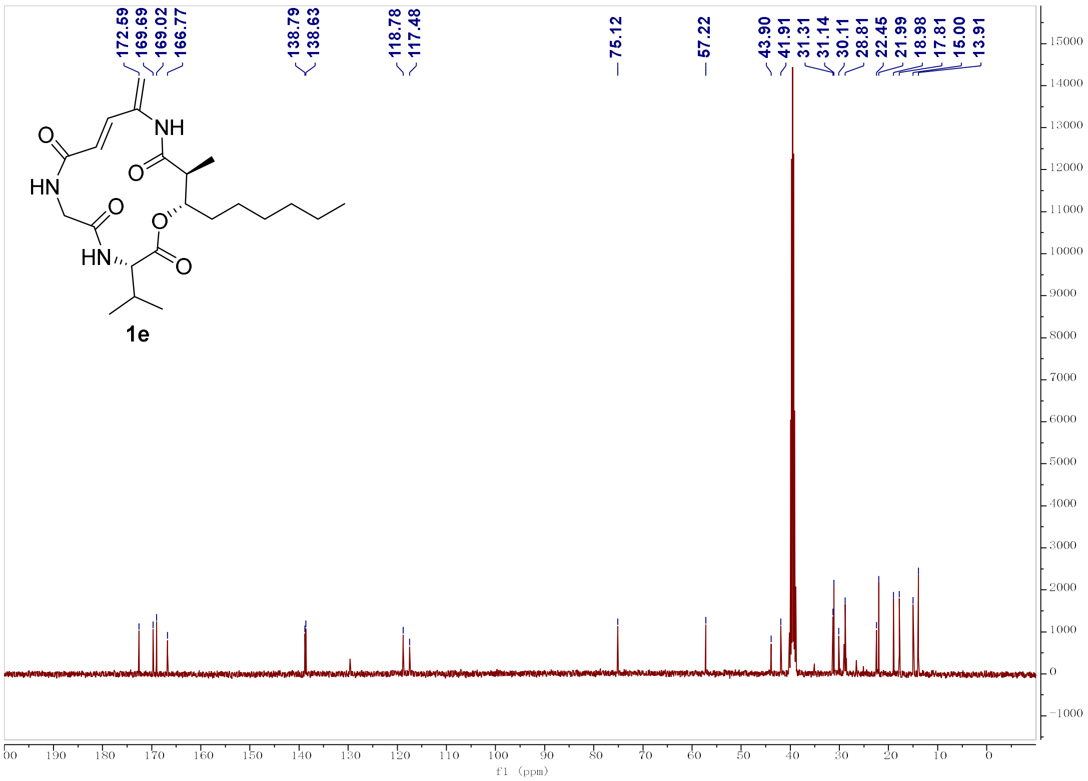

$^{1}$H NMR spectrum of **1f**

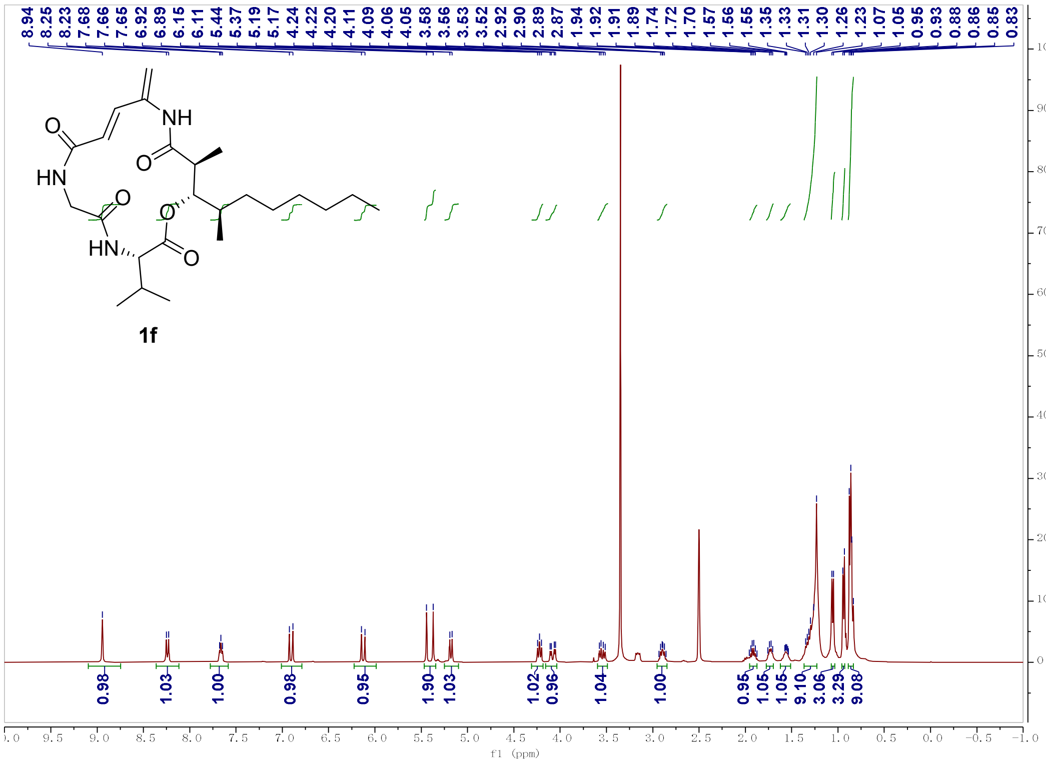

$^{13}$C NMR spectrum of **1f**

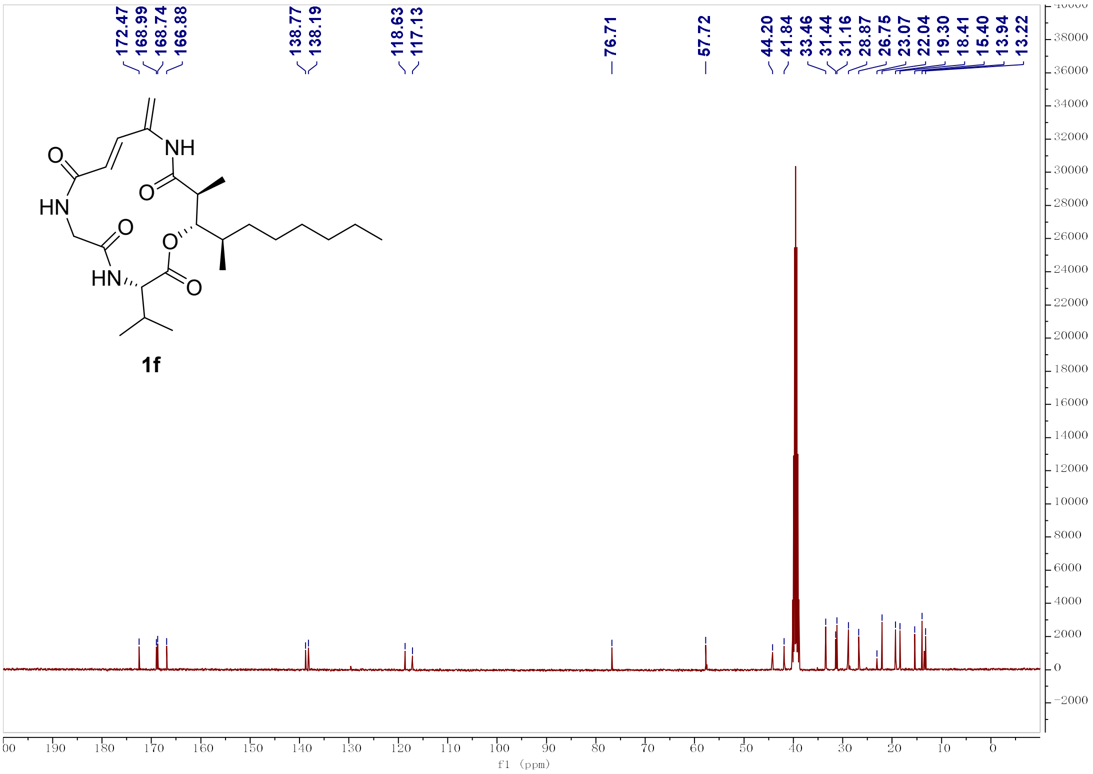

$^{1}$H NMR spectrum of **1g**

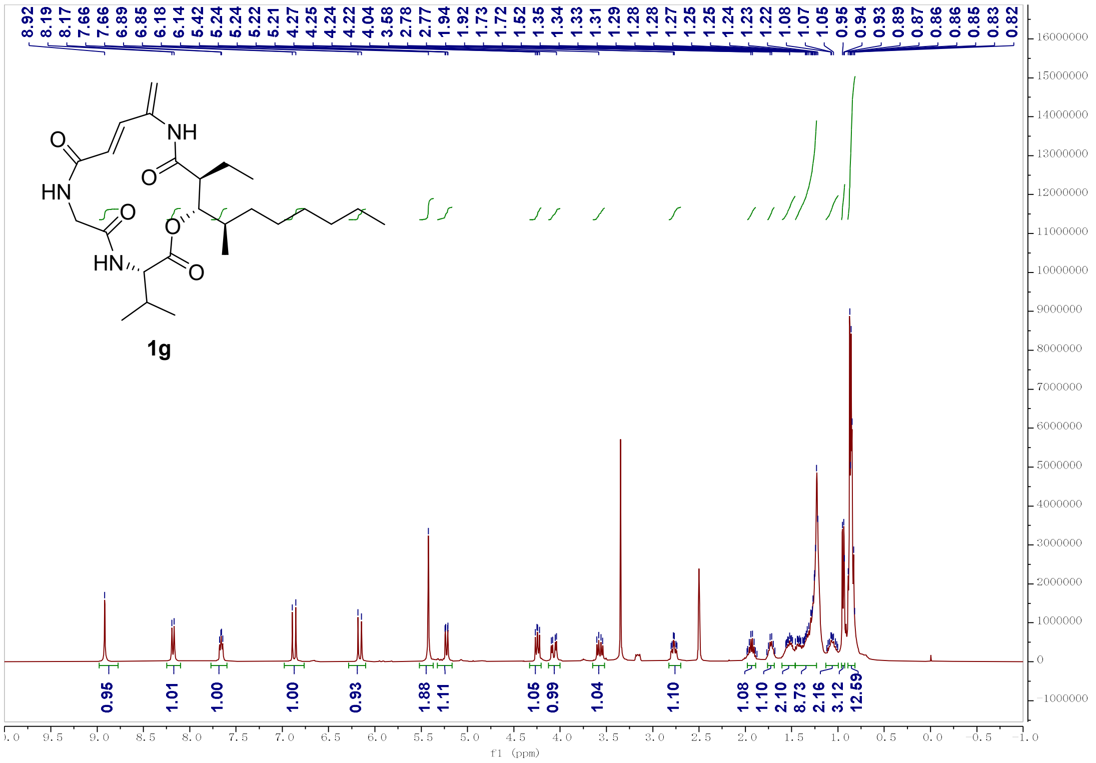

$^{13}$C NMR spectrum of **1g**

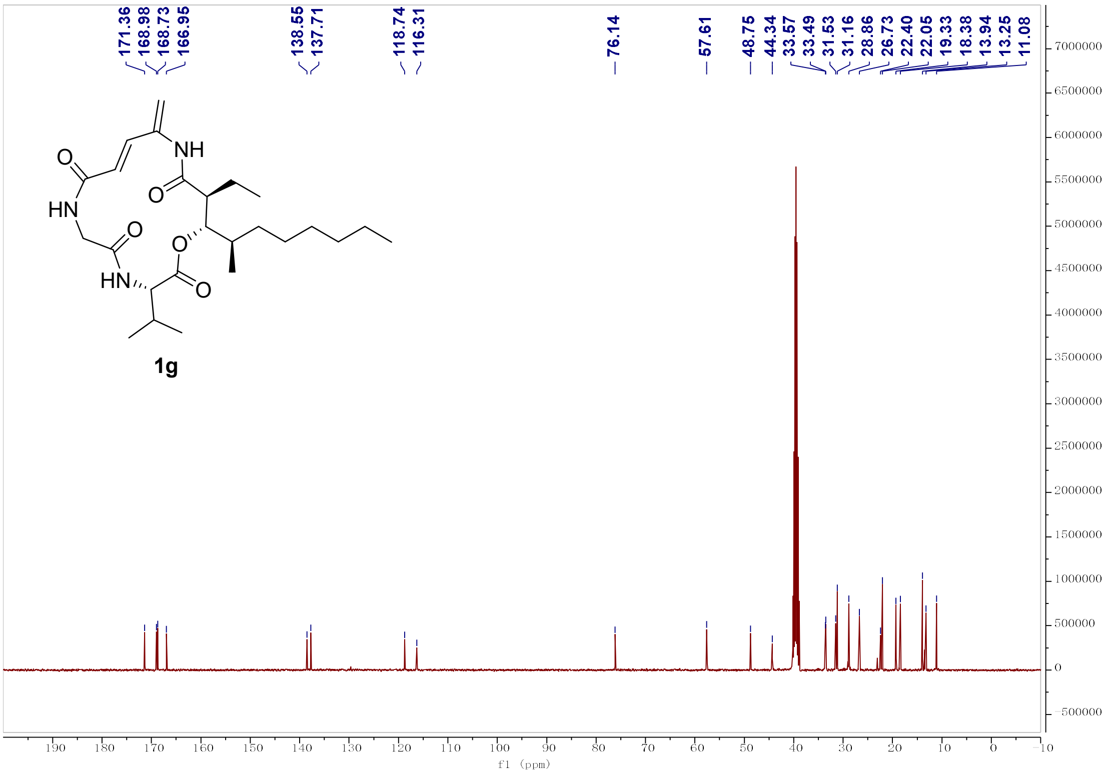

$^{1}$H NMR spectrum of **6h**

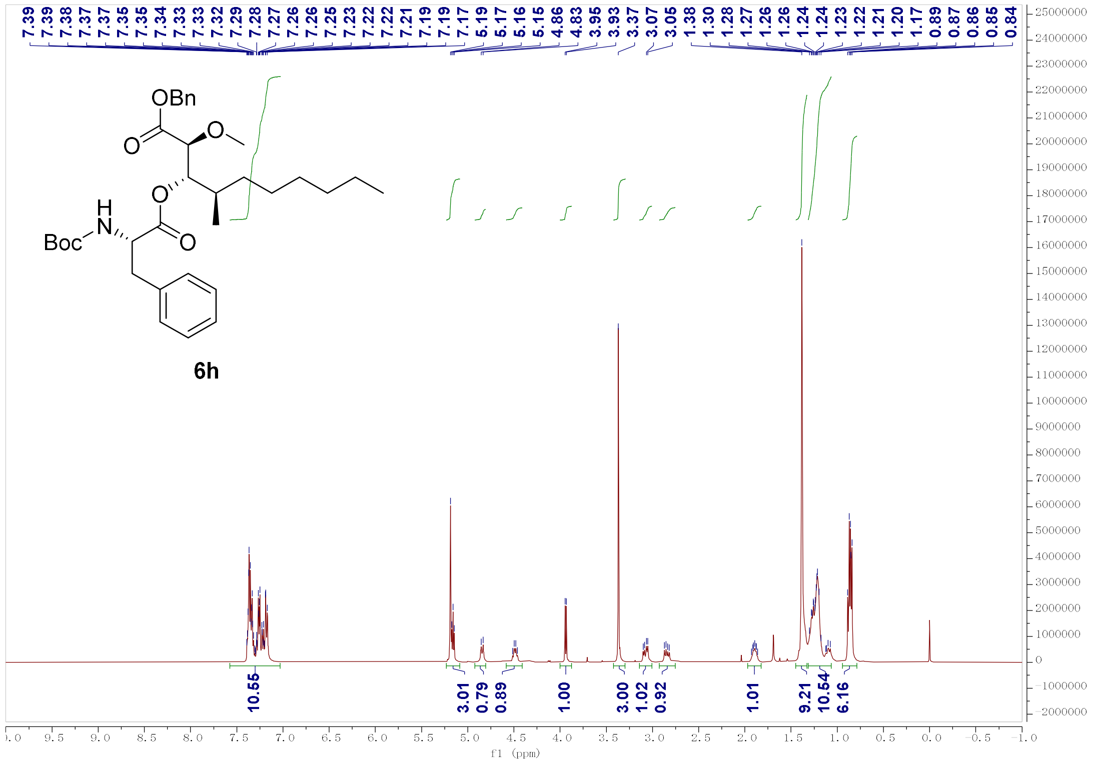

$^{13}$C NMR spectrum of **6h**

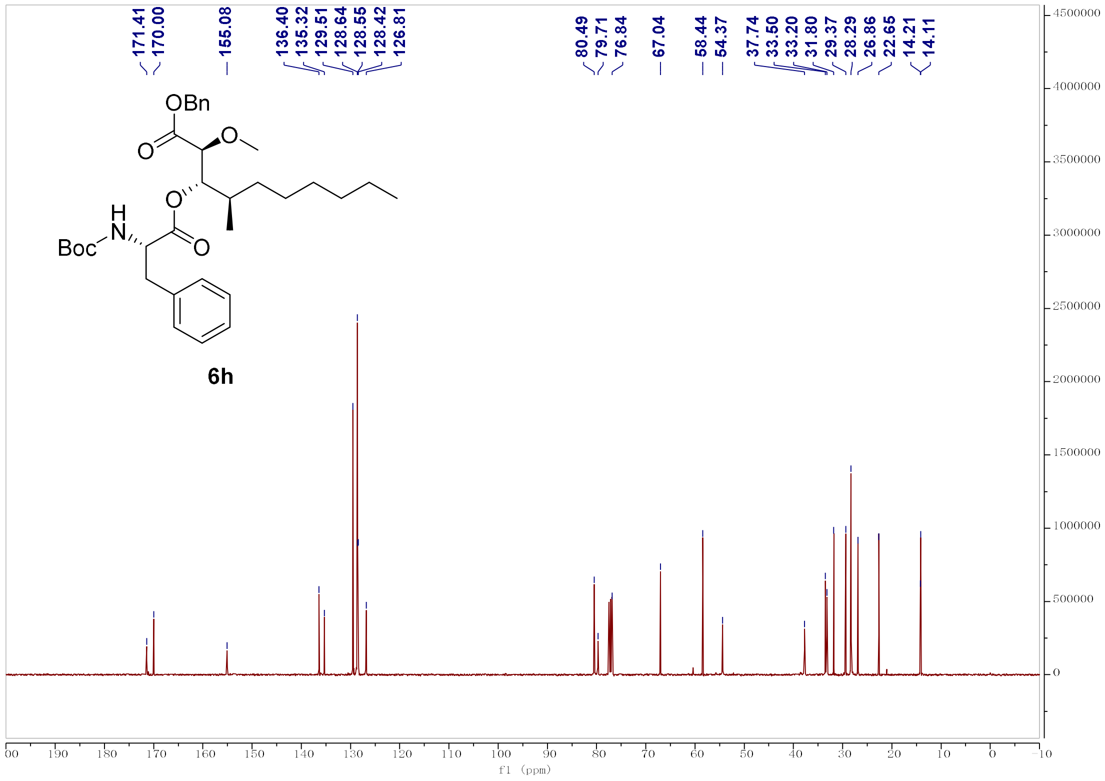

$^{1}$H NMR spectrum of **6i**

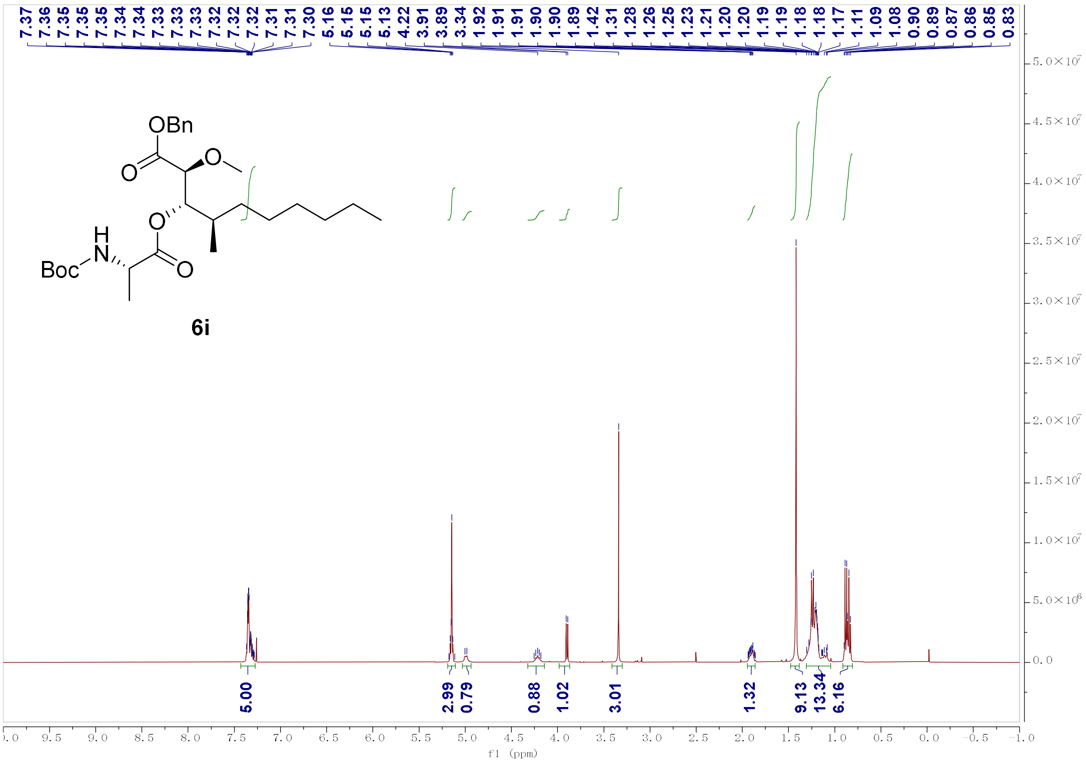

$^{13}$C NMR spectrum of **6i**

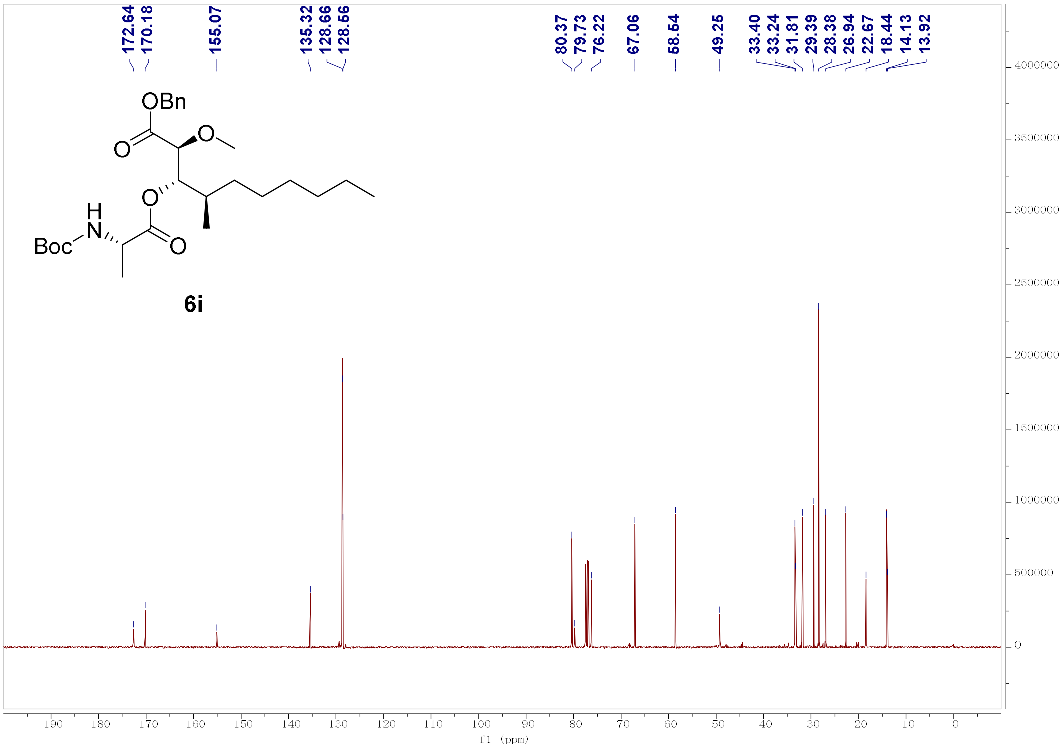

$^{1}$H NMR spectrum of **6j**

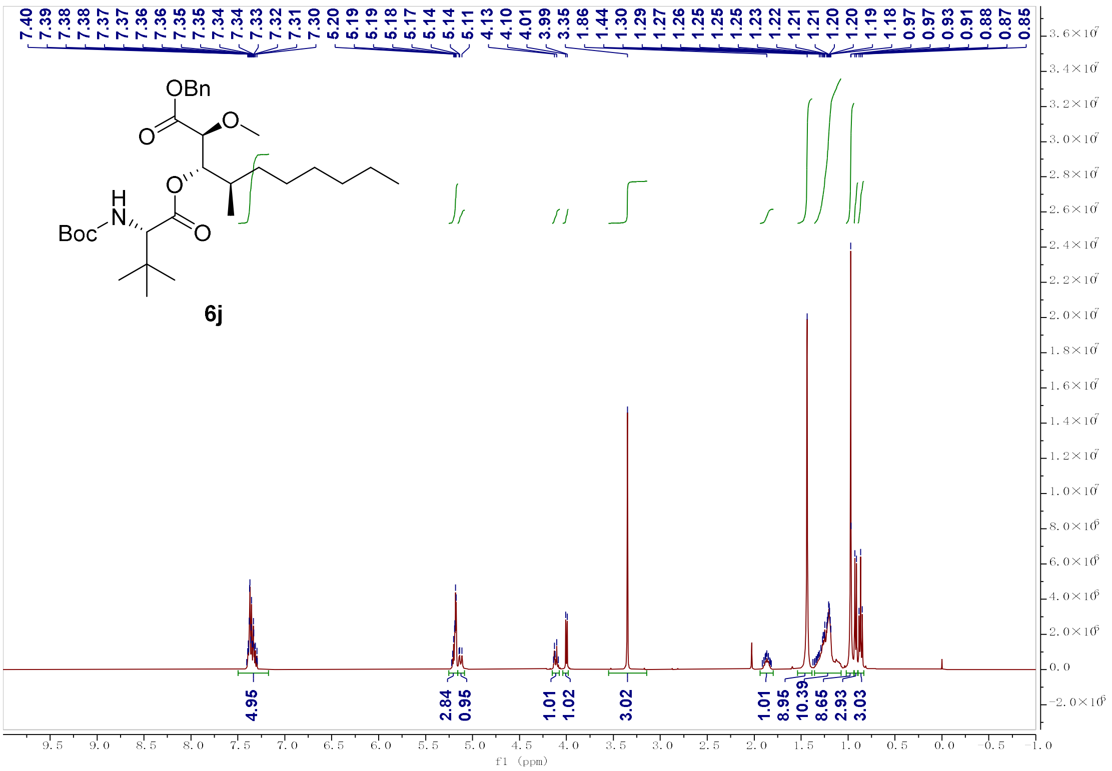

$^{13}$C NMR spectrum of **6j**

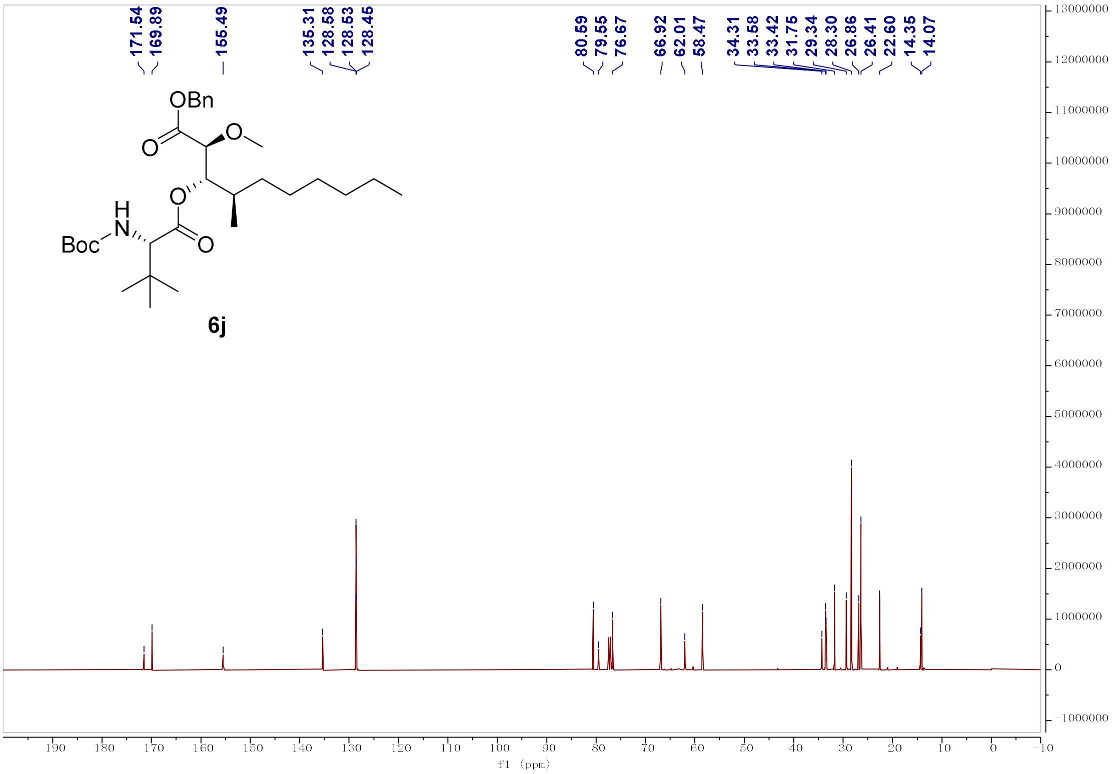

$^{1}$H NMR spectrum of **6k**

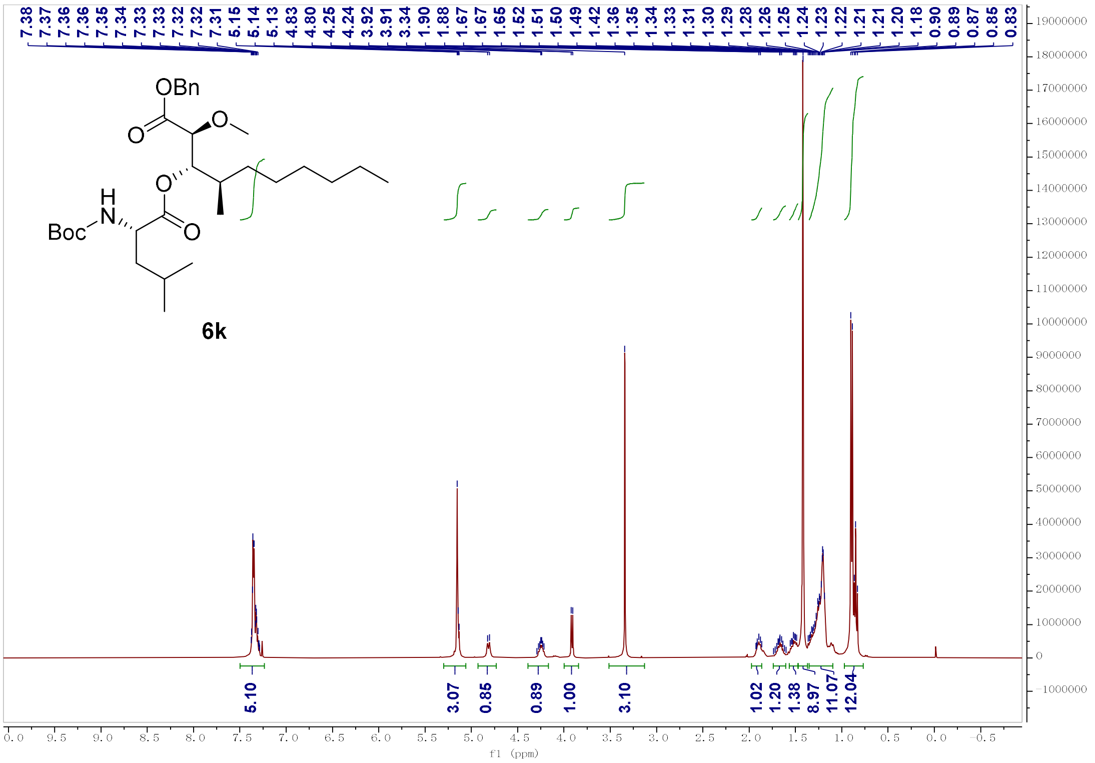


$^{13}$C NMR spectrum of **6k**

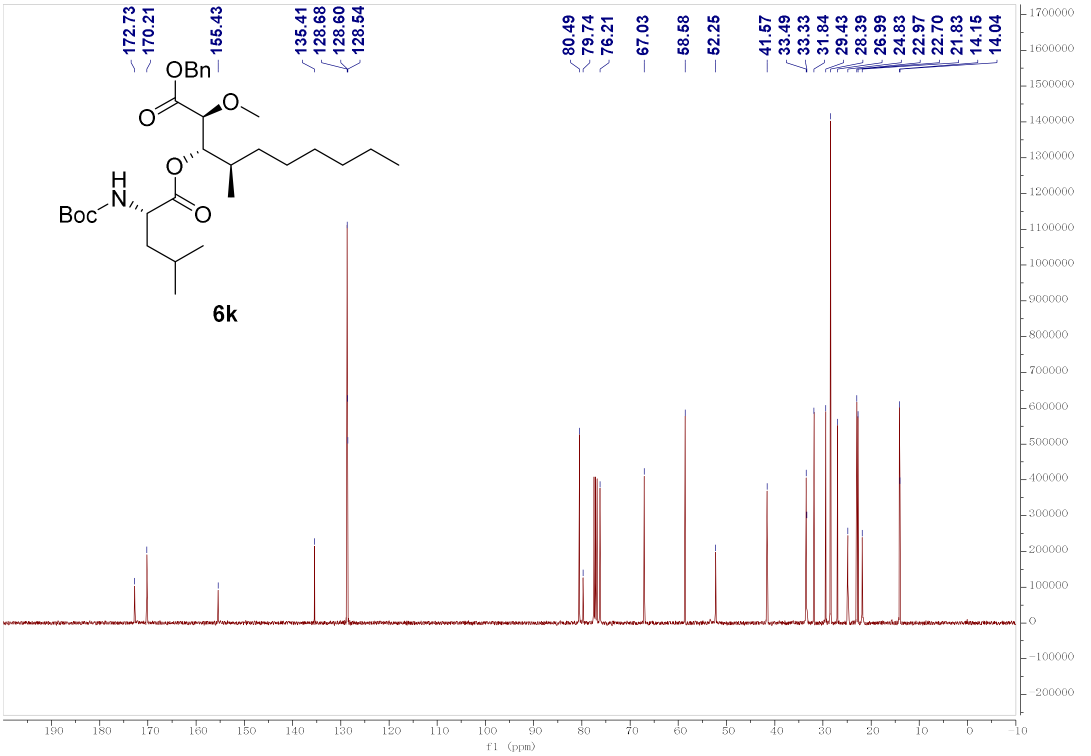

$^{1}$H NMR spectrum of **6l**

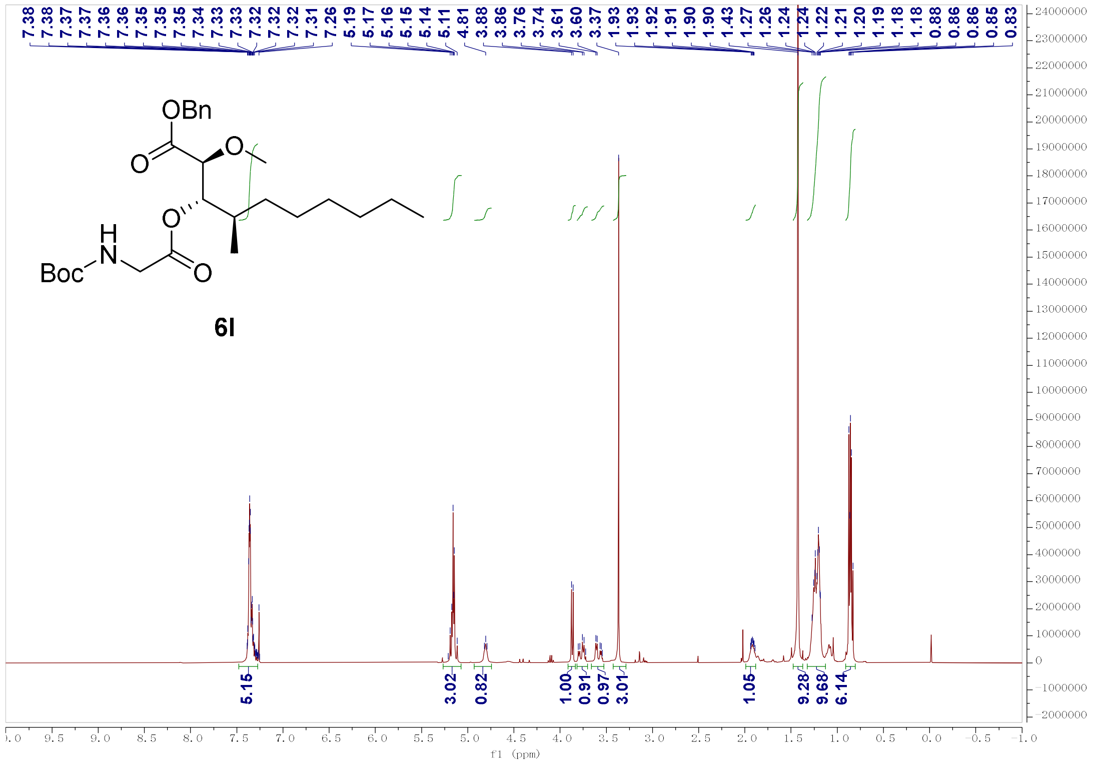

$^{13}$C NMR spectrum of **6l**

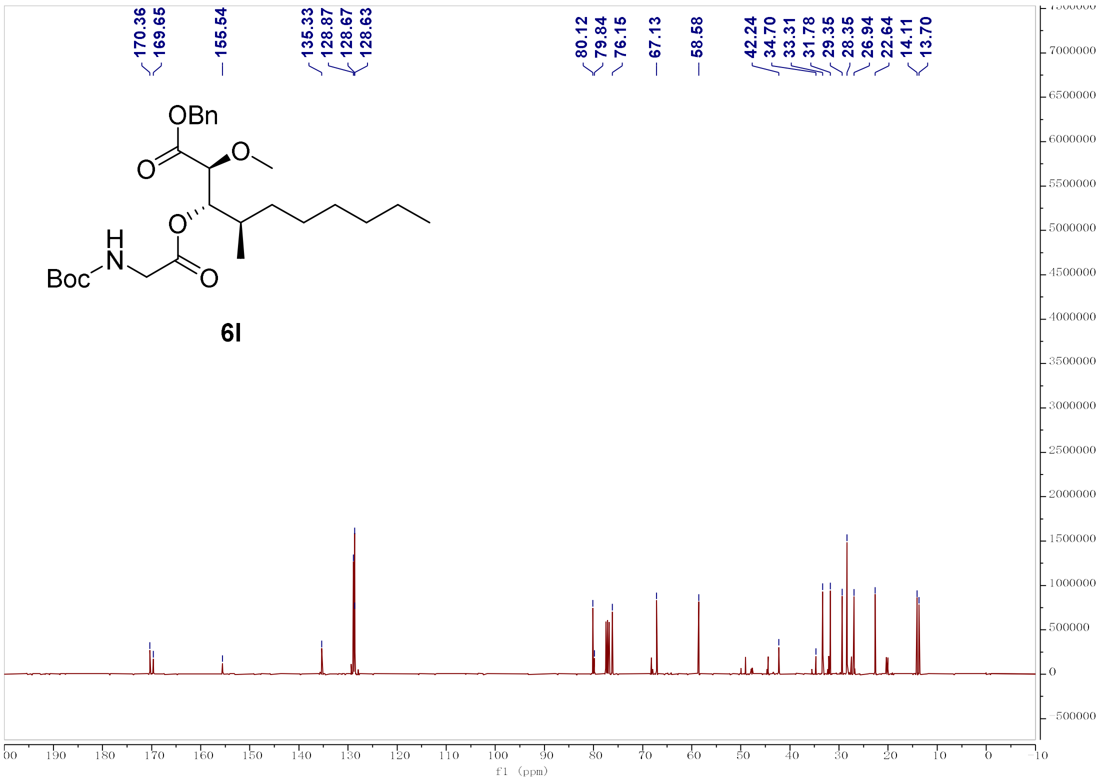

$^{1}$H NMR spectrum of **4h**

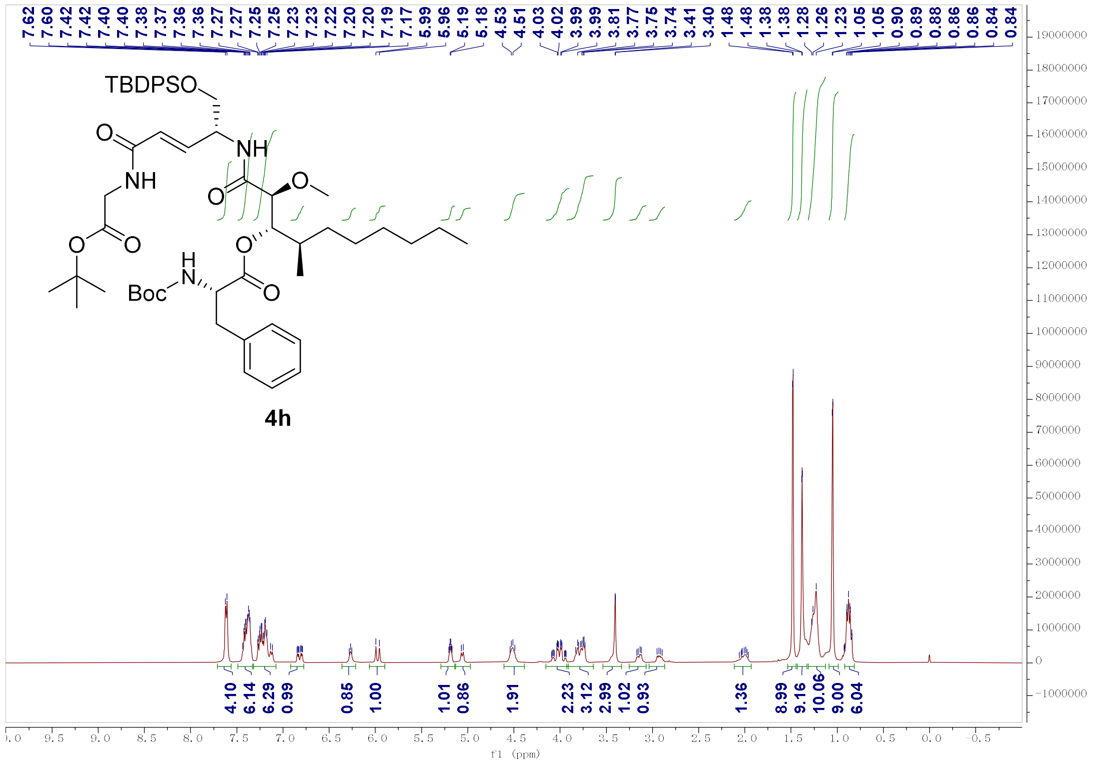

$^{13}$C NMR spectrum of **4h**

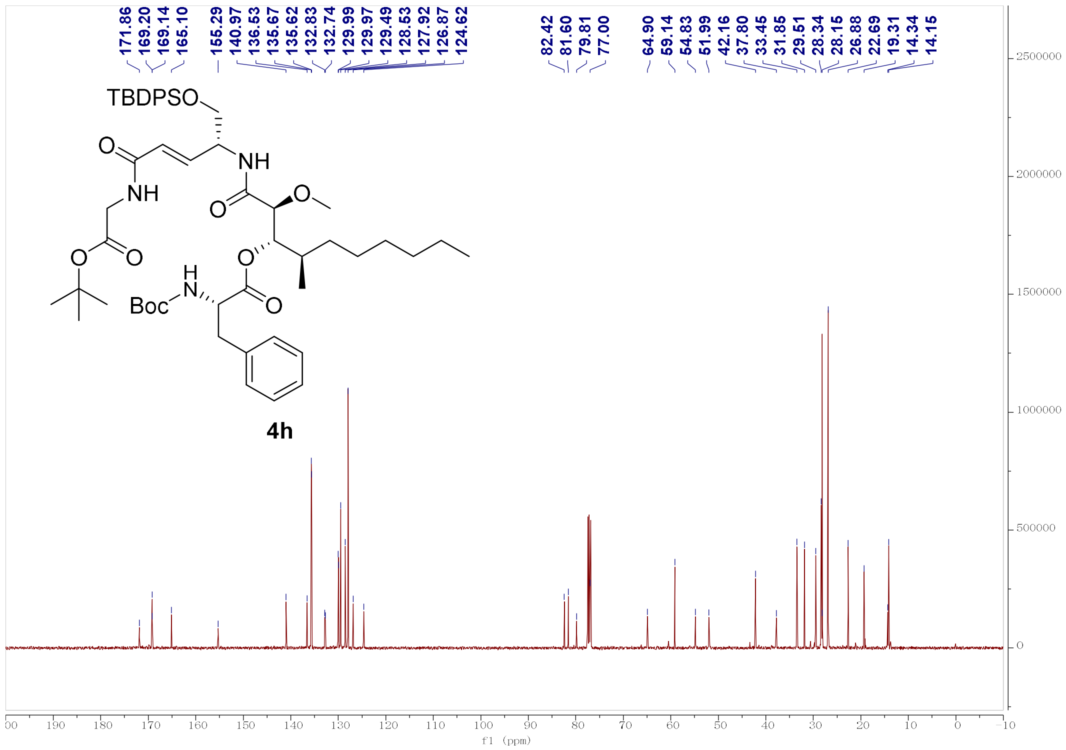

$^{1}$H NMR spectrum of **4i**

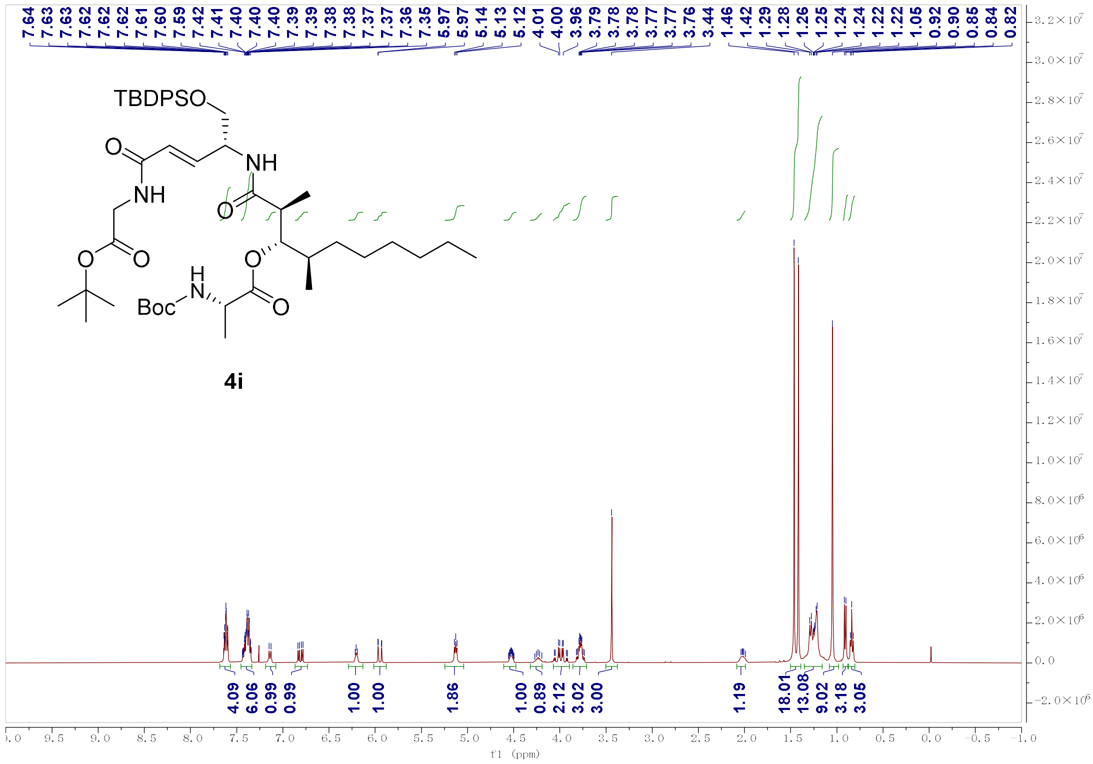

$^{13}$C NMR spectrum of **4i**

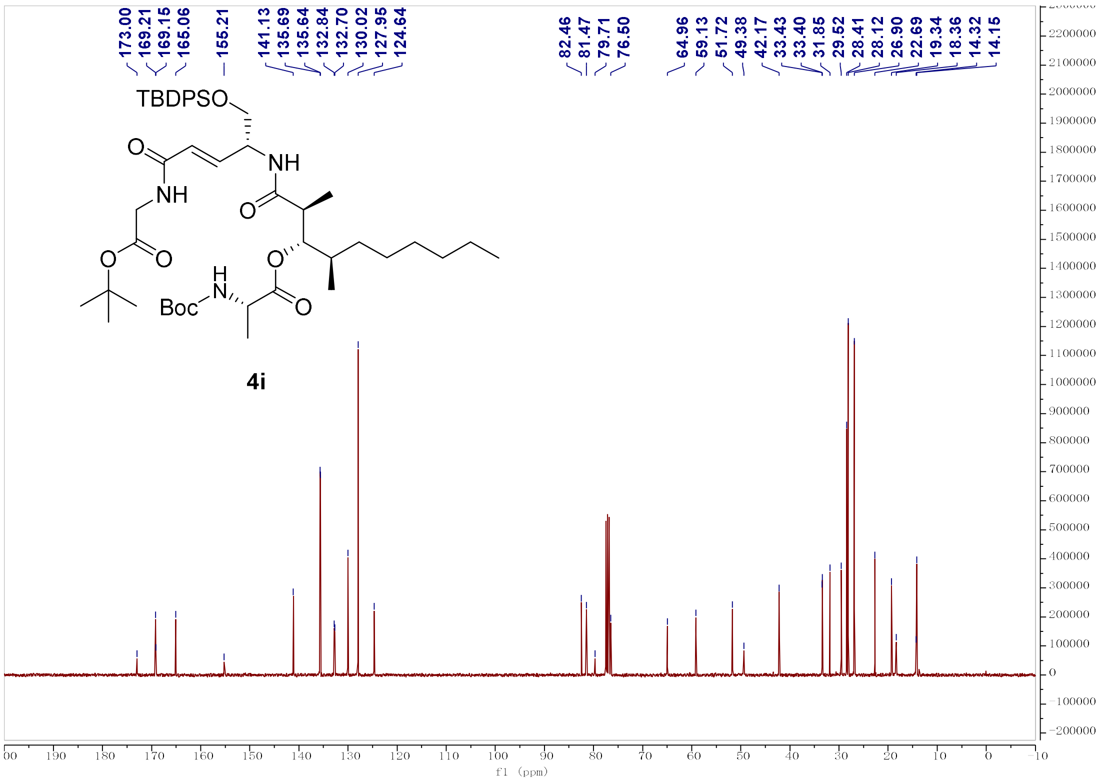

$^{1}$H NMR spectrum of **4j**

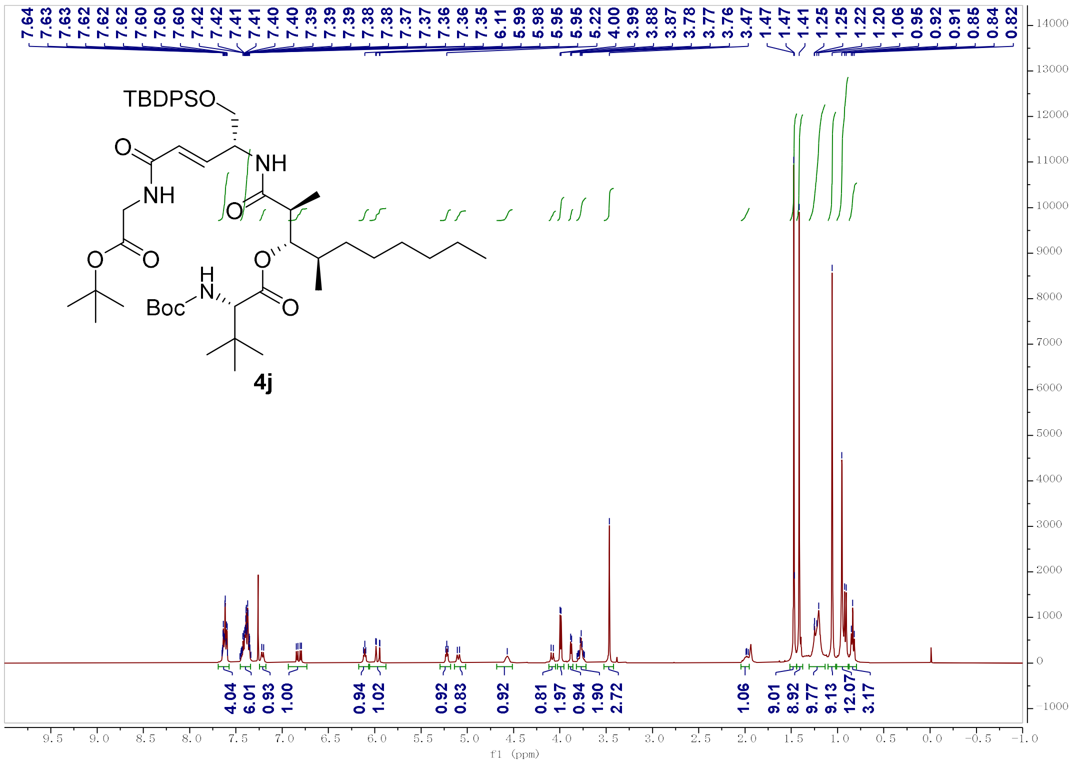

$^{13}$C NMR spectrum of **4j**

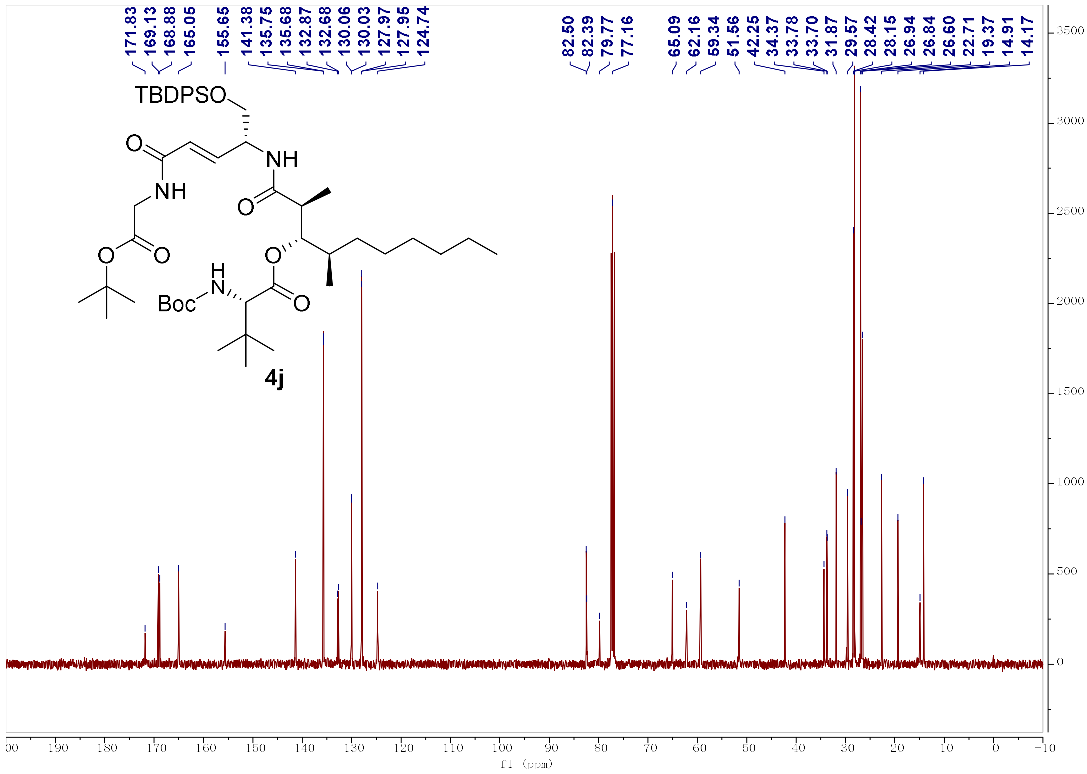

$^{1}$H NMR spectrum of **4k**

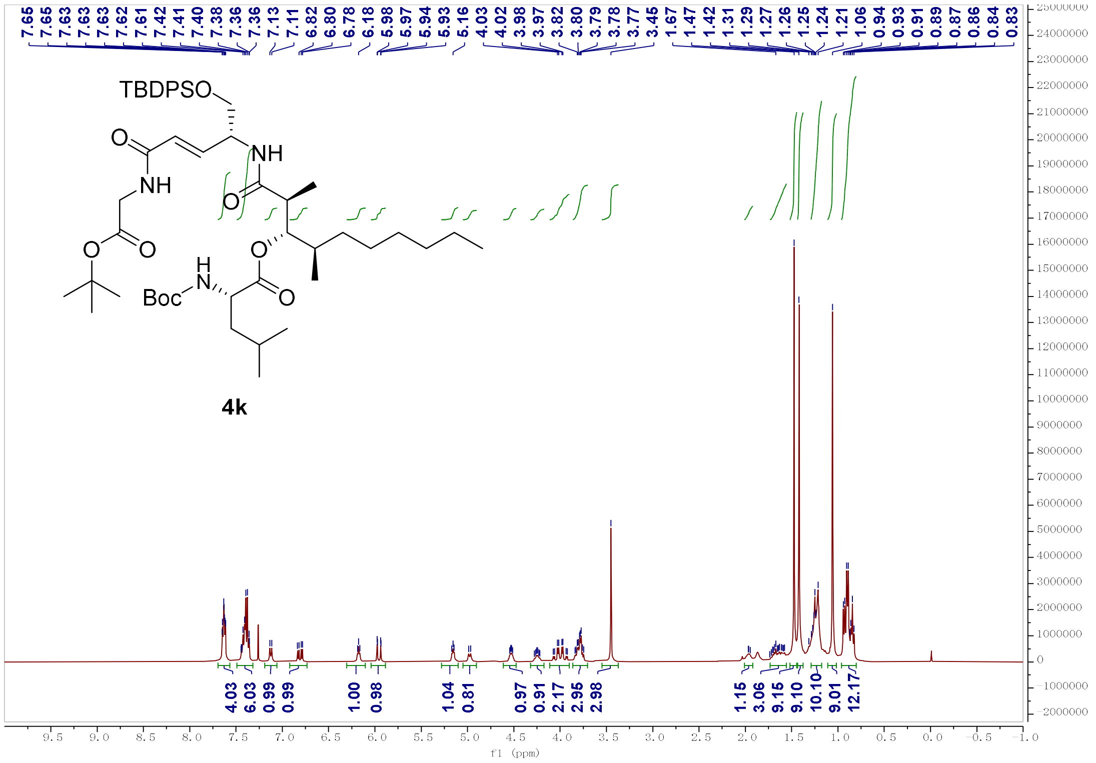

$^{13}$C NMR spectrum of **4k**

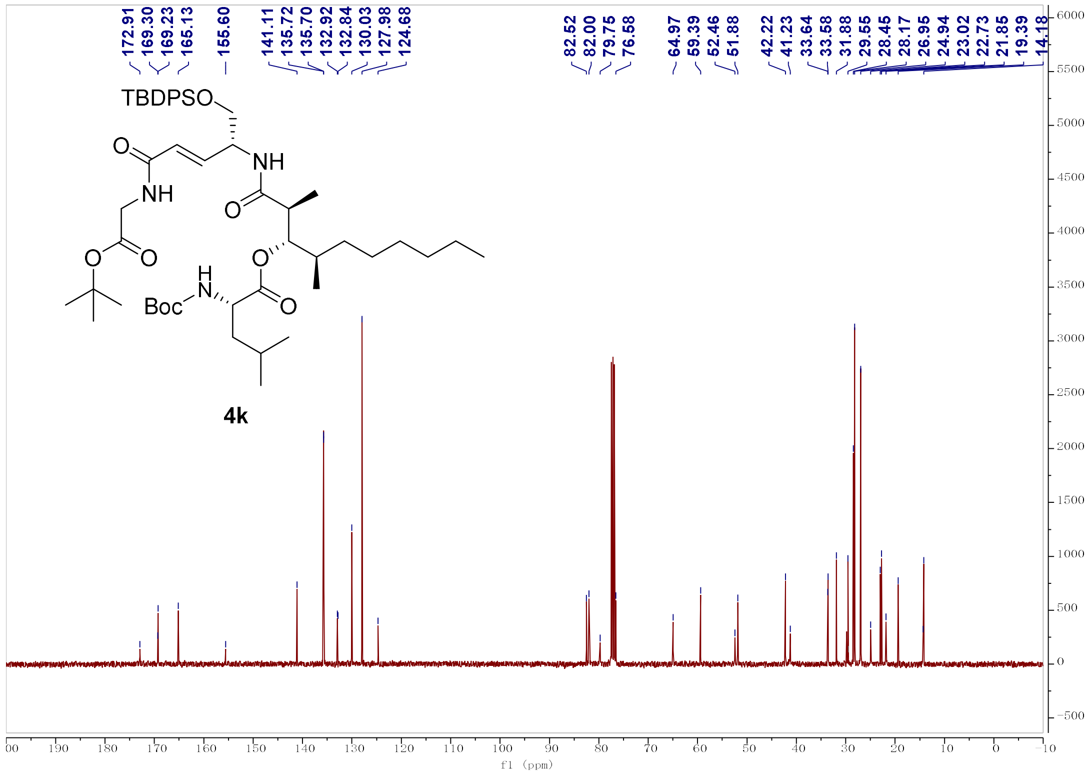

$^{1}$H NMR spectrum of **4l**

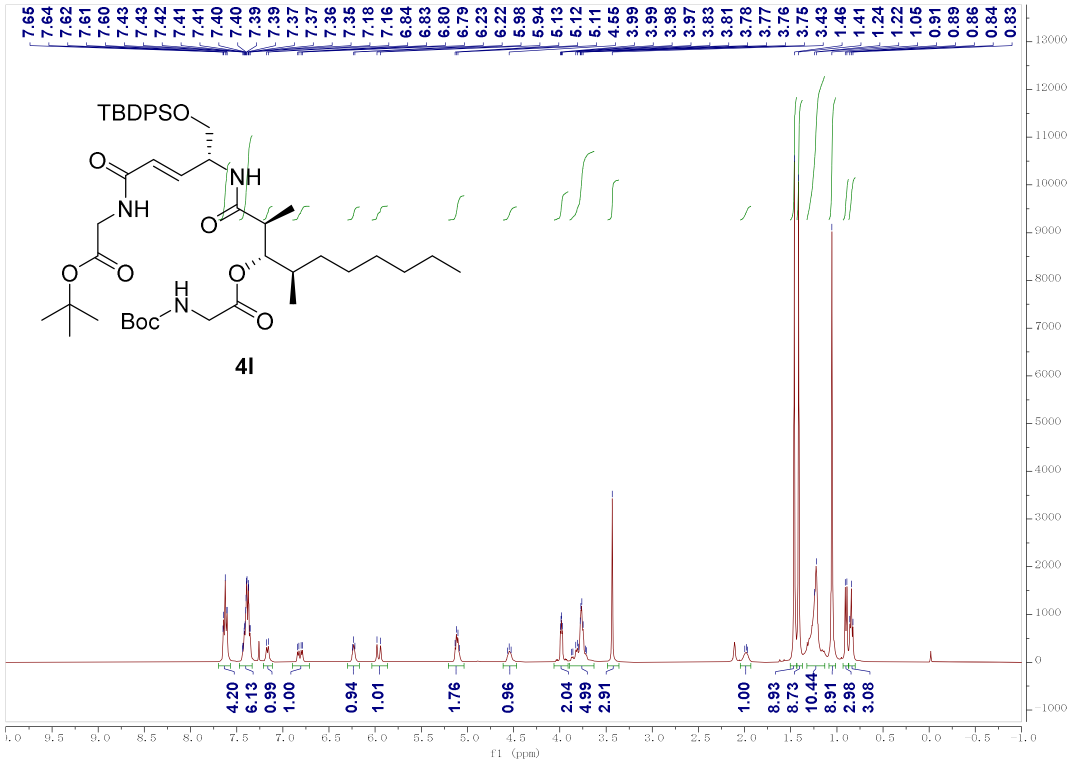

$^{13}$C NMR spectrum of **4l**

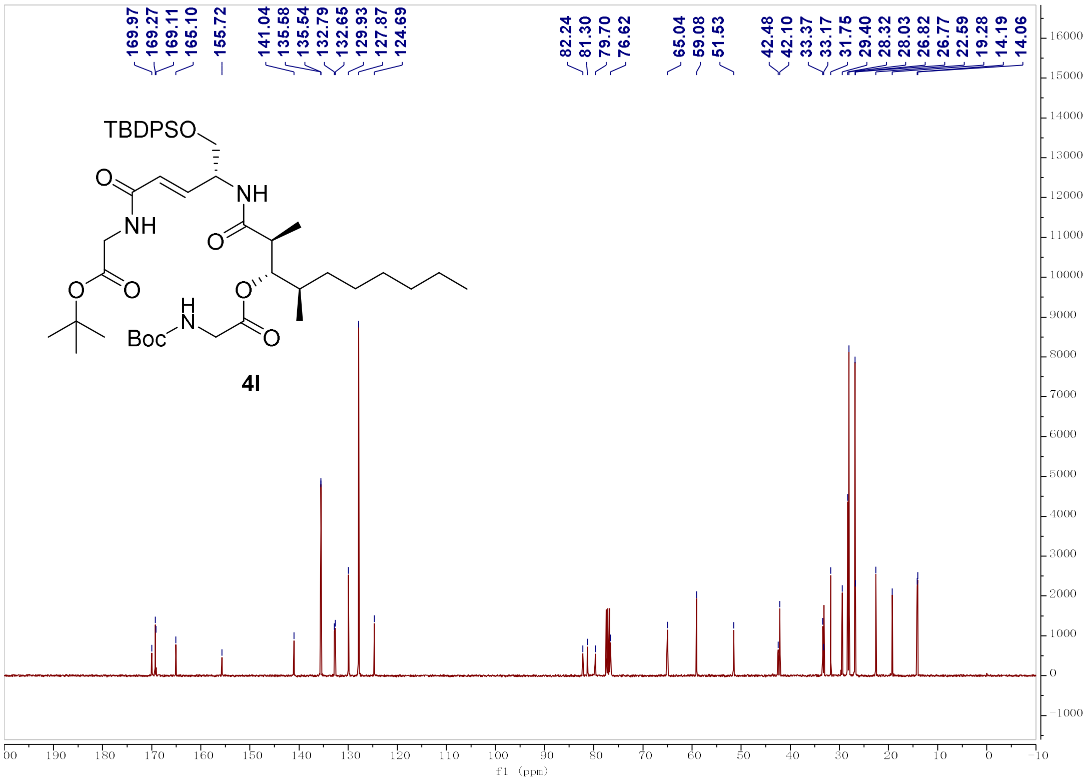

$^{1}$H NMR spectrum of **3h**

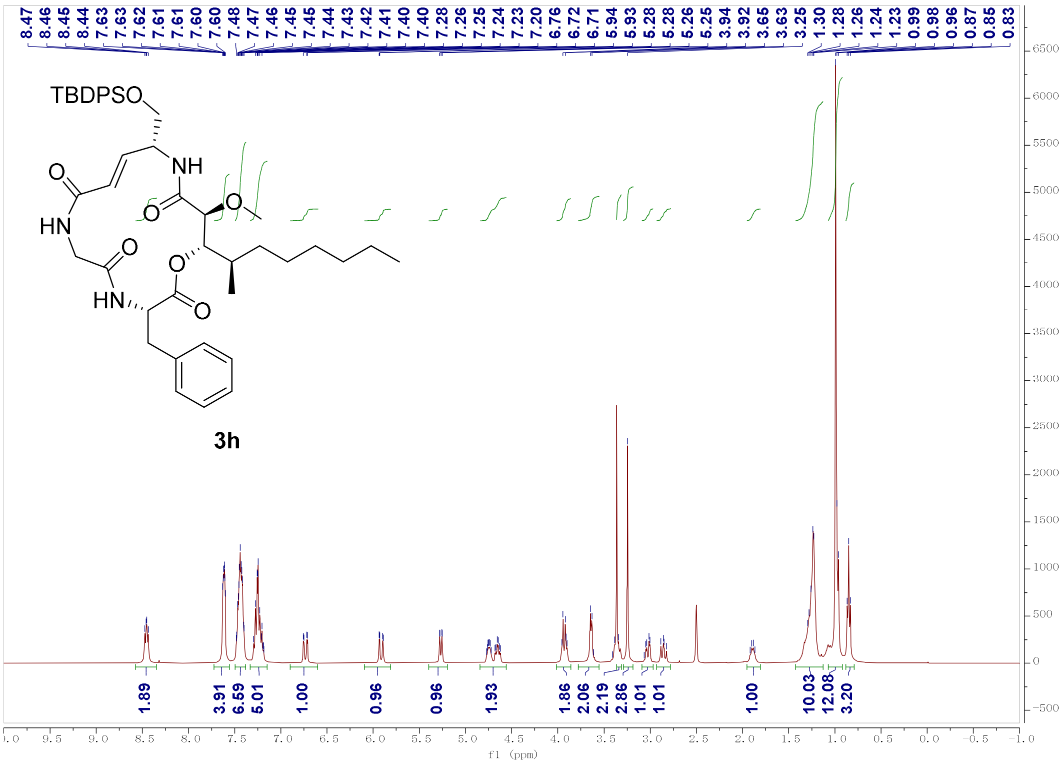


$^{13}$C NMR spectrum of **3h**

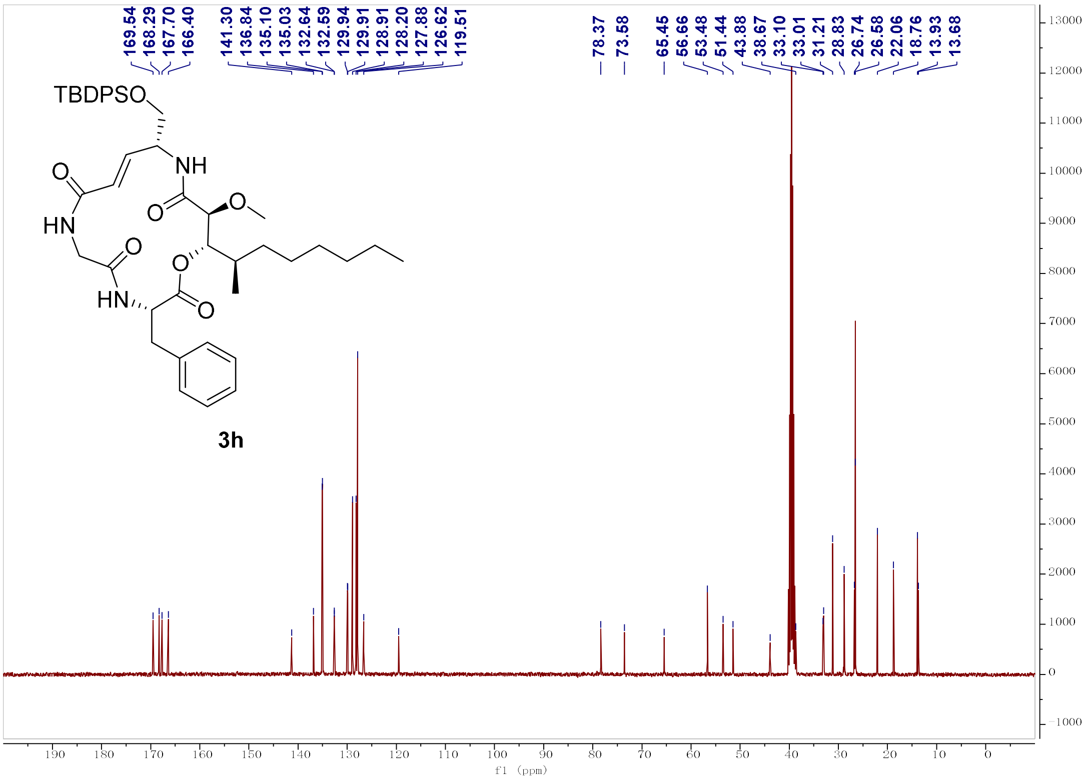

$^{1}$H NMR spectrum of **3i**

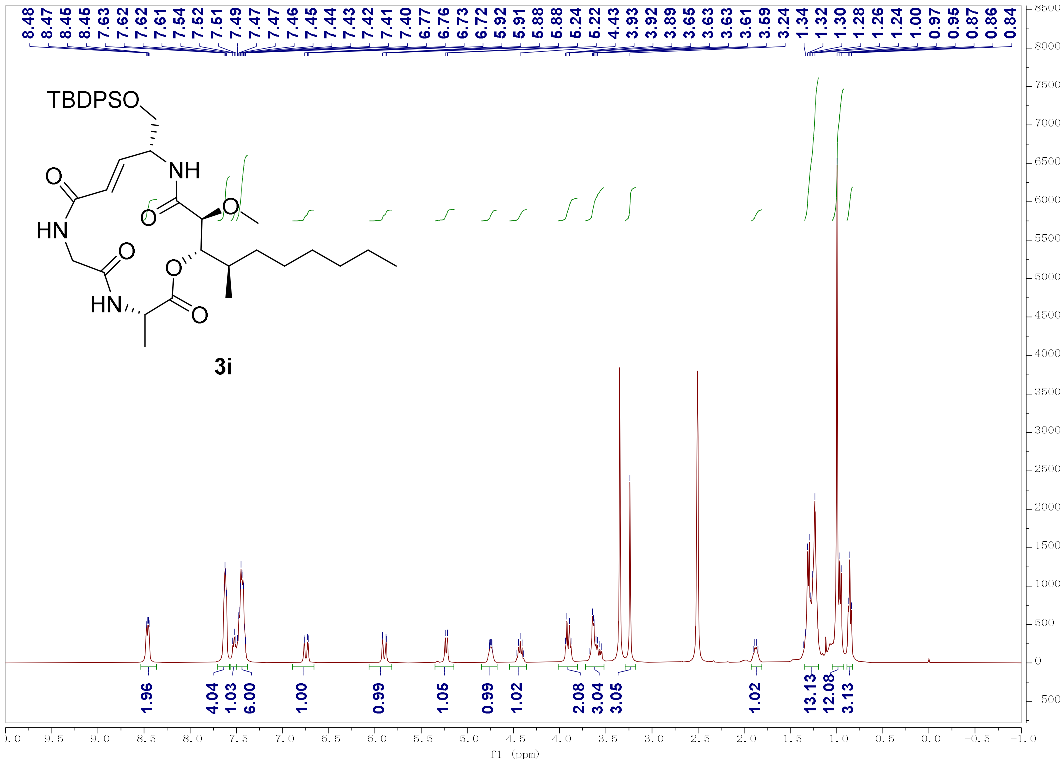


$^{13}$C NMR spectrum of **3i**

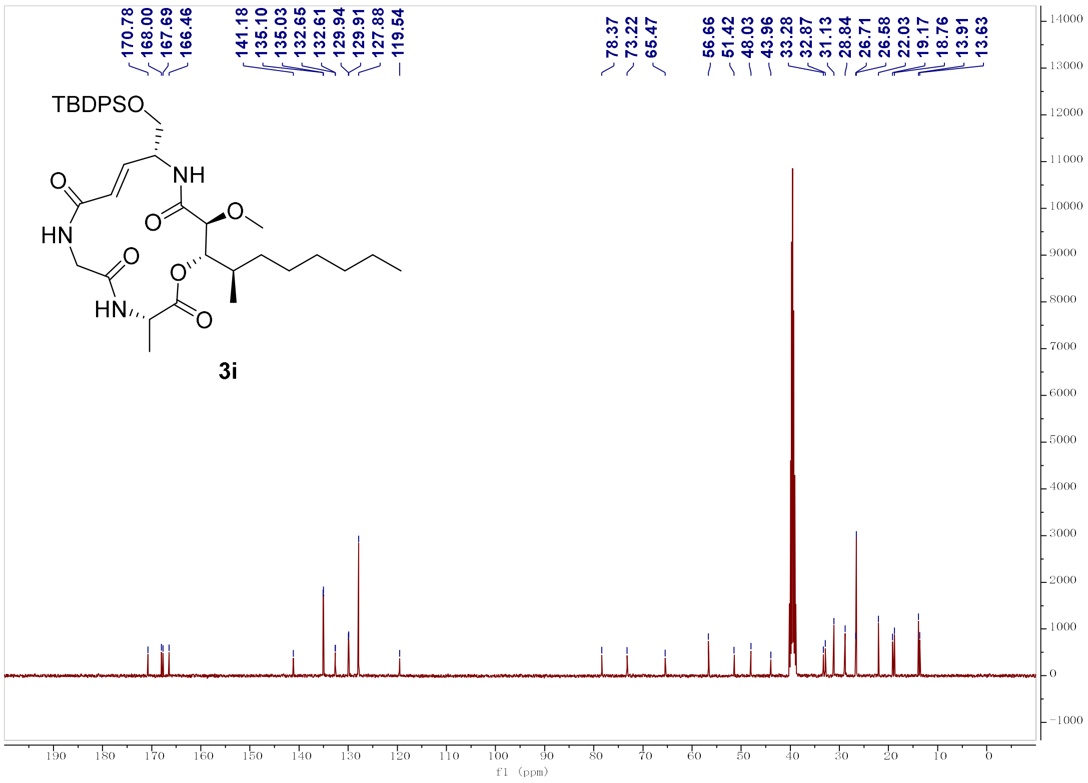

$^{1}H$ NMR spectrum of **3j**

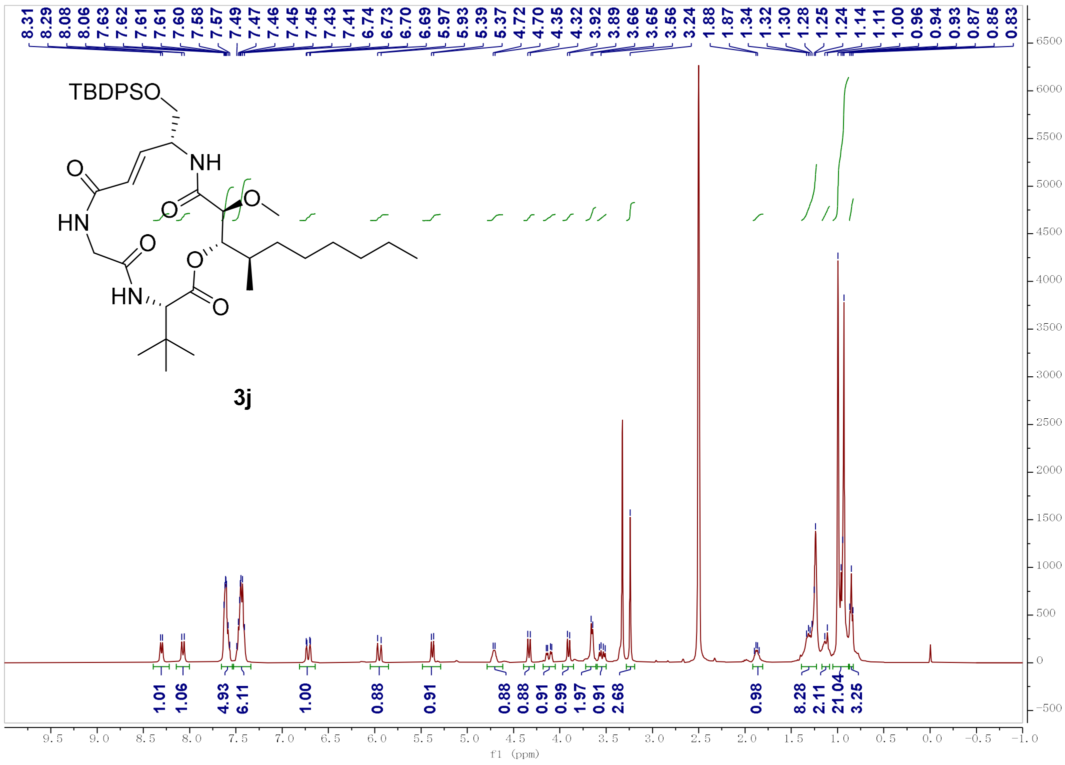

$^{13}C$ NMR spectrum of **3j**

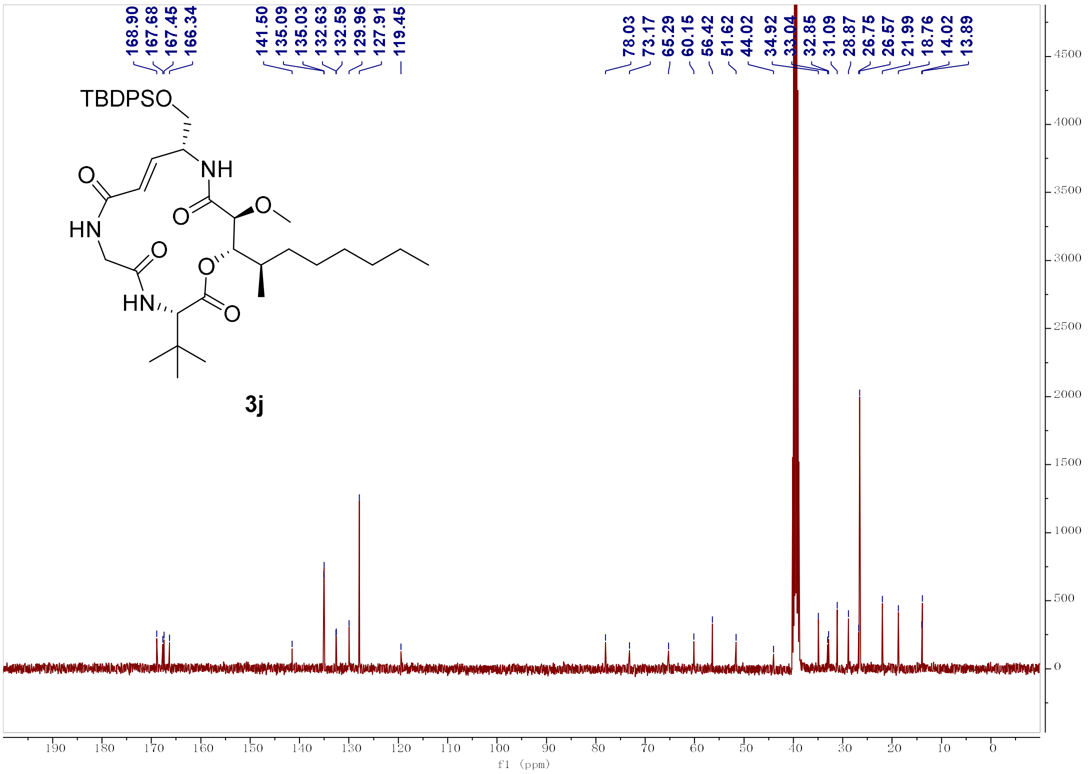

$^{1}H$ NMR spectrum of **3k**

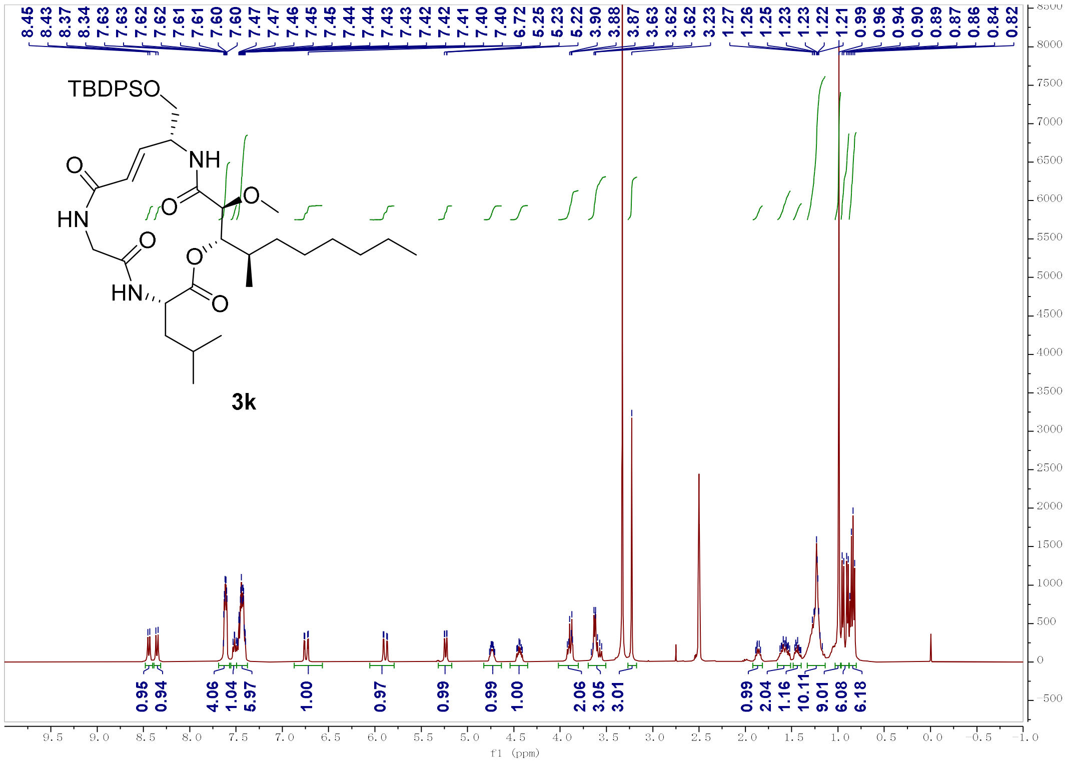

$^{13}C$ NMR spectrum of **3k**

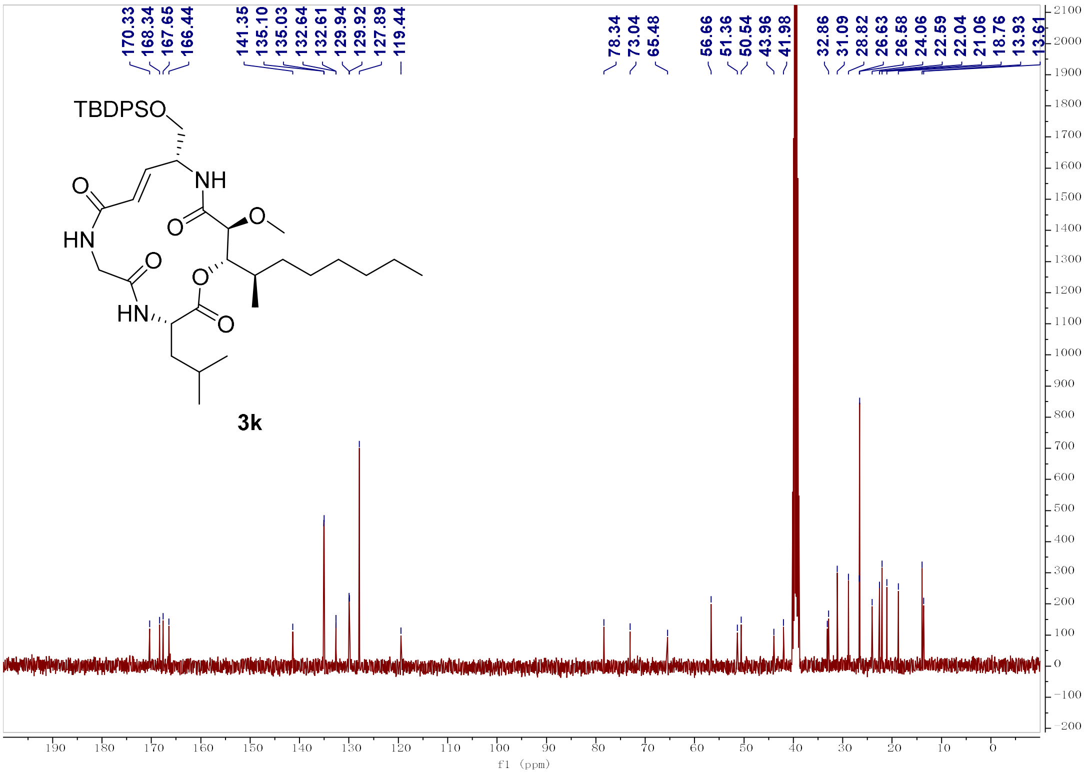

$^{1}$H NMR spectrum of **3l**

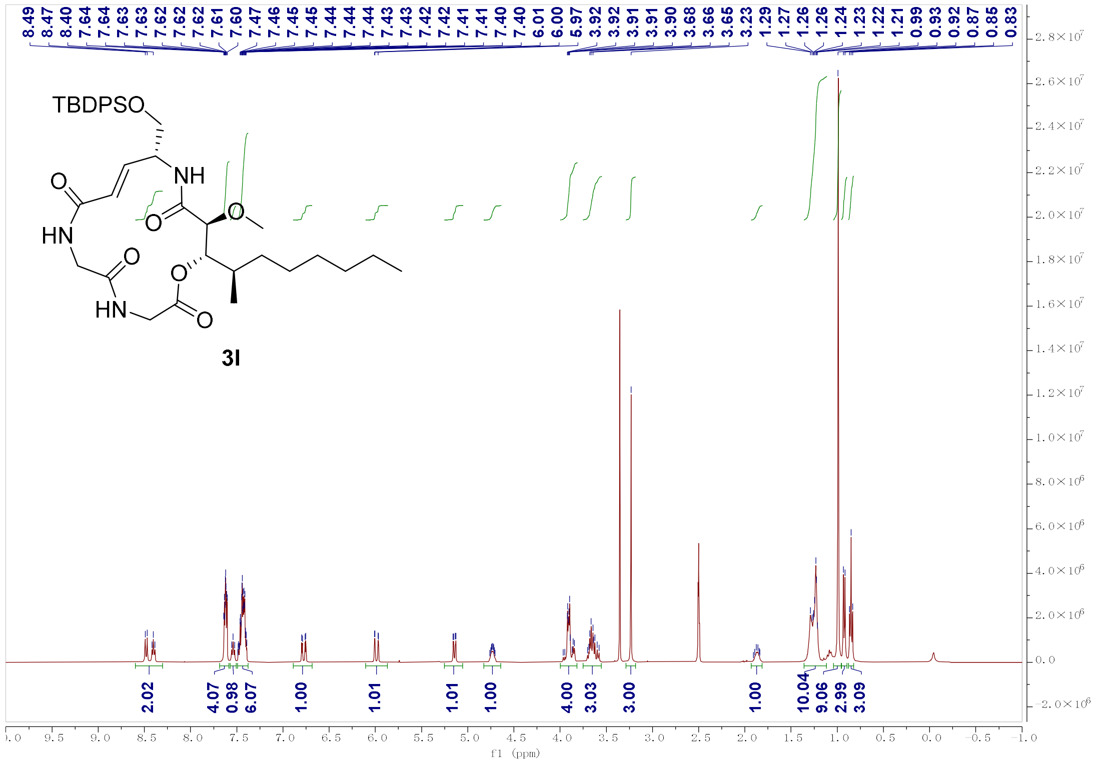

$^{13}$C NMR spectrum of **3l**

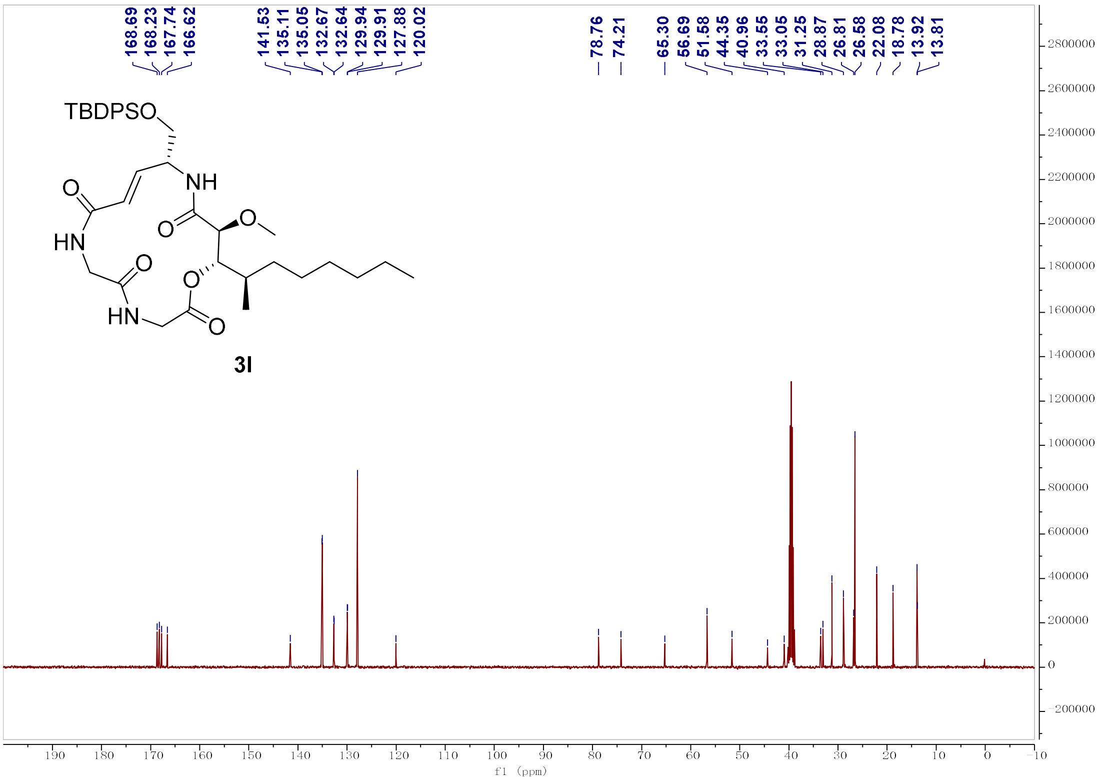

$^{1}H$ NMR spectrum of **1h**

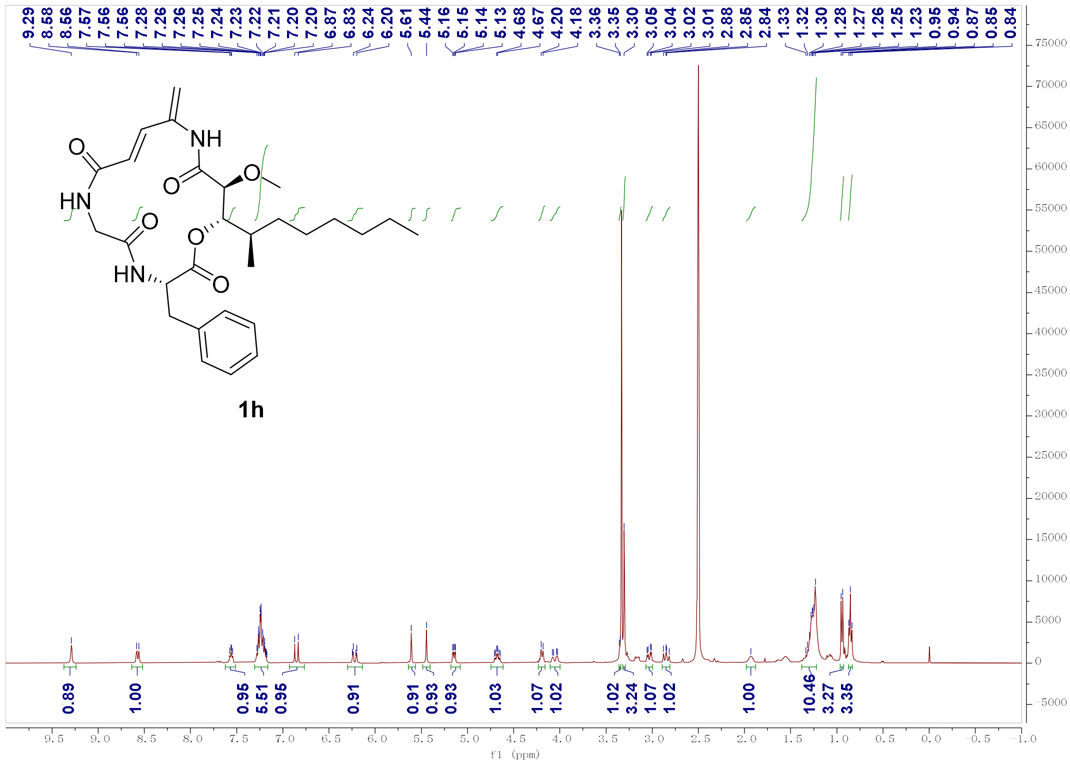

$^{13}C$ NMR spectrum of **1h**

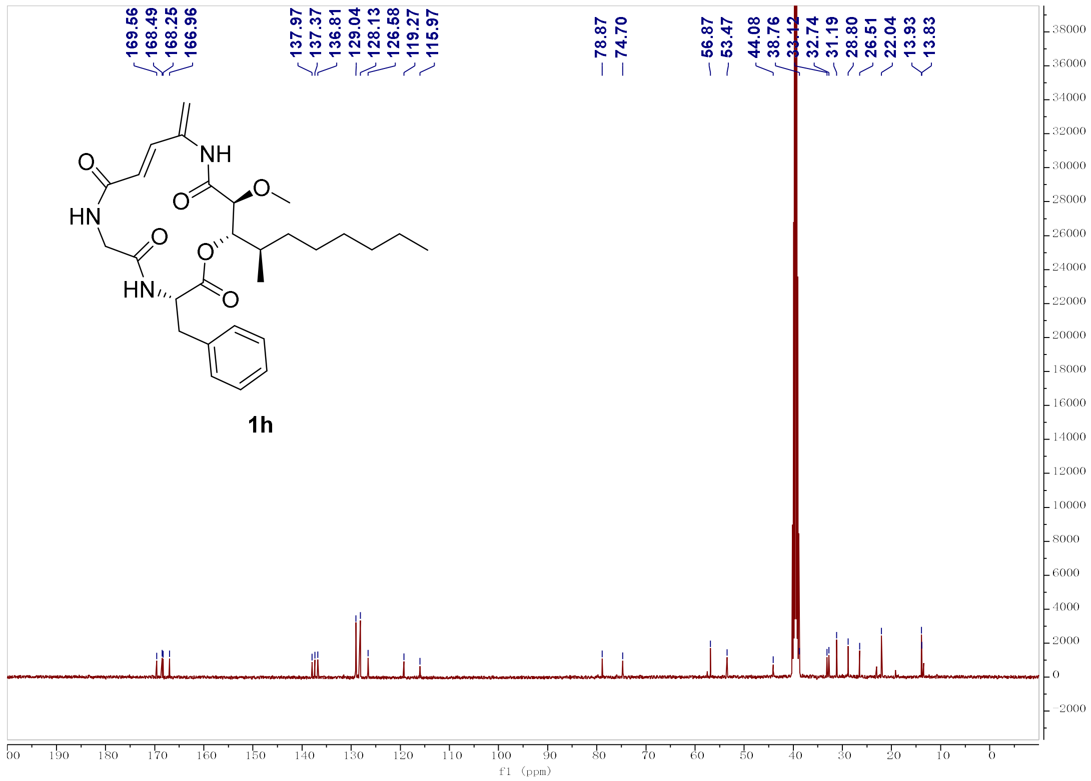

$^{1}$H NMR spectrum of **1i**

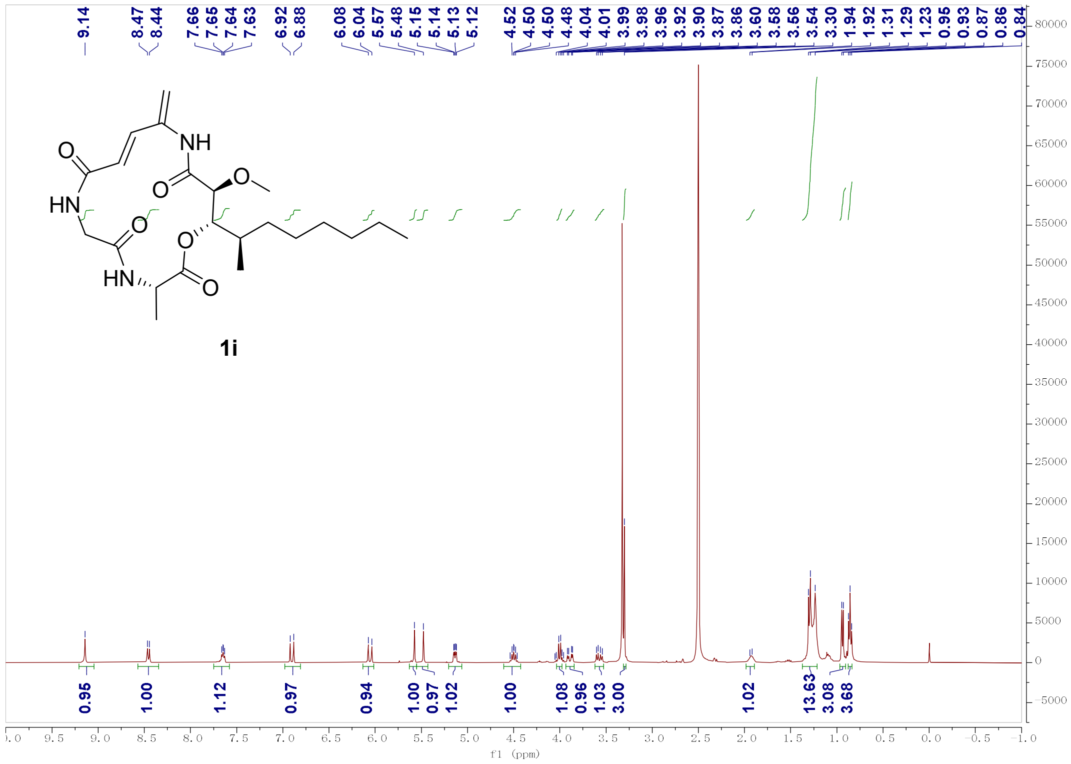


$^{13}$C NMR spectrum of **1i**

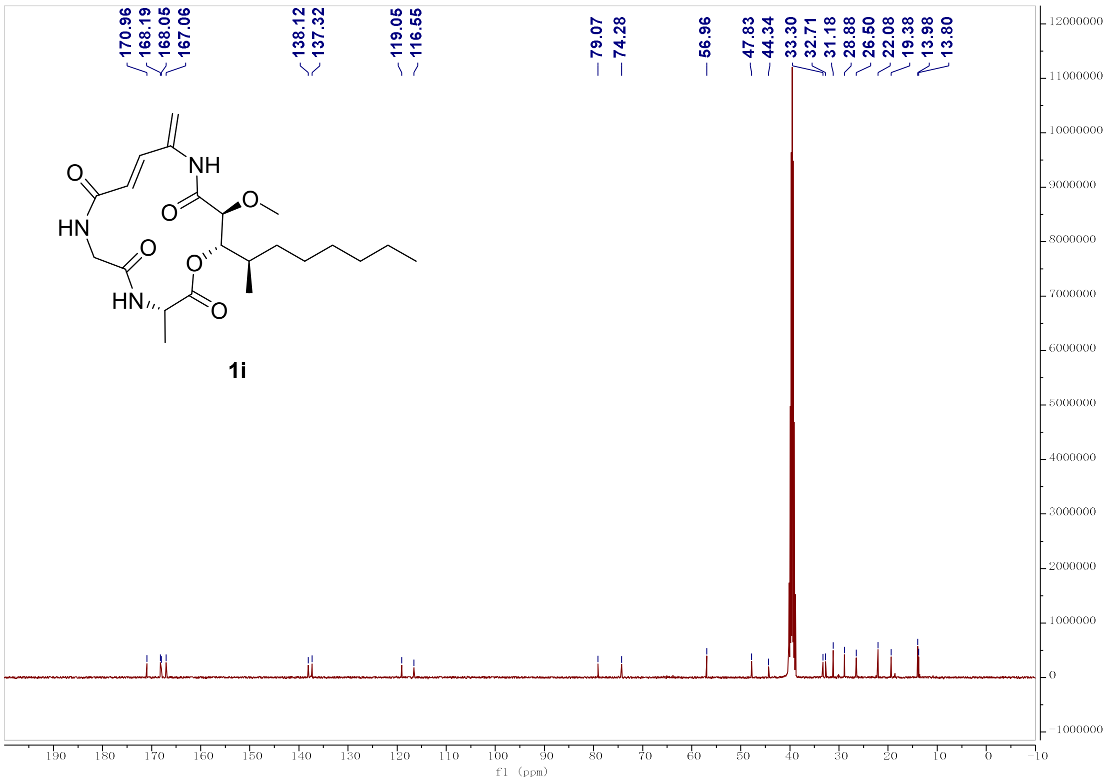

# $^{1}$H NMR spectrum of **1j**

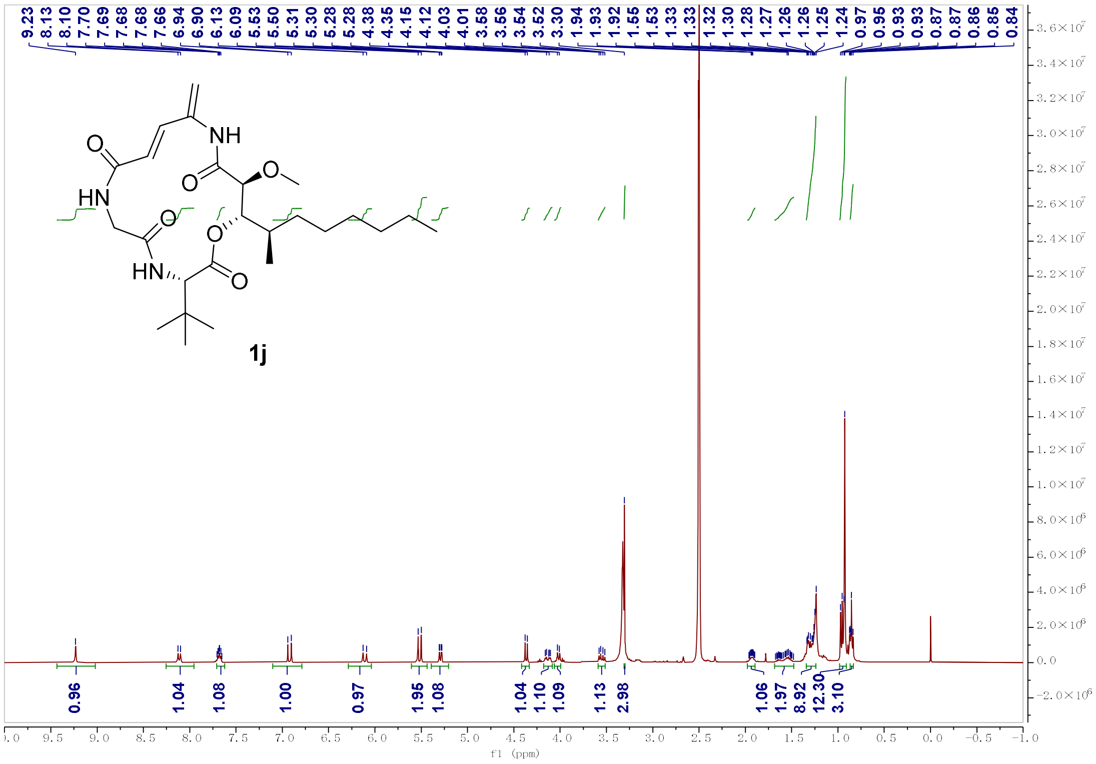


# $^{13}$C NMR spectrum of **1j**

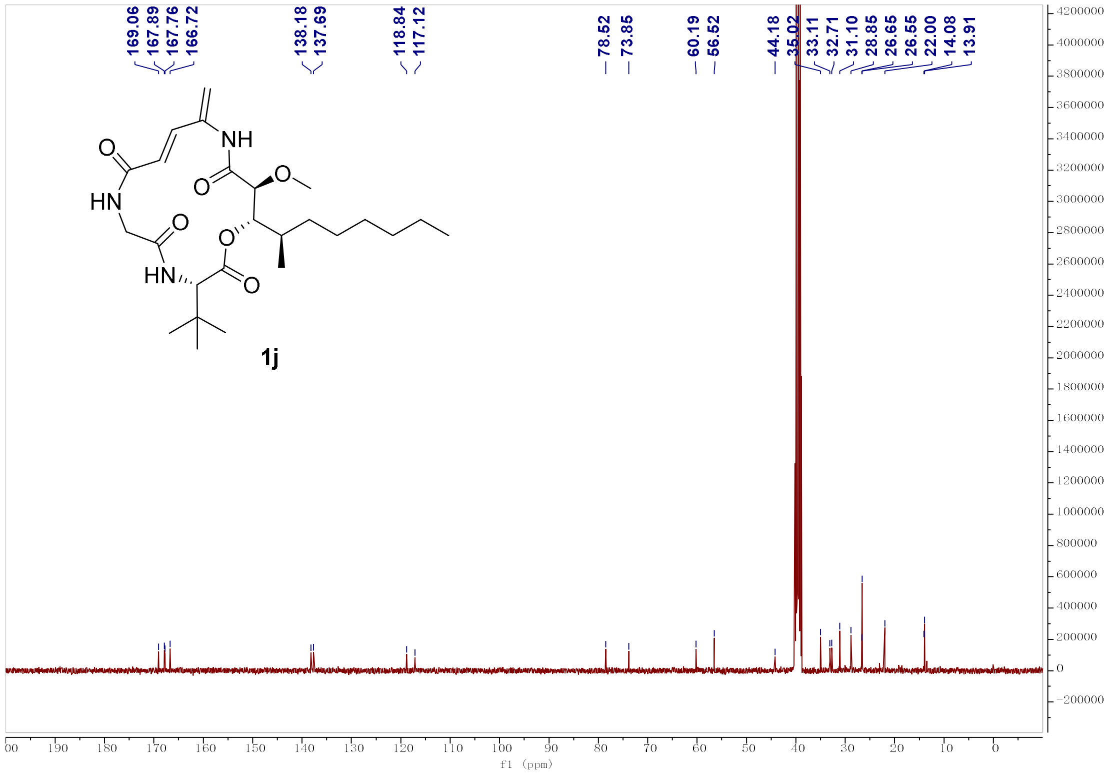

$^{1}$H NMR spectrum of **1k**

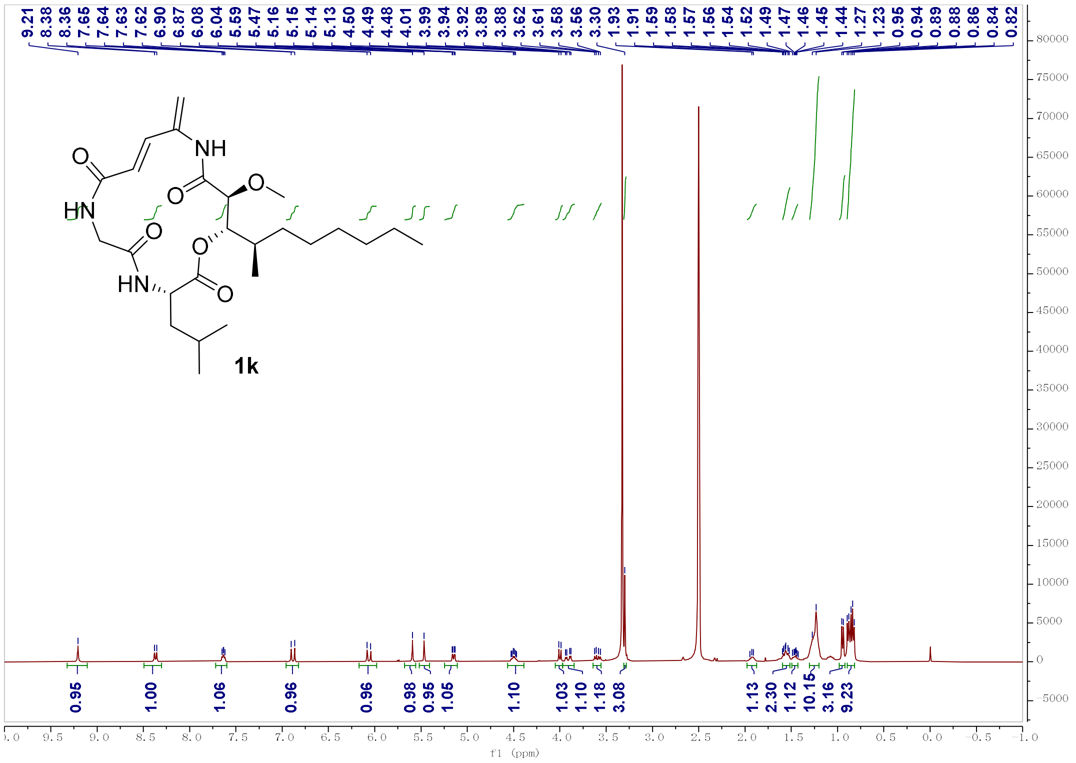

$^{13}$C NMR spectrum of **1k**

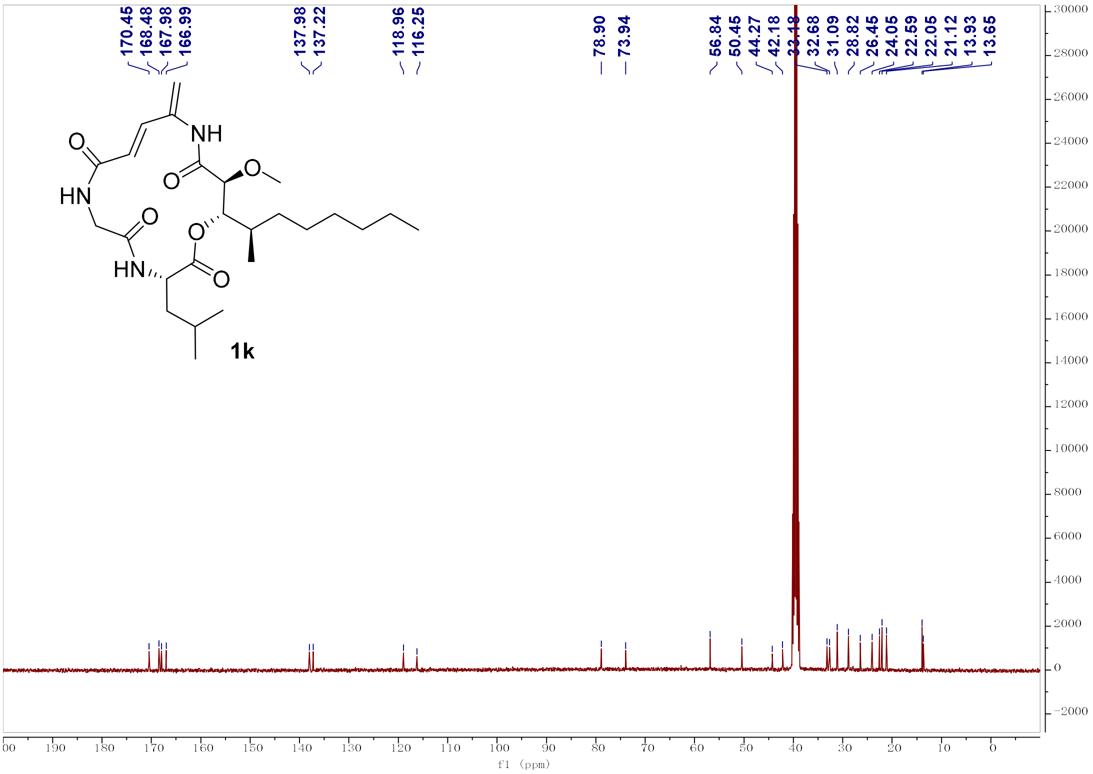

$^{1}$H NMR spectrum of **1l**

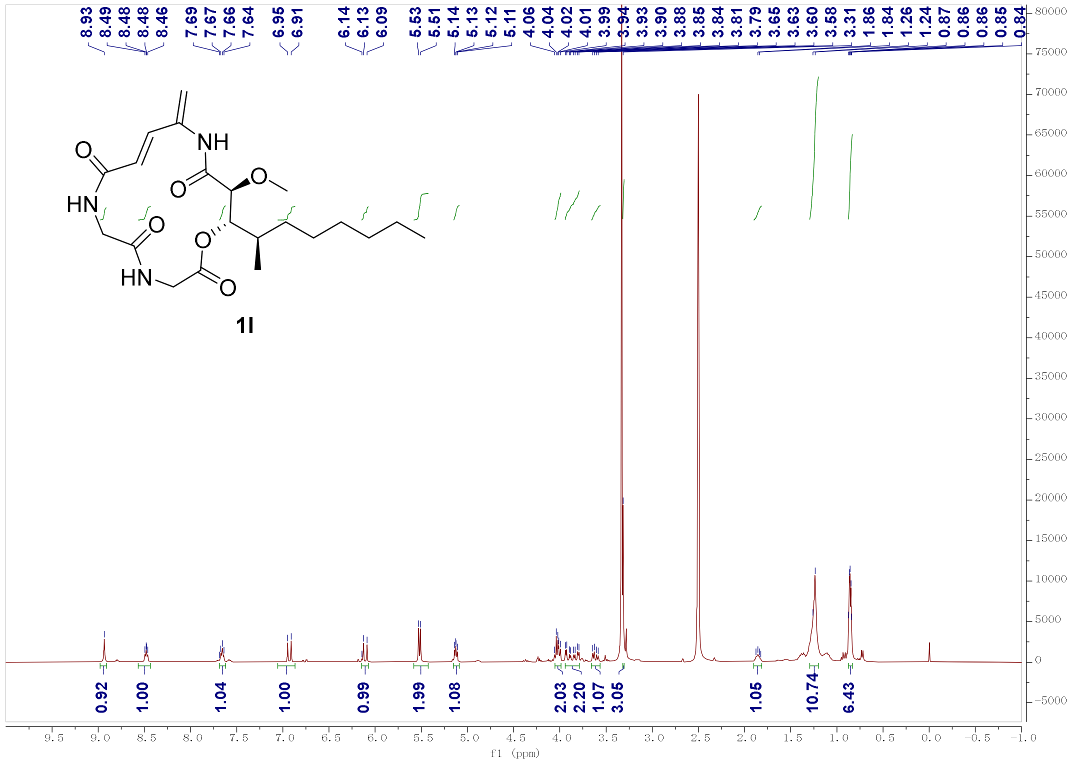

$^{13}$C NMR spectrum of **1l**

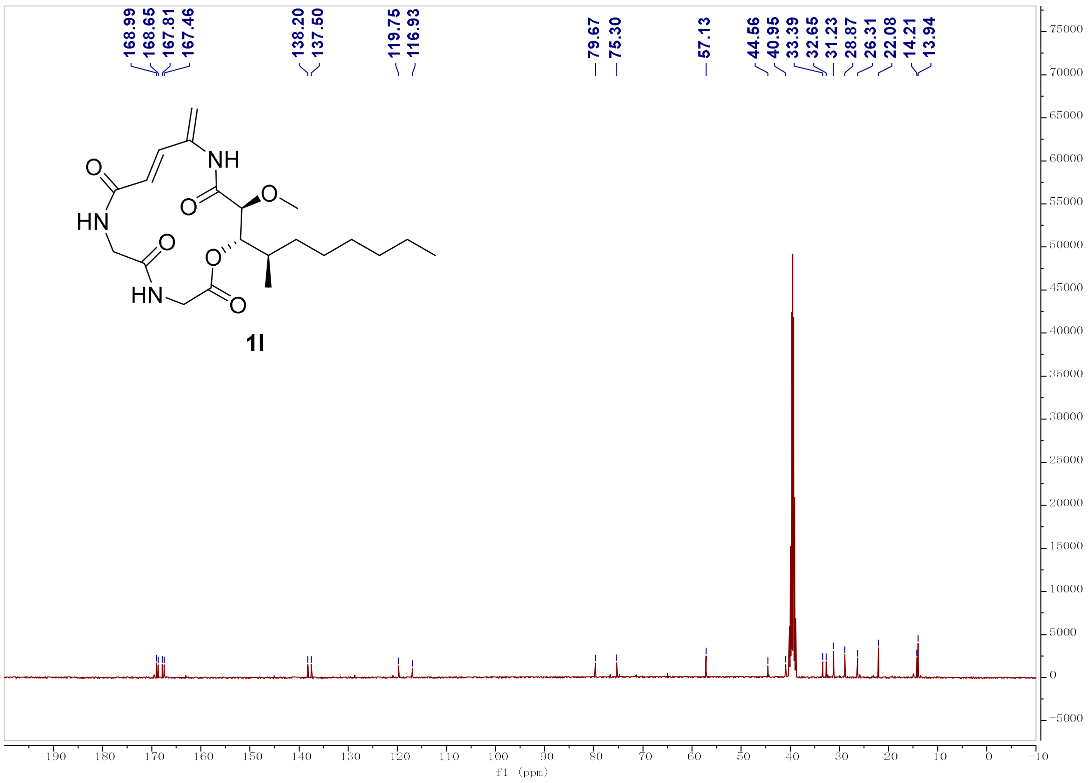

## $^{1}$H NMR spectrum of **13**

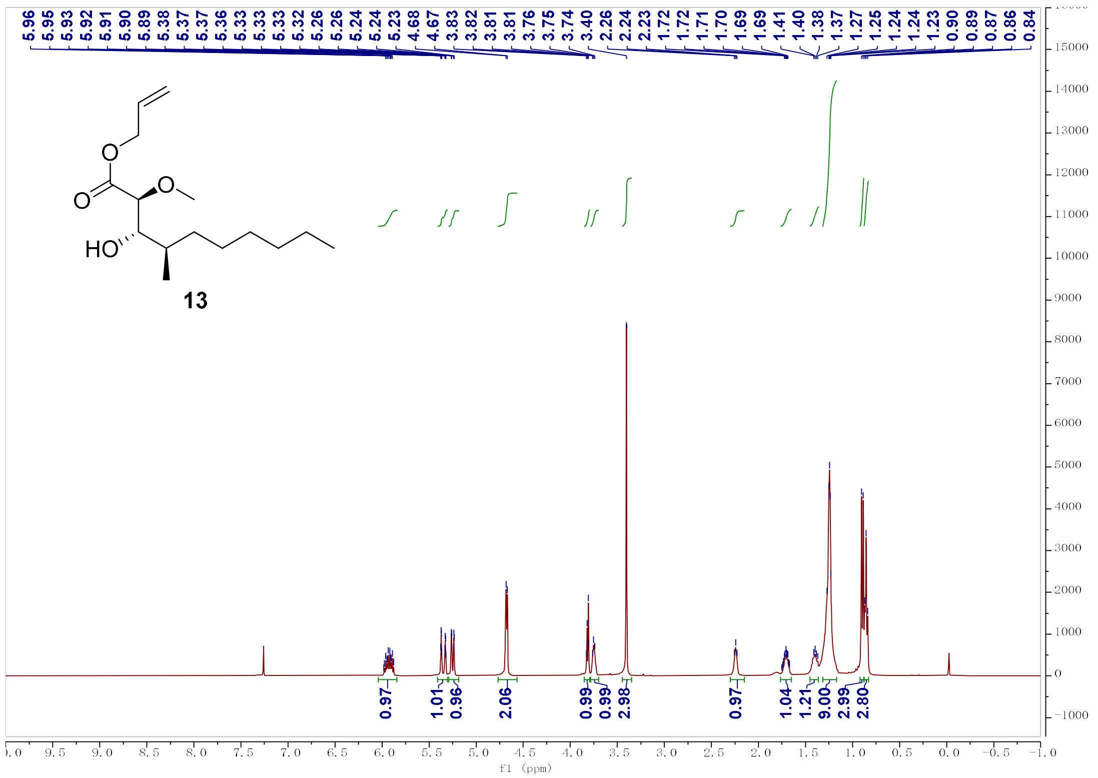


## $^{13}$C NMR spectrum of **13**

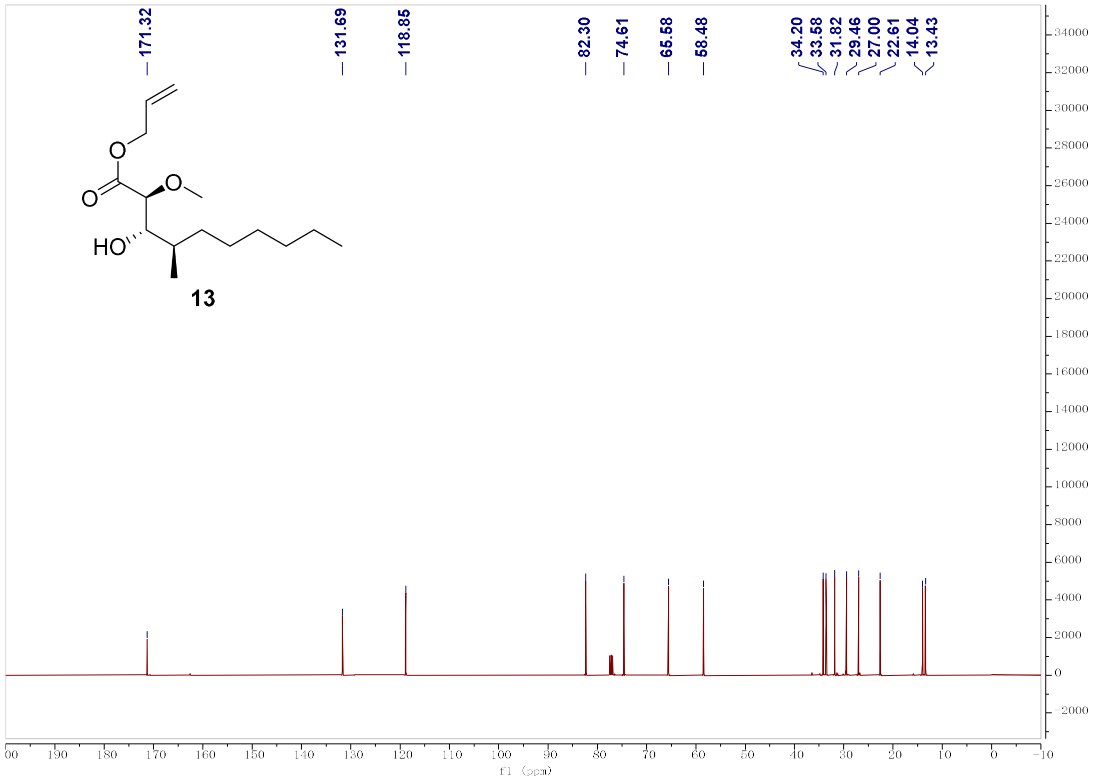

<sup></sup>

$^1$H NMR spectrum of **6m**

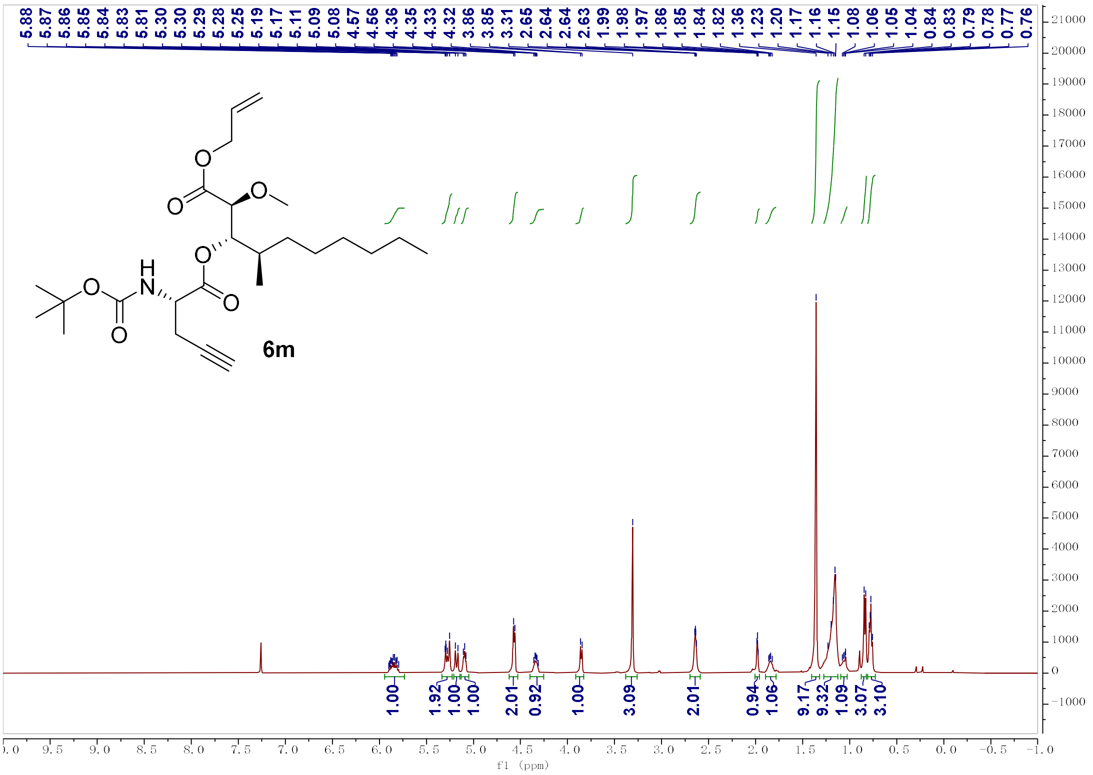


$^{13}$C NMR spectrum of **6m**

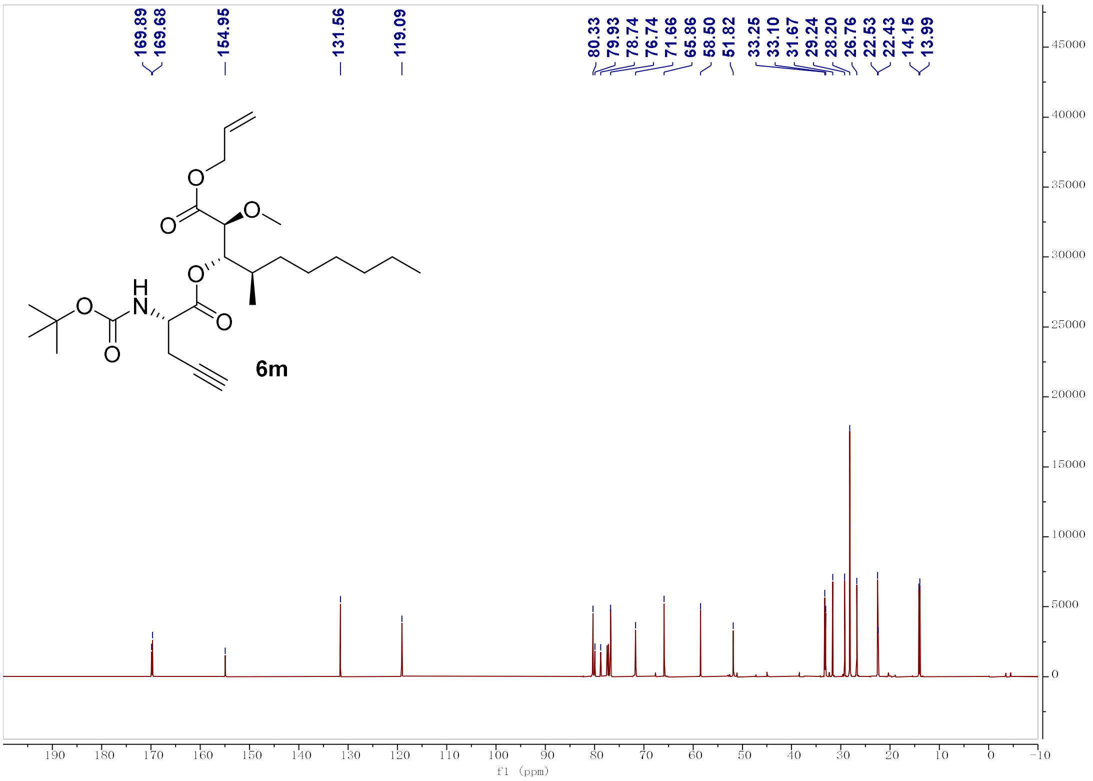

# $^{1}$H NMR spectrum of **4m**

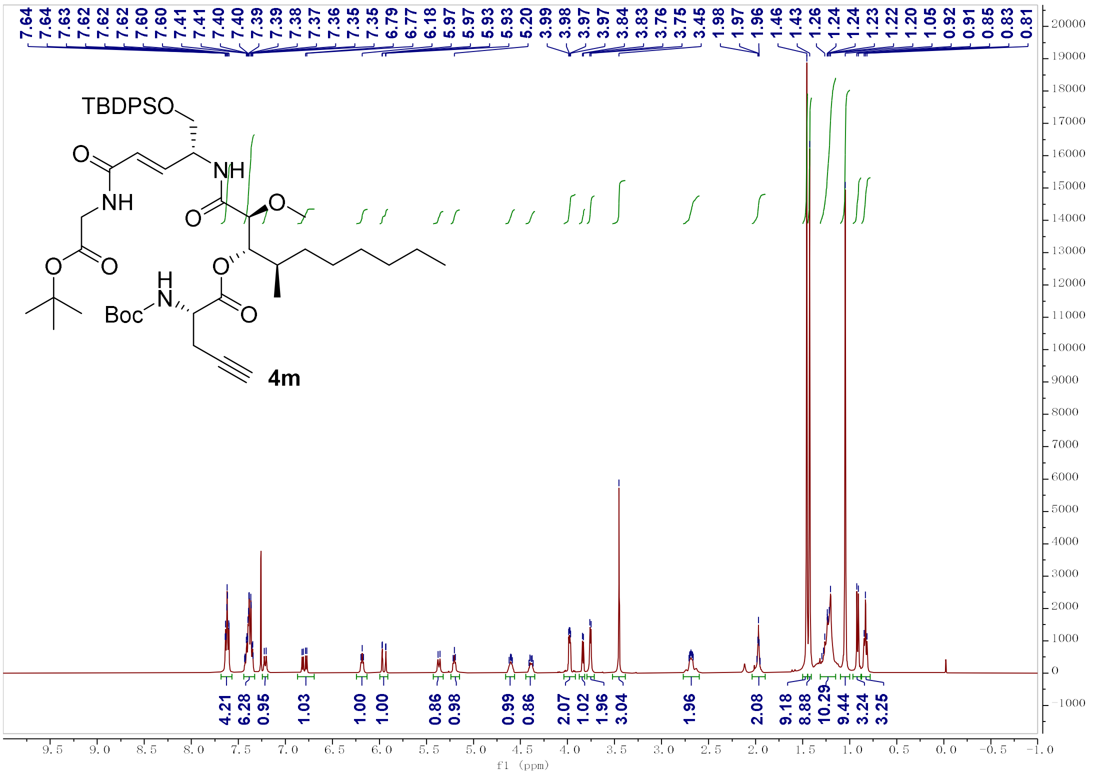


# $^{13}$C NMR spectrum of **4m**

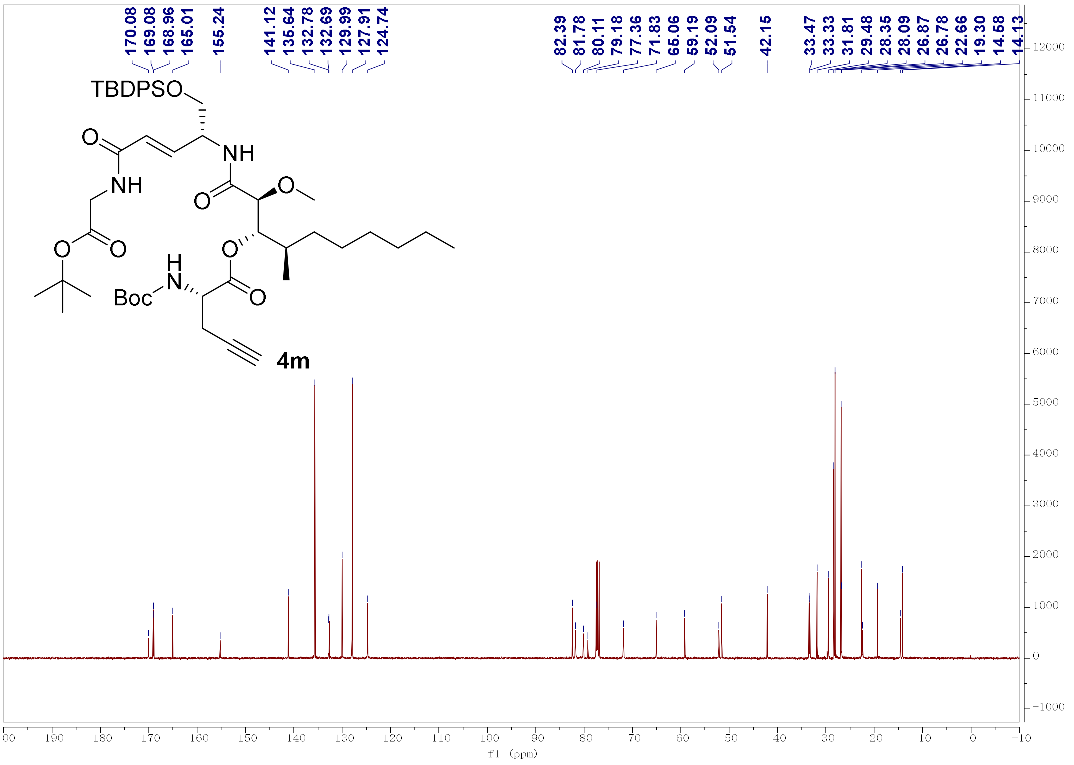

$^{1}$H NMR spectrum of **3m**

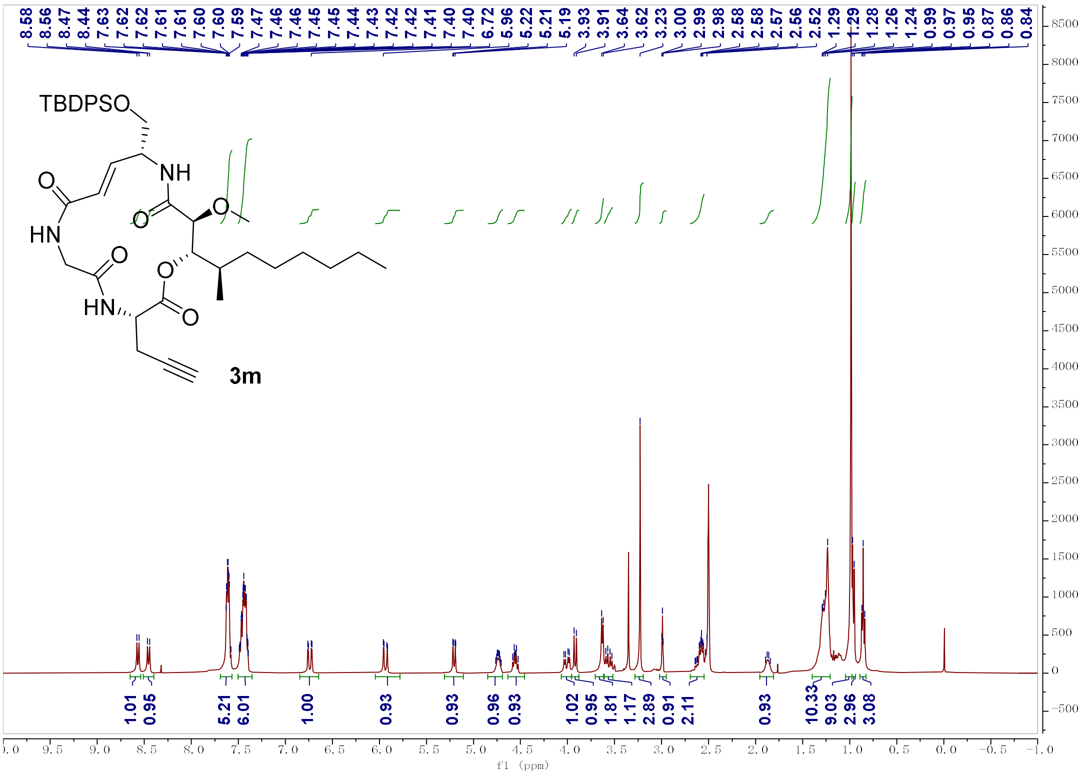

$^{13}$C NMR spectrum of **3m**

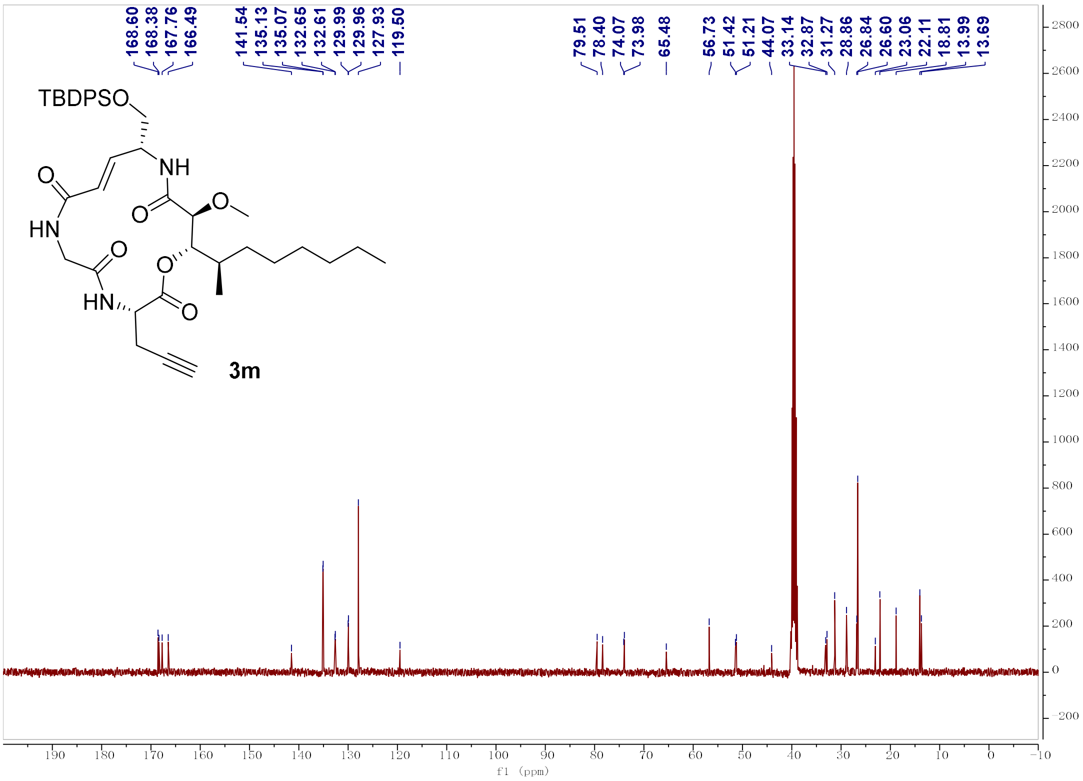

$^{1}$H NMR spectrum of **1m**

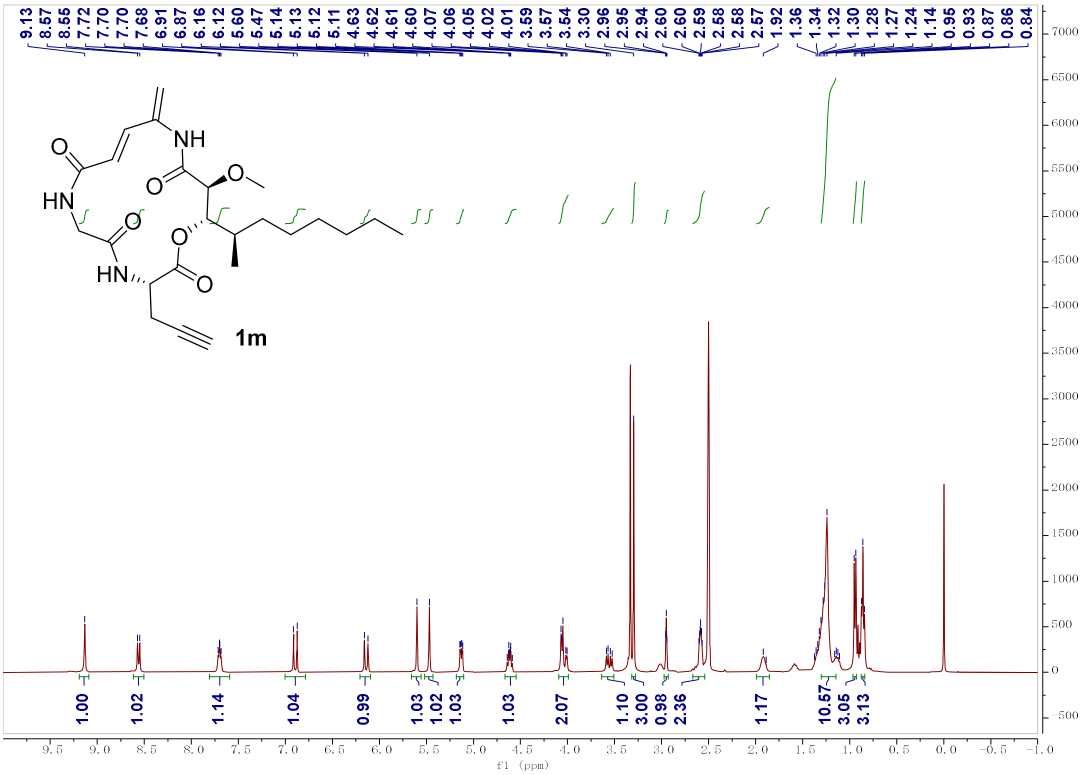

$^{13}$C NMR spectrum of **1m**

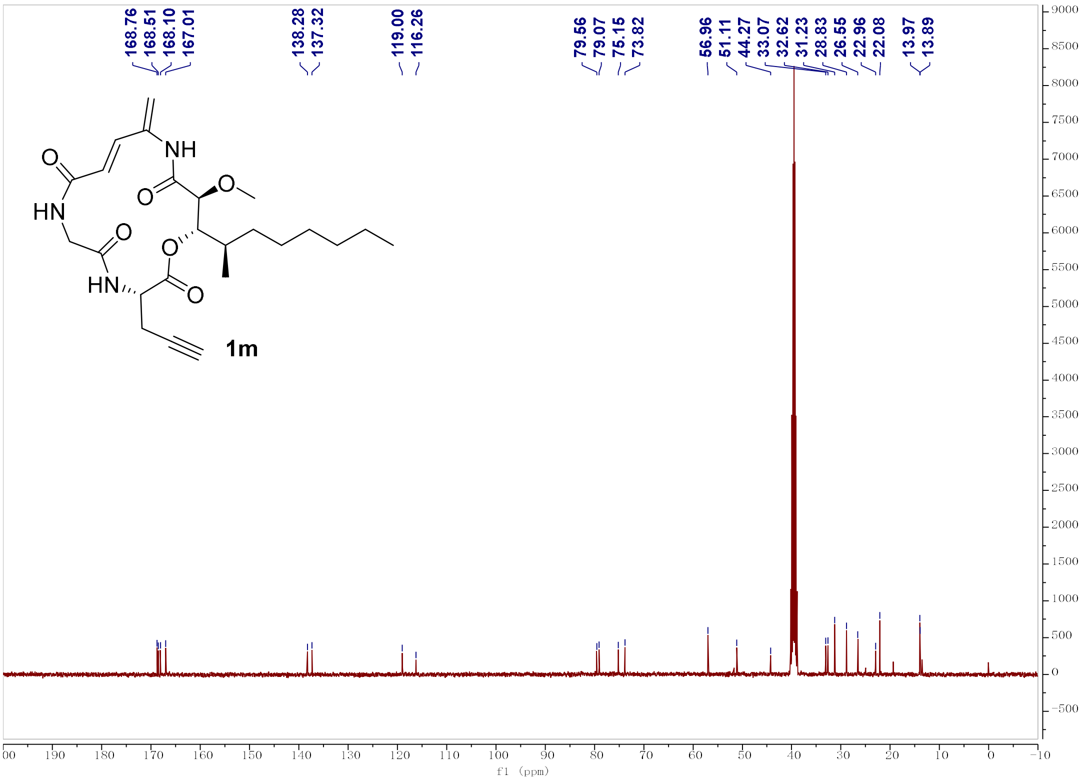

$^{1}$H NMR spectrum of **15**

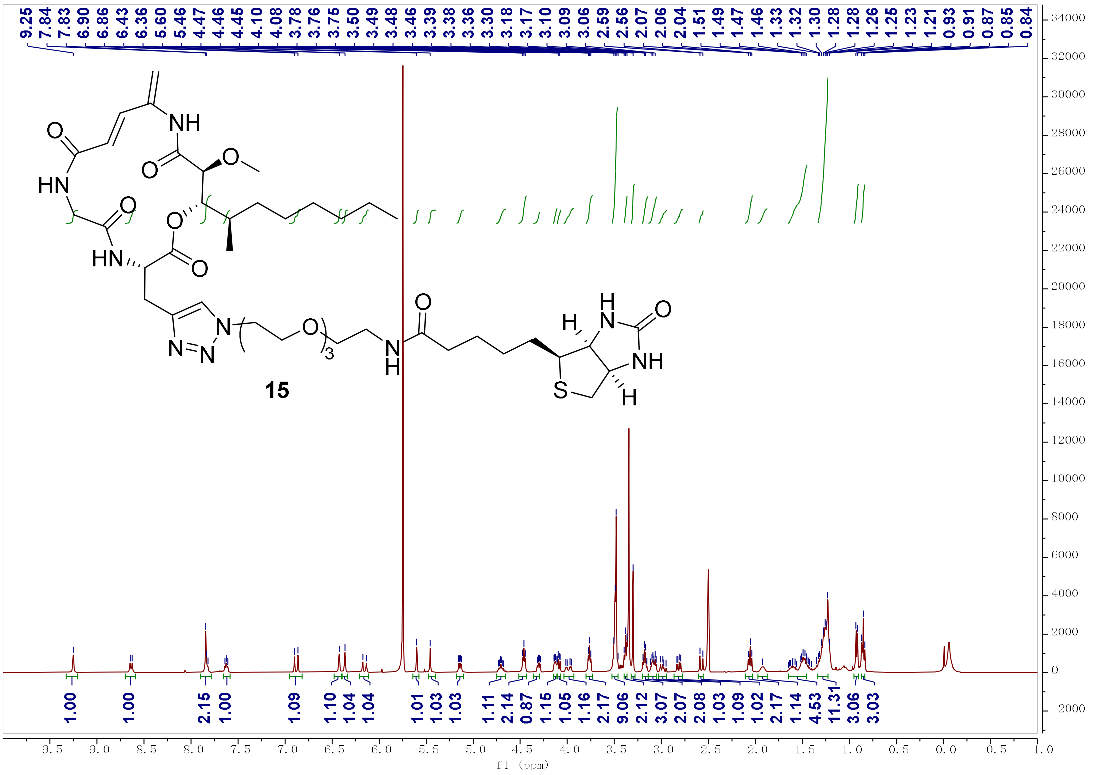

$^{13}$C NMR spectrum of **15**

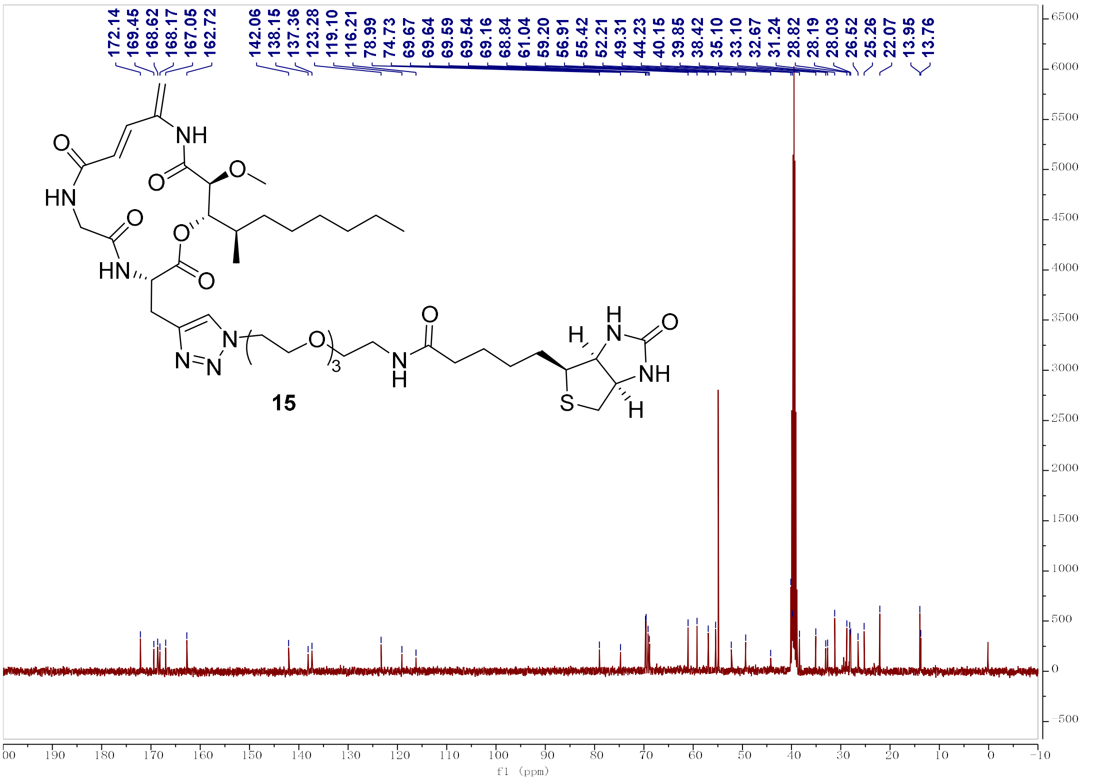

$^{1}$H NMR spectrum of **16**

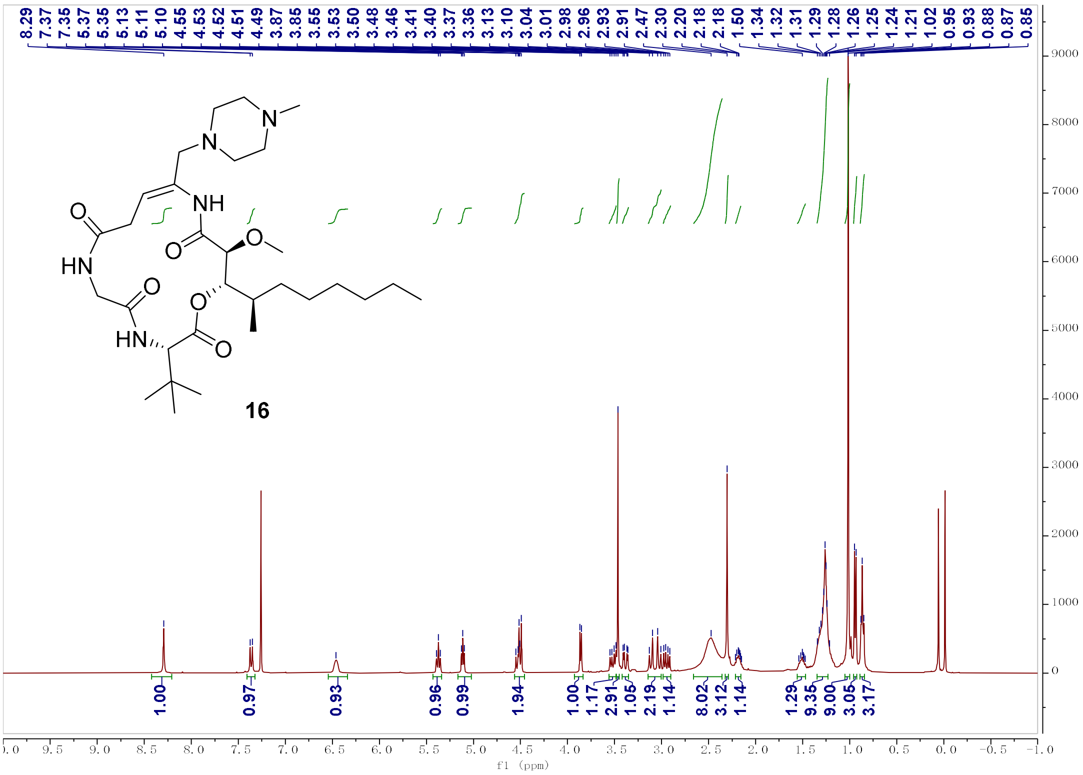

$^{13}$C NMR spectrum of **16**

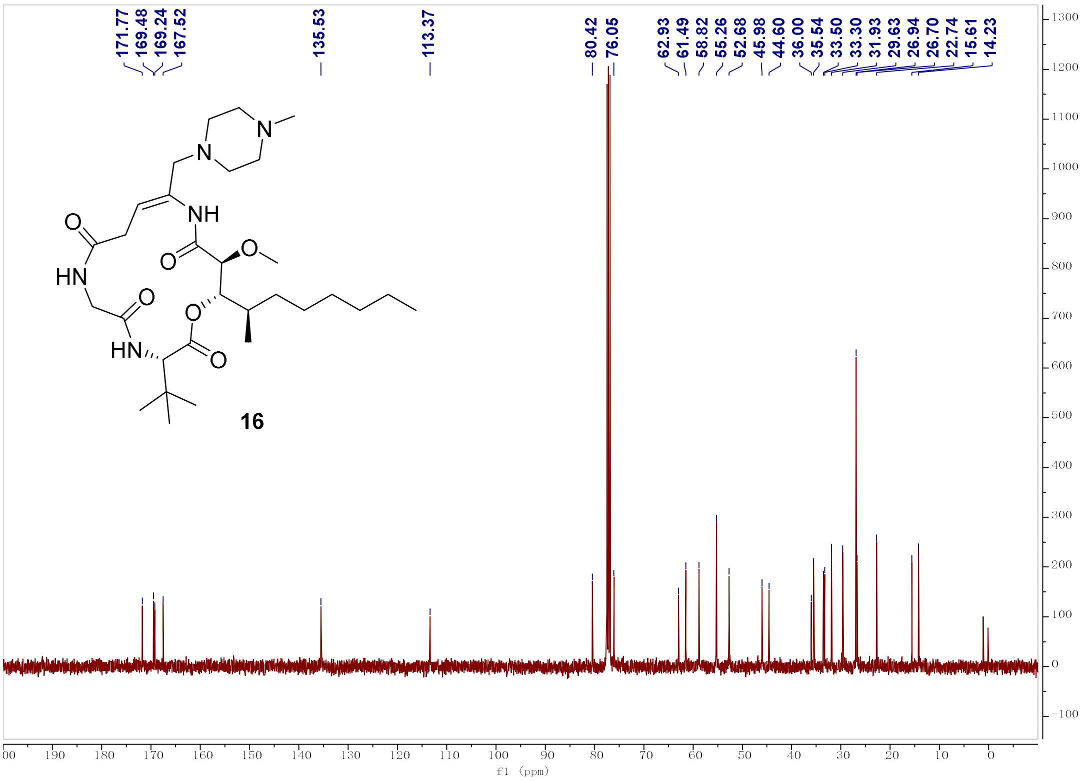

## 4 HPLC spectra.

All tested compounds with a purity of > 95% were used for subsequent assays. We provided the spectra of HPLC assays as below.

Column: MicroPulite XP (Tc18, 3μm, 2.1 mm × 50 mm);

Mobile phase: MeCN/$H_2O$;

Detector: UV detector       Wavelength: 254 nm;

Flow rate: 1.5 mL/min;

Temperature: 30 °C.

**1a**

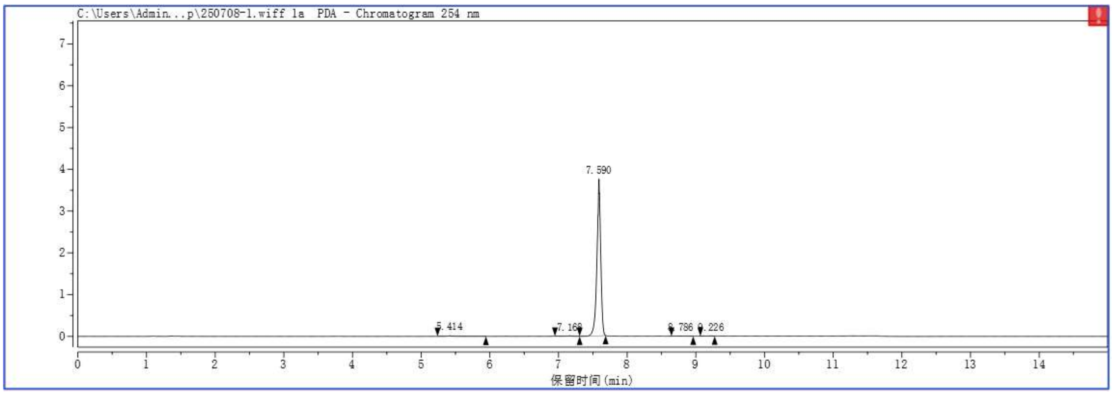


| No. | Retention time (min) | Area | Area% | Peak height |
|---|---|---|---|---|
| 1 | 9.226 | 0.363 | 0.13 | 0.004 |
| 2 | 8.786 | 0.329 | 0.11 | 0.004 |
| 3 | 7.590 | 284.705 | 99.12 | 3.750 |
| 4 | 7.168 | 0.666 | 0.23 | 0.010 |
| 5 | 5.414 | 1.162 | 0.40 | 0.014 |

## 1b

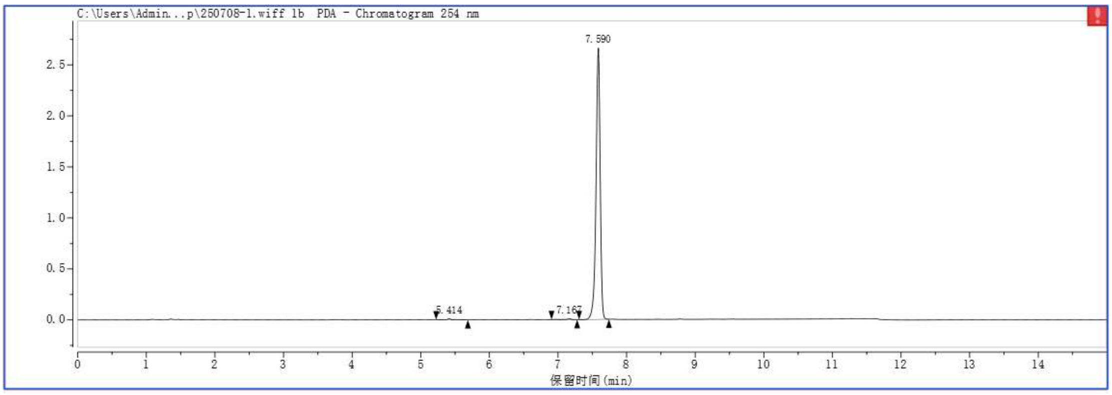


| No. | Retention time (min) | Area | Area% | Peak height |
|---|---|---|---|---|
| 1 | 7.590 | 2.660 | 223.197 | 99.27 |
| 2 | 7.167 | 0.009 | 0.752 | 0.33 |
| 3 | 5.414 | 0.012 | 0.888 | 0.40 |

## 1c

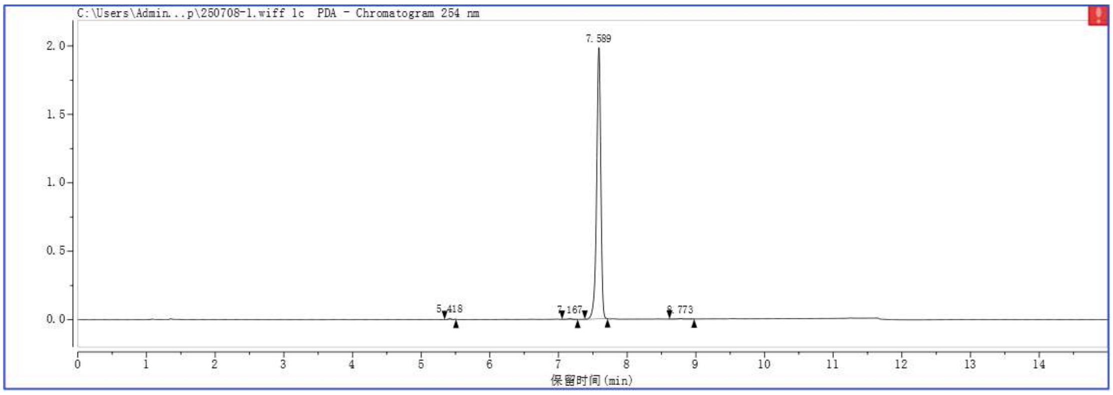


| No. | Retention time (min) | Area | Area% | Peak height |
|---|---|---|---|---|
| 1 | 8.773 | 0.214 | 0.13 | 0.003 |
| 2 | 7.589 | 167.276 | 99.39 | 1.982 |
| 3 | 7.167 | 0.439 | 0.26 | 0.006 |
| 4 | 5.418 | 0.380 | 0.23 | 0.009 |

**1d**

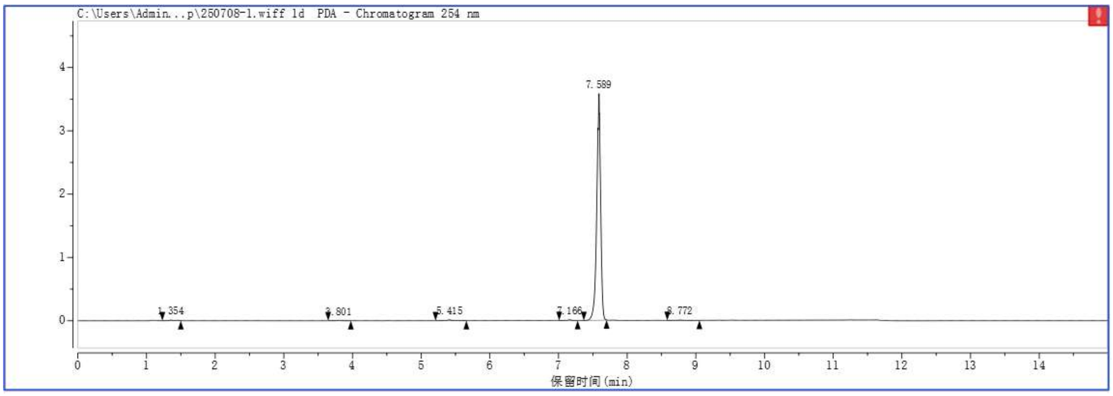


| No. | Retention time (min) | Area | Area% | Peak height |
|---|---|---|---|---|
| 1 | 8.772 | 0.365 | 0.13 | 0.004 |
| 2 | 7.589 | 280.236 | 98.80 | 3.579 |
| 3 | 7.166 | 0.704 | 0.25 | 0.011 |
| 4 | 5.415 | 1.133 | 0.40 | 0.014 |
| 5 | 3.801 | 0.259 | 0.09 | 0.003 |
| 6 | 1.354 | 0.942 | 0.33 | 0.009 |

**1e**

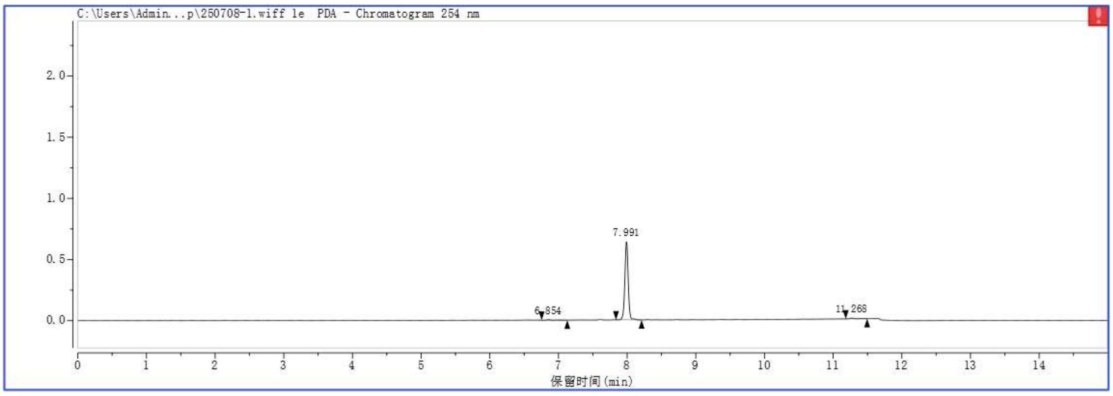


| No. | Retention time (min) | Area | Area% | Peak height |
|---|---|---|---|---|
| 1 | 11.268 | 0.407 | 0.93 | 0.006 |
| 2 | 7.991 | 43.195 | 98.28 | 0.639 |
| 3 | 6.854 | 0.351 | 0.80 | 0.004 |

**1f**

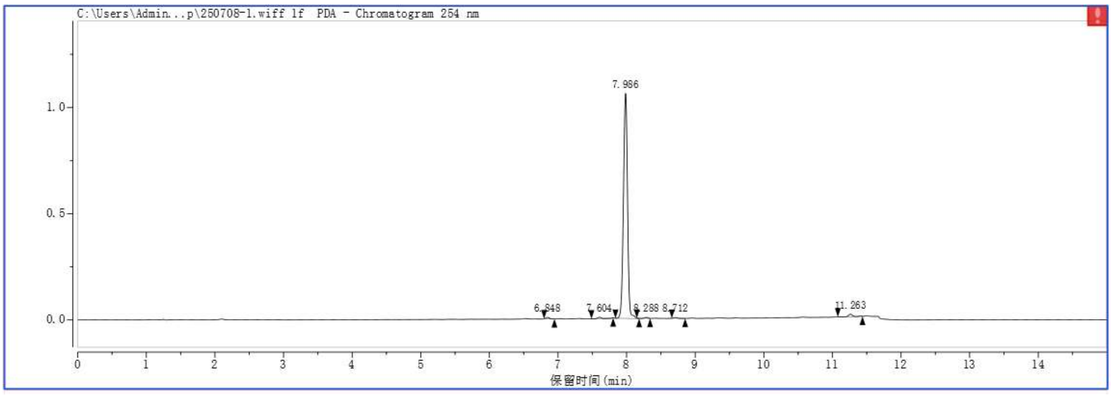


| No. | Retention time (min) | Area | Area% | Peak height |
|---|---|---|---|---|
| 1 | 11.263 | 0.858 | 0.96 | 0.011 |
| 2 | 8.712 | 0.234 | 0.26 | 0.004 |
| 3 | 8.288 | 0.271 | 0.30 | 0.005 |
| 4 | 7.986 | 87.035 | 97.60 | 1.059 |
| 5 | 7.604 | 0.368 | 0.41 | 0.006 |
| 6 | 6.848 | 0.409 | 0.46 | 0.006 |

**1g**

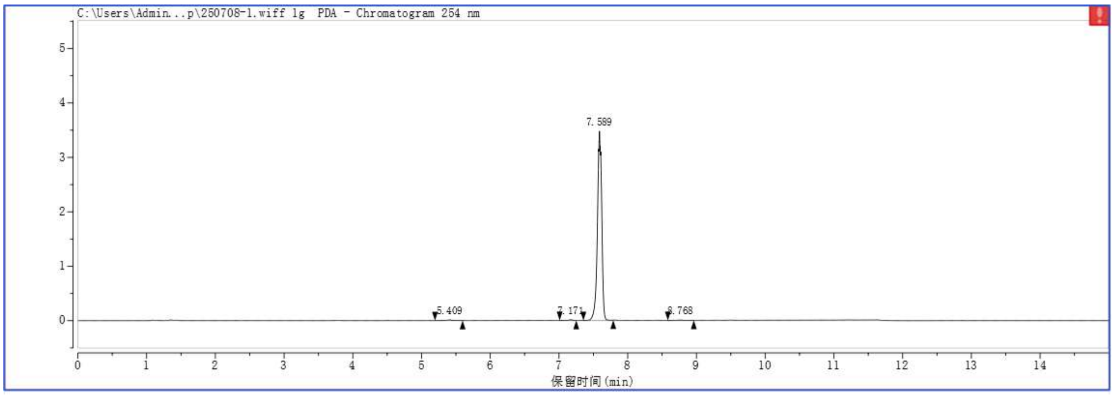


| No. | Retention time (min) | Area | Area% | Peak height |
|---|---|---|---|---|
| 1 | 8.768 | 0.454 | 0.15 | 0.006 |
| 2 | 7.589 | 301.938 | 99.23 | 3.476 |
| 3 | 7.171 | 0.758 | 0.25 | 0.012 |
| 4 | 5.409 | 1.127 | 0.37 | 0.014 |

**1h**

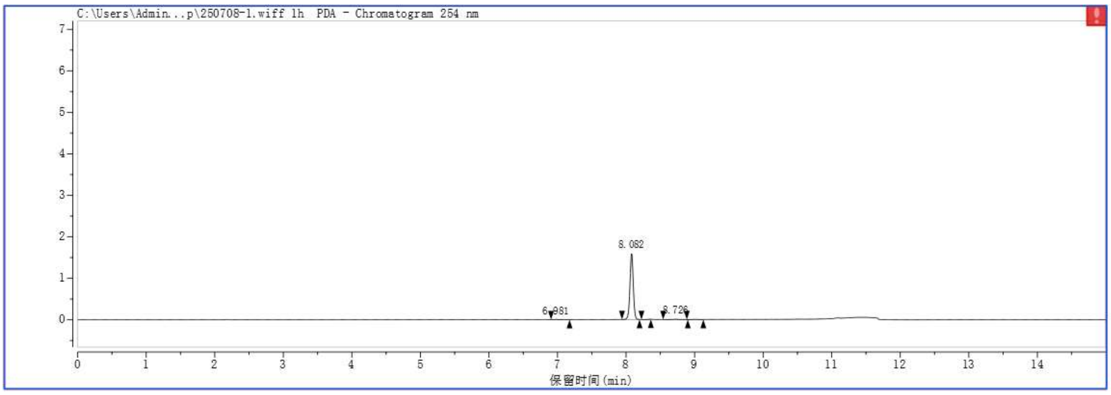


| No. | Retention time (min) | Area | Area% | Peak height |
|---|---|---|---|---|
| 1 | 9.012 | 0.324 | 0.30 | 0.005 |
| 2 | 8.726 | 0.642 | 0.59 | 0.007 |
| 3 | 8.342 | 0.039 | 0.04 | 0.001 |
| 4 | 8.082 | 106.809 | 98.74 | 1.587 |
| 5 | 6.981 | 0.352 | 0.33 | 0.005 |

**1i**

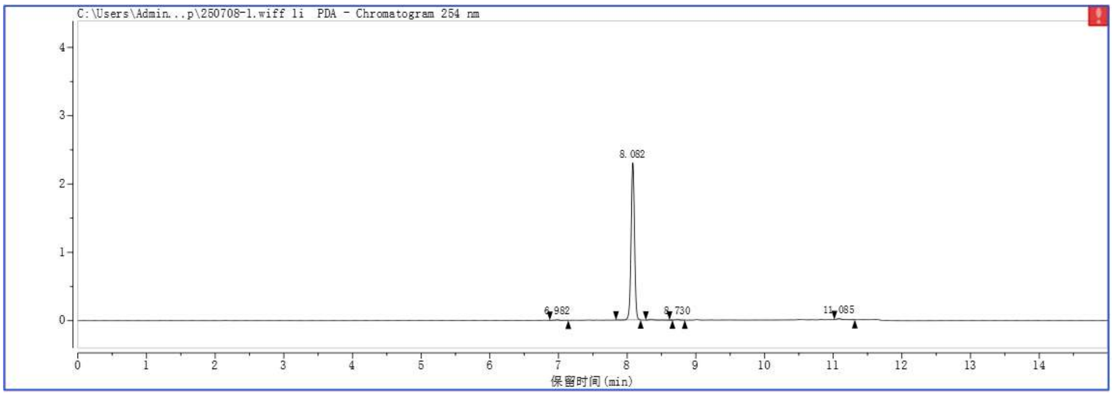


| No. | Retention time (min) | Area | Area% | Peak height |
|---|---|---|---|---|
| 1 | 11.085 | 1.366 | 0.84 | 0.017 |
| 2 | 8.730 | 0.855 | 0.52 | 0.011 |
| 3 | 8.348 | 0.937 | 0.57 | 0.011 |
| 4 | 8.082 | 159.440 | 97.62 | 2.298 |
| 5 | 6.982 | 0.729 | 0.45 | 0.009 |

**1j**

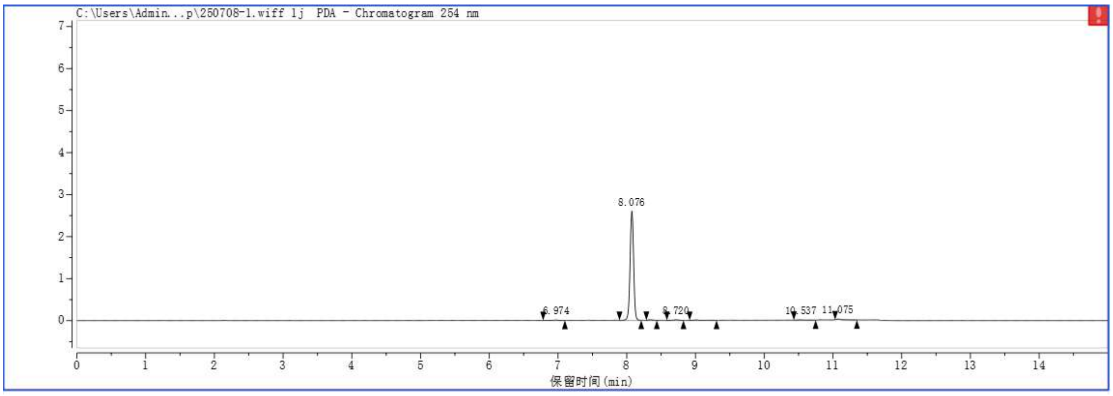


| No. | Retention time (min) | Area | Area% | Peak height |
|---|---|---|---|---|
| 1 | 11.075 | 1.061 | 0.56 | 0.017 |
| 2 | 10.537 | 0.836 | 0.44 | 0.006 |
| 3 | 9.006 | 0.848 | 0.44 | 0.009 |
| 4 | 8.720 | 1.082 | 0.57 | 0.013 |
| 5 | 8.338 | 0.924 | 0.48 | 0.012 |
| 6 | 8.076 | 185.276 | 97.16 | 2.603 |
| 7 | 6.974 | 0.664 | 0.35 | 0.010 |

**1k**

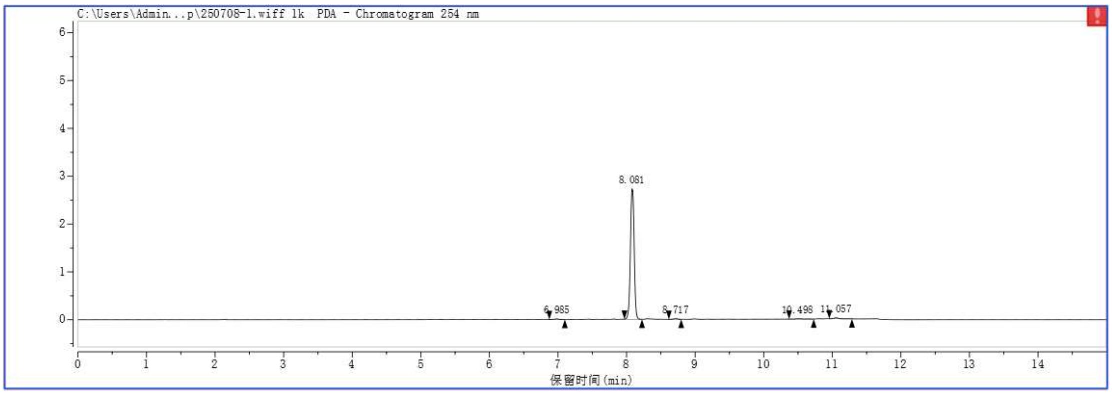


| No. | Retention time (min) | Area | Area% | Peak height |
|---|---|---|---|---|
| 1 | 11.057 | 1.697 | 0.80 | 0.023 |
| 2 | 10.498 | 0.913 | 0.43 | 0.007 |
| 3 | 8.717 | 1.129 | 0.53 | 0.016 |
| 4 | 8.081 | 207.573 | 97.79 | 2.726 |
| 5 | 6.985 | 0.950 | 0.45 | 0.013 |

**1l**

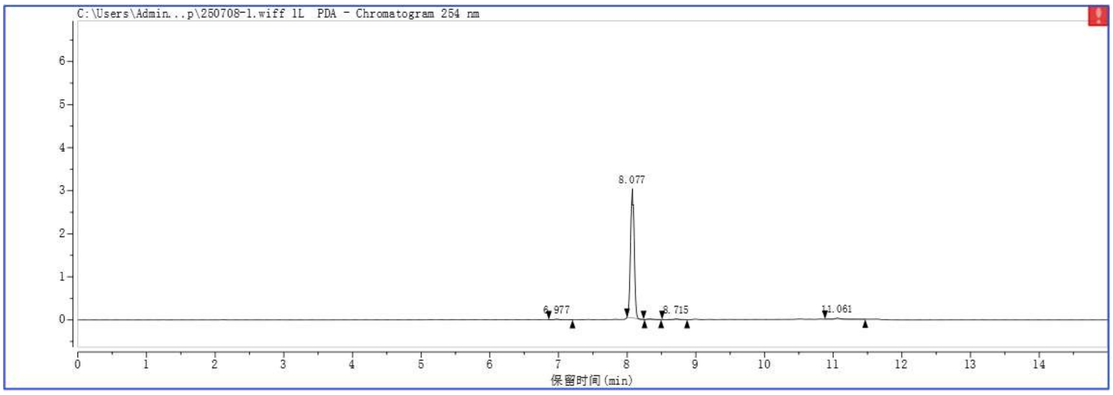


| No. | Retention time (min) | Area | Area% | Peak height |
|---|---|---|---|---|
| 1 | 11.061 | 2.625 | 1.15 | 0.028 |
| 2 | 8.715 | 1.409 | 0.62 | 0.018 |
| 3 | 8.321 | 1.335 | 0.59 | 0.014 |
| 4 | 8.077 | 221.281 | 97.14 | 2.998 |
| 5 | 6.977 | 1.156 | 0.51 | 0.014 |

**1m**

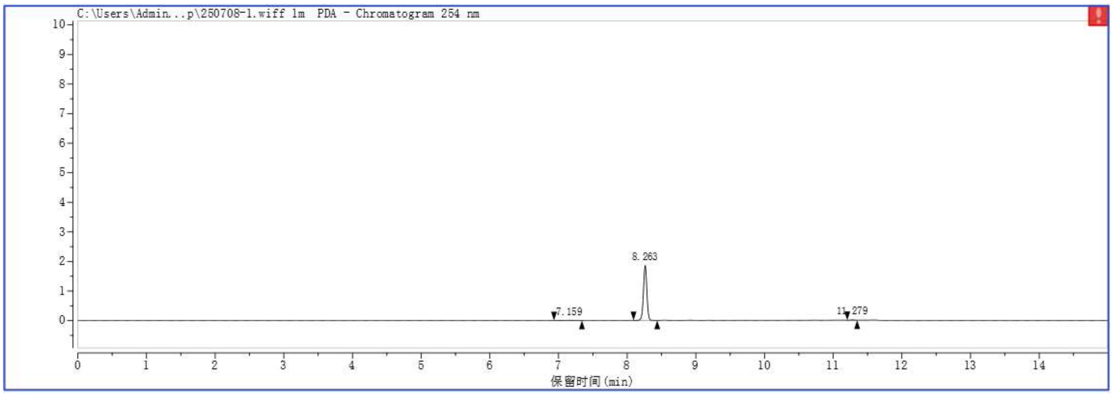


| No. | Retention time (min) | Area | Area% | Peak height |
|---|---|---|---|---|
| 1 | 11.279 | 2.533 | 1.94 | 0.011 |
| 2 | 8.263 | 127.443 | 97.76 | 1.854 |
| 3 | 7.159 | 0.384 | 0.29 | 0.005 |

**14**

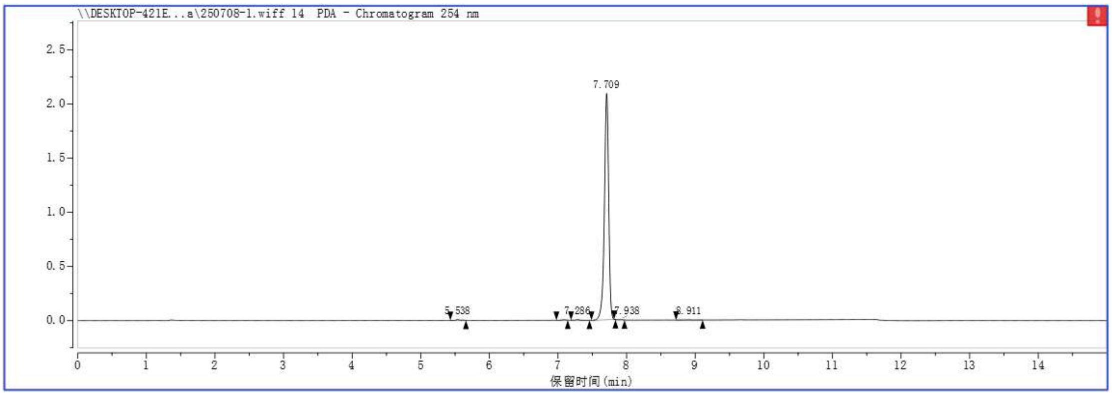


| No. | Retention time (min) | Area | Area% | Peak height |
|---|---|---|---|---|
| 1 | 8.911 | 0.123 | 0.07 | 0.002 |
| 2 | 7.938 | 0.008 | 0.00 | 0.000 |
| 3 | 7.709 | 182.083 | 99.20 | 2.089 |
| 4 | 7.286 | 0.382 | 0.21 | 0.006 |
| 5 | 7.093 | 0.337 | 0.18 | 0.005 |
| 6 | 5.538 | 0.610 | 0.33 | 0.009 |

**15**

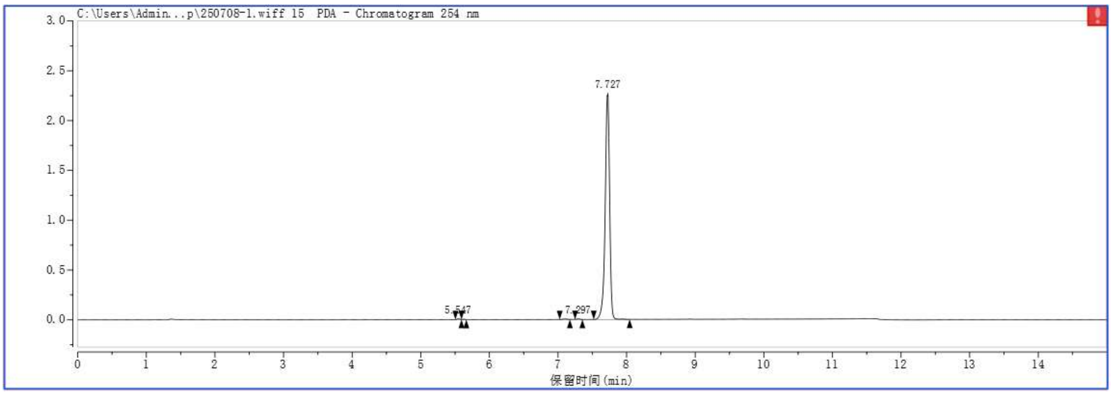


| No. | Retention time (min) | Area | Area% | Peak height |
|---|---|---|---|---|
| 1 | 7.727 | 204.320 | 97.86 | 2.269 |
| 2 | 7.297 | 1.268 | 0.61 | 0.007 |
| 3 | 7.106 | 1.442 | 0.69 | 0.006 |
| 4 | 5.630 | 0.424 | 0.20 | 0.003 |
| 5 | 5.547 | 1.328 | 0.64 | 0.009 |

**16**

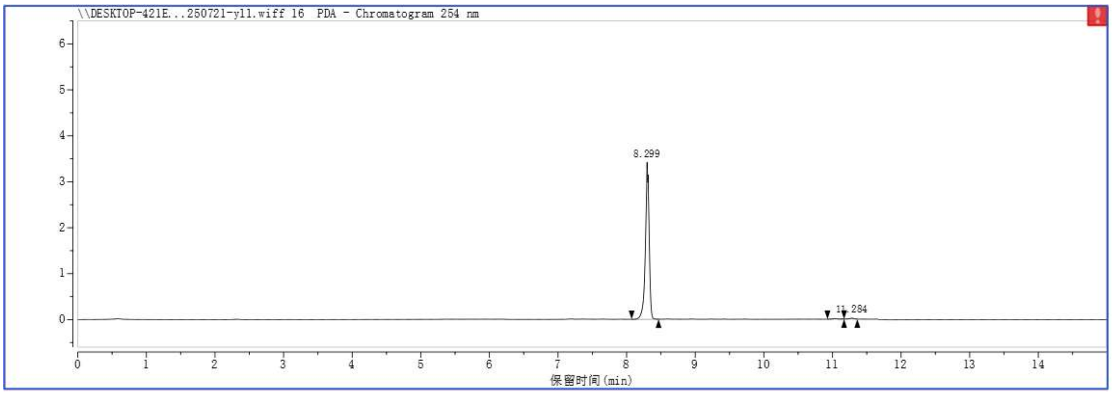


| No. | Retention time (min) | Area | Area% | Peak height |
|---|---|---|---|---|
| 1 | 11.284 | 1.610 | 0.62 | 0.021 |
| 2 | 11.037 | 1.123 | 0.43 | 0.014 |
| 3 | 8.299 | 255.845 | 98.94 | 3.413 |

**BA**

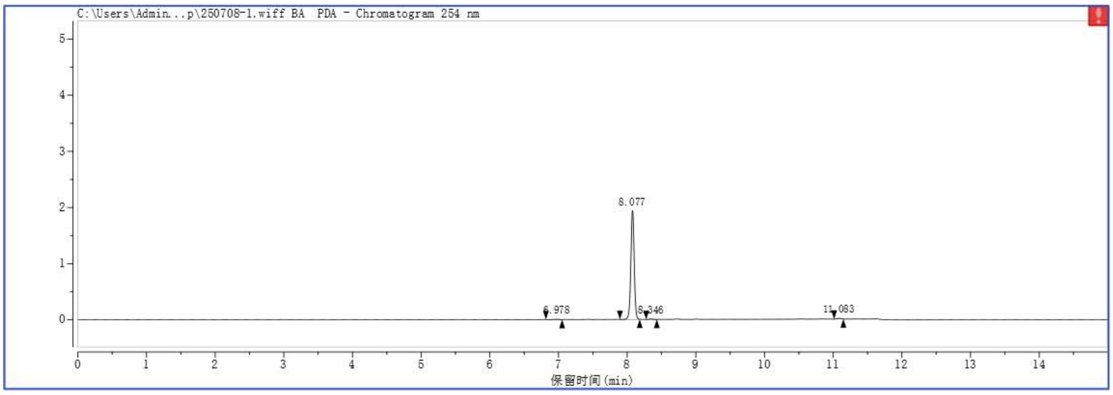


| No. | Retention time (min) | Area | Area% | Peak height |
|---|---|---|---|---|
| 1 | 11.083 | 2.783 | 2.03 | 0.013 |
| 2 | 8.346 | 2.100 | 1.53 | 0.008 |
| 3 | 8.077 | 131.583 | 96.07 | 1.938 |
| 4 | 6.978 | 0.506 | 0.37 | 0.007 |
